# Unravelling the impact of SARS-CoV-2 on hemostatic and complement systems: a systems immunology perspective

**DOI:** 10.3389/fimmu.2024.1457324

**Published:** 2025-01-13

**Authors:** Didar Murad, Rehan Zafar Paracha, Maryum Nisar

**Affiliations:** School of Interdisciplinary Engineering and Sciences (SINES), Department of Sciences, National University of Sciences and Technology (NUST), Islamabad, Pakistan

**Keywords:** hemostatic system, complement system, systems immunology, modeling, ordinary differential equations, COVID-19

## Abstract

The hemostatic system prevents and stops bleeding, maintaining circulatory integrity after injury. It directly interacts with the complement system, which is key to innate immunity. In coronavirus disease 2019 (COVID-19), dysregulation of the hemostatic and complement systems has been associated with several complications. To understand the essential balance between activation and regulation of these systems, a quantitative systems immunology model can be established. The dynamics of the components are examined under three distinct conditions: the disease state representing symptomatic COVID-19 state, an intervened disease state marked by reduced levels of regulators, and drug interventions including heparin, tranexamic acid, avdoralimab, garadacimab, and tocilizumab. Simulation results highlight key components affected, including thrombin, tissue plasminogen activator, plasmin, fibrin degradation products, interleukin 6 (IL-6), the IL-6 and IL-6R complex, and the terminal complement complex (C5b-9). We explored that the decreased levels of complement factor H and C1-inhibitor significantly elevate these components, whereas tissue factor pathway inhibitor and alpha-2-macroglobulin have more modest effects. Furthermore, our analysis reveals that drug interventions have a restorative impact on these factors. Notably, targeting thrombin and plasmin in the early stages of thrombosis and fibrinolysis can improve the overall system. Additionally, the regulation of C5b-9 could aid in lysing the virus and/or infected cells. In conclusion, this study explains the regulatory mechanisms of the hemostatic and complement systems and illustrates how the biopathway machinery sustains the balance between activation and inhibition. The knowledge that we have acquired could contribute to designing therapies that target the hemostatic and complement systems.

## 1 Introduction

The coronavirus disease 2019 (COVID-19) is a highly contagious illness caused by the severe acute respiratory syndrome coronavirus 2 (SARS-CoV-2) (1). SARS-CoV-2 infection has caused a global catastrophe, leading to over 776 million confirmed cases and over seven million deaths globally (2). The body’s response to COVID-19 can involve a sophisticated interaction between several systems, including the hemostatic system (HS) and the complement system (CS). In hemostasis, activation of the coagulation cascade plays a major role in preventing bleeding from a blood vessel (3). In COVID-19, activation of the hemostatic and complement pathways leads to immunothrombosis (4). Immunothrombosis refers to the body immune response where the activation of the coagulation system intersects with the immune system to form clots (thrombi) in blood vessels, particularly in response to infection or injury (5, 6). In severe COVID-19, hyperinflammatory response (7) and hypercoagulation (8) cause serious complications that include acute respiratory distress syndrome (ARDS), stroke, heart attack, and kidney failure (9–12). The coagulation cascade is supported by the complement cascade, a key component of innate immunity. Activation of the complement cascade is necessary for the elimination of foreign pathogens through mechanisms such as opsonization with opsonins IgG, C3b, and C4b, and inflammation via anaphylatoxins C3a and C5a (9, 13). In SARS-CoV-2 infection, excessive activation of the complement cascade leads to increased levels of potent mediators of inflammation, including C3a and C5a. This results in cytokine storms and ultimately multiple organ injury (9).

The exact mechanisms by which SARS-CoV-2 infection leads to immunothrombosis are still under investigation (14–16). However, it is believed that the virus can damage the blood vessels lining cells, which can trigger the formation of clots. In COVID-19, interleukin 6 (IL-6) causes elevated levels of fibrinogen and tissue factor (TF). These hemostatic factors contribute to an increased potential for fibrin clot formation (6). The epidemiological effects of COVID-19 include a high mortality rate in some patients, particularly those with elevated levels of fibrinolytic factors (17).

Recent advancements in systems biology have opened doors to a new frontier in understanding complex biological processes (18–20). The holistic approach in systems biology is crucial for uncovering mechanisms underlying diseases, including COVID-19, and for developing targeted therapies (21). Within this field, systems immunology leverages mathematical and computational methods to explore the intricate interactions within the cellular and molecular networks of the immune system (22).

The previous systems immunology models have lacked adequate detail for a thorough and comprehensive description of the pathway. The first attempts at mathematical framework of the system included simplified descriptions of the complex dynamics in key aspects of complement activation such as the amplification loop (23) in the alternative pathway and formation of the MAC. Korotaevskiy et al. (24) developed a combined mathematical model of classical, alternative, and terminal pathways of the complement system; however, the impact of complement regulators and the differentiation between pathway activation on the cell surface versus in the plasma were not included. Subsequent studies introduced more comprehensive models integrating the alternative, classical, and lectin pathways (25–29). These models explore the complement response to pathogens such as *N. meningitidis*. However, they rely on simplified representations of regulatory dynamics, which fail to fully capture all regulatory effects. Recent advancements, such as Loveleena et al. (29), introduced a quantitative systems pharmacology model of the complement system to evaluate potential drug targets for inhibiting complement activation in autoimmune diseases. The model describes complement activation via the alternative and terminal pathways and the dynamics of several regulatory proteins. In our previous study (9), a kinetic logic model for SARS-CoV-2-induced complement pathways was established. The responses of different complement proteins, their regulators, and proinflammatory cytokines were observed under various conditions in COVID-19.

**Figure 1:**
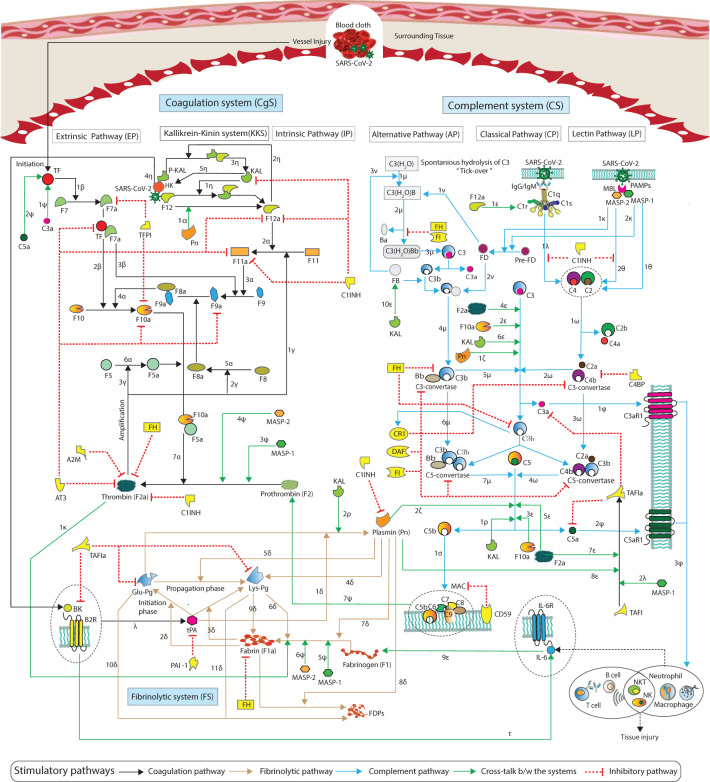
Schematic representation of the cross-talk between the pathways of the hemostatic and complement systems. The hemostatic system and complement system interactions are represented by arrows ${\;}^{\prime \prime }\to {\;}^{\prime \prime }$ , with blue and black arrows indicating complement and coagulation stimulatory pathways, green arrows representing cross-talk pathway activities, and the red blunt edge ${\;}^{\prime \prime }⊣{\;}^{\prime \prime }$ indicating an inhibitory mechanism. Details of the interactions, along with references, are provided in Materials and methods (see Sections 2.1.1–2.1.3).

Existing mathematical models of thrombin generation, along with their shortcomings in the coagulation cascade, were recently reported by Owen et al. (30) They highlighted missing interactions in these models, particularly those involving the tissue factor (TF) pathway, the common pathway, coagulation factors, and key regulators such as antithrombin (AT) and tissue factor pathway inhibitor (TFPI). A few studies have previously reported on mathematical modeling of the interactions between the coagulation and complement cascades (31, 32). As far as we know, no computational models exist that involve the enhancement and suppression mechanisms regulating hemostatic and complement activities. We elucidated the regulatory mechanisms governing the hemostatic and complement systems, demonstrating how these biopathways maintain the balance between activation and inhibition. The developed model effectively addresses the dynamics of factors involved in both systems, which are critical components of the immunothrombosis process in COVID-19. The insights gained from this research may aid in the development of therapies targeting these systems: the HS, including the coagulation and fibrinolytic systems, and CS comprising the lectin pathway (LP), classical pathway (CP), and alternative pathway (AP) cascades. The complement pathways can increase TF activity, thereby triggering the extrinsic pathway (EP) and leading to the generation of thrombin, furthermore stimulating the intrinsic pathway (IP) and the fibrinolytic pathway. The coagulation pathways also influence the activity of complement factors (see **Figure 1**).

Given this background, we constructed a comprehensive systems immunology model of the hemostatic and complement systems, comprising a system of ordinary differential equations (ODEs). The model is simulated to analyze the concentration dynamics of HS and CS entities in response to SARS-CoV-2 infection. These entities include thrombin (F2a), plasmin (Pn), tissue plasminogen activator (tPA), fibrin degradation products (FDPs), and the terminal complement complex (TCC), also known as the membrane attack complex (MAC; C5b-9). They are affected as a result of impairment in inhibitors such as complement factor H (FH), C1-inhibitor (C1INH), tissue factor pathway inhibitor (TFPI), and alpha-2-macroglobulin (A2M). Moreover, the concentration dynamics of IL-6 and the IL-6 and IL-6R complex are observed to estimate inflammation response. The dynamics of the entities are observed under three distinct conditions: the disease state, the intervened disease state (perturbation), and the treatment state (intervened disease state with treatment). In this model, the disease state represents an unhealthy, symptomatic COVID-19 condition, while the intervened disease state reflects impairments in FH, C1INH, TFPI, and A2M. The treatment state incorporates drug interventions, including heparin, tranexamic acid (TXA), avdoralimab, garadacimab, and tocilizumab (TCZ). The impacts of these inhibitors on hemostatic and complement activators are analyzed using global sensitivity analysis (GSA). Furthermore, the effects of kinetic parameters (kinetic constants) on these activators are also examined.

## 2 Materials and methods

The study workflow includes the exploration of the hemostatic and complement systems using information from the Kyoto Encyclopedia of Genes and Genomes (KEGG) database (33) and literature source. For both systems, a biochemical model governed by ODEs has been developed in MATLAB SimBiology. The model is parameterized using kinetic parameters and initial concentration levels of the model entities as initial conditions. The parameters are obtained from previous studies, with some being estimated. The microarray gene expression analysis is performed. The initial concentration levels (initial conditions) of the entities are derived from microarray gene expression data, and some are adopted from previous experimental studies. In the analysis and evaluation, the model is simulated to observe the dynamic concentration levels of the entities. These levels are evaluated under three conditions: (i) the disease state; (ii) the intervened disease state, involving a decrease in the levels of specific inhibitors due to their impairment in SARS-CoV-2 infection, specifically, the concentrations of TFPI, C1INH, FH, and A2M are reduced to 60-, 30-, 2-, and 2-folds of the disease state levels; and (iii) treatment state, incorporating adopted drugs with specific targets. In the therapy modeling strategy, the drugs, dosage amounts, initiation times, rates, intervals, and repeated counts are considered. Additionally, we conducted a variance-based global sensitivity analysis (GSA). The GSA is performed for species-to-species and species-to-kinetic parameters. See the ODE-based modeling framework in **Supplementary File S1**.

### 2.1 Hemostatic and complement systems regulatory network

A schematic representation of the cascades of the hemostatic system (HS) and complement system (CS) is shown in **Figure 1**. Information regarding the kinetic parameters and interactions of the HS and CS entities is provided in **Supplementary Table S1**, along with corresponding references. A brief introduction to each system pathway is presented here.

#### 2.1.1 Hemostatic system

The coagulation system (CgS) comprises two pathways: the extrinsic pathway (EP) and the intrinsic pathway (IP). The intrinsic cascade is known as the contact system or kallikrein-kinin system (KKS) (34). The IP is less important for initiating coagulation than EP. However, the IP is important for the amplification of the cascade. The EP is initiated when tissue factor (TF) is released from the damaged tissue or vessel injury (**Figure 1**). Interactions labeled with ${\;}^{\prime \prime }1\beta^{\prime \prime }$ represent the TF interacts with pro-convertin (F7) and converts it to the active form F7a (35). Interaction marked as ${\;}^{\prime \prime }2\beta^{\prime \prime }$ represents a complex TFF7a that activate F10 to F10a, which has a pivotal role in the coagulation cascade. Similarly, the interaction ${\;}^{\prime \prime }3\beta^{\prime \prime }$ indicates that TFF7a activates antihemophilic factor B (F9) to F9a. Thrombokinase (F10a) is the point at which the EP and IP of the coagulation cascade meet. The IP is initiated upon the activation of coagulation factor F12 by KAL. The association of F12 with KAL results in the product KAL:F12 ( ″1η″ ), which can generate F12a (36). The interaction denoted as ″2η″ indicates that F12a binds and activates pre-kallikrein (P-KAL) (36), leading to the formation of the complex P-KAL:F12a. This complex can further produce KAL ( ″3η″ ) (35, 37). The factor F12 can directly bind to F12a, resulting in the formation of F12:F12a as a product (35). This process involves auto-catalysis and autoactivation, amplifying the expression level of F12a (35, 36). In the amplification process, Pn can increase the levels of F12a ( ″1α″ ). Interactions ″2α″ and ″3α″ represent F12a causing the conversion of F11 to F11a, which, in turn, activates F9 to F9a. The interaction ″4α″ implies that F9a converts F10 to F10a with the help of F8a, an active form of antihemophilic factor A (F8). Interaction ″5α″ represents conversion of F8 to F8a. Interaction ″7α″ represents F10a with the assistance of F5a, converting prothrombin (F2) into F2a. F5a is generated by pro-accelerin (F5). Interaction ″6α″ maintains the conversion of F5 to F5a. The interactions labeled with ″1γ″ , ″2γ″, and ″3γ″ indicate the amplification of F11a, F8a, and F5a, respectively, by F2a (38).

The fibrinolytic system (FS), known as the plasminogen (Pg)–plasmin (Pn) system, is composed of the proteolytic enzyme Pn with its precursor Pg (39). Fibrinolysis is initiated in the presence of tPA, which is involved in the formation of Pn from native plasminogen (Glu-Plasminogen; Glu-Pg) ( ″1δ″ ). The interactions ″2δ″ and ″3δ″ signify that tPA can activate the Pg variants Glu-Pg and Lys-Pg in the presence of F1a (40). The tPA is inhibited by the Pg activator inhibitor (PAI-1) (41). In the amplification of the fibrinolytic cascade, Pn can generate Lys-Pg ( ″4δ″ ). The Pn stimulates its own production in a positive feedback loop (42). The interaction labeled with ″5δ″ represents Pn cleaving Glu-Pg to Lys-Pg (43). Lys-Pg can bind to and cleave fibrinogen (F1) ( ″6δ″ ); it also binds to the surface of F1a, and the complex formation can increase the levels of tPA (43). The association of Lys-Pg to F1a is labeled with ″9δ″ (40). Furthermore, Pn interacts with and cleaves F1 ( ″7δ″ ) and interaction with F1a, resulting in the generation of FDPs ( ″8δ″ ) (44). F1 can be converted to F1a by F2a ( ″1κ″ ), as described by Pieters et al. (45). Glu- and Lys-Pg bind with FDPs (associations labeled with ″10δ″ and ″11δ″ ), and the products are inhibited by activated thrombin-activatable fibrinolysis inhibitor (TAFIa) (46).

#### 2.1.2 Complement system

The complement system (CS) comprises of three pathways including the classical pathway (CP), the lectin pathway (LP), and the alternative pathway (AP) (27, 47, 48). These pathways trigger separately. The CP is activated when C1q binds to the pathogen via antibodies IgG and/or IgM. The LP is activated by the mannose-binding lectin (MBL) binding with MBL-associated serine proteases MASP-1 and MASP-2, which sense pathogen-associated molecular patterns (PAMPs). The AP becomes active through a mechanism called tick-over, which is the spontaneous hydrolysis of C3 to produce C3(H_2_O). The hydrolyzed C3 binds to factor B (FB), forming C3(H_2_O)FB ( ″1μ″ ). Interaction labeled with ″1υ″ represents the cleavage of C3(H_2_O)FB by factor D (FD) to produce C3(H_2_O)Bb ( ″2μ″ ). Further interaction marked as ″3μ″ indicates that the activation and cleavage of C3 results in the formation of C3-convertase C3bBb ( ″4μ″ ). Interactions denoted as ″1λ″ , ″1θ″ , and ″2θ″ represent the involvement of C1qC1rC1s(C1qrs), MASP1, and MASP2 in cleaving C2 and C4. The cleaved fragments assemble into C3-convertase C4bC2a ( ″1ω″ ). The C3-convertases at the central level cleave C3 into C3a and C3b ( ″5μ″ and ″2ω″ ).

All the pathways converge at the level of C3, cleaving it and forming C5-convertases, C4bC2aC3b, and C3bBbC3b ( ″6μ″ and ″3ω″ ). The C5-convertases cleave C5 into C5a and C5b ( ″7μ″ and ″4ω″ ). The proinflammatory anaphylatoxins (peptides) C2a, C3a, and C5a cause inflammation, while opsonins C2b, C3b, and C5b can promote opsonization. Association labeled with ″1σ″ implies that C5b, combined with C6-9, forms C5b-9.

In the activated complement cascade, C3a binds to its receptor C3aR1, and C5a interacts with C5aR1. The interactions C3a-C3aR1 and C5a-C5aR1 are labeled as ″1φ″ and ″2φ″ can induce and promote inflammatory activities. Notably, during SARS-CoV-2 infection, an over-expression of IL-6 was observed due to C3a- and C5a-mediated C3aR1 and C5aR1 levels (49). The expressions of C3aR1 and C5aR1 stimulate leukocytes (inflammatory cells), as demonstrated by the interaction labeled ″3φ″ (9).

These pathways are tightly regulated by downregulators present in serum and on membranes, such as C1INH, factor I (FI), C4 binding protein (C4BP), and FH, which are available in serum. Membrane-bound regulators include decay-accelerating factor (DAF), CR1, and CD59.

#### 2.1.3 Cross-talk between the systems

In the context of cross-reactive cascades, factor F12a can bind with complement component C1r ( ″1ε″ ) (50). The factors F10a and F2a play a critical role in cleaving both central complement C3 and terminal complement C5 ( $\prime \prime 2\varepsilon^{\prime \prime }$ , ″3ε″ , ″4ε″ , and ″5ε″ ). Additionally, Pn cleaves C3 and C5 ( ″1ζ″ and ″2ζ″ ) (51, 52). Furthermore, KAL has also been identified as another factor capable of cleaving C3 and C5 ( ″6ε″ and ″1ρ″ ) (50, 51). The cleaved fragment of C5, such as C5a, functions as a potent complement anaphylatoxin with the ability to induce the expression of tissue factor (TF) (50, 51) and enhance TF activity (53). The cleaved fragment of C3, such as C3a, also increases TF expression (50). The induction of TF by C3a and C5a is represented by interactions marked as ″1Ψ″ and ″2Ψ″ (**Figure 1**).

The entities MASP1 and MASP2 play a pivotal role in converting F2 into F2a ( ″3Ψ″ and ″4Ψ″ ) and converting F1 into F1a ( ″5Ψ″ and ″6Ψ″ ) (53, 54). Additionally, the activation of TAFI to TAFIa by MASP1 ( ″2λ″ ) (55), leads to the interaction of TAFIa with C3a and C5a, effectively suppressing their activity.

In severe cases of COVID-19, the activation of KAL through factor F12a can lead to the generation of BK from high molecular weight kininogen (HK), resulting in hyperinflammation and an increased production of IL-6 (56). The association of P-KAL with HK, resulting in P-KAL-HK complex formation (57), which leads to the production of the proinflammatory peptide (bradykinin; BK), the interaction is labeled with ″4η″ . The complex formed by BK and B2R (BK-B2R) can stimulate tPA, as indicated by the interaction labeled ″λ″ (56). Additionally, BK-B2R stimulates the production of IL-6, as indicated by the interaction labelled ″τ″ . F1 expression can be induced by IL-6 ( ″9ε″ ) (58). Its levels are correlated with IL-6 levels (59).

In the initiation and propagation of the FS, tPA and KAL play a significant role (7). The involvement of KAL in the interaction between Glu-Pg and Pn is indicated by the interaction marked as ″2ρ″ (60). The membrane attack complex (C5b-9) interacts with F2 and can elevate its levels ( ″7Ψ″ ) (51). The resulting activation of F2a and Pn subsequently leads to the activation of TAFI to TAFIa ( ″7ε″ and ″8ε″ ) (42), and TAFIa in turn inactivates BK (61).

The inhibitor C1INH binds to and inhibits the factors F12a, F11a, F2a, and Pn, respectively (53, 62). It can also interact with and suppress KAL (50, 51, 62). Additionally, FH exerts inhibitory effects on F2a and F1a, displaying a strong binding affinity with these components (63).

#### 2.1.4 SARS-CoV-2 interaction with hemostatic and complement systems

The molecular processes observed during infection are significantly influenced by the interaction between the structural proteins of SARS-CoV-2 and various factors from the HS and CS. The four structural proteins of SARS-CoV-2, namely, spike (S), nucleocapsid (N), membrane (M), and envelope (E) proteins, have been shown to bind to these factors, including gC1qR, F12, and HK (7, 64). Additionally, the N-protein interaction with MASP2 potentiates the activation of the CS (17). Notably, direct cleavage of the S-protein by F2a and F10a has also been observed (65).

### 2.2 Modeling of the hemostatic and complement systems

#### 2.2.1 Mathematical model formalism

The signaling pathways of the HS and CS, as depicted in **Figure 1**, served as the foundation for establishing a biochemical reaction network governed by a system of ODEs as a mathematical model. The variables (species) {*s_i_*: i=1 to n} of the ODEs denote the concentration level of bio-molecular entities (species and complexes). The kinetic parameters {*k_i_*: i=1 to m} represent the biochemical reaction rate constants. The general representation of the model is given in Equation 1:


(1) $$\frac{ds_{i}}{dt}=\sum_{j=1}^{n_{i}}r_{j}m_{ij}f_{j}$$

where $\prime \prime n_{i}\prime \prime$ is the number of reactions linked with the species $\prime \prime s_{i}\prime \prime$ . $\prime \prime r_{j}\prime \prime$ = −1 if $\prime \prime s_{i}\prime \prime$ is a reactant and $\prime \prime r_{j}\prime \prime$ = 1 if $\prime \prime s_{i}\prime \prime$ is a product of the jth reaction. The quantities $\prime \prime m_{ij}\prime \prime$ indicate the stoichiometric coefficients. By law of mass action, the function $\prime \prime f_{j}\prime \prime$ can be written in the form $f_{j}=k_{\alpha }s_{a}$ or $f_{j}=k_{\alpha }s_{b}$ or $f_{j}=k_{\alpha }s_{a}s_{b}$ . By law of Michaelis–Menten $f_{j}=\frac{k_{\alpha }s_{a}s_{b}}{\left(k_{\beta }+s_{b}\right)}$ , such that $k_{\alpha }=k_{cats_{b}}^{s_{a}}$ and $k_{\beta }=k_{ms_{b}}^{s_{a}}$ with a,b ∈ {1,2,3,…,n}, and α,β ∈ {1,2,3,…,m} representing the corresponding reaction rates (66). A dummy mathematical model with a governing ODEs system is described in **Supplementary File S2**.

The model is established using MATLAB-SimBiology, calibrated with parameters from **Supplementary Table S1** and provided with initial conditions listed in **Supplementary Table S2**. Specific drugs, along with their targets, are listed in **Supplementary Table S3**. The model comprises 192 ODEs (see **Supplementary File S3** and **Supplementary File S4**). The entities in the model are described in **Supplementary File S5**.

#### 2.2.2 Kinetic parameters

The kinetic parameters, along with the pieces of evidence and references, are provided in **Supplementary Table S1**. The parameters are explored in the literature; some are estimated based on functional and/or structural homologous proteins, and a few were randomly assumed within a certain range. For example, the estimation of decay rates for CS entities, such as the downregulation of functionally homologous protein C3-convertase (fC3bBb) by FH, is assumed based on the decay of C5-convertase (C3bBbC3b) by the inhibitor FH (26). The estimation procedures for parameters, as described in Link et al. (67) and Chatterjee et al. (35), are being employed.

#### 2.2.3 Initial conditions

For the initial conditions (initial concentration levels) of the model entities, we performed gene expression analysis. The gene expression data were obtained from a microarray dataset of SARS-CoV-2-infected individuals in Pakistan, with samples collected from multiple cities, representing diverse populations. The *Homo sapiens* dataset (source: GEO accession GSE177477) is not influenced by any drug or therapy. The dataset consisted of 47 samples, with 11 samples from symptomatic COVID-19 patients, 18 from asymptomatic COVID-19 patients, and 18 from normal individuals.

In this study, both COVID-19 symptomatic and normal (healthy) cases are considered. The workflow for gene expression analysis includes expression data quantification, quality control normalization, clustering analysis, and differential expression analysis (DEA). A step-by-step analysis is conducted using the $\prime \prime maEndToEnd^{\prime \prime }$ data library, which is a Bioconductor R package (R code is provided in **Supplementary File S6**). The details of the DEA and the DEA results depicted in the volcano plot are given in **Supplementary File S7**.

### 2.3 Sensitivity analysis

Sensitivity analysis (SA) is a mathematical approach designed to quantify how changes in model outputs can be attributed to model inputs, such as initial concentrations and parameters. These approaches allow researchers to assess the level of confidence in results obtained from the mathematical model. It can be conducted to identify the critical reactions that control the systems during infection (66).

In this study, a global sensitivity analysis (GSA) of the model was conducted to assess species-to-species and species-to-kinetic parameters relationships using the variance-based Sobol method. This method uses Sobol indices to quantify the variance contribution of individual model inputs.

The first-order Sobol index (main effect index) measures the variance in the output attributable to a single input, while the second-order Sobol index captures interactions between two inputs. The total-order Sobol index represents the combined variance contribution of all interactions involving a given input (67). Specifically, the first-order index indicates the proportion of total variance due to an individual input alone, whereas the total-order index represents the variance contribution from both the input itself and its interactions with other inputs.

For each input entity, a total-order Sobol index greater than the first-order index suggests that interactions among parameters significantly contribute to output variance. Conversely, if the total-order index is equal to or only slightly greater than the first-order index, it indicates that individual parameters primarily drive the variance, with minimal impact from parameter interactions.

The mathematical representations of first- and total-order Sobol indices are provided below as follows:

The direct variance-based measure of sensitivity *S_i_*, called the first-order sensitivity index. Assume X is a vector of n uncertain model inputs $\left\{X_{1},X_{2},\ldots X_{n}\right\}$ , and Y is a chosen univariate model output. *S_i_* is stated as:


$$S_{i}=1-\frac{E_{X_{i}}(Var_{X_{∼}i}(Y|X_{i}))}{Var\left(Y\right)}$$

where $E_{X_{i}}(Var_{X_{∼}i}(Y|X_{i}))$ is a Monte Carlo estimator. $X_{∼}i$ notation indicates the set of all inputs except $X_{i}$ . $S_{i}$ is the contribution to the output variance of the main effect of $X_{i}$ ; therefore, it measures the effect of varying $X_{i}$ alone, but averaged over variations in other input parameters (68).

Using the $S_{i}$ , Sij , and higher-order indices, we can build a picture of the importance of each input parameter as a variable in determining the output variance. However, when the number of variables is large, this requires the evaluation of $2^{n}-1$ indices, which can be too computationally demanding. For this reason, a measure known as the total-effect index or total-order index, $S_{Ti}$ , is used (69). This measures the contribution to the output variance of $X_{i}$ , including all variance caused by its interactions, of any order, with any other input variables. It is given as,


$$\begin{array}{c} S_{Ti}=\sum_{i=1}^{n}S_{i}+\sum_{i<j}^{n}S_{ij}+\ldots S_{1,2,\ldots ,n}=1=\frac{E_{X_{∼}i}(Var_{X_{i}}(Y|X_{∼}i))}{Var\left(Y\right)}\\ =1-\frac{Var_{X_{∼}i}(E_{X_{i}}(Y|X_{∼}i))}{Var\left(Y\right)} \end{array}$$

The workflow of GSA in the Sobol method is described as follows: define the domain of interest in parameter space, then sample it; this step is followed to simulate the model and calculate sensitivity measure. Using uniform distribution for the selected parameters generated a series of independent random numbers, the lower and upper distribution is generated according to the current input values provided. Sobal as Sobol sequence sampling is used. Refer to the range of parameters available in **Supplementary File S8**.

### 2.4 Regulation dynamic of the hemostatic and complement systems

**Figure 2:**
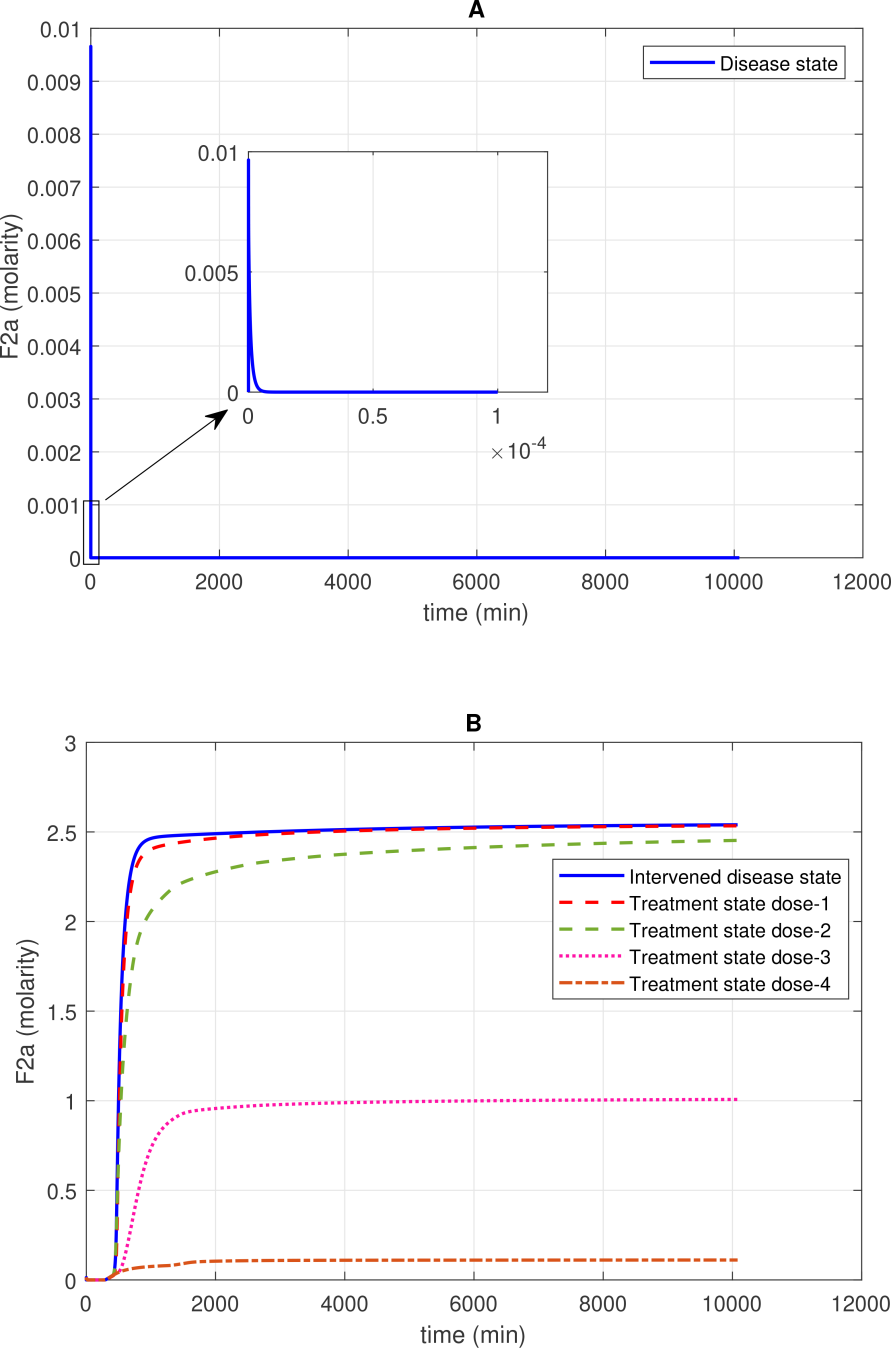
Concentration–time profiles for thrombin (F2a) in the disease, intervened disease, and treatment states. **(A)** In the disease state, the concentration of F2a initially increases and then gradually decreases to a minimal level. **(B)** In the intervened disease state, a decrease in the levels of FH, C1INH, TFPI, and A2M leads to an increase in F2a and an altered trend that stabilizes at a peak level. Furthermore, in the treatment state, with different doses of heparin, the level of F2a is controlled and reduced to a minimal level. Activated coagulation factor 10 (thrombokinase; F10a) and the complement enzymes MASP-1 and MASP-2, through their binding to prothrombin (F2), play a significant role in the induction of F2a. In contrast, complement system inhibitors FH and C1INH, along with coagulation cascade inhibitors AT3 and A2M, can suppress F2a levels. Heparin, despite its efficacy in anticoagulation, poses bleeding risks that require cautious dose management and monitoring.

**Figure 3:**
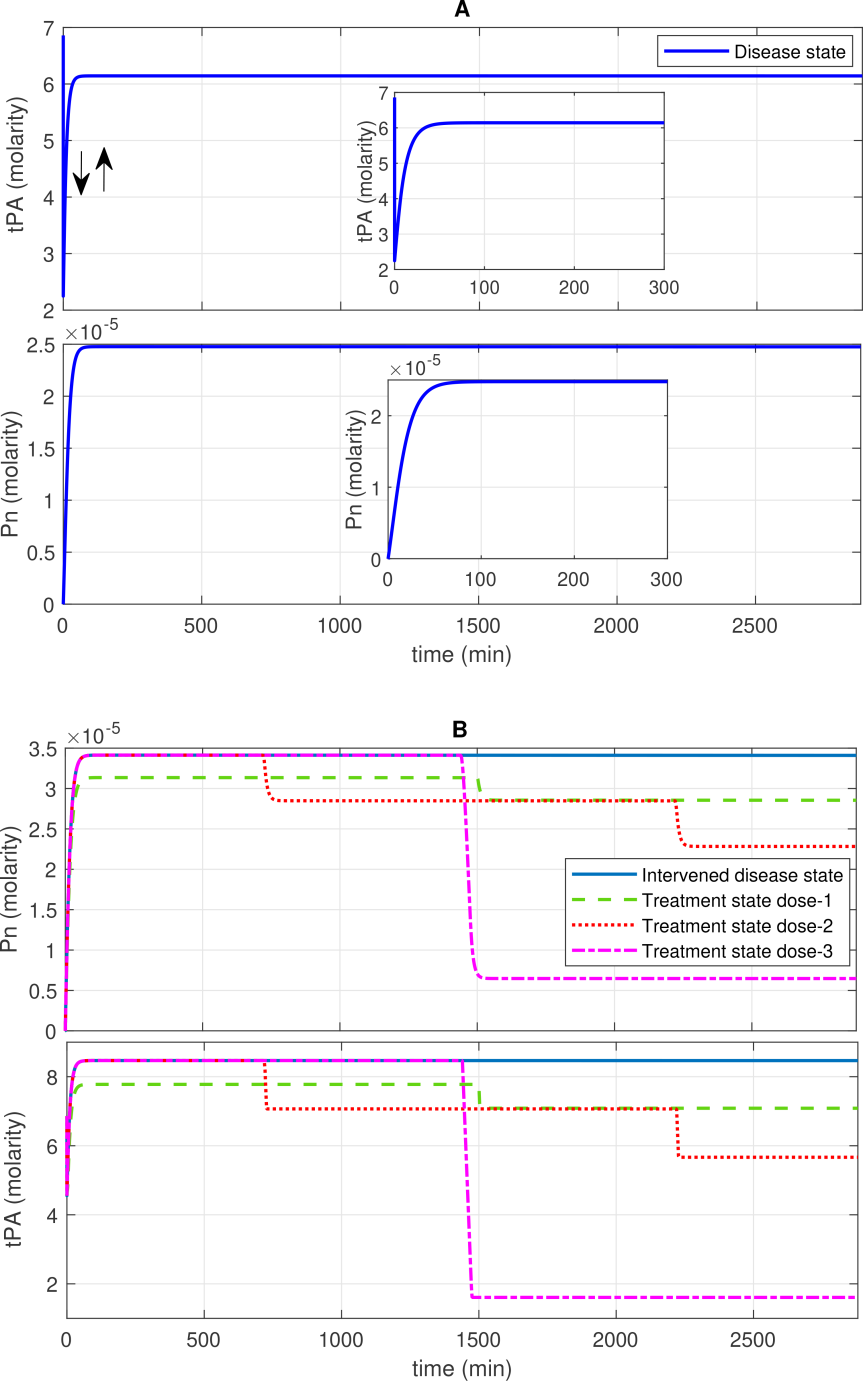
Concentration–time profiles for plasmin (Pn) and tissue plasminogen activator (tPA) in the disease, intervened disease, and treatment states. **(A)** The concentration dynamic plots show that tPA initially decreases, then increases to a stable level, while Pn increases to a stable high level in the disease state. **(B)** The perturbation (decrease) in the levels of FH, C1INH, TFPI, and A2M in the intervened disease state causes an increase in the levels of Pn and tPA. Doses of tranexamic acid treatment effectively manage the levels of these entities. In the infection, due to the high response of the BK-B2R pathway, the levels of tPA increase, while high levels of PAI-1 tend to regulate it. Elevated levels of KAL and tPA, which are associated with Glu-Pg, significantly increase Pn levels, while a deficiency in C1INH is unable to mitigate its levels.

**Figure 4:**
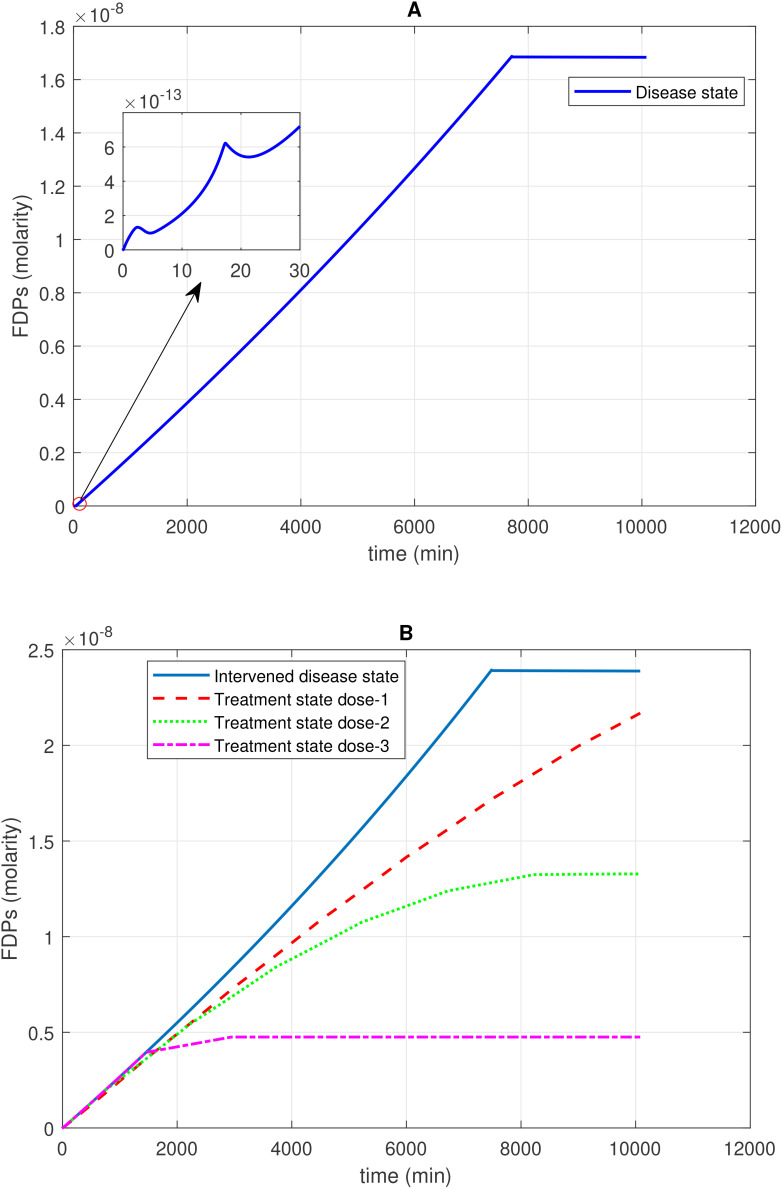
Time profile of fibrin degradation products (FDPs) generation in the disease, intervened disease, and treatment states. **(A)** In the disease state, the generation–time profile graph for FDPs shows a steady increase, followed by a plateau at its peak level. On the side of the main graph, there are irregular generation patterns of FDPs for a short time. **(B)** In the intervened disease state, a decrease in the levels of FH, C1INH, TFPI, and A2M results in a very minor increase in the generation of FDPs compared to the disease state, but they follow a similar pattern. During treatment, tranexamic acid, which targets tPA, is used to control FDPs. Higher doses of the drug significantly reduce the generation of FDPs. The enzyme plasmin plays a key role in the generation of FDPs, as plasmin breaks down fibrin to generate FDPs Tranexamic acid, while effective in controlling excessive bleeding, may increase the likelihood of thrombotic events in certain patient populations.

**Figure 5:**
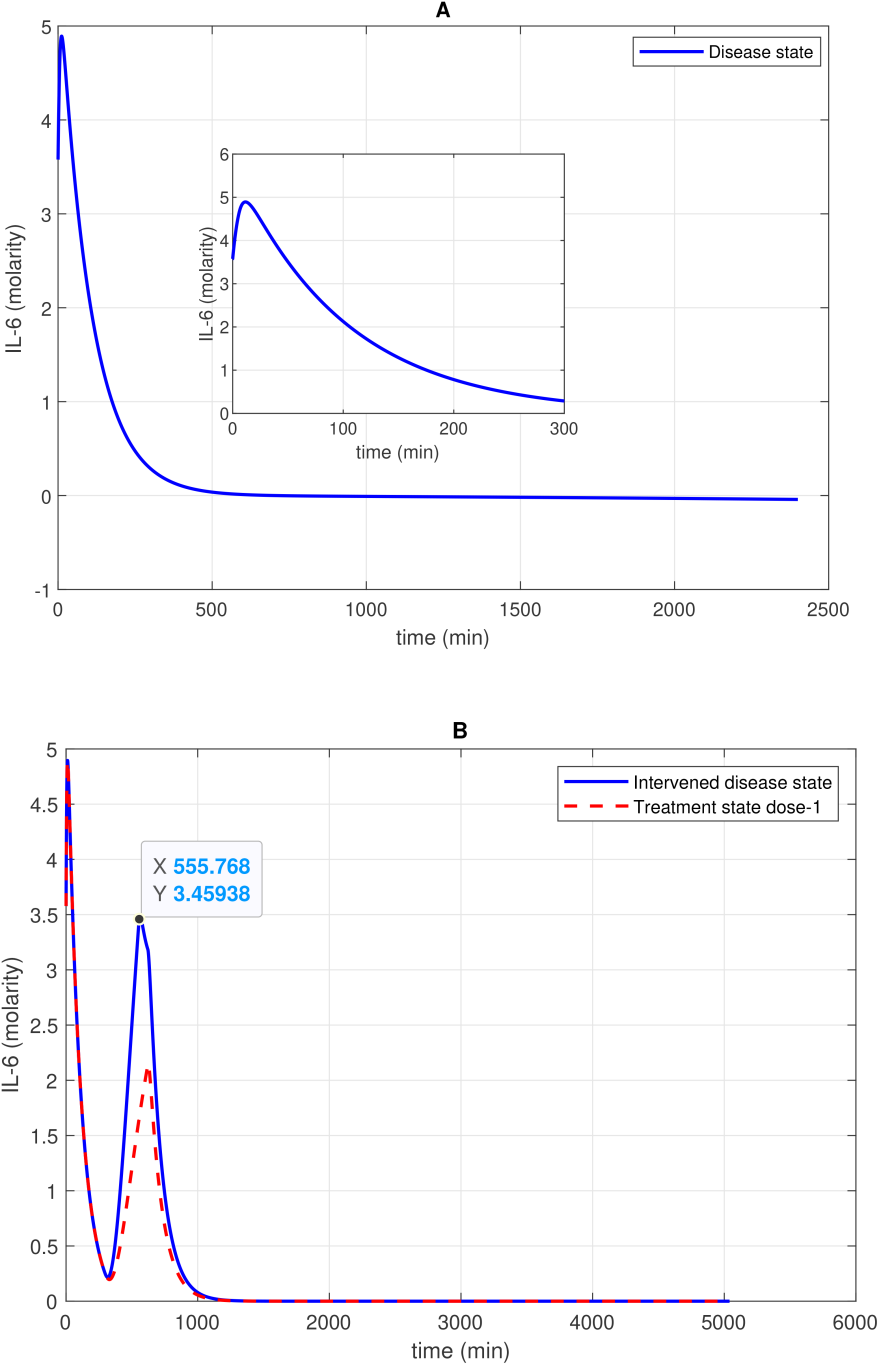
Concentration–time profile of 1L-6 levels in the disease, intervened disease, and treatment states. **(A)** In the disease state, the concentration of 1L-6 increases rapidly in the initial stage and then decreases exponentially to a minimum level. **(B)** In the intervened disease state, a decrease in the levels of FH, C1INH, TFPI, and A2M impacts IL-6 levels, showing an irregular pattern. Treatment with avdoralimab, which targets C5aR1, has a restorative effect on IL-6 levels. The activation of the complement pathways C3a-C3aR1 and C5a-C5aR1, along with the kallikrein-kinin BK-B2R pathway, leads to increased IL-6 levels. Avdoralimab, an emerging therapy targeting C5aR1, offers promise in mitigating inflammation but raises concerns about immunological side effects that could impact treatment safety.

**Figure 6:**
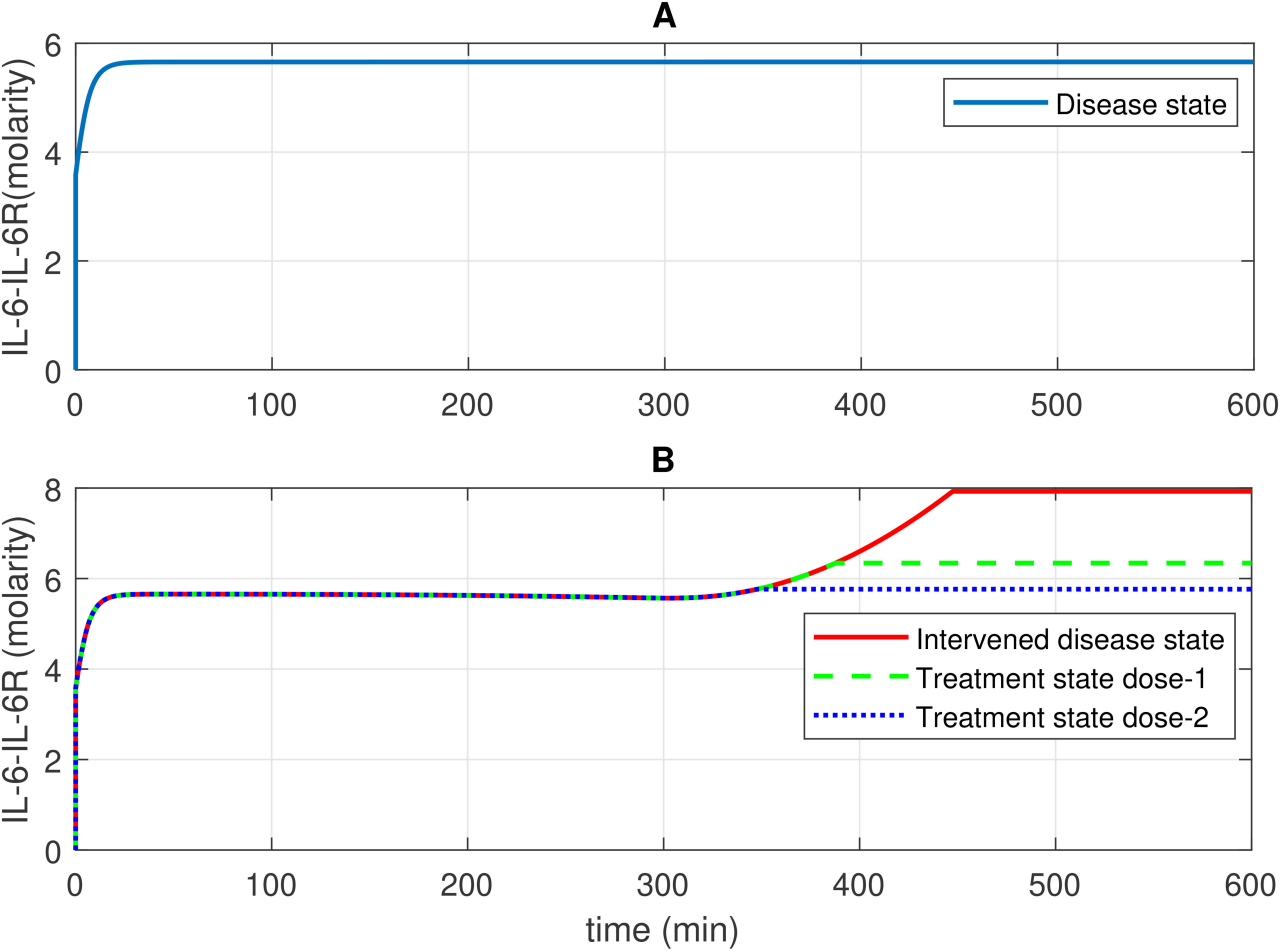
Concentration–time profiles for IL-6 and 1L-6R complex in the disease, intervened disease, and treatment states. **(A)** In the disease state, the concentration of the IL-6-IL-6R complex rises and stabilizes at a certain level. **(B)** In the intervened disease state, a decrease in the levels of FH, C1INH, TFPI, and A2M affects the IL-6-IL-6R complex in the later phase, causing its levels to increase and eventually stabilize. In the treatment state, tocilizumab (TCZ), an anti-IL-6R antibody, restores the IL-6–IL-6R complex levels. The production of IL-6 and IL-2R levels are key factors that increase the levels of the IL-6–IL-6R complex. TCZ can be highly effective in managing inflammation; however, high doses increase the likelihood of more severe side effects, including liver enzyme elevations and increased blood pressure. Careful monitoring is essential to minimize these risks.

**Figure 7:**
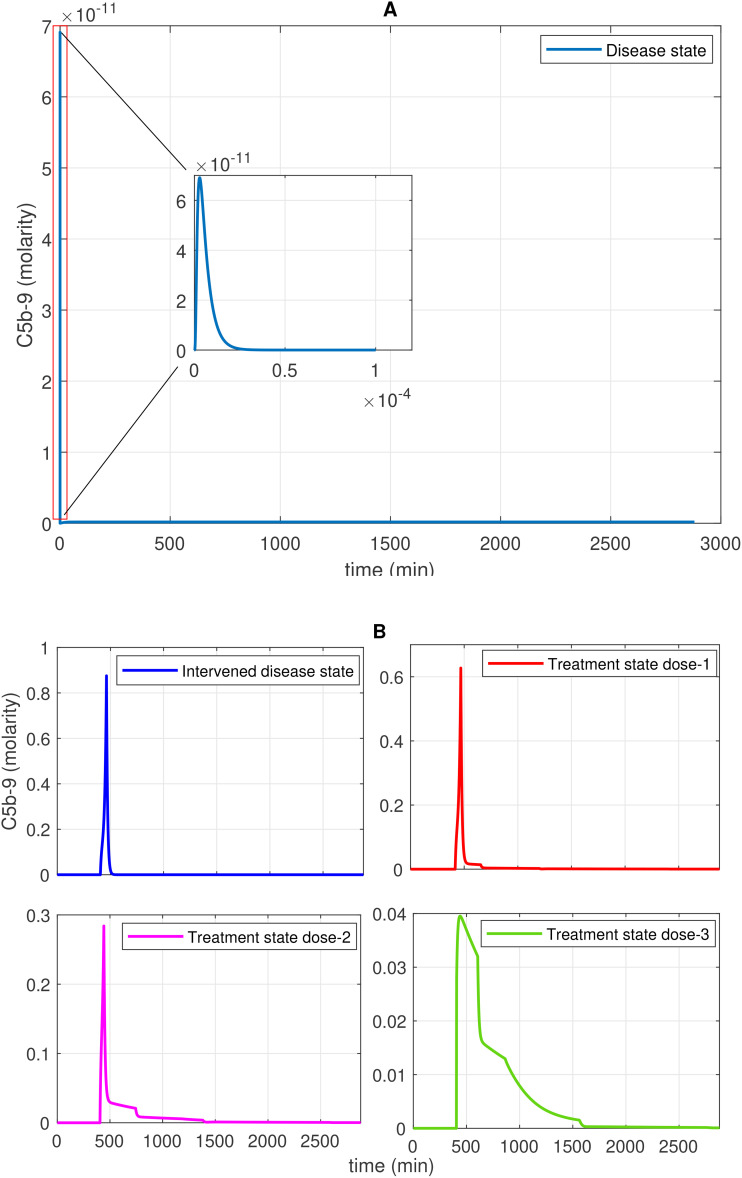
Time profile of membrane attack complex (MAC; C5b-9) generation in the disease, intervened disease, and treatment states. **(A)** In the disease state, C5b-9 generation initially shows a rapid increase, reaches a peak, and then declines to a minimum level. **(B)** In the intervened disease state, a perturbation (decrease) in the levels of FH, C1INH, TFPI, and A2M affects complement proteins, causing C5b-9 levels to increase and rapidly reach their peak, approximately 462 min later. In this state, C5b-5 levels are higher than those in the disease state. During heparin treatment targeting complement proteins (C2–C9), drug doses demonstrate a restorative effect on C5b-9 generation. In COVID-19, the cleaved complement fragment C5b interacts with complement proteins C6–C9, resulting in elevated C5b-9 levels. The regulator CD59 contributes to the suppression of C5b-9. Heparin, despite its efficacy in anticoagulation, poses bleeding risks that require cautious dose management and monitoring.

The study presents the concentration–time profiles of various entities including components thrombin (F2a), plasmin (Pn), tissue plasminogen activator (tPA), fibrin degradation products (FDPs), interleukin-6 (IL-6), the IL-6-IL-6R complex, and the membrane attack complex (C5b-9), respectively (**Figures 2**–**7**). The concentration–time profiles of these entities were evaluated under the disease state, intervened disease state, and treatment state, respectively.

The simulation results for the treatment state showed the restorative effects of the drugs heparin, tranexamic acid (TXA), avdoralimab, garadacimab, and tocilizumab (TCZ) on hemostatic components such as coagulation and fibrinolytic factors, the complement terminal complex, and inflammation components, which can be linked to clinically significant control in treated COVID-19 patients. Several experimental studies have demonstrated a mitigation of the responses of these components as a result of treatment.

We also consider the potential side effects of the drugs evaluated to provide a comprehensive assessment of their clinical applicability. Heparin, while commonly used for anticoagulation, is associated with bleeding risks, particularly in patients requiring high doses or with pre-existing bleeding disorders. Tranexamic acid, a widely used antifibrinolytic agent, carries a risk of thrombotic complications, especially in patients with a predisposition to thromboembolism. For avdoralimab, an anti-C5aR1 antibody, potential immunological side effects such as immune dysregulation and hypersensitivity are noted. Similarly, garadacimab, a factor F12a inhibitor, has been associated with rare hypersensitivity reactions, necessitating close monitoring in clinical use. TCZ, an anti-IL-6R antibody, has severe side effects, including liver enzyme elevations and increased blood pressure. These considerations emphasize the importance of balancing therapeutic benefits with potential risks in the context of personalized treatment strategies.

#### 2.4.1 Concentration–time profile for F2a

**Figure 8:**
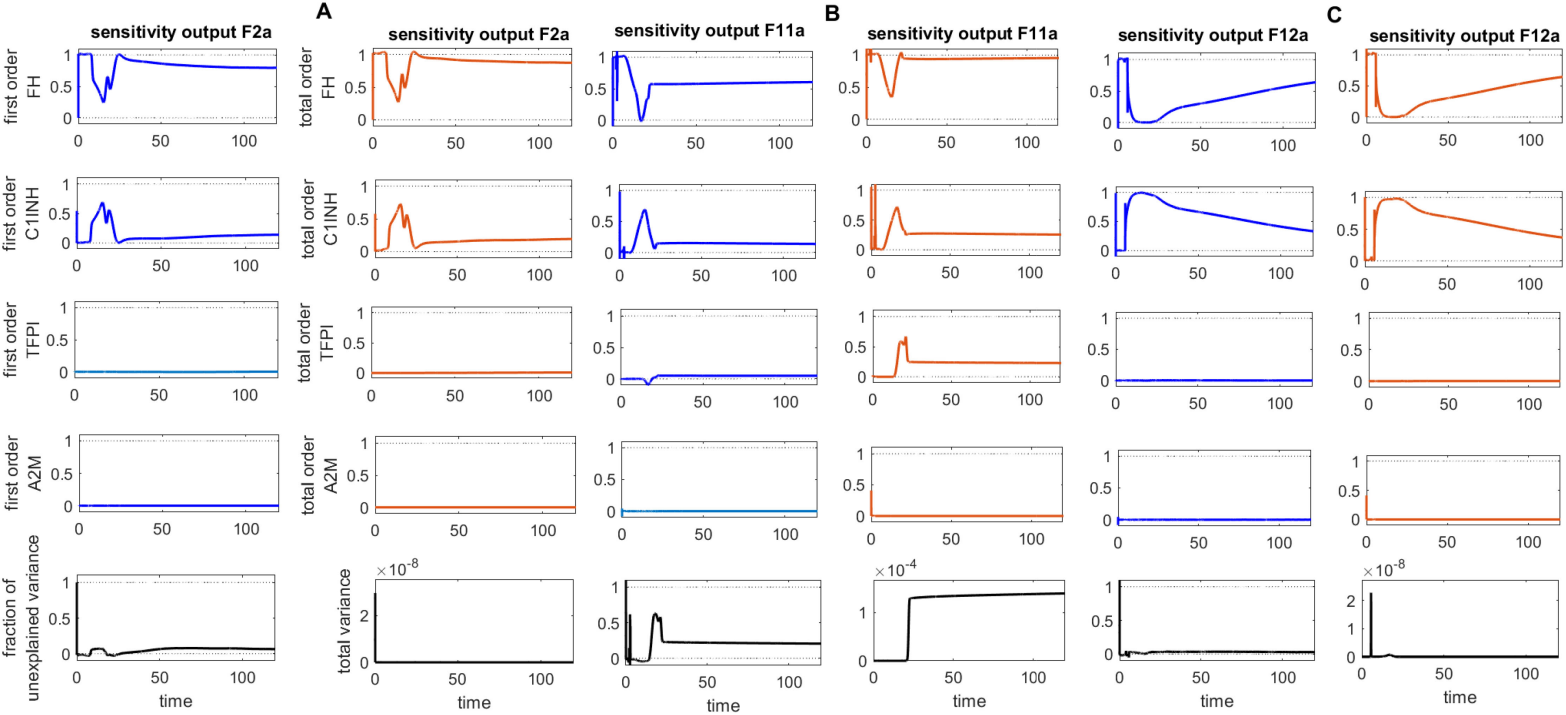
Global sensitivity analysis over time for the effects of regulators FH, C1INH, TFPI, and A2M on the coagulation factors thrombin (F2a), activated thromboplastin antecedent (F11a), and activated Hageman factor (F12a) in the disease state. **(A)** FH is the most sensitive component to F2a, with its Sobol indices increasing around ~ t = 15. C1INH has a moderate effect on F2a, showing a decreasing trend in its Sobol indices around ~ t = 15, while TFPI is only minimally sensitive. **(B)** FH is the most sensitive component to F11a, with its Sobol indices increasing around ~ t = 15. C1INH has a moderate effect on F11a, showing a decreasing trend in its Sobol indices around ~ t = 15, while TFPI shows irregular, small increasing trends in its Sobol indices around ~ t = 19, which moderately affect F11a. **(C)** FH is the most sensitive component to F12a, with its Sobol indices increasing around ~ t = 15. C1INH has a moderate effect on F12a, showing a decreasing trend in its Sobol indices around ~ t = 15. The components FH, C1INH, and TFPI significantly impact the responses of F2a, F11a, and F12a, respectively, as indicated by total-order Sobol index being greater than first-order Sobol index. Additionally, the total variance plot showing low variance for the F2a response and the fraction of unexplained variance plots showing little variation imply that the unexplained variance may be significant. In contrast, the total variance plots showing increased variances for the responses of F11a and F12a, along with the fraction of unexplained variance plots showing little variation, suggest that the unexplained variance may be insignificant. The unexplained variance is found by subtracting the sum of all first-order Sobol indices from 1. The total variance is calculated using var(response), where response refers to the model output at each time point. Overall, this analysis indicates that FH and C1INH may be the most important parameters to investigate further. Total-order Sobol index (maroon) greater than the first-order index (blue) suggests that interactions among entities significantly contribute to output variance.

The concentration dynamics of the coagulation cascade factor thrombin (F2a) are examined under the disease, intervened disease, and treatment states. In the disease state, the levels of F2a rapidly increased, reaching a peak amplitude of ~0.0096 M after 4.9 × 10^–9^ min and then sharply decreased to 0 M (**Figure 2A**). The downregulation of F2a is primarily attributed to FH and C1INH. FH significantly affects F2a, and C1INH has a mild impact on F2a levels (see GSA in **Figure 8A**). Additionally, for the upregulation of F2a, the complement components MASP1 and MASP2 play a key role.

In the intervened disease state, the response of F2a is altered due to perturbations in the levels of TFPI, C1INH, FH, and A2M. The levels of F2a gradually increased to ~2.5 M after ~2009 min and remained consistently high during the stationary phase. Notably, in perturbation, where only 50% of FH and ~ 3.3% of C1INH, along with other factors, remained, they are unable to reduce F2a. These results indicate that FH and C1INH are potent inhibitors that play a significant role in regulating the response of F2a in the disease state compared to the response in perturbation (**Figure 2B**).

In treatment state with heparin treatment, heparin is found to potentiate the action of target antithrombin III (AT3). Heparin is known to be an effective drug for severe COVID-19 patients (70). Heparin interacts with AT3 at an association rate of 13.3 × 10^7^ M^–1^ *s* ^–1^ (71). Under different dosing scenarios, the expression level of F2a has been evaluated. High restoration trends are observed due to the higher heparin doses. Dose 1, at 0.561 mol/L (fivefold lower than the concentration of AT3), has a minor effect on F2a levels, while dose 2, at 2.8034 mol/L (one-to-one ratio with AT3), has a better therapeutic effect on F2a levels. Moreover, dose 3, at 14.017 mol/L (fivefold higher than the concentration of AT3), and dose 4, at 19.62 mol/L (10-fold higher than the concentration of AT3), result in a high recovery of F2a levels (**Figure 2B**).

The heparin dose in mol/L is a computational estimate designed to simulate its potential effects on biological pathways associated with SARS-CoV-2 infection. In the model, heparin binds to AT3 as a potentiator (72) and to complement proteins C2–C9 as a suppressor (73). These interactions help control the formation of the C5b-9 complex, which is involved in stimulating prothrombin (see the interaction labeled as $\prime \prime 7{\text{Ψ}}^{\prime \prime }$ in **Figure 1**).

Although heparin treatment carries a risk of bleeding, low molecular weight heparin (LMWH) has been widely used during COVID-19 (72, 74). During infection, a reduction in AT3 disrupts anticoagulation, leading to increased clotting and thrombosis. LMWH treatment has proven effective in addressing these issues (75, 76). These findings suggest that heparin treatment can improve AT3 levels, contributing to the effective management of thrombosis.

#### 2.4.2 Concentration–time profiles for Pn and tPA

**Figure 9:**
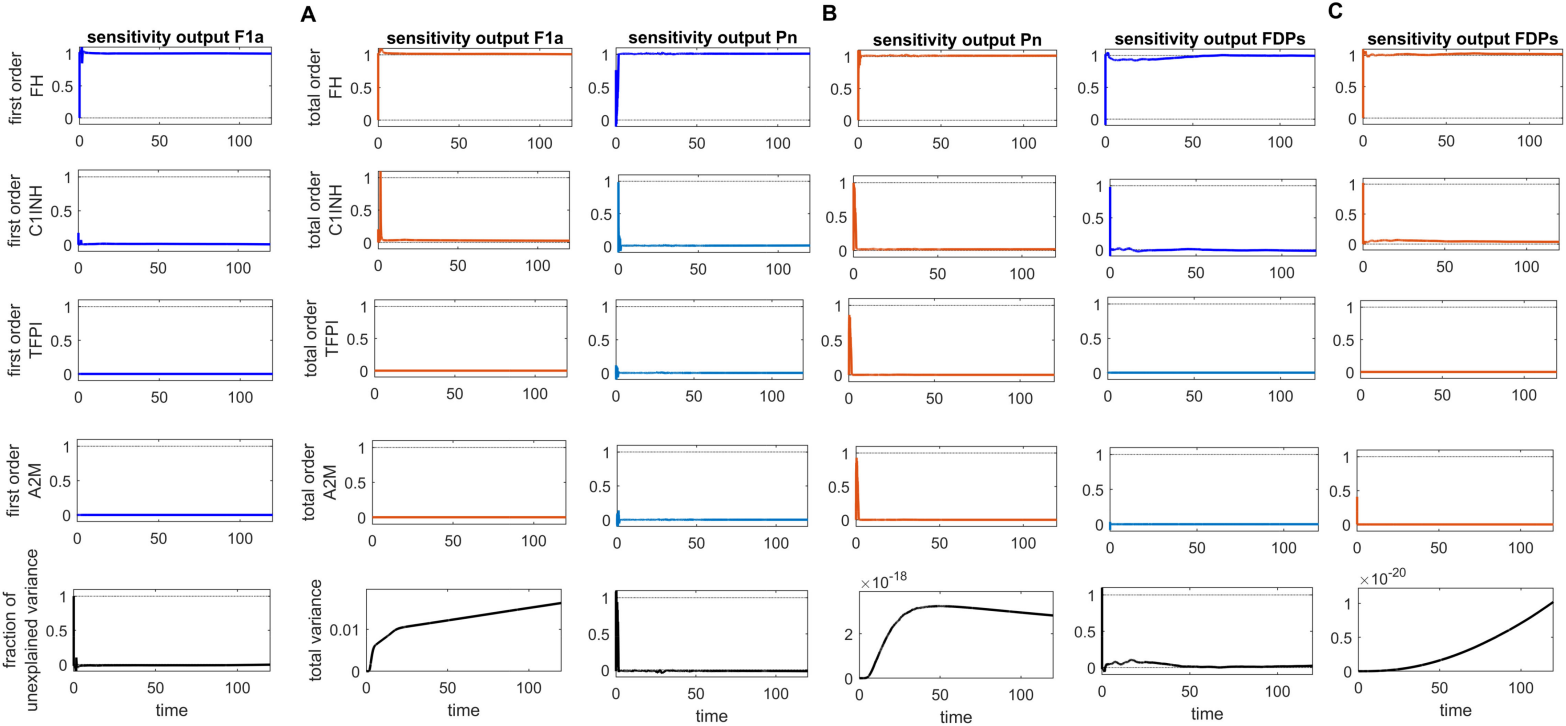
Global sensitivity analysis over time for the effects of regulators FH, C1INH, TFPI, and A2M on the fibrinolytic factors fibrin (F1a), plasmin (Pn), and fibrin degradation products (FDPs) in the disease state. **(A)** FH is the most sensitive for F1a, with its Sobol indices increasing significantly, although showing slight irregularities around ~ t = 2. C1INH shows a decrease in Sobol indices after ~ t = 2 and has a moderate effect. In contrast, TFPI and A2M have a minor effect on F1a, with very low Sobol indices. **(B)** FH is the most sensitive component for Pn, with high Sobol indices that increase around ~ t = 2. C1INH, TFPI, and A2M have a moderately significant effect on Pn, showing a decreasing trend in Sobol indices around ~ t = 2. **(C)** FH is the most sensitive component for FDPs, with its Sobol indices increasing around ~ t = 2. C1INH has a moderate effect on FDPs, showing a decreasing trend in Sobol indices around ~ t = 2. In contrast, TFPI and A2M have a minor impact on FDPs, with very low Sobol indices. The components FH, C1INH, and TFPI significantly affect the responses of F1a, Pn, and FDPs, respectively, as indicated by the total-order Sobol index being greater than first-order Sobol index. Additionally, the total variance plots showing increased variances for the responses of fibrinolytic factors suggest that the input components may be more important to investigate further. The fraction of unexplained variance plots shows little variation, but the total variance increasing trends indicate that the unexplained variance may be insignificant. The unexplained variance is found by subtracting the sum of all first-order Sobol indices from 1. The total variance is calculated using var(response), where response refers to the model output at each time point. Total-order Sobol index (maroon) greater than the first-order index (blue) suggests that interactions among entities significantly contribute to output variance.

In the disease state, increasing trends of Pn response are found after ~120 min, reaching ~2.5 × 10^–5^ M, and subsequently increased and remained high (**Figure 3A**). In the upregulation of Pn, factors such as KAL, tPA, and Glu-Pg play a significant role. In contrast, the downregulation of Pn is significantly influenced by FH, while C1INH, TFPI, and A2M exhibit moderate sensitivity (see GSA, **Figure 9B**).

In the propagation phase of fibrinolysis, F1a generates tPA in the presence of Lys-Pg (43). In the model, a product of F1a and Lys-Pg increases tPA production. Moreover, a complex of BK and B2R formation also enhances tPA levels. Initially, tPA levels decreased from ~6.8 M to approximately 2.2 M. Subsequently, there is an increasing trend in tPA levels, leading to a high level of ~6.14 M after ~507 min.

The decrease in inhibitor levels in the intervened disease state leads to an increasing trend in Pn and tPA levels. In perturbation, compared to the disease state, Pn levels increased slightly to 3.4 × 10^–5^ M after ~75 min, while tPA levels increased to ~8.46 M after ~60 min (**Figure 3B**).

In the treatment state with tranexamic acid (TXA) treatment, dose-dependent regulatory responses are observed for Pn and tPA. A low dose, like dose 1 at 0.69 mol/L (10-fold lower than the concentration of tPA), has minor regulatory effects. Dose 2 at 1.40 mol/L (fivefold lower than the concentration of tPA) exhibits a better regulatory effect. A high dose, such as dose 3 at 6.86 mol/L (equivalent to tPA concentration), results in significant recovery effects after 1,500 min (**Figure 3B**).

The TXA modulates coagulopathy through the suppression of fibrinolysis and, hence, could prevent disseminated intravascular coagulopathy (DIC) during SARS-CoV-2 infection (77). These results imply that inhibiting tPA with TXA, a potential therapeutic drug, can effectively decrease Pn levels in COVID-19. In fibrinolysis, regulating tPA to lower Pn levels may help prevent hyperfibrinolysis.

#### 2.4.3 Concentration–time profiles for FDPs

The enzymatic interaction of Pn with F1a, as indicated by Michaelis–Menten kinetics, plays a pivotal role in increasing the levels of FDPs. High production of FDPs (1.7 × 10^–8^ M) is observed after 10,080 min in the disease state. In the intervened disease state, the disorder in inhibitors leads to an increase in FDPs, rising from 0 M to 2.4 × 10^–8^ M after 10,080 minutes (**Figure 4A**).

The uncertainty in the generation of FDPs is caused by the levels of FH, C1INH, TFPI, and A2M. The GSA (**Figure 9C**) suggests that FH has a significant impact on FDPs. In contrast, C1INH has a moderate effect, while A2M and TFPI have only a minor impact on FDPs. FH and C1INH play a significant role in the regulation of activated coagulation and complement cascades. Notably, the depletion of FH and C1INH could result in the progression of SARS-CoV-2-associated coagulopathy, leading to thromboinflammation (4), which contributes to elevated D-dimer levels (78).

In the treatment state with TXA treatment, FDPs are reduced. The reduction in FDPs depends on the amount of TXA administered. When considering tPA as the target and TXA as an inhibitor of tPA, it becomes evident that different doses of TXA have varying effects on FDPs levels. A low dose in dose 1, at 0.69 mol/L (10-fold lower than the concentration of tPA), results in lower FDPs level. Dose 2, at 1.40 mol/L (fivefold lower than the concentration of tPA), exhibits a restorative effect on FDP generation. Dose 3, at 6.86 mol/L (equivalent to tPA concentration), results in a better recovery of FDPs (**Figure 4B**).

In critical COVID-19 patients, hypercoagulation and hyperfibrinolysis responses with elevated levels of FDPs have been reported (79). TXA therapy can help mitigate FDP overproduction during COVID-19.

#### 2.4.4 Concentration–time profile for IL-6 and the IL-6-IL-6R complex

**Figure 10:**
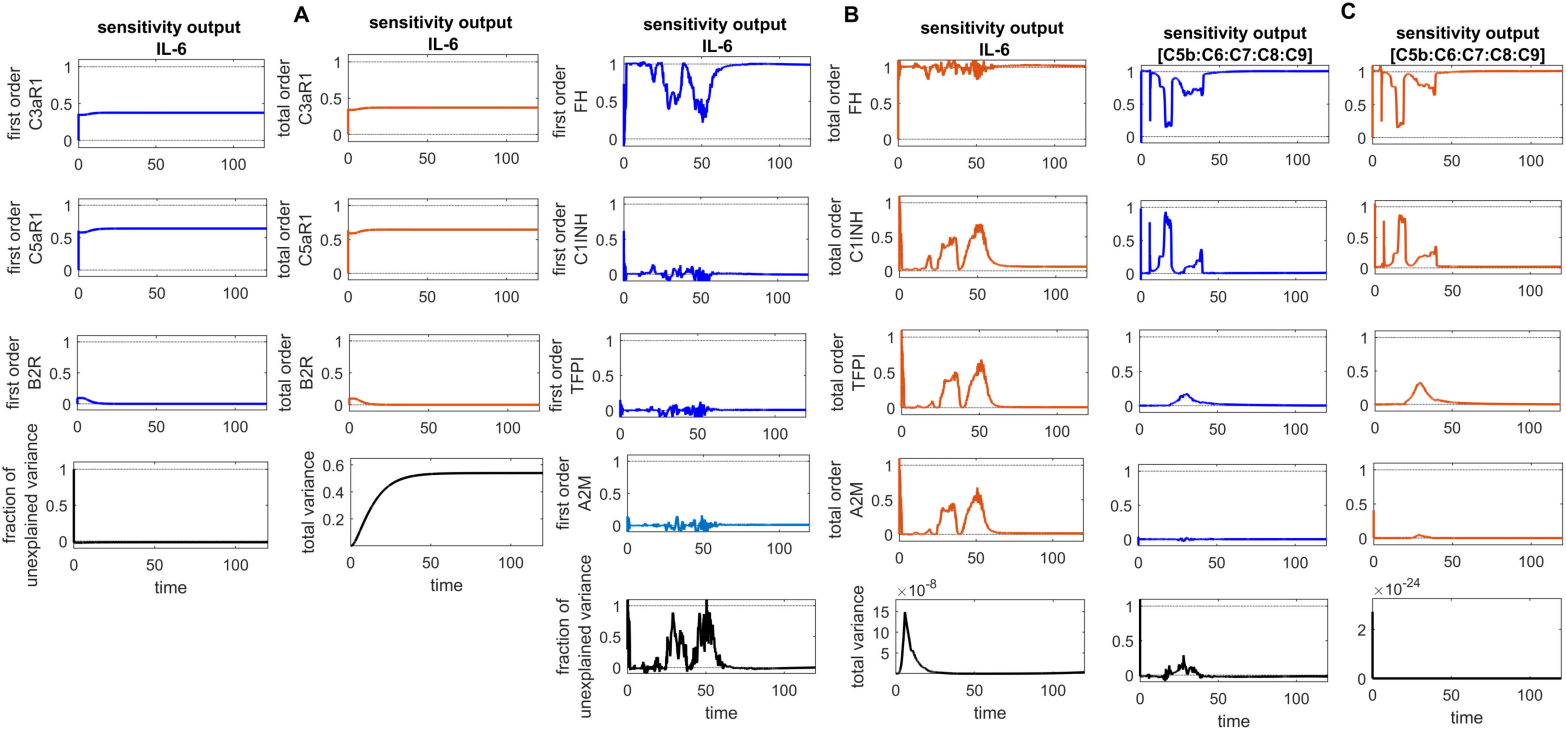
Global sensitivity analysis over time in the disease state. **(A)** Sobol indices plots show an increasing trend for C5aR1 and C3aR1 around ~ t = 50, while they decrease for B2R. C5aR1 and C3aR1 have a significant impact on IL-6, whereas B2R has a minor impact on IL-6 levels. **(B)** The irregular and sharply increasing pattern of Sobol indices for FH indicates that FH is highly sensitive and significantly affects IL-6 levels. In contrast, the mildly increasing patterns of Sobol indices for C1INH, TFPI, and A2M around ~ t = 50 suggest a moderate influence on IL-6. **(C)** The analysis for the complement terminal complex (C5b:C6:C7:C8:C9) shows that FH is highly sensitive, C1-INH is moderately sensitive, and TFPI and A2M are less sensitive. High, irregular Sobol indices are observed for FH and C1-INH around ~ t = 50. Additionally, the total variance plot showing major variance for IL-6 suggests that input components may be more important to investigate further. The fraction of unexplained variance is very low but non-zero and shows some variation around ~ t = 50, implying that the unexplained variance could be insignificant. For FH, C1INH, TFPI, and A2M, the fraction of unexplained variance shows disturbed variances, but the total variance plot shows moderate variance initially for the IL-6, implying that the unexplained variance could be significant. The total variance plot shows a very low variance for C5b-9. Unexplained variance is found by subtracting the sum of all first-order Sobol indices from 1. The total variance is calculated using var(response), where response refers to the model output at each time point. Total-order Sobol index (maroon) greater than the first-order index (blue) suggests that interactions among entities significantly contribute to output variance.

In the context of the disease state, IL-6 levels rapidly increase to approximately 4.9 M within approximately 14 min from an initial expression level of approximately 3.58 M and subsequently reduce to 0 M (refer to **Figure 5A**). In the upregulation phase, entities like C5aR1, C3aR1, and B2R play pivotal roles. C5aR1 and C3aR1 significantly influences IL-6 levels, and B2R has a minor effect on IL-6 levels (see GSA in **Figure 10A**). Furthermore, in the downregulation of the IL-6 response, inhibitors FH and C1INH are found to be significant. **Figure 10B** shows that FH has a high effect on IL-6 levels, while C1INH, TFPI, and A2M have a moderate impact on IL-6 levels. IL-6 binds to the IL-6 receptor (IL-6R) forming the IL-6–IL-6R complex. In the disease state, the concentration–time profile (**Figure 6A**) shows that the level of this complex increases and stabilizes at approximately 5.6 M.

In the inflammatory response through the C5a and C5aR1 interaction, the formation of C5a-C5aR1 complex plays a crucial role in inducing IL-6 levels. The levels of IL-6 are positively correlated with the expression of C5aR1, C3aR1, and B2R (80, 81). Furthermore, F1 is directly correlated with IL-6. In COVID-19, due to excessive IL-6 levels, there is a high production of F1 (59). Interestingly, it was reported in Woodruff and Shukla (82) that C5aR1 is a potential target with avdoralimab treatment for managing inflammation during COVID-19.

In the context of the intervened disease state, perturbations in FH, C1INH, TAFI, and A2M levels altered the trends of the IL-6 response. It exhibited a high expression level, increasing from 3.58 M to 4.9 M before declining to approximately 0.21 M within approximately 314 min. A subsequent increase to approximately 3.5 M after 556 min, ultimately decreased to nearly 0 M. Targeting C5aR1 in the treatment state with avdoralimab treatment led to the suppression of IL-6 levels. In avdoralimab treatment, a dosing amount of 10.1662 mol/L (one-to-one with the levels of target C5aR1) resulted in a restorative effect (**Figure 5B**). In the perturbation state, due to lower levels of FH, C1INH, TFPI, and A2M, the IL-6-IL-6R response is higher in the late phase (after 300 min) and increases to a stable level of approximately 8.0 M (**Figure 6B**). Tocilizumab (TCZ), an anti-IL-6 receptor antibody treatment at two doses—dose 1 (1.5855 mol/L, which is five times lower than the concentration of the target IL-6R) and dose 2 (7.9273 mol/L, equal to the levels of the target IL-6R)—reduced the levels of the IL-6–IL-6R complex (**Figure 6B**).

The role of IL-6 in the pathophysiology of the cytokine storm associated with SARS-CoV-2 infection is well-recognized, and therapies targeting this pathway, such as TCZ have been widely implemented in clinical practice. Tocilizumab reduces IL-6R signaling, thereby mitigating the downstream inflammatory cascade that contributes to the severe progression of (83). However, studies have raised concerns about an increased risk of thrombosis associated with the use of tocilizumab in critically ill COVID-19 patients (84).

These findings imply that C3aR1 could serve as an early-stage target, while C5aR1 may have a more significant impact at later stages. These results are consistent with experimental findings that suggest that the severity of SARS-CoV-2 infection is associated with hyperinflammation resulting from an overresponse to C5a and C5aR1 (49).

#### 2.4.5 Concentration–time profiles for C5b-9

**Figure 11:**
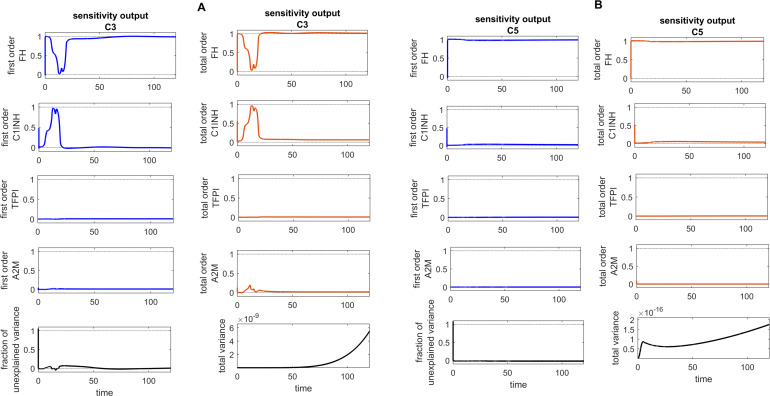
Global sensitivity analysis over time for the effects of regulators FH, C1INH, TFPI, and A2M on the complement proteins C3 and C5 in the disease state. **(A)** FH is the most sensitive component to C3, with its Sobol indices increasing around ~ t = 13. C1INH has a moderate effect on C3, showing a decreasing trend in its Sobol indices around ~ t = 13, while TFPI and A2M exhibit minimal sensitivity and have little impact on C3. **(B)** FH is the most sensitive componentto C5, with its Sobol indices increasing significantly after ~ t = 13. C1INH has a moderate effect on C5, showing very low Sobol indices after ~ t = 13, while TFPI and A2M have minimal impact on C5, with very low Sobol indices. Additionally, the total variance plots showing major variances for both C3 and C5 responses suggest that FH and C1INH may be more important to investigate further. The fraction of unexplained variance shows some variation around ~ t = 13, but the total variance plot shows little change at that time, indicating that the unexplained variance may be insignificant. The unexplained variance is found by subtracting the sum of all first-order Sobol indices from 1. The total variance is calculated using var(response), where response refers to the model output at each time point. Total-order Sobol index (maroon) greater than the first-order index (blue) suggests that interactions among entities significantly contribute to output variance.

In the disease state modeling, the levels of C5b-9 are increased to reach a peak amplitude response of 6.9 × 10^–11^ M after 3.1 × 10^–6^ min and then decrease to 0 M. The inhibition of C5b-9 by CD59, consumption of C5b-9 levels in the generation of F1, and disassociation of the C5b-8 complex from C9 play significant roles in the decrease in C5b-9 level (**Figure 7A**). Notably, the GSA reveals that FH has a significant impact on C5b-9 levels, followed by C1INH, which moderately affects C5b-9 levels. In contrast, TFPI and A2M are less sensitive and have minor effects on C5b-9 levels (**Figure 10C**). This implies that the regulation of complement pathways by FH and C1INH has an indirect effect on the downregulation of C5b-9. Furthermore, the significant impact of FH and C1INH on C3 and C5 is shown in **Figures 11A, B**. The cleaved fragments of C3 and C5, such as C3b and C5b, have a necessary role in C5b-9 complex formation. C5b can combine with the complements C6 to C9 to form C5b-9.

In the intervened disease state modeling, TFPI decreased 60-fold, C1INH 30-fold, FH 2-fold, and A2M 2-fold. The remaining concentrations, TFPI at ~1.7%, C1INH at ~3.3%, FH at 50.0%, and A2M at 50.0% are found insufficient to reduce C5b-9 response. It is observed that C5b-9 levels under perturbation slowly increased, with a delay in reaching ~1.0 × 10^–4^ M within approximately 405 min from a starting concentration of 0 M. After ~462 min, C5b-9 levels suddenly spiked to an amplitude of ~0.87 M. The decreasing trend is started after ~462 min (**Figure 7B**).

Interestingly, considering C2–C9 as targets, the association and disassociation rates of heparin with the complements are provided in **Supplementary Table S1**. In the treatment state with heparin treatment, the levels of C5b-9 are significantly reduced (**Figure 7B**). In heparin treatment, consider dose 1 with a dosage of 2.325 mol/L (twofold lower than the average concentration values of targets), dose 2 with a dosage of 4.650 mol/L (matching the average concentration values of targets), and dose 3 with a dosage of 9.300 mol/L (twofold higher than the average concentration values of targets). Increasing the dosage led to a significant recovery of C5b-9. These results suggest that the components of the CS could serve as prognostic markers and therapeutic targets in COVID-19.

### 2.5 Sensitivity analysis

The global sensitivity analysis (GSA) of the model was performed using the Sobol method. In this method, both first- and total-order indices were computed. First-order index represents the proportion of total variance attributable to an individual input alone, while total-order index captures the variance contribution from the input itself and its interactions with other inputs.

**Figure 12:**
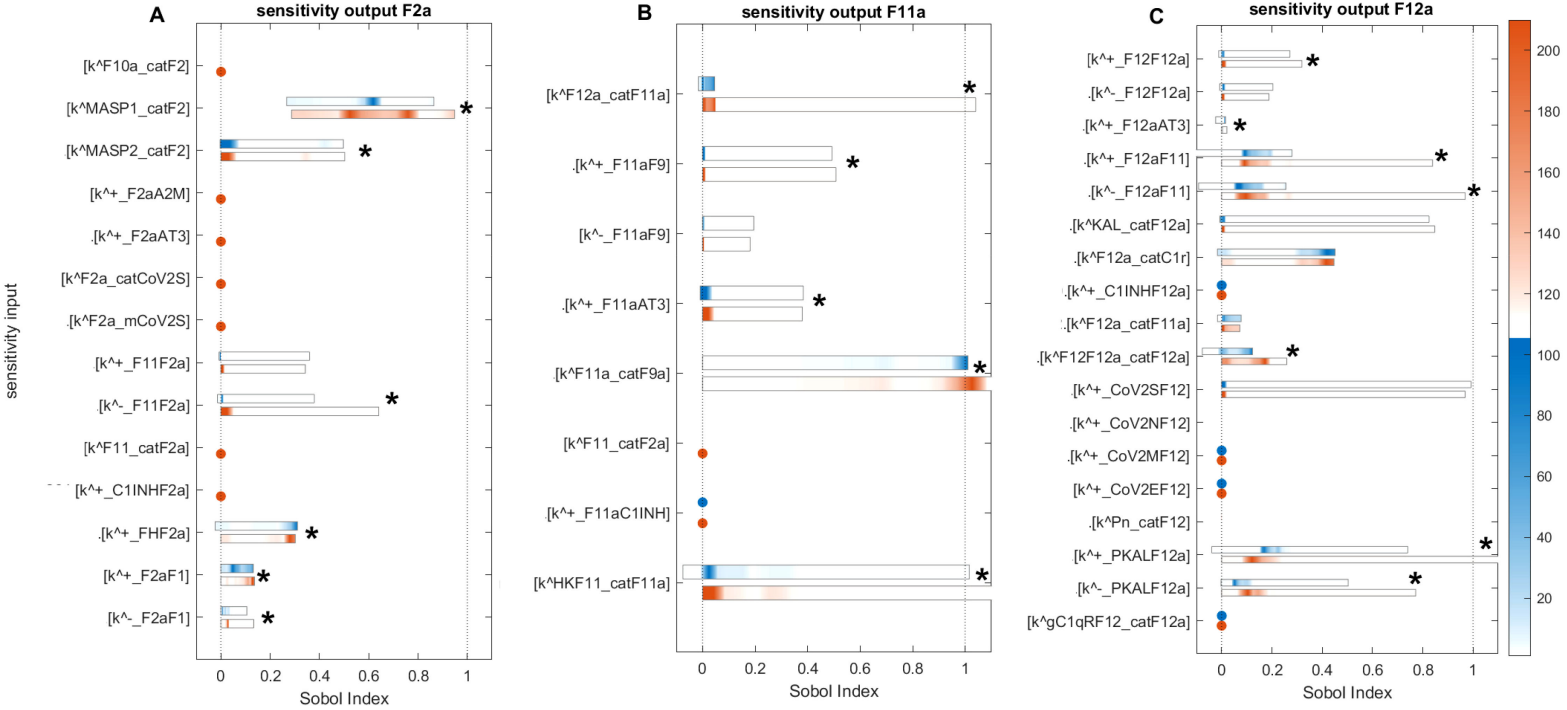
Global sensitivity analysis of the key parameters influencing F2a, F11a, and F12a in the disease state. **(A)** Sensitive parameters to the F2a: the most sensitive parameters is $k_{catF2}^{MASP1}$ , which represents the activation rate of F2 by MASP1. $k_{catF2}^{MASP2}$ , representing the activation rate of F2 by MASP2, and $k_{F11F2a}^{-}$ representing the disassociation of F2a from F11 in the amplification loop, have a moderately significant impact on F2a. **(B)** Sensitive parameters to the F11a: the most significant sensitive parameters are the generation rate of F9a by F11a ( $k_{catF9a}^{F11a}$ ), the production rate of F11a by the HK and F11 complex ( $k_{catF11a}^{HKF11}$ ), and the production rate of F11a by F12a ( $k_{catF11a}^{F12a}$ ). **(C)** Sensitive parameters to the F12a: the parameters that have a highly significant effect on F12a include the dissociation and association rates of F12a and F11, indicated by $k_{F12aF11}^{-}$ and $k_{F12aF11}^{+}$ , and the association and dissociation rates of PKAL and F12a, indicated by $k_{PKALF12a}^{+}$ and $k_{PKALF12a}^{-}$ . The auto-catalytic rate of F12a by F12 and F12a complex ( $k_{catF12a}^{F12F12a}$ ) has a minimally significant impact on F12a. First- and total-order indices are calculated and displayed as bar plots. Bars where the total-order indices (maroon) are larger than the first-order indices (blue) are marked with a star, indicating that the model output is significantly influenced by interactions between the parameters. The colorbar indicates the intensity of the Sobol indices.

**Figure 13:**
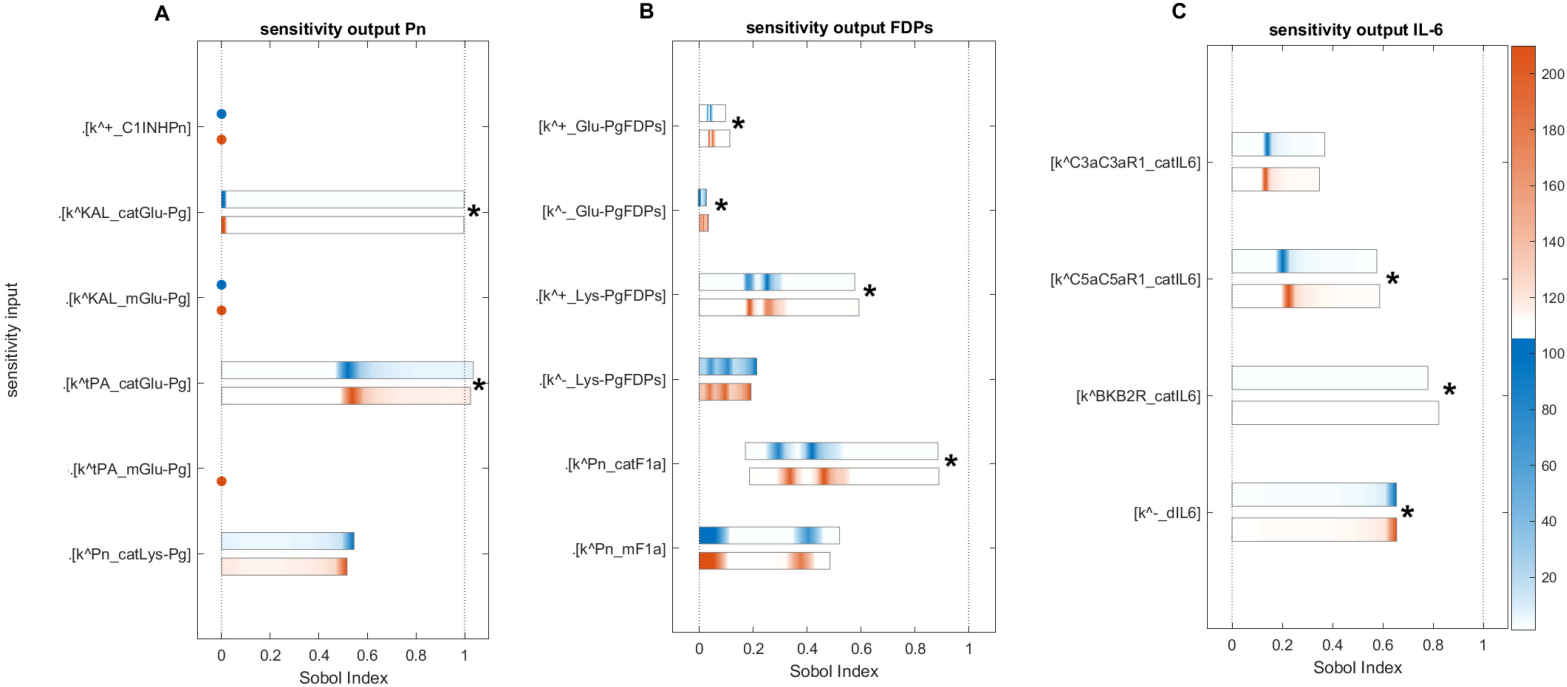
Global sensitivity analysis of the key parameters influencing Pn, FDPs, and IL-6 in the disease state. **(A)** Sensitive parameters to the Pn: the cleavage rates of Glu-Pg by tPA ( $k_{catGlu-Pg}^{tPA}$ ) and by KAL ( $k_{catGlu-Pg}^{KAL}$ ), which have a major and significant impact on Pn. **(B)** Sensitive parameters to the FDPs: the cleavage rate of F1a by Pn ( $k_{catF1a}^{Pn}$ ) and the rate of binding Lys-Pg with FDPs ( $k_{Lys-PgFDPs}^{+}$ ), which have a major and significant impact on FDPs. **(C)** Sensitive parameters to the IL-6: the generation rates of IL-6 by C5a-C5aR1 and BK-B2R complexes, represented by $k_{catIL6}^{C5aC5aR1}$ and $k_{catIL6}^{BKB2R}$ , respectively, and the degradation rate of IL-6, denoted by $k_{dIL6}^{-}$ , significantly affect IL-6 levels. First- and total-order indices are calculated and displayed as bar plots. Bars where the total-order indices (maroon) are larger than the first-order indices (blue) are marked with a star, indicating that the model output is significantly influenced by interactions between the parameters. The colorbar indicates the intensity of the Sobol indices.

In the GSA, we evaluated the impact of species (genes) and parameters perturbations on key biological pathways associated with SARS-CoV-2 infection. The computed Sobol indices quantified the extent to which each gene and parameter influenced these critical pathways, with higher values indicating greater significance. For the species and parameters with total-order indices exceeding their first-order indices were considered to have a significant impact on the interactions. Based on these findings, targets can be prioritized to emphasize those with the greatest impact on disease-related pathways for further investigation. **Figures 8** **–** **10** illustrate the GSA results for species-to-species interactions, while **Figures 12** and **13** present the GSA results for species to parameters.

#### 2.5.1 Sensitivity analysis for F2a

Computed Sobol indices for parameters involved in the state equation of F2a are shown (**Figure 12A**). We recognize that some parameters exhibit significant sensitivity, ranked as follows: the most sensitive parameter mediating F2a levels is the activation rate of F2 by MASP1 ( $k_{catF2}^{MASP1}$ ). Notably, significant interactions are detected for $k_{catF2}^{MASP1}$ with other parameters, as the total-order Sobol indices are greater than the first-order Sobol indices for a majority of computed indices. The associated reaction is labeled as $\prime \prime 3{\text{Ψ}}^{\prime \prime }$ (**Figure 1**). The activation rate of F2 by MASP2 ( $k_{catF2}^{MASP2}$ ) and the dissociation rate of F2a from F11 ( $k_{F11F2a}^{-}$ ) have a moderate effect on F2a but still significantly influence its levels. These associated reactions are labeled as $\prime \prime 3{\text{Ψ}}^{\prime \prime }$ and $\prime \prime 1\gamma^{\prime \prime }$ , respectively.

In contrast, the binding rate of F2a to F1 ( $k_{F2aF1}^{+}$ ) and the unbinding rate of F2a from F1 ( $k_{F2aF1}^{-}$ ) have small Sobol indices, indicating low sensitivity and a minor effect on F2a levels. The associated reaction is labeled as $\prime \prime 1\kappa^{\prime \prime }$ . Regarding the regulation of F2a by FH, the rate at which FH binds to F2a ( $k_{FHF2a}^{+}$ ) shows slight sensitivity. Meanwhile, $k_{F11F2a}^{+}$ , which represents the binding of F2a to F11, demonstrates low sensitivity. Both rates individually contribute to the variation in the F2a response but have a limited overall impact.

The sensitivity of the CS to alter the rate of inhibition of F2a by FH suggests that the regulatory mechanism of F2a by FH plays a pivotal role in the management of thrombosis. In the intervened disease state, an increasing pattern emerged for F2a response due to decrease in FH levels (**Figure 2B**). Suppression of MASP1 and MASP2, along with the potentiation of FH, could significantly contribute to thrombosis management during severe COVID-19.

#### 2.5.2 Sensitivity analysis for F11a

Computed Soboi indices for parameters are involved in the state equation of F11a (**Figure 12B**). For $k_{catF9a}^{F11a}$ , $k_{catF11a}^{HKF11}$ , and $k_{catF11a}^{F12a}$ total-order Sobol indices are greater than first-order Sobol indices for many of the computed Sobol indices. This indicates that these parameters play an important role in influencing the F11a response. They are ranked as follows: $k_{catF9a}^{F11a}$ , referring to the generation of F9a by F11a, and $k_{catF11a}^{HKF11}$ , representing the production of F11a by HK and the F11 complex, are the most sensitive parameters affecting the induction of F11a. In contrast, $k_{catF11a}^{F12a}$ , indicating the production of F11a by F12a, is moderately sensitive and impacts the generation of F11a. Additionally, $k_{F11aAT3}^{+}$ , indicating AT3-mediated regulation of F11a, and $k_{F11aF9}^{+}$ , which is the association rate of F11a to F9, are also moderately sensitive. Finally, $k_{F11aF9}^{-}$ , which refers to the unbinding rate of the F11a-F9 complex, exhibits low sensitivity and has only a minor effect on F11a levels.

This investigation indicates that F11a could be controlled via an external modulator, heparin, as a potentiator of AT3, serving as a target. Moreover, the sensitivity of the system to changes in the parameters associated with the products F11a-F9, HK-F11, and F12a-F11 suggests that preventing the formation of these products could play an effective role in the suppression of coagulation.

#### 2.5.3 Sensitivity analysis for F12a

Computed Sobol indices for parameters involved in the state equation of F12a are shown (**Figure 12C**). The reactions associated with the binding and unbinding of F12a and F11 and those involving PKAL and F12a are found to have the most significant effect on F12a. Their sensitivity rankings, in order, are $k_{F12aF11}^{-}$ , $k_{F12aF11}^{+}$ , $k_{PKALF12a}^{+}$ , and $k_{PKALF12a}^{-}$ , with all being highly sensitive parameters. Furthermore, $k_{catF12a}^{KAL}$ , which represents the production of F12a by KAL, and $k_{catF12a}^{F12F12a}$ , which represents the auto-catalysis of F12a amplification by F12-F12a complex both play significant roles in increasing the effect on F12a levels.

Additionally, in the cross-talk activities between the coagulation and complement pathways, the activation rate of C1r by F12a, denoted as $k_{catCr1}^{F12a}$ , is mildly sensitive and moderately affects F12a. AT3 inhibits F12a, and the rate of binding of AT3 to F12a, denoted by $k_{F12aAT3}^{+}$ , has a negligible decreasing effect on F12a levels.

Moreover, for some parameters, the total-order indices statistically do not exceed first-order indices, implying that these parameters have no significant impact on the F12a response. Such parameters include $k_{CoV2SF12}^{+}$ , which indicates the association rate of the spike protein of SARS-CoV-2 to F12 and does not strongly impact F12a; F12a:F11 amplification of F12a occurs only minimally with the rate of production $k_{catF11a}^{F12a}$ ; and $k_{F12F12a}^{-}$ , referring to the unbinding rate of F12-F12a, has a very minor effect on F12a.

This analysis indicates that the contact system cascade activation mechanism by KAL and positive feedback activity by F12-F12a played an important role in the induction of the coagulation cascade.

#### 2.5.4 Sensitivity analysis for Pn

Computed Sobol indices for the parameters involved in the state equation of Pn are shown (**Figure 13A**). The results reveal several important parameters ranked as follows: the most sensitive parameter, $k_{catGlu-Pg}^{tPA}$ , involved in the catalytic activity of Glu-Pg in the Michaelis–Menten kinetic reaction (M-MKR) causes the cleavage of Pn, which significantly impacted on Pn production. For $k_{catGlu-Pg}^{tPA}$ , the major number of indices fall between ~0.49 and ~0.921 after iteration 2678 within the time span of 8.5–120 min. The reaction Glu-Pg → Pn, involving tPA, is shown in **Figure 1**. Similarly, $k_{catGlu-Pg}^{KAL}$ , involved in the catalytic activities of Glu-Pg by KAL, also causes the cleavage of Pn in M-MKR. $k_{catGlu-Pg}^{KAL}$ is found to be the most sensitive parameter and significantly affects Pn generation. The interactions of KAL with GLu-Pg is labeled as $\prime \prime 2\rho^{\prime \prime }$ in **Figure 1**.

In the propagation phase and amplification mechanism of fibrinolysis, Pn can amplify Lys-Pg (43, 85). $k_{catLys-Pg}^{Pn}$ , which refers to the generation rate of Lys-Pg by Pn, moderately affects Pn levels but does not significantly impact Pn when considered alongside other KCs, as for $k_{catLys-Pg}^{Pn}$ , total-order Sobol indices less than first-order Sobol indices.

For evaluating the expression dynamic of Pn in the treatment state with TXA treatment, the target is tPA. TXA therapy demonstrates a strong restorative effect on Pn levels (**Figure 3B**). This experimental study (86) aligns well with the computational result, as it employed tPA as the target for treating COVID-19 patients.

#### 2.5.5 Sensitivity analysis for FDPs

Computed Sobol indices for the parameters involved in the state equation of FDPs are shown (**Figure 13B**). The degradation of F1a through cleavage by Pn and the induction of FDPs, driven by the catalytic activities of the Michaelis–Menten kinetic reaction, played a critical role. Among these, $k_{catF1a}^{Pn}$ is identified as the most influential parameter, significantly affecting the generation of FDPs in conjunction with other parameters. For $k_{catF1a}^{Pn}$ , the Sobol indices range between ~0.2 and ~0.9, with first-order Sobol indices less than total-order Sobol indices. The interaction labeled as $\prime \prime 8\delta^{\prime \prime }$ (**Figure 1**) highlights the involvement of Pn in the reaction F1a → FDPs. Additionally, the parameter $k_{Lys-PgFDPs}^{+}$ is found to be statistically significant, with total-order Sobol indices greater than first-order Sobol indices. This reaction is labeled as $\prime \prime 11\delta^{\prime \prime }$ .

Furthermore, $k_{Glu-PgFDPs}^{-}$ , representing the unbinding rate of Glu-Pg and FDPs, exhibits low sensitivity but plays a significant role in increasing the impact of FDPs in conjunction with other KCs. For $k_{Glu-PgFDPs}^{-}$ , a maximum first-order index ~0.02 and maximum total-order Sobol index ~0.034, with first-order Sobol indices less than total-order Sobol indices. The binding rate of Glu-Pg to FDPs, represented as $k_{Glu-PgFDPs}^{+}$ , has computed Sobol indices that are low but significant. The reaction is labeled as $\prime \prime 10\delta^{\prime \prime }$ (**Figure 1**).

These results suggest that the reactions associated with F1a and Pn play a major role in the production of FDPs. Additionally, the parameter associated with tPA-mediated induction of Pn significantly impacts Pn levels. tPA is targeted in the treatment state with TXA treatment, and it effectively restores the levels of FDPs when administered in a one-to-one ratio with the level of tPA (**Figure 4B**).

#### 2.5.6 Sensitivity analysis for IL-6

Computed Sobol indices for the KCs governing the state equation of IL-6 (**Figure 13C**). The parameters include $k_{catIL6}^{C5aC5aR1}$ , $k_{catIL6}^{C3aC3aR1}$ , and $k_{catIL6}^{BKB2R}$ , which represent the rates of IL-6 generation by the C5a-C5aR1, C3a-C3aR1, and BK-B2R complexes, respectively.

For $k_{catIL6}^{C5aC5aR1}$ , the measured Sobol indices, with first-order Sobol indices less than total-order Sobol indices, indicate its substantial role in the interactions between parameters influencing the levels of IL-6. Furthermore, small Sobol indices with first-order Sobol less than total-order Sobol indices for $k_{catIL6}^{BKB2R}$ suggest that it contributes minimally, and its interactions with other parameters have minor effect on IL-6 production. Regarding $k_{catIL6}^{C3aC3aR1}$ , the measured first- and total-order Sobol indices, with first-order Sobol indices greater than total-order Sobol indices, imply that it does not exhibit significant interactions with other parameters. However, it does make a moderate individual contribution to the generation of IL-6 variance. The degradation rate of IL-6, represented by $k_{dIL6}^{-}$ , is identified as the most sensitive parameter. It exhibits high Sobol indices with total-order Sobol indices greater than first-order Sobol indices, implying significant interactions with other parameters.

Interestingly, in the disease state, the IL-6 response (**Figure 5A**) shows an increasing trend toward a stationary phase. This suggests that the factors driving this increase involve reactions associated with $k_{catIL6}^{C5aC5aR1}$ , $k_{catIL6}^{BKB2R}$ , and $k_{catIL6}^{C3aC3aR1}$ . The reactions labeled as $\prime \prime 3\phi^{\prime \prime }$ for C5a-C5aR1 → IL-6, C3a-C3aR1 → IL-6, and $\prime \prime \tau^{\prime \prime }$ for BK-B2R → IL-6 are shown in **Figure 1**. In the context of the treatment state with avdoralimab-treatment targeting C5aR1, IL-6 levels are effectively controlled.

## 3 Discussion

In the current study, a quantitative system-level model is developed to better understand the complex interactions and effects between the activation and regulation activities of the integrated hemostatic and complement systems in COVID-19. SARS-CoV-2 has caused deleterious effects on the coagulation and fibrinolytic cascades. Moreover, complement cascades, including the lectin, classical, and alternative pathways, are affected. The model is constructed and calibrated using known parameters and the concentration levels of the entities in symptomatic COVID-19 cases.

In the disease state, certain HS factors, including F2a, tPA, Pn, and FDPs, increasing trends are consistent with previous experimental studies. These factors have been reported as high as a result of COVID-19-associated coagulopathy (CAC) (88, 89). IL-6 levels are increased, wherein C5a via C5aR1 can significantly induce IL-6 (49). The high induction of IL-6 can contribute to thromboinflammation (90). Fibrinolysis increases during COVID-19 due to elevated IL-6 levels, and it has been reported that IL-6 levels are associated with high levels of fibrinogen (F1) (91). The CS terminal complement complex (TCC; C5b-9) is overactivated, which is in good agreement with experimental findings. In the plasma of patients with SARS-CoV-2 infection, C5b-9 levels are significantly elevated, leading to thromboembolic complications and increased mortality rates (92). The high C5b-9 level can promote endothelial injury and dysfunction through multiple mechanisms (93). Furthermore, it has pro-thrombotic properties that can increase prothrombin (F2) levels (94). The regulation in the levels of entities, from peak amplitudes to lower levels, reflect the potent regulatory effect of unperturbed inhibitors, particularly C1INH and FH, on the systems.

**Figure 14:**
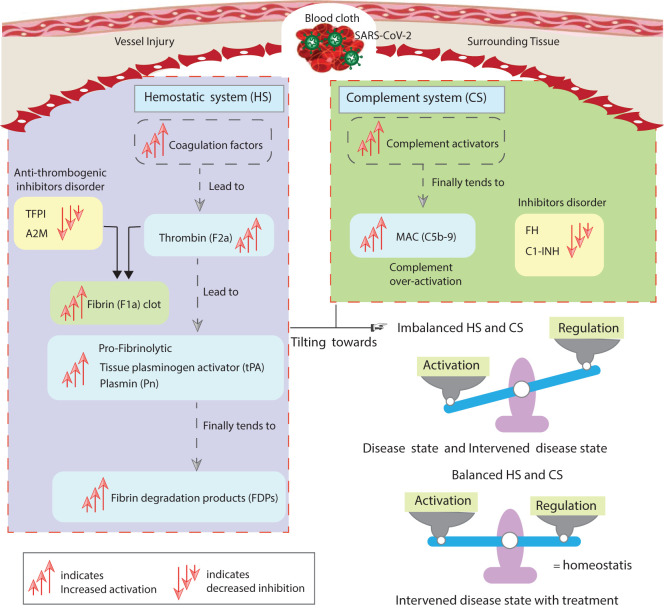
The study’s implications. The disordered inhibitors in the hemostatic and complement systems increased the activation of hemostatic factors. The activation of components of the complement system also increases. The main highly activated entities of both systems include F2a, thrombin; tPA, tissue plasminogen activator; Pn, plasmin; FDPs, fibrin degradation products; and C5b-9, terminal complement complex. The suppressed inhibitors comprise TFPI, tissue factor pathway inhibitor; A2M, alpha-2-macroglobulin; FH, complement factor H; and C1INH, C1-inhibitor. In the state of unhealthy symptomatic COVID-19 patients, referred to as the disease state, and during decreased levels of the inhibitors termed as the intervened disease state, there is an imbalanced activation and regulation of HS and CS entities. Meanwhile, drug intervention tends to regulate these behaviors towards homeostasis. The computational results are in good agreement with experimental studies (8, 87).

In the intervened disease state modeling, perturbations such as a decrease in the basal levels of inhibitors including C1INH, FH, TFPI, and A2M not only affected the amplitudes of the HS and CS entities but also influenced their trends of change. The F2a response increased to a stable high level in perturbation compared to the disease state. The disruption in the inhibitors had a minor impact on the fibrinolytic response, resulting in a slight increase in the levels of Pn and FDPs. This observation aligns with hyper-coagulation and hyper-inflammation response in COVID-19. Deficiencies in FH have been reported in hemolytic-uremic syndrome (HUS) patients during SARS-CoV-2 infection (95, 96). Furthermore, due to perturbations in the levels of inhibitors, the increasing trend of IL-6 changed compared to its response in the disease state. The impairment in the inhibitors has led to elevated levels of C5b-9, which could potentially increase the level of F2, thereby amplifying the levels of F5a, F8a, and F11a through re-induction mechanisms, increasing the likelihood of hypercoagulation. The overall dynamics of the impaired signaling pathways in the hemostatic and complement systems are shown in **Figure 14**.

The overactive response of entities is mitigated via drug intervention modeling. In the treatment state via heparin, TXA, avdoralimab, garadacimab, and TCZ result in restorative effects on the levels of the entities. Heparin, a potentiator of AT3, can increase the inhibition activity of AT3. As a result, the generation of F2a is suppressed and restored. TXA, an inhibitor of tPA, can suppress tPA, resulting in the restoration of Pn and FDPs. The lowered levels of tPA, Pn, and FDPs indicate that fibrinolysis is controlled. The C5aR1 inhibitor, avdoralimab, can regulate C5aR1 expression. Reducing C5aR1 levels leads to lower IL-6 levels. Additionally, TCZ, an anti-IL-6R antibody, effectively suppresses the IL-6-IL-6R complex. This suggests that inflammation can be managed through these mechanisms. Furthermore, heparin, an inhibitor of C2-9 complements, forms complexes with these complements ( $Hep:C_{n}$ , where n=2 to 9). By interacting with complement proteins, heparin regulates their activity, thereby controlling C5b-9 levels.

In GSA, FH and C1INH are found to be prominent inhibitors. C1INH and FH emerge as highly significant inhibitors that effectively influence the entities response. Furthermore, TFPI and A2M show low sensitivity in influencing the response of the entities, implying that C1INH and FH regulate the cascades of the systems more rigorously compared to TFPI and A2M. Furthermore, in the analysis, KCs associated with MASP1 and MASP2 are identified as the most sensitive for affecting F2a, making them potential candidates. The sensitivity of the system to changes in the activation rates of F2 suggested that the activation mechanisms by MASP1 and MASP2 play a crucial role in thrombosis during the increasing trend of coagulation. The analysis for Pn revealed that critical reactions associated with tPA and KAL played a major role in upregulating the fibrinolytic response. The critical reactions associated with C5aR1 played an essential role in the over-expression of IL-6.

The SA results were not cross-validated with experimental data, which represents a limitation of this study. Future work should aim to validate these findings through experimental approaches to enhance their biological relevance. Integrating experimental data will help improve the robustness and applicability of the SA. This step is essential to ensure the reliability and practical significance of the insights derived from the model.

To enhance the clinical relevance of this study, we provide additional interpretation of our findings within real-world therapeutic contexts. The results underscore the importance of careful drug selection and dosing strategies to optimize patient outcomes. For instance, the selection of heparin for anticoagulation must account for individual bleeding risks, while dosing adjustments are critical to minimize complications. Similarly, the use of TXA should be carefully considered in patients with a predisposition to thromboembolic events to balance its antifibrinolytic benefits against potential thrombotic risks.

Emerging therapies, such as avdoralimab, garadacimab, and TCZ, highlight the potential for targeted approaches in controlling inflammation and reducing disease severity. However, these novel treatments require further evaluation in clinical settings to refine dosing protocols and mitigate risks such as immunological reactions or hypersensitivity. These findings provide valuable insights for clinicians in designing personalized treatment regimens that maximize therapeutic efficacy while minimizing adverse effects. By bridging the gap between experimental results and clinical application, this study contributes to the development of effective and safe therapeutic strategies for managing complex condition like COVID-19.

The model developed in this study could also be adapted for other diseases, such as sepsis. In sepsis, hyper-activation of the coagulation and complement systems leads to disseminated intravascular coagulation (DIC) and widespread inflammatory responses. Our model can be extended by incorporating parameters related to inflammatory mediators and organ-specific effects observed in septic patients. This would allow for the exploration of therapeutic interventions targeting the coagulation–complement axis in sepsis.

Additionally, the model could be applied to systemic lupus erythematosus (SLE), a chronic autoimmune disease where immune-complex-mediated complement activation contributes to pro-thrombotic states. By including features such as autoimmune triggers, chronic complement activation, and endothelial cell damage, the model could provide valuable insights into disease progression and the effectiveness of treatments targeting the complement and coagulation pathways in SLE.

In this study, the findings are based on computational simulations that provide valuable insights into potential therapeutic targets. However, it is important to emphasize that these results should be validated through experimental data. We recommend that future research incorporate experimental approaches, such as gene knockdown/overexpression or perturbation assays, to validate the predictions made by the model and further refine the therapeutic strategies proposed in this study.

Based on the computational predictions from our study, several potential therapeutic targets have been identified for further investigation. These targets, which play crucial roles in the biological pathways associated with SARS-CoV-2 infection, sepsis, and SLE, can guide drug development efforts. To advance this process, we recommend experimental validation of the identified targets. Once key targets are validated, high-throughput screening of small molecules can be performed to identify compounds that modulate their activity. Computational docking studies can then predict potential interactions between these targets and drug candidates.

To further evaluate the therapeutic potential of the identified compounds, preclinical *in vivo* models should be employed to assess their efficacy, safety, and pharmacokinetics. The findings from these preclinical studies can subsequently inform the design of clinical trials, with a focus on identifying biomarkers for patient stratification and evaluating the therapeutic effects of the drugs across diverse patient populations.

## 4 Conclusion

We have developed a comprehensive systems immunology model to study the interactions between the hemostatic and complement systems activated by SARS-CoV-2. This study analyzes the concentration dynamics of key components, including F2a, tPA, Pn, FDPs, IL-6, the IL-6-IL-6R complex, and C5b-9, under three distinct conditions, highlighting characteristic trends. In the disrupted disease state, changes in the behavior of these components are primarily driven by reduced levels of inhibitors such as FH, C1INH, TFPI, and A2M. The decreased levels of FH and C1INH are major factors contributing to an exaggerated response in both the hemostatic and complement systems. In the treatment context, thrombosis can be managed by enhancing AT3 activity, which helps restore F2a levels. Fibrinolysis is regulated by reducing Pn and FDPs through the suppression of tPA. Targeting C5aR1 and IL-6R can help mitigate the inflammatory response. Additionally, targeting complement proteins C1 through C9 helps regulate C5b-9 levels, ensuring a balanced immune response. Our findings highlight the crucial roles of key regulators and effectors within the complement and hemostatic cascades, revealing their intricate contributions to immune modulation and homeostasis. The complement system is tightly regulated by factors such as FH, C1INH, MASP-1, MASP-2, C3, C5, and the C5a-C5aR1 and C3a-C3aR1 complexes, which collectively shape immune responses and inflammation. In parallel, the hemostatic system is governed by essential factors such as TFPI, A2M, AT3, F2a, F11a, F12a, tPA, Pn, F1a, and the BK-B2R complex, ensuring the delicate balance between coagulation and fibrinolysis. These regulatory networks are pivotal in maintaining immune and hemostatic equilibrium, and their dysregulation may contribute to the pathogenesis of immune-related disorders. Our work underscores the potential of targeting these pathways for the development of novel therapeutic strategies aimed at restoring immune balance and mitigating disease. The results can also help identify which biomarkers are suitable for assessing the extent of dysregulation in the hemostatic and complement systems. The computational modeling presented in this study has several strengths. It provides a systematic approach to analyzing complex biological pathways associated with SARS-CoV-2 infection, facilitating the identification of potential therapeutic targets. By leveraging available data, the model simulates molecular interactions and predicts the effects of interventions, offering valuable insights to guide experimental studies and therapeutic development. However, the study also has limitations, as it raises many questions that require further experimental validation. In future work, we plan to conduct *in vitro* experiments to investigate our predictions in greater detail and provide explicit insights into the diagnosis and treatment of COVID-19, focusing on key targets in the hemostatic and complement pathways.

## Data Availability

Publicly available datasets were analyzed in this study. This data can be found here: https://www.ncbi.nlm.nih.gov/geo/query/acc.cgi?acc=GSE177477.

## Supplementary material

SUPPLEMENTARY MATERIAL The Supporting Information (SI) includes the following: (I) Files (S1–S12) related to the ODE-based modeling framework, which include a simplified representation of the hemostatic and complement systems, the research methodology workflow, the system of ODEs, modeling codes, and gene expression data with an analysis of differentially expressed genes. (II) Tables (S1–S3) detailing kinetic constants, concentration levels of entities in the hemostatic and complement systems, and drug information, including dosages. (III) Figures (S1–S5).
