# Supplementary material for: Unravelling the impact of SARS-CoV-2 on hemostatic and complement systems: a systems immunology perspective
Source: Front Immunol. 2025 Jan 13;15:1457324. doi: 10.3389/fimmu.2024.1457324 (PMC11781117; doi:10.3389/fimmu.2024.1457324)
Supplement: SUPPLEMENTARY MATERIAL — The Supporting Information (SI) includes the following: (I) Files (S1–S12) related to the ODE-based modeling framework, which include a simplified representation of the hemostatic and complement systems, the research methodology workflow, the system of ODEs, modeling codes, and gene expression data with an analysis of differentially expressed genes. (II) Tables (S1–S3) detailing kinetic constants, concentration levels of entities in the hemostatic and complement systems, and drug information, including dosages. (III) Figures (S1–S5). [file DataSheet1.pdf]

Supplementary Material

Differential Expression R Code

```
suppressPackageStartupMessages({library("maEndToEnd")})


if (!require("BiocManager"))
  install.packages("EnhancedVolcano")
BiocManager::install("maEndToEnd", version = "devel")
BiocManager::install(version = '3.16')
library(BiocManager)
# if (!requireNamespace("BiocManager", quietly = TRUE))
#   install.packages("BiocManager")
# BiocManager::install("hgu133a.db")

if (!requireNamespace("BiocManager", quietly = TRUE))
  install.packages("BiocManager")

BiocManager::install("EnhancedVolcano")
BiocManager::install("pd.clariom.s.human")
BiocManager::install("clariomdhumantranscriptcluster.db")
#General Bioconductor packages
library(Biobase)
library(oligoClasses)
install.packages("pd.clariom.s.human")
#Annotation and data import packages
library(ArrayExpress)
library(pd.clariom.s.human)
library(clariomdhumantranscriptcluster.db)
library(AnnotationDbi)
library(org.Hs.eg.db)
# library(hgu133plus2.db)

#Quality control and pre-processing packages
library(oligo)
library(arrayQualityMetrics)
```

```
#Analysis and statistics packages
library(limma)
library(topGO)
library(ReactomePA)
library(clusterProfiler)

#Plotting and color options packages
library(gplots)
library(ggplot2)
library(geneplotter)
library(RColorBrewer)
library(pheatmap)
library(viridis)
#Formatting/documentation packages
#library(rmarkdown)
#library(BiocStyle)
library(dplyr)
library(tidyr)

#Helpers:
library(stringr)
library(matrixStats)
library(genefilter)
library(openxlsx)
library(devtools)

####Downloading the raw data from ArrayExpress
#D:\PhD\NGS_work\pancreatic_cancer\Adjacent\E-GEOD-15471
raw_data_dir <- "raw_data_dir"
```

```r
if (!dir.exists(raw_data_dir)) {
  dir.create(raw_data_dir)
}
#anno_AE <- getAE("E-GEOD-48121", path =
#raw_data_dir, type = "raw")

####Import of annotation data and microarray
#expression data as "ExpressionSet"

sdrf_location <- file.path(raw_data_dir, "GSE177477.sdrf.txt")
SDRF <- read.delim(sdrf_location)
write.csv(SDRF,"SDRF.csv")
rownames(SDRF) <- SDRF$Array.Data.File
SDRF <- AnnotatedDataFrame(SDRF)
pData(SDRF)

raw_data <- oligo::read.celfiles(filenames = file.path(raw_data_dir,
                                                        SDRF$Array.Data.File),
                                  verbose = FALSE, phenoData = SDRF)
stopifnot(validObject(raw_data))

head(Biobase::pData(raw_data))
Biobase::pData(raw_data)
```

```
#selecting columns of interest
Biobase::pData(raw_data) <- Biobase::pData(raw_data)[, c("Array.Data.File",
                                                          "FactorValue..clinical.information.")]
pData(raw_data)$Array.Data.File
pData(raw_data)$FactorValue..clinical.information.
#pData(raw_data)$FactorValue..time.


#Quality control of the raw data
#show 1 to 5 columns and rows
Biobase::exprs(raw_data)[1:5, 1:5]

#log2, principal component analysis (PCA) and plot
exp_raw <- log2(Biobase::exprs(raw_data))
write.csv(exp_raw,"exp_raw.csv")
sum(is.na(exp_raw))

#exp_raw <- scale(exp_raw)

PCA_raw <- prcomp(t(exp_raw), scale. = FALSE)
percentVar <- round(100*PCA_raw$sdev^2/sum(PCA_raw$sdev^2),1)
sd_ratio <- sqrt(percentVar[2] / percentVar[1])
dataGG <- data.frame(PC1 = PCA_raw$x[,1], PC2 = PCA_raw$x[,2],
                Disease = pData(raw_data)$FactorValue..clinical.information.,
                Individual = pData(raw_data)$Array.Data.File)

pdf("PCA_before_normalization.pdf",width=10,height=7,paper='special')
ggplot(dataGG, aes(PC1, PC2)) +
  geom_point(aes(shape = Disease, color = Disease)) +
  ggtitle("PCA plot of the log-transformed raw expression data") +
  xlab(paste0("PC1, VarExp: ", percentVar[1], "%")) +
  ylab(paste0("PC2, VarExp: ", percentVar[2], "%")) +
  theme(plot.title = element_text(hjust = 0.5))+
  coord_fixed(ratio = sd_ratio) +
  scale_shape_manual(values = c(4,15)) +
  scale_color_manual(values = c("darkorange2", "dodgerblue4" ))
dev.off()
#bowplot
# oligo::boxplot(raw_data, target = "core",
#                main = "Boxplot of log2-intensitites for the raw data")
library(viridis)
pdf("Boxplot_before_normalization.pdf",width=10,height=7,paper='special')
oligo::boxplot(raw_data, color="blue", target = "core",
               col=viridis_pal(option = "A")
               (n=90), las=2, cex.axis=0.35,
               main = "Boxplot of log2-intensitites for the raw data")

dev.off()
```

```
#library(hexbin)

arrayqualitybefore <- file.path(getwd(), "arrayqualitymetrics_BEfore_RMA")
if(!dir.exists(arrayqualitybefore)){
  dir.create(arrayqualitybefore)
}
#report for our raw data
arrayQualityMetrics(expressionset = raw_data,
                    outdir = arrayqualitybefore,
                    force = TRUE, do.logtransform = TRUE,
                    intgroup = c("FactorValue..clinical.information."))

#Background adjustment, calibration, summarization and annotation
#head(ls("package:hgu133a.db"))

#####Relative Log Expression data quality analysis
palmieri_eset <- oligo::rma(raw_data, normalize = FALSE)

exp_pal_raw <- Biobase::exprs(palmieri_eset)
write.csv(exp_pal_raw,"MLraw_data15471.csv")
row_medians_assayData <-
  Biobase::rowMedians(as.matrix(Biobase::exprs(palmieri_eset)))

RLE_data <- sweep(Biobase::exprs(palmieri_eset), 1, row_medians_assayData)

RLE_data <- as.data.frame(RLE_data)
RLE_data_gathered <-
  tidyr::gather(RLE_data, patient_array, log2_expression_deviation)
pdf("RLE_before_normalization.pdf",width=10,height=7,paper='special')
ggplot2::ggplot(RLE_data_gathered, aes(patient_array,
                                       log2_expression_deviation)) +
  geom_boxplot(outlier.shape = NA) +
  ylim(c(-2, 2)) +
  theme(axis.text.x = element_text(colour = "aquamarine4",
                                   angle = 60, size = 6.5, hjust = 1 ,
                                   face = "bold"))
dev.off()
RLE_data_gathered
#RMA calibration of the data
palmieri_eset_norm <- oligo::rma(raw_data)

palmieri_eset_norm
[illegible]
```

```
#RLE after normalization
row_medians_assayData <-
  Biobase::rowMedians(as.matrix(Biobase::exprs(palmieri_eset_norm)))

RLE_data <- sweep(Biobase::exprs(palmieri_eset_norm), 1, row_medians_assayData)

RLE_data <- as.data.frame(RLE_data)
RLE_data_gathered <-
  tidyr::gather(RLE_data, patient_array, log2_expression_deviation)

pdf("RLE_after_normalization.pdf",width=10,height=7,paper='special')
ggplot2::ggplot(RLE_data_gathered, aes(patient_array,
                                       log2_expression_deviation)) +
  geom_boxplot(outlier.shape = NA) +
  ylim(c(-2, 2)) +
  theme(axis.text.x = element_text(colour = "aquamarine4",
                                   angle = 60, size = 6.5, hjust = 1 ,
                                   face = "bold"))
dev.off()


#Quality assessment of the calibrated data
write.csv(exp_palmieri,"Normalized_exp.csv")
exp_palmieri <- Biobase::exprs(palmieri_eset_norm)
PCA <- prcomp(t(exp_palmieri), scale = FALSE)

percentVar <- round(100*PCA$sdev^2/sum(PCA$sdev^2),1)
sd_ratio <- sqrt(percentVar[2] / percentVar[1])

dataGG <- data.frame(PC1 = PCA$x[,1], PC2 = PCA$x[,2],
                     Disease =
Biobase::pData(palmieri_eset_norm)$FactorValue..clinical.information.)

pdf("PCA_after_normalization.pdf",width=10,height=7,paper='special')
ggplot(dataGG, aes(PC1, PC2)) +
  geom_point(aes(shape = Disease, colour = Disease)) +
  ggtitle("PCA plot of the calibrated, summarized data") +
  xlab(paste0("PC1, VarExp: ", percentVar[1], "%")) +
  ylab(paste0("PC2, VarExp: ", percentVar[2], "%")) +
  theme(plot.title = element_text(hjust = 0.5)) +
  coord_fixed(ratio = sd_ratio) +
  scale_shape_manual(values = c(4,15)) +
  scale_color_manual(values = c("darkorange2", "dodgerblue4"))
dev.off()
```

```
# oligo::boxplot(palmieri_eset_norm, target = "core",
#         main = "Boxplot of log2-intensitites for the normalized data")
pdf("Boxplot_after_normalization.pdf",width=10,height=7,paper='special')
oligo::boxplot(palmieri_eset_norm, color="blue",
target = "core", col=viridis_pal(option = "A")
                    (n=90), las=2, cex.axis=0.35,
        main = "Boxplot of log2-intensitites for the Normalized data")
dev.off()

#Heatmap clustering analysis
pData(palmieri_eset_norm)$FactorValue..clinical.information.


sampletype<- str_replace_all(Biobase::pData(palmieri_eset_norm)
                $FactorValue..clinical.information.," ", "")
sampletype

disease_names <- ifelse(str_detect(sampletype,
                                    "Symptomatic"), "Covid", "Normal")

# phenotype_names
# disease_names <- ifelse(str_detect(pData
#  (palmieri_eset_norm)$FactorValue..category.0...normal..1...
#hpv.without.progression..2...hpv.with.progression.,
#"normal"), "Normal", disease_names <- ifelse(str_detect(pData
#(palmieri_eset_norm)$FactorValue..category.0...normal..1...hpv.
#without.progression..2...hpv.with.progression.,
#"cancer"),  "Cancer", "hpvNorm"))

disease_names

annotation_for_heatmap <- data.frame( Covid = disease_names)
annotation_for_heatmap
row.names(annotation_for_heatmap) <- row.names(pData(palmieri_eset_norm))
row.names(pData(palmieri_eset_norm))
##
dists <- as.matrix(dist(t(exp_palmieri), method = "manhattan"))
dists
rownames(dists) <- row.names(pData(palmieri_eset_norm))
rownames(dists)
hmcol <- rev(colorRampPalette(RColorBrewer::brewer.pal(9, "YlOrRd"))(255))
colnames(dists) <- NULL
diag(dists) <- NA
```

```
ann_colors <- list(
  Disease = c(Covid = "blue4", Normal = "cadetblue2"))
pdf("Heatmap.pdf",width=10,height=7,paper='special')
#pdf("Heatmap.pdf",width=10,height=7,paper='special')
pheatmap(dists, col = (hmcol),
         annotation_row = annotation_for_heatmap,
         annotation_colors = ann_colors,
         legend = TRUE,
         treeheight_row = 0,
         legend_breaks = c(min(dists, na.rm = TRUE),
                           max(dists, na.rm = TRUE)),
         legend_labels = (c("small distance", "large distance")),
         main = "Clustering heatmap")
dev.off()
#Filtering based on intensity

palmieri_medians <- rowMedians(Biobase::exprs(palmieri_eset_norm))

#pdf("hist1.pdf")
hist_res <- hist(palmieri_medians, 100, col = "cornsilk1", freq = FALSE,
                 main = "Histogram of the median intensities",
                 border = "antiquewhite4",
                 xlab = "Median intensities")

#choose a threshold of 4
man_threshold <- 4

pdf("Intensities_with_threshold.pdf",width=10,height=7,paper='special')
hist_res <- hist(palmieri_medians, 100, col = "cornsilk", freq = FALSE,
                 main = "Histogram of the median intensities",
                 border = "antiquewhite4",
                 xlab = "Median intensities")
abline(v = man_threshold, col = "coral4", lwd = 2)
dev.off()

WGCNAdata = exp_palmieri[DEG_P$PROBEID,]
write.csv(WGCNAdata,"WGCNAdata_18670_02.csv")

Biobase::pData(palmieri_eset_norm)

no_of_samples <-
  table(paste0(pData(palmieri_eset_norm)$FactorValue..clinical.information.))
no_of_samples
samples_cutoff <- min(no_of_samples)
```

```
idx_man_threshold <- apply(Biobase::exprs(palmieri_eset_norm), 1,
                           function(x){
                             sum(x > man_threshold) >= samples_cutoff})
table(idx_man_threshold)
idx_man_threshold
palmieri_manfiltered <- subset(palmieri_eset_norm, idx_man_threshold)
BiocManager::install("hgu133plus2.db")
library(hgu133plus2.db)
library(clariomdhumantranscriptcluster.db)
#Annotation of the transcript clusters
anno_palmieri <- AnnotationDbi::select(clariomdhumantranscriptcluster.db,
                                       keys = (featureNames(palmieri_eset_norm)),
                                       columns = c("SYMBOL", "GENENAME"),
                                       keytype = "PROBEID")

anno_palmieri <- subset(anno_palmieri, !is.na(SYMBOL))


#Removing multiple mappings

#devtools::install_github("hadley/assertthat")

anno_grouped <- group_by(anno_palmieri, PROBEID)
anno_summarized <-
  dplyr::summarize(anno_grouped, no_of_matches = n_distinct(SYMBOL))

head(anno_summarized)
anno_filtered <- filter(anno_summarized, no_of_matches > 1)
head(anno_filtered)

probe_stats <- anno_filtered
nrow(probe_stats)

ids_to_exlude <- (featureNames(palmieri_manfiltered) %in% probe_stats$PROBEID)

table(ids_to_exlude)

palmieri_final <- subset(palmieri_manfiltered, !ids_to_exlude)

validObject(palmieri_final)

head(anno_palmieri)
```

```
fData(palmieri_final)$PROBEID <- rownames(fData(palmieri_final))

fData(palmieri_final) <- left_join(fData(palmieri_final), anno_palmieri)
exp_palmieri$x <- rownames(exp_palmieri)
normalizeddata<- read.csv("Normalized_exp.csv")
data <- left_join(normalizeddata, anno_palmieri, by=c('X'='PROBEID'))

# restore rownames after left_join
rownames(fData(palmieri_final)) <- fData(palmieri_final)$PROBEID
validObject(palmieri_final)

Biobase::pData(palmieri_final)

com_genes<- "C1S|C1R|C1QA|C1QB|C1QC|C2|C3|C3AR1|C4A|C4B|C5
|C5AR2|C6|C7|C8A|C8B|C9|CD55|CR1|MASP1|MASP2|MBL2|CFB|C1-INH
|Properdin|CFH|CFI|CFD|C4BPA|C4BPB|IGHG1|IGHG2|IGHG3|IGHG4|IL6|IL6R|ACE2"
com_genes_3494 <- subset(data, str_detect(SYMBOL, com_genes))
data <- subset(data, !is.na(SYMBOL))
colnames(data)
data$Avg_covid = rowMeans(data[,c(2:11)])
data$Avg_Normal = rowMeans(data[,c(31:48)])
data$Avg_Asymcovid = rowMeans(data[,c(12:30)])
write.csv(data,"complete.csv")

write.csv(com_genes_3494,"filtered.csv")
```

```r
#A linear model for the data

individual <-
  as.character(Biobase::pData(palmieri_final)$Array.Data.File)

individual

tissue_type <- str_replace_all(Biobase::pData(palmieri_final)
                               $FactorValue..clinical.information.," ", "")
tissue_type



tissue <-
  ifelse(str_detect(tissue_type,
                    "Symptomatic"), "Covid", "Normal")

tissue
design_palmieri_oncolytic <- model.matrix(~ 0 + tissue )
design_palmieri_oncolytic
colnames(design_palmieri_oncolytic)[1:2] <- c("Covid", "Normal")
rownames(design_palmieri_oncolytic) <- individual
design_palmieri_oncolytic
write.csv(design_palmieri_oncolytic,"design matrix1.csv")
```

```
#Contrasts and hypotheses tests
#######################################Comparison1
contrast_matrix_Cov_Nor <- makeContrasts(Covid-Normal,
levels = design_palmieri_oncolytic) contrast_matrix_Cov_Nor
palmieri_fit_Cov_Nor <- eBayes(contrasts.fit(lmFit(palmieri_final,
design_palmieri_oncolytic), contrast_matrix_Cov_Nor))

#Extracting results
table_Cov_Nor <- topTable(palmieri_fit_Cov_Nor, number = Inf)
head(table_Cov_Nor)
write.csv(table_Cov_Nor ,"table_Cov_Nor.csv")

table_Cov_Nor <- subset(table_Cov_Nor, !is.na(SYMBOL))
pdf("P-value_histogram.pdf",width=10,height=7,paper='special')
hist(table_Cov_Nor$P.Value, col = brewer.pal(2, name = "Set2")[1],
     main = "Covid 19 and Normal", xlab = "p-values")
dev.off()
##DEGs by setting cutoff
DEG_Cov_Nor<- subset(table_Cov_Nor, P.Value < 0.05)
colnames(DEG_Tum_Nor)
DEG_Cov_Nor$P.Value
DEG_Cov_Nor <- subset(DEG_Cov_Nor, logFC < -0.5 | logFC > 0.5)
DEG_Cov_Nor$logFC
write.csv(DEG_Cov_Nor, 'DDEG_Cov_Nor.csv')
```

```r
##########################  WGCNA GENE list
DEG_P<- subset(table_Tum_Nor, P.Value < 0.05)
colnames(DEG_P)
DEG_P$P.Value
DEG_P <- subset(DEG_P, logFC < -0.2 | logFC > 0.2)
DEG_P$logFC
write.csv(DEG_P, 'OE/WGCNA_geneList0_2.csv')


DEG_Tum_Nor
library(stringr)
DEG_Tum_Nor_rec <- subset(DEG_Tum_Nor,
str_detect(GENENAME, ".receptor."))

write.csv(DEG_Tum_Nor_rec, 'OE/DEG_Tum_Nor_receptors.csv')
DEG_overexpressed <- subset(DEG_Tum_Nor, logFC > 1)
DEG_overexpressed$logFC
write.csv(DEG_overexpressed, 'OE/DEG_overexpressed.csv')
write.table(DEG_overexpressed$SYMBOL,
file = "OE/DEG_overexpressed.txt", sep = "\n",
            row.names = FALSE)

#################code for specific genes values#####
#strings <- c("EPHA4", " GPRC5A ", " LRP8", " MET ",
#" NDC1", " TNFRSF10A "," TNFRSF10B "," TNFRSF11B ",
#"TNFRSF21"," ADGRF1"," ADGRG6"," ARNTL2"," GABRP ","
#HRH1"," ITPR3"," IFITM1"," IL1RAP "," IL1RN"," IL1R2",
#"IL2RG"," MST1R"," NPR3"," OSMR"," PMEPA1"," PTPN14",
#" REEP3"," RTP4"," TM4SF1"," TMC5"," TMPRSS3","
#TMPRSS4"," TMEM184B"," TMEM41A"," TMEM45B")
DEG_Tum_Nor_specific <- subset(DEG_overexpressed,
str_detect(SYMBOL, "FXYD3|MET|ADGRF1|GABRP|PLAUR|TMPRSS4"))
DEG_Tum_Nor_specific
write.csv(DEG_Tum_Nor_specific, 'OE/DEG_Tum_Nor_specific.csv')

DEG_Tum_Nor_overexpreceptor <- subset(DEG_overexpressed, str_detect
(GENENAME,".receptor.|.transmembrane."))
write.csv(DEG_Tum_Nor_overexpreceptor,
'OE/DEG_Tum_Nor_overexpreceptor.csv')
```

```
########################Valcano
install.packages("EnhancedVolcano")
library(EnhancedVolcano)
pdf("Volcano_plot.pdf",width=10,height=7,paper='special')
EnhancedVolcano(table_Cov_Nor,

                lab = table_Cov_Nor$SYMBOL,

                x = "logFC",

                y = "P.Value",

                pCutoff = 0.05,

                FCcutoff = 0.5,

                title = "Covid 19 & Normal")
dev.off()

#######################################################
library(ReactomePA)
library(reactome.db)
library(dplyr)
library(clusterProfiler)
symbol<-as.character(DEG_Tum_Nor$SYMBOL)
df<-mapIds(org.Hs.eg.db, symbol, 'ENTREZID', 'SYMBOL')
write.xlsx(df,file="OE/ENTREZID.xlsx",sep="\t")
##de<-read.xlsx("Book1.xlsx")
x <- enrichPathway(gene=df,pvalueCutoff=0.05, readable=T)
head(as.data.frame(x))
pdf("Images/enrich_bar_plot.pdf",width=10,height=7,paper='special')
barplot(x, showCategory=8)
dev.off()
pdf("Images/enrich_dot_plot.pdf",width=10,height=7,paper='special')
enrichplot::dotplot(x, showCategory=15)
dev.off()
pdf("Images/enrich_emap_plot.pdf",width=10,height=7,paper='special')
emapplot(x)
dev.off()
pdf("Images/enrich_cnet_plot.pdf",width=10,height=7,paper='special')
cnetplot(x, categorySize="pvalue")
dev.off()
write.xlsx(x,file="enrichpathway.xlsx",sep="\t")
```

```
G_Tum_Nor
ldchange<-DEG_Tum_Nor[,4]
b<-rbind(df,foldchange)
b2<-t(tab)
ite.xlsx(tab2,file="OE/AFTERBIND.xlsx",sep="\t")

a<-read.xlsx("threshold.xlsx")
ym<-aa$Gene.symbol
oga<- aa[,4:5]
e_U<- loga[!(is.na(loga$entrez) | loga$entrez==""), ]

 feature 1: numeric vector
neList1 = tab2[,2]
 feature 2: named vector
mes(geneList1) = as.character(tab2[,1])
 feature 3: decreasing order
neList1 = sort(geneList1, decreasing = TRUE)

neList1
ass(geneList1)
neList1 <- sapply(geneList1, as.numeric)
ass(geneList1)

######clusterprofile
require(clusterProfiler)
#data(genelist1)
res3 <- compareCluster(geneList1, fun="enrichPathway")
dotplot(res3)
################gsea
y1 <- gsePathway(geneList1, nPerm=1000,
                 pvalueCutoff=0.05,
                 pAdjustMethod="BH", verbose=FALSE)
res1 <- as.data.frame(y1)
head(res1)
gseaplot(y1, geneSetID ="R-HSA-6798695")
library(graphite)
viewPathway("Immune System", readable=TRUE,
foldChange=geneList1)
viewPathway("Innate Immune System", readable=TRUE,
foldChange=geneList1)
viewPathway("Transport of small molecules",
readable=TRUE, foldChange=geneList1)
viewPathway("E2F mediated regulation of DNA replication",
readable=TRUE, foldChange=geneList1)
```

```
install.packages("pathfindR")
install.packages("devtools") # if you have not installed this
devtools::install_github("egeulgen/pathfindR")
library(openxlsx)
library(pathfindR)
de<-read.xlsx("FINAL20291.xlsx")
de1<-de[-c(1)]
de1<-de1[-c(7:8)]
de1<-de1[-c(4:5)]
de1<-de1[-c(2)]
symbol<-as.character(de1$SYMBOL)
df<-mapIds(org.Hs.eg.db, symbol, 'ENTREZID', 'SYMBOL')
RA_output <- run_pathfindR(de1)
RA_clustered <- cluster_pathways(RA_output, plot_hmap = TRUE,
plot_clusters_graph = FALSE)
RA_clustered <- cluster_pathways(RA_output, method = "fuzzy")

RLE_data <- sweep(Biobase::exprs(palmieri_eset_norm), 1,
row_medians_assayData)
RLE_data <- as.data.frame(RLE_data)
RLE_data_gathered <-
  tidyr::gather(RLE_data, patient_array, log2_expression_deviation)
pdf("RLEplot.pdf",width=10,height=10,paper='special')
ggplot2::ggplot(RLE_data_gathered, aes(patient_array,
                                       log2_expression_deviation)) +
  geom_boxplot(outlier.shape = NA) +
  ylim(c(-2, 2)) +
  theme(axis.text.x = element_text(colour = "aquamarine4",
                                   angle = 60, size = 6.5, hjust = 1 ,
                                   face = "bold"))
dev.off()
oligo::boxplot( palmieri_eset_norm,las=2, cex.axis= 0.35,
                main = "Boxplot of log2-intensitites of  data")

oligo::boxplot(palmieri_eset_norm,col=viridis_pal(option ="C")
               (n=56),las=2, cex.axis= 0.4,
        main = "Boxplot of log2-intensitites for the normalize data")
```
