# Supplementary material for: Unravelling the impact of SARS-CoV-2 on hemostatic and complement systems: a systems immunology perspective
Source: Front Immunol. 2025 Jan 13;15:1457324. doi: 10.3389/fimmu.2024.1457324 (PMC11781117; doi:10.3389/fimmu.2024.1457324)
Supplement: Supplementary file 2 [file DataSheet2.pdf]

Supplementary Material

DIFFERENTIAL EXPRESSION GENES ANALYSIS

In this study, expression data is quantified, performed quality control normalization, clustering analysis, and differential expression (DE) analysis. The expression data platform Affymetrix Clariom S Assay, Human (includes Pico Assay) is used. Explored raw data CELL files that are imported into a Bioconductor ExpressionSet and the raw data is normalized.

The basic quality control of raw and normalized expression data set is performed and shown via box plots (Fig. S1 and Fig. S2). The box plots indicate log2-intensities of the data. First principal component analysis (PCA) is performed before normalization of the data (Fig. S3). This step is followed by the application of the deconvolution method via oligo package. Performed background correction an essential adjustment for the probe intensities that give accurate measurements of specific array hybridization.

Performed quantile normalization (calibration) and the RMA (robust multichip average) algorithm for summarization. Before calibration and evaluation of the data, another quality control analysis relative Log Expression (RLE) was performed (Fig. S4) for normalized data. Computed median log2-intensity of every transcript across all arrays and plot the RLE (Fig. S5) after normalization. The RLE plots represent the variation in data. After performing RMA, for the calibrated data generate heatmap (Fig. S6) and performed another PCA (Fig. S7).

RLE as a relative measure, focuses on the relative differences in expression levels between samples for each gene. It does not provide absolute expression values but instead compares the expression of each gene in each sample to a reference sample or to the median expression across all samples. RLE involves taking the logarithm such as log2 of the expression values for each gene in each sample. This log transformation helps stabilize the variance of the data and makes it more suitable for assessing relative differences. The heatmap visualizes patterns in gene expression data. The PCA plots depicted the distribution of data points along the first and second principal components (PC1 and PC2) axes. PC1-axis represents the most variance and the PC2-axis indicates the second most variance in the data.

Computed t-statistics and p-values for the analysis of significant differentially expressed (DE) genes (S6 File). The overall picture of DE genes is visualized via the volcano plot, as depicted in Fig. S8.

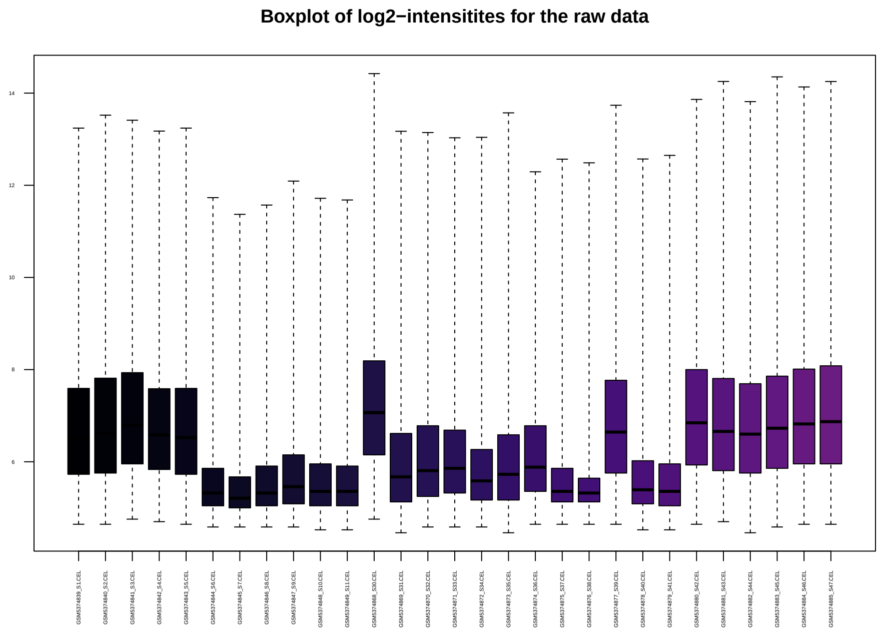

Figure S1. Box plots before normalization. Quality control depiction via box plots for the raw expression data.

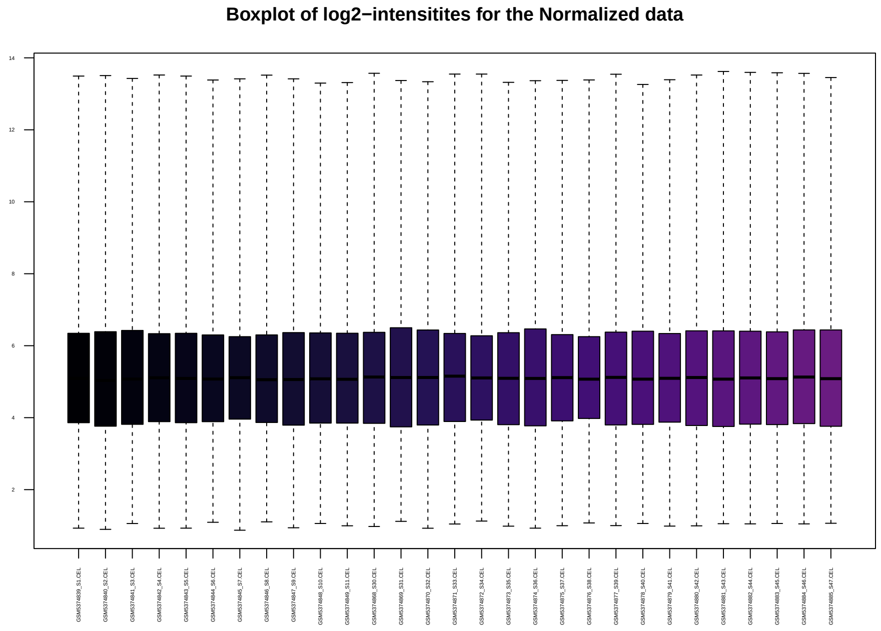

Figure S2. Box plots after normalization. Quality control depiction through box plots for the normalized expression data.

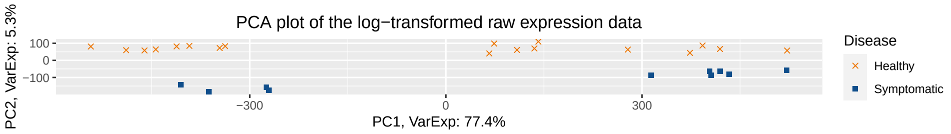

Figure S3. Principal component analysis before normalization of the expression data.

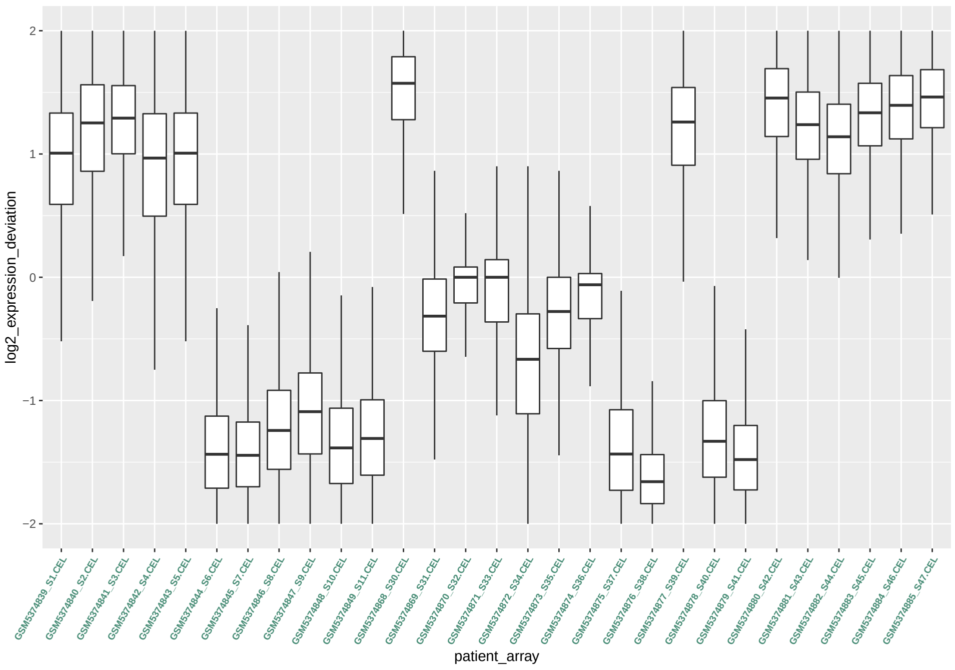

Figure S4. Relative Log Expression (RLE). Quality control analysis via RLE before normalization

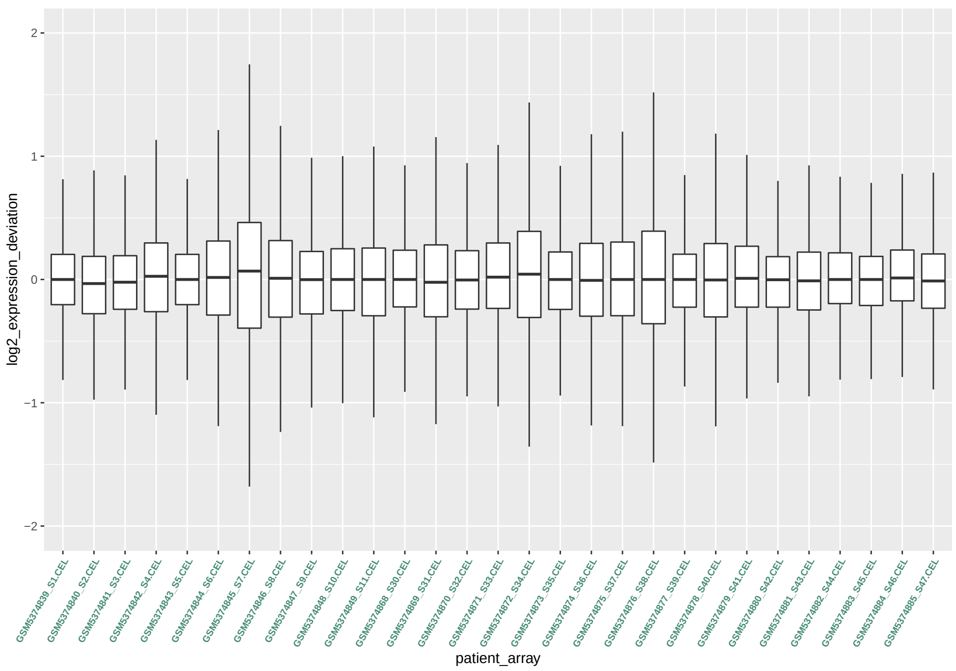

Figure S5. Relative Log Expression (RLE). Quality control analysis via RLE after normalization.

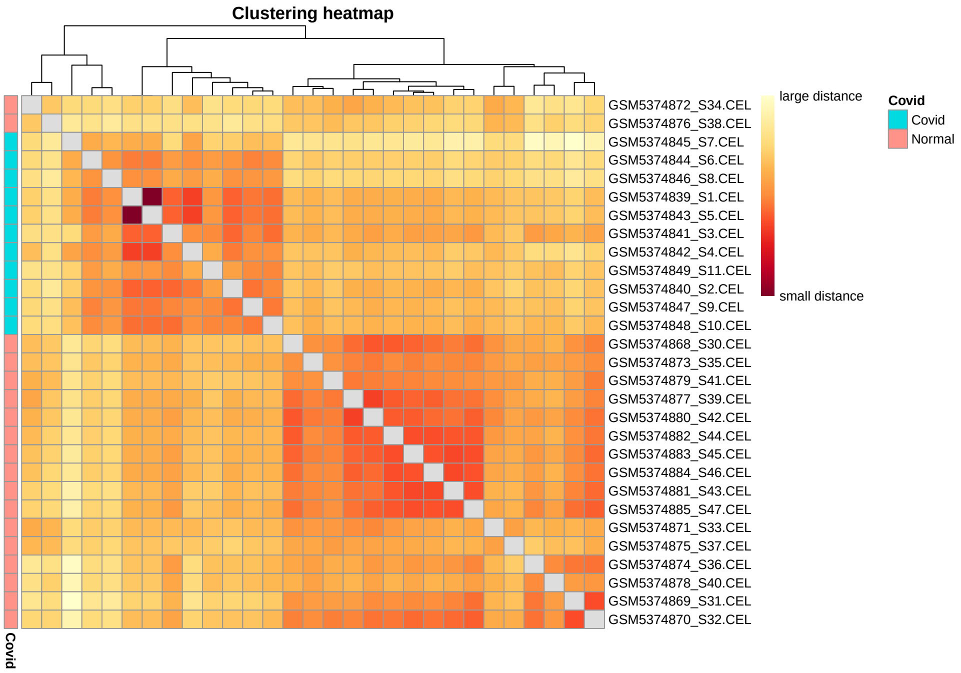

Figure S6. Heatmap. Each row in the heatmap represents a gene, and each column represents a sample or condition. The color intensity at each intersection of a row and column indicates the expression level of a gene in a particular sample. The color scale identifies genes that are upregulated (higher expression) or downregulated (lower expression) in specific conditions or samples.

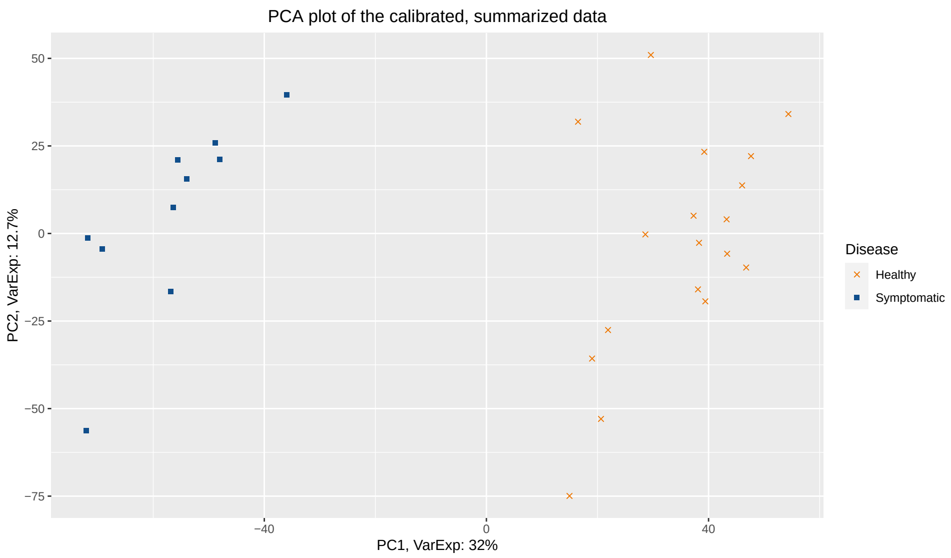

Figure S7. Principal component analysis after normalization

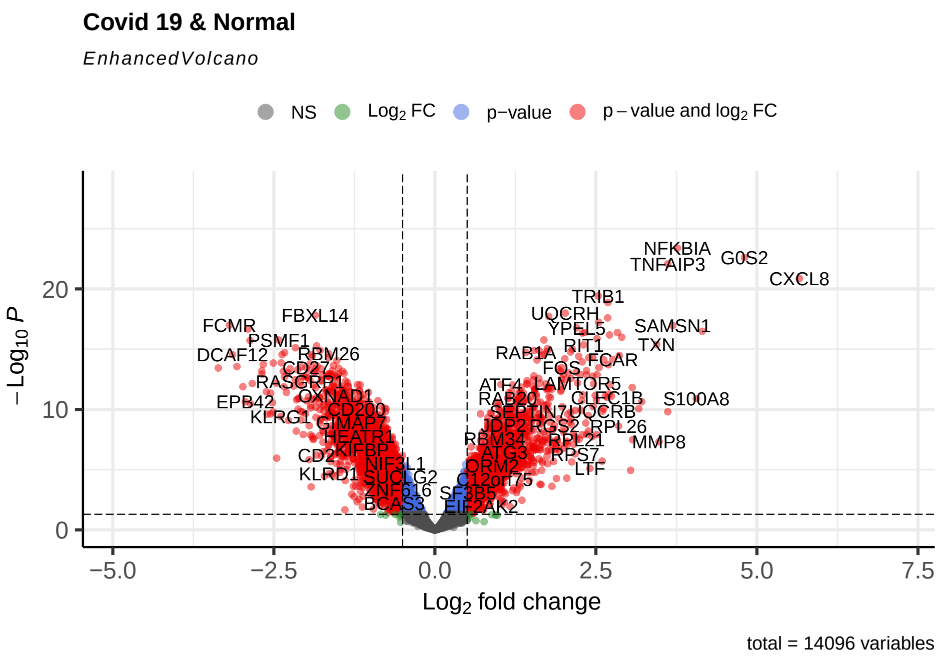

Figure S8. Volcano plot. Visualization of differentially expressed genes.
