# Supplementary material for: Unravelling the impact of SARS-CoV-2 on hemostatic and complement systems: a systems immunology perspective
Source: Front Immunol. 2025 Jan 13;15:1457324. doi: 10.3389/fimmu.2024.1457324 (PMC11781117; doi:10.3389/fimmu.2024.1457324)
Supplement: Supplementary file 8 [file DataSheet8.pdf]

Supplementary Material

Ordinary differential equations (ODEs)

```
ODE-1

d(CoV2S)/dt = 1/Plasma*(-(([k^+_CoV2SlgG]*CoV2S*lgG-
[k^-_CoV2SlgG]*[CoV2S:lgG])*Plasma) +
(([k^F10a_catCoV2S]*CoV2S*F10a/([k^F10a_mCoV2S]+CoV2S))*Plasma) +
(([k^F2a_catCoV2S]*CoV2S*F2a/([k^F2a_mCoV2S]+CoV2S))*Plasma) -
(([k^CoV2S_catgC1qR]*CoV2S*gC1qR)*Plasma) -
(([k^+_CoV2SHK]*HK*CoV2S)*Plasma))


ODE-2

d(lgG)/dt = 1/Plasma*(-(([k^+_CoV2SlgG]*CoV2S*lgG-
[k^-_CoV2SlgG]*[CoV2S:lgG])*Plasma) -
((r49.[k^+_lgGfC3b]*lgG*fC3b)*Plasma))


ODE-3

d([CoV2S:lgG])/dt = 1/Plasma*(((([k^+_CoV2SlgG]*
CoV2S*lgG-[k^-_CoV2SlgG]*[CoV2S:lgG])*Plasma) -
(([k^+_CoV2SlgGC1q]*[CoV2S:lgG]*C1q)*Plasma))


ODE-4

d(C1q)/dt = 1/Plasma*(-(([k^+_C1qrs]*[C1r:C1s]*C1q)*Plasma-
([k^-_C1qrs]*[C1q:C1r:C1s])*Plasma) +
(([k^+_CoV2SlgGC1q]*[CoV2S:lgG]*C1q)*Plasma) -
(([k^+_C1qgC1qR]*C1q*gC1qR)*Plasma-([k^-_C1qgC1qR]*[C1q:gC1qR])*Plasma)
```

```
ODE-5

d(C2)/dt = 1/Plasma*(-(([k^+_C4bC2]*C2*C4b)*Plasma-
([k^-_C4bC2]*[C4b:C2])*Plasma) -
(([k^+_fC3bC4bC2]*[fC3b:C4b]*C2)*Plasma-
([k^-_fC3bC4bC2]*[fC3b:C4b:C2])*Plasma) -
(([k^MASP2_catC2]*C2*MASP2/([k^MASP2_mC2]+C2)+
[k^C1s_catC2]*C2*C1s/([k^C1s_mC2]+C2))*Plasma))


ODE-6

d(C1INH)/dt = 1/Plasma*(-(([k^+_C1C1INH]*C1INH*C1)*Plasma) -
((r14.[k^+_C1INHMASP2]*C1INH*MASP2)*Plasma) -
((r12.[k^+_C1INHMASP1]*C1INH*MASP1)*Plasma) -
(([k^+_C1INHF12a]*C1INH*F12a)*Plasma) -
(([k^+_C1INHF2a]*C1INH*F2a)*Plasma) -
(([k^+_C1INHPn]*C1INH*Pn)*Plasma) -
(([k^+_C1INHKAL]*C1INH*KAL)*Plasma) -
(([k^+_F11aC1INH]*C1INH*F11a)*Plasma) -
(([k^+_C1INHC1s]*C1INH*C1s)*Plasma))


ODE-7

d(MBL)/dt = 1/Plasma*(-(([k^+_MBLMASP1]*MBL*MASP1)*Plasma-
([k^-_MBLMASP1]*[MBL:MASP1])*Plasma) -
(([k^+_MBLMASP2]*MBL*MASP2)*Plasma-([k^-_MBLMASP2]*[MBL:MASP2])*Plasma))


ODE-8

d(C4)/dt = 1/Plasma*(-(([k^C1_catC4]*C4*C1/([k^C1_mC4]*(1+[C4b:C2]/[k^C1_mC2]+
[fC3b:C4b:C2]/[k^C1_mC2]+C4/r35.[k^C1_mC4]))+
[k^MASP1_catC4]*[MBL:MASP1]*C4/([k^MASP1_mC4]*(1+[C4b:C2]/[k^MASP1_mC2]+
[fC3b:C4b:C2]/[k^MASP1_mC2]+C4/[k^MASP1_mC4]))+
[k^MASP2_catC4]*[MBL:MASP2]*C4/([k^MASP2_mC4]*(1+[C4b:C2]/[k^MASP2_mC2]+
[fC3b:C4b:C2]/[k^MASP2_mC2]+C4/[k^MASP2_mC4])))*Plasma) -
(([k^+_C1sC4]*C4*C1s)*Plasma-([k^-_C1sC4]*[C1s:C4])*Plasma) -
(([k^+_MASP2C4]*MASP2*C4)*Plasma-([k^-_MASP2C4]*[MASP2:C4])*Plasma))
```

ODE-9

```
d(C2a)/dt = 1/Plasma*(-((([k^+_C4bC2a]*C2a*C4b)*Plasma-
([k^-_C4bC2a]*[C4b:C2a])*Plasma) +
(([k^-_dC4bC2aC4BP]*[C4b:C2a:C4BP]+[k^-_dC4bC2aCR1]*[C4b:C2a:CR1]+
[k^-_C4bC2aC3b]*[C4b:C2a:C3b]+[k^-_dC4bC2aC3bCR1]*[C4b:C2a:C3b:CR1])*Plasma) +
(([k^MASP2_catC2]*C2*MASP2/([k^MASP2_mC2]+C2)+
[k^C1s_catC2]*C2*C1s/([k^C1s_mC2]+C2))*Plasma))
```

ODE-10

```
d(C4b)/dt = 1/Plasma*(((([k^C1_catC4]*C4*C1/([k^C1_mC4]*(1+[C4b:C2]/[k^C1_mC2]+
[fC3b:C4b:C2]/[k^C1_mC2]+C4/r35.[k^C1_mC4]))+
[k^MASP1_catC4]*[MBL:MASP1]*C4/([k^MASP1_mC4]*(1+[C4b:C2]/[k^MASP1_mC2]+
[fC3b:C4b:C2]/[k^MASP1_mC2]+C4/[k^MASP1_mC4]))+
[k^MASP2_catC4]*[MBL:MASP2]*C4/([k^MASP2_mC4]*(1+[C4b:C2]/[k^MASP2_mC2]+
[fC3b:C4b:C2]/[k^MASP2_mC2]+C4/[k^MASP2_mC4])))*Plasma) -
(([k^+_C4bC2a]*C2a*C4b)*Plasma-([k^-_C4bC2a]*[C4b:C2a])*Plasma) -
(([k^+_C4bC4BP]*C4BP*C4b)*Plasma-([k^-_C4bC4BP]*[C4b:C4BP])*Plasma) -
(([k^+_C4bC2]*C2*C4b)*Plasma-([k^-_C4bC2]*[C4b:C2])*Plasma) -
(([k^+_fC3bC4b]*fC3b*C4b)*Plasma))
```

ODE-11

```
d([C4b:C2a])/dt = 1/Plasma*(((([k^+_C4bC2a]*C2a*C4b)*Plasma-
([k^-_C4bC2a]*[C4b:C2a])*Plasma) -
(([k^+_C4bC2aCR1]*CR1*[C4b:C2a])*Plasma-
([k^-_C4bC2aCR1]*[C4b:C2a:CR1])*Plasma) -
(([k^+_C4bC2aDAF]*DAF*[C4b:C2a]-
[k^-_C4bC2aDAF]*[C4b:C2a:DAF])*Plasma) -
(([k^+_C4bC2aC3b]*C3b*[C4b:C2a])*Plasma-
([k^-_C4bC2aC3b]*[C4b:C2a:C3b])*Plasma) -
(([k^+_C4bC2aC4BP]*C4BP*[C4b:C2a])*Plasma-
([k^-_C4bC2aC4BP]*[C4b:C2a:C4BP])*Plasma) +
(([k^C1_catC2]*[C4b:C2]*C1/([k^C1_mC2]*
(1+[C4b:C2]/r102.[k^C1_mC2]+
[fC3b:C4b:C2]/r102.[k^C1_mC2]+C4/[k^C1_mC4]))+
[k^MASP1_catC2]*[MBL:MASP1]*[C4b:C2]/([k^MASP1_mC2]*
(1+[C4b:C2]/r102.[k^MASP1_mC2]+
[fC3b:C4b:C2]/r102.[k^MASP1_mC2]+C4/[k^MASP1_mC4]))+
[k^MASP2_catC2]*[MBL:MASP2]*[C4b:C2]/([k^MASP2_mC2]*
(1+[C4b:C2]/r102.[k^MASP2_mC2]+
[fC3b:C4b:C2]/[k^MASP2_mC2]+C4/r102.[k^MASP2_mC4])))*Plasma))
```

ODE-12

```
d(C4BP)/dt = 1/Plasma*(-(([k^+_C4bC4BP]*C4BP*C4b)*Plasma-
([k^-_C4bC4BP]*[C4b:C4BP])*Plasma) -
(([k^+_C4bC2aC4BP]*C4BP*[C4b:C2a])*Plasma-
([k^-_C4bC2aC4BP]*[C4b:C2a:C4BP])*Plasma))
```

ODE-13

```
d(C3)/dt = 1/Plasma*(-(([k^C3WBb_catC3]*[C3W:Bb]*
C3/([k^C3WBb_mC3]+C3)+[k^C4bC2a_catC3]*[C4b:C2a]*
C3/([k^C4bC2a_mC3]*(1+C3/[k^C4bC2a_mC3]+C5/[k^C4bC2a_mC5]))+
[k^fC3bBb_catC3]*([fC3b:Bb]+[fC3b:Bb:P])*C3/([k^fC3bBb_mC3]*
(1+C3/[k^fC3bBb_mC3]+C5/[k^fC3bBb_mC5]))
+[k^fC3bBb_catC3]*[fC3b:Bb:C3b]*C3/([k^fC3bBb_mC3]*
(1+C3/[k^fC3bBb_mC3]+C5/r122.[k^fC3bBbC3b_mC5]))+
[k^C4bC2a_catC3]*[C4b:C2a:C3b]*C3/([k^C4bC2a_mC3]*
(1+C3/[k^C4bC2a_mC3]+C5/[k^C4bC2aC3b_mC5]))+
[k^fC3bBb_catC3]*[fC3b:Bb:C3b:P]*C3/([k^fC3bBb_mC3]*
(1+C3/[k^fC3bBb_mC3]+C5/[k^fC3bBbC3b_mC5]))+
[k^KAL_catC3]*KAL*C3/([k^KAL_mC3]+C3)+[k^Pn_catC3]*
Pn*C3/([k^Pn_mC3]+C3)+
[k^F2a_catC3]*F2a*C3/([k^F2a_mC3]+C3)+[k^F10a_catC3]*
F10a*C3/([k^F10a_mC3]+C3))*Plasma) -
(([k^+_C3W]*C3)*Plasma) + (([k^+_sC3])*Plasma))
```

ODE-14

```
d(MASP1)/dt = 1/Plasma*(-(([k^+_MBLMASP1]*MBL*MASP1)*Plasma-
([k^-_MBLMASP1]*[MBL:MASP1])*Plasma) -
(([k^+_C1INHMASP1]*C1INH*MASP1)*Plasma))
```

ODE-15

```
d([MBL:MASP1])/dt = 1/Plasma*((([k^+_MBLMASP1]*MBL*MASP1)*Plasma-
([k^-_MBLMASP1]*[MBL:MASP1])*Plasma) -
(([k^+_C1INHMASP1]*C1INH*[MBL:MASP1])*Plasma))
```

ODE-16

```
d(C3W)/dt = 1/Plasma*(((r24.[k^+_C3W]*C3)*Plasma) -
(([k^+_C3WFH]*FH*C3W)*Plasma-([k^-_C3WFH]*[C3W:FH])*Plasma) -
(([k^+_C3WFB]*FB*C3W)*Plasma-([k^-_C3WFB]*[C3W:FB])*Plasma) -
(([k^-_dC3W]*C3W)*Plasma))
```

ODE-17

```
d([C3W:Bb])/dt = 1/Plasma*(-(([k^+_C3WBbFH]*FH*[C3W:Bb])*Plasma-
([k^-_C3WBbFH]*[C3W:Bb:FH])*Plasma) +
(([k^FD_catC3WFB]*[C3W:FB]*FD/([k^FD_mC3WFB]+[C3W:FB]+[fC3b:FB]+[fC3b:FB:P]+
[lgG:fC3b:FB]+[lgG:fC3b:FB:P]+[C3b:FB]+[fC3b:C4b:FB]+[fC3b:C4b:P:FB]+[fC3b:C3b:FB])+
[k^KAL_catC3WFB]*[C3W:FB]*KAL/([k^KAL_mC3WFB]+[C3W:FB]))*Plasma) -
(([k^-_C3WBb]*[C3W:Bb])*Plasma) - (([k^-_dC3WBb]*[C3W:Bb])*Plasma))
```

ODE-18

```
d(FH)/dt = 1/Plasma*(-(([k^+_C3WFH]*FH*C3W)*Plasma-
([k^-_C3WFH]*[C3W:FH])*Plasma) -
(([k^+_fC3bBbFH]*FH*[fC3b:Bb]-[k^-_fC3bBbFH]*[fC3b:Bb:FH])*Plasma) +
(([k^FI_catfC3bFH]*[fC3b:FH]*FI/([k^FI_mfC3bFH]+[fC3b:FH]+[fC3b:CR1]+
[fC3b:C4b:FH]+[fC3b:C4b:CR1]+[C3W:FH]+[C3b:FH]+[C3b:CR1])+
[k^FI_catC3bFH]*[C3b:FH]*FI/([k^FI_mC3bFH]+[C3b:FH]+[fC3b:FH]+[C3W:FH]+
[fC3b:CR1]+[fC3b:C4b:FH]+[fC3b:C4b:CR1]+[C3b:CR1])+
r65.[k^FI_catC3WFH]*[C3W:FH]*FI/([k^FI_mC3WFH]+[C3W:FH]+[fC3b:FH]+[fC3b:CR1]+
[fC3b:C4b:FH]+[fC3b:C4b:CR1]+[C3b:FH]+[C3b:CR1])+
[k^FI_catfC3bC4bFH]*[fC3b:C4b:FH]*FI/([k^FI_mfC3bC4bFH]+[fC3b:C4b:FH]+[C3W:FH]+
[fC3b:FH]+[fC3b:CR1]+[fC3b:C4b:CR1]+[C3b:FH]+[C3b:CR1]))*Plasma) -
(([k^+_fC3bBbC3bFH]*FH*[fC3b:Bb:C3b])*Plasma-
([k^-_fC3bBbC3bFH]*[fC3b:Bb:C3b:FH])*Plasma) -
(([k^+_fC3bFH]*FH*fC3b)*Plasma-([k^-_fC3bFH]*[fC3b:FH])*Plasma) -
(([k^+_C3bFH]*FH*C3b)*Plasma-([k^-_C3bFH]*[C3b:FH])*Plasma) -
(([k^+_C3WBbFH]*FH*[C3W:Bb])*Plasma-([k^-_C3WBbFH]*[C3W:Bb:FH])*Plasma) -
(([k^-_dFH]*FH)*Plasma) + (([k^+_sFH])*Plasma) -
(([k^+_fC3bC4bFH]*FH*[fC3b:C4b])*Plasma-([k^-_fC3bC4bFH]*[fC3b:C4b:FH])*Plasma) -
(([k^+_FHF2a]*FH*F2a)*Plasma) - (([k^+_FHF1a]*FH*F1a)*Plasma))
```

ODE-19

```
d(fC3b)/dt = 1/Plasma*((([k^+_fC3b]*fC3b*W)*Plasma) +
(([k^-_fC3bBb]*[fC3b:Bb])*Plasma) -
(([k^+_fC3bFB]*FB*fC3b)*Plasma-([k^-_fC3bFB]*[fC3b:FB])*Plasma) -
(([k^+_fC3bFH]*FH*fC3b)*Plasma-([k^-_fC3bFH]*[fC3b:FH])*Plasma) -
(([k^+_lgGfC3b]*lgG*fC3b)*Plasma) -
(([k^+_fC3bCR1]*CR1*fC3b)*Plasma-([k^-_fC3bCR1]*[fC3b:CR1])*Plasma) -
(([k^+_fC3bC4b]*fC3b*C4b)*Plasma) -
(([k^+_fC3bC3bFB]*[C3b:FB]*fC3b)*Plasma -
([k^-_fC3bC3bFB]*[fC3b:C3b:FB])*Plasma) -
(([k^-_dfC3b]*fC3b)*Plasma))
```

ODE-20

```
d([fC3b:Bb])/dt = 1/Plasma*(-((r94.[k^-_fC3bBb]*[fC3b:Bb])*Plasma) -
(([k^+_fC3bBbFH]*FH*[fC3b:Bb]-[k^-_fC3bBbFH]*[fC3b:Bb:FH])*Plasma) -
(([k^+_fC3bBbCR1]*CR1*[fC3b:Bb])*Plasma -
([k^-_fC3bBbCR1]*[fC3b:Bb:CR1])*Plasma) -
(([k^+_fC3bBbDAF]*DAF*[fC3b:Bb])*Plasma-
([k^-_fC3bBbDAF]*[fC3b:Bb:DAF])*Plasma) -
(([k^+_fC3bBbC3b]*C3b*[fC3b:Bb])*Plasma-
([k^-_fC3bBbC3b]*[fC3b:Bb:C3b])*Plasma) +
(([k^FD_catfC3bFB]*[fC3b:FB]*FD/([k^FD_mfC3bFB]+[fC3b:FB]+
[fC3b:FB:P]+[C3W:FB]+[lgG:fC3b:FB]+
[lgG:fC3b:FB:P]+[C3b:FB]+[fC3b:C4b:FB]+[fC3b:C4b:P:FB]+[fC3b:C3b:FB])+
[k^KAL_catfC3bFB]*[fC3b:FB]*KAL/([k^KAL_mfC3bFB]+[fC3b:FB]))*Plasma) -
(([k^+_fC3bBbP]*P*[fC3b:Bb]-r99.[k^-_fC3bBbP]*[fC3b:Bb:P])*Plasma) -
(([k^-_dfC3bBb]*[fC3b:Bb])*Plasma))
```

ODE-21

```
d(FI)/dt = 1/Plasma*(-((r131.[k^FI_catfC3bCR1]*[fC3b:CR1]*
FI/([k^FI_mfC3bCR1]+[fC3b:CR1]+
[fC3b:FH]+[C3W:FH]+[fC3b:C4b:FH]+[fC3b:C4b:CR1]+
[C3b:FH]+[C3b:CR1])+[k^FI_catC3bCR1]*[C3b:CR1]*
FI/(r131.[k^FI_mC3bCR1]+[C3b:CR1]+
[fC3b:FH]+[fC3b:CR1]+[C3W:FH]+[fC3b:C4b:FH]+
[fC3b:C4b:CR1]+[C3b:FH])+
[k^FI_catfC3bC4bCR1]*[fC3b:C4b:CR1]*FI/([k^FI_mfC3bC4bCR1]+
[fC3b:C4b:CR1]+[fC3b:FH]+[fC3b:CR1]+[C3W:FH]+[fC3b:C4b:FH]+
[C3b:FH]+[C3b:CR1]))*Plasma)
-(([k^FI_catfC3bFH]*[fC3b:FH]*FI/([k^FI_mfC3bFH]+[fC3b:FH]+
[fC3b:CR1]+[fC3b:C4b:FH]+
[fC3b:C4b:CR1]+[C3W:FH]+[C3b:FH]+[C3b:CR1])+
[k^FI_catC3bFH]*[C3b:FH]*FI/([k^FI_mC3bFH]+[C3b:FH]+
[fC3b:FH]+[C3W:FH]+[fC3b:CR1]+
[fC3b:C4b:FH]+[fC3b:C4b:CR1]+[C3b:CR1])+
[k^FI_catC3WFH]*[C3W:FH]*FI/([k^FI_mC3WFH]+[C3W:FH]+
[fC3b:FH]+[fC3b:CR1]+
[fC3b:C4b:FH]+[fC3b:C4b:CR1]+[C3b:FH]+[C3b:CR1])+
[k^FI_catfC3bC4bFH]*[fC3b:C4b:FH]*
FI/([k^FI_mfC3bC4bFH]+[fC3b:C4b:FH]+
[C3W:FH]+[fC3b:FH]+[fC3b:CR1]+[fC3b:C4b:CR1]+
[C3b:FH]+[C3b:CR1]))*Plasma))
```

```
ODE-22

d(CR1)/dt = 1/Plasma*((([k^FI_catfC3bCR1]*[fC3b:CR1]*
FI/([k^FI_mfC3bCR1]+[fC3b:CR1]+[fC3b:FH]+
[C3W:FH]+[fC3b:C4b:FH]+[fC3b:C4b:CR1]+[C3b:FH]+[C3b:CR1])+
[k^FI_catC3bCR1]*[C3b:CR1]*FI/(r131.[k^FI_mC3bCR1]+[C3b:CR1]+
[fC3b:FH]+[fC3b:CR1]+[C3W:FH]+
[fC3b:C4b:FH]+[fC3b:C4b:CR1]+[C3b:FH])+
[k^FI_catfC3bC4bCR1]*[fC3b:C4b:CR1]*FI/([k^FI_mfC3bC4bCR1]+
[fC3b:C4b:CR1]+[fC3b:FH]+
[fC3b:CR1]+[C3W:FH]+[fC3b:C4b:FH]+[C3b:FH]+[C3b:CR1]))*Plasma) -
(([k^+_C4bC2aCR1]*CR1*[C4b:C2a])*Plasma-
([k^-_C4bC2aCR1]*[C4b:C2a:CR1])*Plasma) -
(([k^+_fC3bBbCR1]*CR1*[fC3b:Bb])*Plasma-
([k^-_fC3bBbCR1]*[fC3b:Bb:CR1])*Plasma) -
(([k^+_fC3bBb3bCR1]*CR1*[fC3b:Bb:C3b])*Plasma-
([k^-_fC3bBb3bCR1]*[fC3b:Bb:C3b:CR1])*Plasma) -
(([k^+_C4b2a3bCR1]*CR1*[C4b:C2a:C3b])*Plasma-
([k^-_C4b2a3bCR1]*[C4b:C2a:C3b:CR1])*Plasma) -
(([k^+_C3bCR1]*CR1*C3b)*Plasma-([k^-_C3bCR1]*[C3b:CR1])*Plasma) -
(([k^+_fC3bCR1]*CR1*fC3b)*Plasma-([k^-_fC3bCR1]*[fC3b:CR1])*Plasma) -
(([k^+_fC3bC4bCR1]*[fC3b:C4b]*CR1)*Plasma-
([k^-_fC3bC4bCR1]*[fC3b:C4b:CR1])*Plasma))


ODE-23

d(DAF)/dt = 1/Plasma*(-(([k^+_C4bC2aDAF]*DAF*[C4b:C2a]-
[k^-_C4bC2aDAF]*[C4b:C2a:DAF])*Plasma) -
(([k^+_fC3bBbDAF]*DAF*[fC3b:Bb])*Plasma-
([k^-_fC3bBbDAF]*[fC3b:Bb:DAF])*Plasma) -
(([k^+_fC3bBbC3bDAF]*DAF*[fC3b:Bb:C3b])*Plasma-
([k^-_fC3bBbC3bDAF]*[fC3b:Bb:C3b:DAF])*Plasma) -
(([k^+_C4bC2aC3bDAF]*DAF*[C4b:C2a:C3b]-
[k^-_C4bC2aC3bDAF]*[C4b:C2a:C3b:DAF])*Plasma))
```

ODE-24

```
d(C3b)/dt = 1/Plasma*((([k^C3WBb_catC3]*[C3W:Bb]*C3/([k^C3WBb_mC3]+C3)+
[k^C4bC2a_catC3]*[C4b:C2a]*C3/([k^C4bC2a_mC3]*
(1+C3/[k^C4bC2a_mC3]+C5/[k^C4bC2a_mC5]))+
[k^fC3bBb_catC3]*([fC3b:Bb]+[fC3b:Bb:P])*C3/([k^fC3bBb_mC3]*
(1+C3/[k^fC3bBb_mC3]+C5/[k^fC3bBb_mC5]))+
[k^fC3bBb_catC3]*[fC3b:Bb:C3b]*C3/(r122.[k^fC3bBb_mC3]*
(1+C3/[k^fC3bBb_mC3]+C5/r122.[k^fC3bBbC3b_mC5]))+
[k^C4bC2a_catC3]*[C4b:C2a:C3b]*C3/([k^C4bC2a_mC3]*
(1+C3/[k^C4bC2a_mC3]+C5/[k^C4bC2aC3b_mC5]))+
[k^fC3bBb_catC3]*[fC3b:Bb:C3b:P]*C3/(r122.[k^fC3bBb_mC3]*
(1+C3/r122.[k^fC3bBb_mC3]+C5/[k^fC3bBbC3b_mC5]))+
[k^KAL_catC3]*KAL*C3/([k^KAL_mC3]+C3)+[k^Pn_catC3]*
Pn*C3/([k^Pn_mC3]+C3)+[k^F2a_catC3]*F2a*C3/([k^F2a_mC3]+C3)+
[k^F10a_catC3]*F10a*C3/([k^F10a_mC3]+C3))*Plasma) -
(([k^+_fC3bBbC3b]*C3b*[fC3b:Bb])*Plasma-([k^-_fC3bBbC3b]*[fC3b:Bb:C3b])*Plasma) -
(([k^+_C4bC2aC3b]*C3b*[C4b:C2a])*Plasma-([k^-_C4bC2aC3b]*[C4b:C2a:C3b])*Plasma) -
(([k^+_C3bFH]*FH*C3b)*Plasma-([k^-_C3bFH]*[C3b:FH])*Plasma) -
(([k^+_C3bCR1]*CR1*C3b)*Plasma-([k^-_C3bCR1]*[C3b:CR1])*Plasma) -
(([k^+_C3bP]*P*C3b)*Plasma-([k^-_C3bP]*[C3b:P])*Plasma) -
(([k^+_C3bFB]*FB*C3b)*Plasma-([k^-_C3bFB]*[C3b:FB])*Plasma) -
(([k^-_dC3b]*C3b)*Plasma))
```

ODE-25

```
d([fC3b:Bb:C3b])/dt = 1/Plasma*((([k^+_fC3bBbC3b]*C3b*[fC3b:Bb])*Plasma-
([k^-_fC3bBbC3b]*[fC3b:Bb:C3b])*Plasma) -
(([k^+_fC3bBb3bCR1]*CR1*[fC3b:Bb:C3b])*Plasma-
([k^-_fC3bBb3bCR1]*[fC3b:Bb:C3b:CR1])*Plasma) -
(([k^+_fC3bBbC3bDAF]*DAF*[fC3b:Bb:C3b])*Plasma-
([k^-_fC3bBbC3bDAF]*[fC3b:Bb:C3b:DAF])*Plasma) -
(([k^+_fC3bBbC3bFH]*FH*[fC3b:Bb:C3b])*Plasma-
([k^-_fC3bBbC3bFH]*[fC3b:Bb:C3b:FH])*Plasma) -
(([k^+_fC3bBbC3bP]*P*[fC3b:Bb:C3b])*Plasma-
([k^-_fC3bBbC3bP]*[fC3b:Bb:C3b:P])*Plasma) +
(([k^FD_catfC3bC3bFB]*[fC3b:C3b:FB]*FD/([k^FD_mfC3bC3bFB]+
[fC3b:C3b:FB]+[C3W:FB]+[fC3b:FB]+
[C3b:FB]+[fC3b:FB:P]+[fC3b:C4b:FB]+[lgG:fC3b:FB]+
[lgG:fC3b:FB:P]+[fC3b:C4b:P:FB]))*Plasma))
```

ODE-26

```
d([C4b:C2a:C3b])/dt = 1/Plasma*((([k^+_C4bC2aC3b]*C3b*[C4b:C2a])*Plasma-
(r132.[k^-_C4bC2aC3b]*[C4b:C2a:C3b])*Plasma) -
(([k^+_C4b2a3bCR1]*CR1*[C4b:C2a:C3b])*Plasma-
([k^-_C4b2a3bCR1]*[C4b:C2a:C3b:CR1])*Plasma) -
(([k^+_C4bC2aC3bDAF]*DAF*[C4b:C2a:C3b]-
[k^-_C4bC2aC3bDAF]*[C4b:C2a:C3b:DAF])*Plasma) +
((r107.[k^C1_catC2]*[fC3b:C4b:C2]*C2/(r107.[k^C1_mC2]*
(1+[C4b:C2]/r107.[k^C1_mC2]+
[fC3b:C4b:C2]/r107.[k^C1_mC2]+C4/r107.[k^C1_mC4]))+
r107.[k^MASP1_catC2]*[MBL:MASP1]*[fC3b:C4b:C2]/(r107.[k^MASP1_mC2]*
(1+[C4b:C2]/r107.[k^MASP1_mC2]+
[fC3b:C4b:C2]/r107.[k^MASP1_mC2]+C4/r107.[k^MASP1_mC4]))+
r107.[k^MASP2_catC2]*[MBL:MASP2]*[fC3b:C4b:C2]/(r107.[k^MASP2_mC2]*
(1+[C4b:C2]/r107.[k^MASP2_mC2]+
[fC3b:C4b:C2]/r107.[k^MASP2_mC2]+C4/r107.[k^MASP2_mC4])))*Plasma))
```

ODE-27

```
d(C2b)/dt = 1/Plasma*(((r76.[k^C1_catC2]*[C4b:C2]*C1/(r76.[k^C1_mC2]*
(1+[C4b:C2]/r76.[k^C1_mC2]+
[fC3b:C4b:C2]/r76.[k^C1_mC2]+C4/r76.[k^C1_mC4]))+
r76.[k^C1_catC2]*[fC3b:C4b:C2]*C1/(r76.[k^C1_mC2]*
(1+[C4b:C2]/r76.[k^C1_mC2]+
[fC3b:C4b:C2]/r76.[k^C1_mC2]+C4/r76.[k^C1_mC4]))+
r76.[k^MASP1_catC2]*[MBL:MASP1]*[C4b:C2]/(r76.[k^MASP1_mC2]*
(1+[C4b:C2]/r76.[k^MASP1_mC2]+
[fC3b:C4b:C2]/r76.[k^MASP1_mC2]+C4/r76.[k^MASP1_mC4]))+
r76.[k^MASP2_catC2]*[MBL:MASP2]*[C4b:C2]/(r76.[k^MASP2_mC2]*
(1+[C4b:C2]/r76.[k^MASP2_mC2]+
[fC3b:C4b:C2]/r76.[k^MASP2_mC2]+C4/r76.[k^MASP2_mC4]))+
r76.[k^MASP1_catC2]*[MBL:MASP1]*[fC3b:C4b:C2]/(r76.[k^MASP1_mC2]*
(1+[C4b:C2]/r76.[k^MASP1_mC2]+
[fC3b:C4b:C2]/r76.[k^MASP1_mC2]+C4/r76.[k^MASP1_mC4]))+
r76.[k^MASP2_catC2]*[MBL:MASP2]*[fC3b:C4b:C2]/(r76.[k^MASP2_mC2]*
(1+[C4b:C2]/r76.[k^MASP2_mC2]+
[fC3b:C4b:C2]/r76.[k^MASP2_mC2]+C4/r76.[k^MASP2_mC4])))*Plasma) +
((r75.[k^MASP2_catC2]*C2*MASP2/(r75.[k^MASP2_mC2]+C2)+[k^C1s_catC2]*
C2*C1s/([k^C1s_mC2]+C2))*Plasma))
```

ODE-28

```
d(C4a)/dt = 1/Plasma*((([k^C1_catC4]*C4*C1/(r35.[k^C1_mC4]*(1+[C4b:C2]/r35.[k^C1_mC2]+
[fC3b:C4b:C2]/r35.[k^C1_mC2]+C4/r35.[k^C1_mC4]))+
[k^MASP1_catC4]*[MBL:MASP1]*C4/(r35.[k^MASP1_mC4]*(1+[C4b:C2]/r35.[k^MASP1_mC2]+
[fC3b:C4b:C2]/r35.[k^MASP1_mC2]+C4/r35.[k^MASP1_mC4]))+
[k^MASP2_catC4]*[MBL:MASP2]*C4/(r35.[k^MASP2_mC4]*(1+[C4b:C2]/r35.[k^MASP2_mC2]+
[fC3b:C4b:C2]/r35.[k^MASP2_mC2]+C4/r35.[k^MASP2_mC4])))*Plasma))
```

ODE-29

```
d([C4b:C4BP])/dt = 1/Plasma*((([k^+_C4bC4BP]*C4BP*C4b)*Plasma-
([k^-_C4bC4BP]*[C4b:C4BP])*Plasma))
```

ODE-30

```
d([C4b:C2a:C4BP])/dt = 1/Plasma*((([k^+_C4bC2aC4BP]*C4BP*[C4b:C2a])*Plasma-
([k^-_C4bC2aC4BP]*[C4b:C2a:C4BP])*Plasma) -
((r88.[k^-_dC4bC2aC4BP]*[C4b:C2a:C4BP])*Plasma))
```

ODE-31

```
d(C3a)/dt = 1/Plasma*((([k^C3WBb_catC3]*[C3W:Bb]*
C3/([k^C3WBb_mC3]+C3)+[k^C4bC2a_catC3]*[C4b:C2a]*
C3/([k^C4bC2a_mC3]*(1+C3/[k^C4bC2a_mC3]+C5/[k^C4bC2a_mC5]))+
[k^fC3bBb_catC3]*([fC3b:Bb]+[fC3b:Bb:P])*C3/(r122.[k^fC3bBb_mC3]*
(1+C3/r122.[k^fC3bBb_mC3]+C5/r122.[k^fC3bBb_mC5]))+[k^fC3bBb_catC3]*
[fC3b:Bb:C3b]*C3/(r122.[k^fC3bBb_mC3]*(1+C3/r122.[k^fC3bBb_mC3]+
C5/r122.[k^fC3bBbC3b_mC5]))+[k^C4bC2a_catC3]*[C4b:C2a:C3b]*
C3/([k^C4bC2a_mC3]*(1+C3/[k^C4bC2a_mC3]+
C5/[k^C4bC2aC3b_mC5]))+[k^fC3bBb_catC3]*[fC3b:Bb:C3b:P]*
C3/(r122.[k^fC3bBb_mC3]*(1+C3/r122.[k^fC3bBb_mC3]+
C5/r122.[k^fC3bBbC3b_mC5]))+[k^KAL_catC3]*
KAL*C3/([k^KAL_mC3]+C3)+[k^Pn_catC3]*Pn*C3/([k^Pn_mC3]+C3)+
[k^F2a_catC3]*F2a*C3/([k^F2a_mC3]+C3)+[k^F10a_catC3]*
F10a*C3/([k^F10a_mC3]+C3))*Plasma) -
(([k^+_C3aC3aR1]*C3a*C3aR1)*Plasma-
([k^-_C3aC3aR1]*[C3a:C3aR1])*Plasma) -
(([k^C3a_catTF]*C3a)*Plasma) -
(([k^+_C3aTAFIa]*TAFIa*C3a)*Plasma) -
(([k^-_dC3a]*C3a)*Plasma))
```

ODE-32

```
d([fC3b:Bb:CR1])/dt = 1/Plasma*((([k^+_fC3bBbCR1]*CR1*[fC3b:Bb])*Plasma-
([k^-_fC3bBbCR1]*[fC3b:Bb:CR1])*Plasma) -
((r108.[k^-_dfC3bBbCR1]*[fC3b:Bb:CR1])*Plasma))
```

ODE-33

```
d([fC3b:Bb:DAF])/dt = 1/Plasma*((([k^+_fC3bBbDAF]*DAF*[fC3b:Bb])*Plasma-
([k^-_fC3bBbDAF]*[fC3b:Bb:DAF])*Plasma) -
((r120.[k^-_dfC3bBbDAF]*[fC3b:Bb:DAF])*Plasma))
```

ODE-34

```
d([C4b:C2a:CR1])/dt = 1/Plasma*((([k^+_C4bC2aCR1]*CR1*[C4b:C2a])*Plasma-
([k^-_C4bC2aCR1]*[C4b:C2a:CR1])*Plasma) -
((r106.[k^-_dC4bC2aCR1]*[C4b:C2a:CR1])*Plasma))
```

ODE-35

```
d([C4b:C2a:DAF])/dt = 1/Plasma*((([k^+_C4bC2aDAF]*DAF*[C4b:C2a]-
[k^-_C4bC2aDAF]*[C4b:C2a:DAF])*Plasma) -
((r104.[k^-_dC4bC2aDAF]*[C4b:C2a:DAF])*Plasma))
```

ODE-36

```
d([fC3b:Bb:FH])/dt = 1/Plasma*((([k^+_fC3bBbFH]*FH*[fC3b:Bb]-
[k^-_fC3bBbFH]*[fC3b:Bb:FH])*Plasma) -
((r84.[k^-_dfC3bBbFH]*[fC3b:Bb:FH])*Plasma))
```

ODE-37

```
d([fC3b:Bb:C3b:CR1])/dt = 1/Plasma*((([k^+_fC3bBb3bCR1]*CR1*[fC3b:Bb:C3b])*Plasma-
([k^-_fC3bBb3bCR1]*[fC3b:Bb:C3b:CR1])*Plasma) -
((r114.[k^-_dfC3bBbC3bCR1]*[fC3b:Bb:C3b:CR1])*Plasma))
```

ODE-38

```
d([fC3b:Bb:C3b:DAF])/dt = 1/Plasma*((([k^+_fC3bBbC3bDAF]*DAF*[fC3b:Bb:C3b])*Plasma-
([k^-_fC3bBbC3bDAF]*[fC3b:Bb:C3b:DAF])*Plasma) -
((r136.[k^-_dfC3bBbC3bDAF]*[fC3b:Bb:C3b:DAF])*Plasma))
```

ODE-39

d([fC3b:Bb:C3b:FH])/dt = 1/Plasma*((([k^+_fC3bBbC3bFH]*FH*[fC3b:Bb:C3b])*Plasma-([k^-_fC3bBbC3bFH]*[fC3b:Bb:C3b:FH])*Plasma) - ((r116.[k^-_dfC3bBbC3bFH]*[fC3b:Bb:C3b:FH])*Plasma))

ODE-40

d([C4b:C2a:C3b:CR1])/dt = 1/Plasma*((([k^+_C4b2a3bCR1]*CR1*[C4b:C2a:C3b])*Plasma-([k^-_C4b2a3bCR1]*[C4b:C2a:C3b:CR1])*Plasma) - ((r134.[k^-_dC4bC2aC3bCR1]*[C4b:C2a:C3b:CR1])*Plasma))

ODE-41

d([C4b:C2a:C3b:DAF])/dt = 1/Plasma*((([k^+_C4bC2aC3bDAF]*DAF*[C4b:C2a:C3b]-[k^-_C4bC2aC3bDAF]*[C4b:C2a:C3b:DAF])*Plasma) - (([k^-_dC4b2a3bDAF]*[C4b:C2a:C3b:DAF])*Plasma))

ODE-42

d(FB)/dt = 1/Plasma*(-(([k^+_C3WFB]*FB*C3W)*Plasma- ([k^-_C3WFB]*[C3W:FB])*Plasma) - (([k^+_fC3bFB]*FB*fC3b)*Plasma-([k^-_fC3bFB]*[fC3b:FB])*Plasma) + (([k^+_sFB])*Plasma) - (([k^+_lgGfC3bFB]*[lgG:fC3b]*FB)*Plasma-([k^-_lgGfC3bFB]*[lgG:fC3b:FB])*Plasma) - (([k^+_C3bFB]*FB*C3b)*Plasma-([k^-_C3bFB]*[C3b:FB])*Plasma) - (([k^+_fC3bC4bFB]*FB*[fC3b:C4b]-[k^-_fC3bC4bFB]*[fC3b:C4b:FB])*Plasma) - (([k^+_fC3bC4bPFB]*FB*[fC3b:C4b:P])*Plasma-([k^-_fC3bC4bPFB]*[fC3b:C4b:P:FB])*Plasma) - (([k^-_dFB]*FB)*Plasma))

ODE-43

d([C3W:FB])/dt = 1/Plasma*((([k^+_C3WFB]*FB*C3W)*Plasma-([k^-_C3WFB]*[C3W:FB])*Plasma) - ((r26.[k^FD_catC3WFB]*[C3W:FB]*FD/(r26.[k^FD_mC3WFB]+[C3W:FB]+[fC3b:FB]+[fC3b:FB:P]+[lgG:fC3b:FB]+[lgG:fC3b:FB:P]+[C3b:FB]+[fC3b:C4b:FB]+[fC3b:C4b:P:FB]+[fC3b:C3b:FB])+[k^KAL_catC3WFB]*[C3W:FB]*KAL/([k^KAL_mC3WFB]+[C3W:FB]))*Plasma) - (([k^-_dC3WFB]*[C3W:FB])*Plasma))

ODE-44

d(FD)/dt = 1/Plasma*((([k^-_sFD])*Plasma) - (([k^-_dFD]*FD)*Plasma) + (([k^MASP1_catPFD]*PFD*MASP1+[k^MASP2_catPFD]*PFD*MASP2+[k^F2a_catPFD]*PFD*F2a)*Plasma))

ODE-45

```
d([fC3b:FB])/dt = 1/Plasma*((([k^+_fC3bFB]*FB*fC3b)*Plasma-([k^-_fC3bFB]*[fC3b:FB])*Plasma) -
(([k^FD_catfC3bFB]*[fC3b:FB]*FD/([k^FD_mfC3bFB]+[fC3b:FB]+[fC3b:FB:P]+[C3W:FB]+[1gG:fC3b:FB]+
[1gG:fC3b:FB:P]+[C3b:FB]+[fC3b:C4b:FB]+[fC3b:C4b:P:FB]+[fC3b:C3b:FB])+
[k^KAL_catfC3bFB]*[fC3b:FB]*KAL/([k^KAL_mfC3bFB]+[fC3b:FB]))*Plasma) -
(([k^+_fC3bFBP]*P*[fC3b:FB])*Plasma-([k^-_fC3bFBP]*[fC3b:FB:P])*Plasma) -
(([k^-_dfC3bFB]*[fC3b:FB])*Plasma))
```

ODE-46

```
d([fC3b:FH])/dt = 1/Plasma*((([k^+_fC3bFH]*FH*fC3b)*Plasma-([k^-_fC3bFH]*[fC3b:FH])*Plasma) -
((r38.[k^FI_catfC3bFH]*[fC3b:FH]*FI/(r38.[k^FI_mfC3bFH]+[fC3b:FH]+[C3b:FH]+[C3b:CR1]+[fC3b:CR1]+
[C3W:FH]+[fC3b:C4b:FH]+[fC3b:C4b:CR1]))*Plasma))
```

ODE-47

```
d([C3b:FH])/dt = 1/Plasma*((([k^+_C3bFH]*FH*C3b)*Plasma-([k^-_C3bFH]*[C3b:FH])*Plasma) -
((r127.[k^FI_catC3bFH]*[C3b:FH]*FI/(r127.[k^FI_mC3bFH]+[C3b:FH]+[fC3b:FH]+[C3b:CR1]+[fC3b:CR1]+
[C3W:FH]+[fC3b:C4b:FH]+[fC3b:C4b:CR1]))*Plasma))
```

ODE-48

```
d([C3b:CR1])/dt = 1/Plasma*((([k^+_C3bCR1]*CR1*C3b)*Plasma-([k^-_C3bCR1]*[C3b:CR1])*Plasma) -
((r129.[k^FI_catC3bCR1]*[C3b:CR1]*FI/(r129.[k^FI_mC3bCR1]+[C3b:CR1]+[fC3b:CR1]+[C3b:FH]+[fC3b:FH]+
[C3W:FH]+[fC3b:C4b:FH]+[fC3b:C4b:CR1]))*Plasma))
```

ODE-49

```
d([C3W:FH])/dt = 1/Plasma*((([k^+_C3WFH]*FH*C3W)*Plasma-([k^-_C3WFH]*[C3W:FH])*Plasma) -
((r68.[k^FI_catC3WFH]*[C3W:FH]*FI/(r68.[k^FI_mC3WFH]+[C3W:FH]+[fC3b:FH]+[fC3b:CR1]+[fC3b:C4b:FH]+
[fC3b:C4b:CR1]+[C3b:FH]+[C3b:CR1]))*Plasma))
```

ODE-50

```
d(C3aR1)/dt = 1/Plasma*(-(([k^+_C3aC3aR1]*C3a*C3aR1)*Plasma-
([k^-_C3aC3aR1]*[C3a:C3aR1])*Plasma))
```

ODE-51

```
d([C3a:C3aR1])/dt = 1/Plasma*((([k^+_C3aC3aR1]*C3a*C3aR1)*Plasma-
([k^-_C3aC3aR1]*[C3a:C3aR1])*Plasma) -
(([k^C3aC3aR1_catIL6]*[C3a:C3aR1])*Plasma))
```

ODE-52

```
d(C5)/dt = 1/Plasma*(-((([k^fC3bBb_catC5]*[fC3b:Bb]*
C5/(r140.[k^fC3bBb_mC5]*(1+C3/r140.[k^fC3bBb_mC3]+
C5/r140.[k^fC3bBb_mC5]))+[k^fC3bBb_catC5]*[fC3b:Bb:P]*
C5/(r140.[k^fC3bBb_mC5]*(1+C3/r140.[k^fC3bBb_mC3]+
C5/r140.[k^fC3bBb_mC5]))+[k^C4b2a_catC5]*[C4b:C2a]*
C5/([k^C4b2a_mC5]*(1+C3/[k^C4b2a_mC3]+C5/[k^C4b2a_mC5]))+
[k^C4b2a3b_catC5]*[C4b:C2a:C3b]*C5/([k^C4b2a3b_mC5]*
(1+C3/[k^C4b2a_mC3]+C5/[k^C4b2a3b_mC5]))+
[k^fC3bBbC3b_catC5]*([fC3b:Bb:C3b]+[fC3b:Bb:C3b:P])*
C5/(r140.[k^fC3bBbC3b_mC5]*(1+C3/r140.[k^fC3bBb_mC3]+
C5/r140.[k^fC3bBbC3b_mC5]))+[k^KAL_catC5]*
KAL*C5/([k^KAL_mC5]+C5)+[k^Pn_catC5]*Pn*C5/([k^Pn_mC5]+C5)+
[k^F2a_catC5]*F2a*C5/([k^F2a_mC5]+C5)+[k^F10a_catC5]*
F10a*C5/([k^F10a_mC5]+C5))*Plasma) -
(([k^-_dC5]*C5)*Plasma) + (([k^+_sC5])*Plasma))
```

ODE-53

```
d(C5b)/dt = 1/Plasma*((([k^fC3bBb_catC5]*[fC3b:Bb]*
C5/(r140.[k^fC3bBb_mC5]*(1+C3/r140.[k^fC3bBb_mC3]+
C5/r140.[k^fC3bBb_mC5]))+[k^fC3bBb_catC5]*[fC3b:Bb:P]*
C5/(r140.[k^fC3bBb_mC5]*(1+C3/r140.[k^fC3bBb_mC3]+
C5/r140.[k^fC3bBb_mC5]))+[k^C4b2a_catC5]*[C4b:C2a]*
C5/([k^C4b2a_mC5]*(1+C3/[k^C4b2a_mC3]+C5/[k^C4b2a_mC5]))+
[k^C4b2a3b_catC5]*[C4b:C2a:C3b]*C5/([k^C4b2a3b_mC5]*
(1+C3/[k^C4b2a_mC3]+C5/[k^C4b2a3b_mC5]))+
[k^fC3bBbC3b_catC5]*([fC3b:Bb:C3b]+[fC3b:Bb:C3b:P])*
C5/(r140.[k^fC3bBbC3b_mC5]*(1+C3/r140.[k^fC3bBb_mC3]+
C5/r140.[k^fC3bBbC3b_mC5]))+[k^KAL_catC5]*
KAL*C5/([k^KAL_mC5]+C5)+[k^Pn_catC5]*Pn*C5/([k^Pn_mC5]+C5)+
[k^F2a_catC5]*F2a*C5/([k^F2a_mC5]+C5)+[k^F10a_catC5]*
F10a*C5/([k^F10a_mC5]+C5))*Plasma) -
(([k^+_C5bC6]*C5b*C6-[k^-_C5bC6]*[C5b:C6])*Plasma) -
(([k^-_dC5b]*C5b)*Plasma))
```

ODE-54

```
d(C5a)/dt = 1/Plasma*((([k^fC3bBb_catC5]*[fC3b:Bb]*
C5/(r140.[k^fC3bBb_mC5]*(1+C3/r140.[k^fC3bBb_mC3]+
C5/r140.[k^fC3bBb_mC5]))+[k^fC3bBb_catC5]*[fC3b:Bb:P]*
C5/(r140.[k^fC3bBb_mC5]*(1+C3/r140.[k^fC3bBb_mC3]+
C5/r140.[k^fC3bBb_mC5]))+[k^C4b2a_catC5]*[C4b:C2a]*
C5/([k^C4b2a_mC5]*(1+C3/[k^C4b2a_mC3]+C5/[k^C4b2a_mC5]))+
[k^C4b2a3b_catC5]*[C4b:C2a:C3b]*C5/([k^C4b2a3b_mC5]*
(1+C3/[k^C4b2a_mC3]+C5/[k^C4b2a3b_mC5]))+
[k^fC3bBbC3b_catC5]*([fC3b:Bb:C3b]+[fC3b:Bb:C3b:P])*
C5/(r140.[k^fC3bBbC3b_mC5]*(1+C3/r140.[k^fC3bBb_mC3]+
C5/r140.[k^fC3bBbC3b_mC5]))+[k^KAL_catC5]*
KAL*C5/([k^KAL_mC5]+C5)+[k^Pn_catC5]*Pn*C5/([k^Pn_mC5]+C5)+
[k^F2a_catC5]*F2a*C5/([k^F2a_mC5]+C5)+[k^F10a_catC5]*
F10a*C5/([k^F10a_mC5]+C5))*Plasma) -
(([k^+_C5aC5aR1]*C5a*C5aR1)*Plasma-
([k^-_C5aC5aR1]*[C5a:C5aR1])*Plasma) -
(([k^C5a_catTF]*C5a)*Plasma) -
(([k^+_C5aTAFIa]*TAFIa*C5a)*Plasma) -
(([k^-_dC5a]*C5a)*Plasma))
```

ODE-55

```
d(C6)/dt = 1/Plasma*(-(([k^+_C5bC6]*C5b*C6-[k^-_C5bC6]*[C5b:C6])*Plasma) -
(([k^-_dC6]*C6)*Plasma)
+ (([k^+_sC6])*Plasma))
```

ODE-56

```
d([C5b:C6])/dt = 1/Plasma*(((([k^+_C5bC6]*C5b*C6-[k^-_C5bC6]*[C5b:C6])*Plasma) -
(([k^+_C5bC6C7]*C7*[C5b:C6])*Plasma-([k^-_C5bC6C7]*[C5b:C6:C7])*Plasma))
```

ODE-57

```
d(C7)/dt = 1/Plasma*(-(([k^+_C5bC6C7]*C7*[C5b:C6])*Plasma-
([k^-_C5bC6C7]*[C5b:C6:C7])*Plasma) +
(([k^+_sC7])*Plasma) - (([k^-_dC7]*C7)*Plasma))
```

ODE-58

d([C5b:C6:C7])/dt = 1/Plasma*((([k^+_C5bC6C7]*C7*[C5b:C6])*Plasma-([k^-_C5bC6C7]*[C5b:C6:C7])*Plasma) - (([k^+_C5bC6C7C8]*C8*[C5b:C6:C7])*Plasma-([k^-_C5bC6C7C8]*[C5b:C6:C7:C8])*Plasma))

ODE-59

d(C8)/dt = 1/Plasma*(-(([k^+_C5bC6C7C8]*C8*[C5b:C6:C7])*Plasma-([k^-_C5bC6C7C8]*[C5b:C6:C7:C8])*Plasma) - (([k^-_dC8]*C8)*Plasma) + (([k^+_sC8])*Plasma))

ODE-60

d([C5b:C6:C7:C8])/dt = 1/Plasma*((([k^+_C5bC6C7C8]*C8*[C5b:C6:C7])*Plasma-([k^-_C5bC6C7C8]*[C5b:C6:C7:C8])*Plasma) - (([k^+_C5bC6C7C8C9]*C9*[C5b:C6:C7:C8])*Plasma-([k^-_C5bC6C7C8C9]*[C5b:C6:C7:C8:C9])*Plasma))

ODE-61

d(C9)/dt = 1/Plasma*(-(([k^+_C5bC6C7C8C9]*C9*[C5b:C6:C7:C8])*Plasma-([k^-_C5bC6C7C8C9]*[C5b:C6:C7:C8:C9])*Plasma) - (([k^-_dC9]*C9)*Plasma) + (([k^+_sC9])*Plasma))

ODE-62

d([C5b:C6:C7:C8:C9])/dt = 1/Plasma*((([k^+_C5bC6C7C8C9]*C9*[C5b:C6:C7:C8])*Plasma-([k^-_C5bC6C7C8C9]*[C5b:C6:C7:C8:C9])*Plasma) - (([k^MAC_catF2]*[C5b:C6:C7:C8:C9])*Plasma) - (([k^+_CD59MAC]*CD59*[C5b:C6:C7:C8:C9])*Plasma))

ODE-63

d(C5aR1)/dt = 1/Plasma*(-(([k^+_C5aC5aR1]*C5a*C5aR1)*Plasma-([k^-_C5aC5aR1]*[C5a:C5aR1])*Plasma))

ODE-64

d([C5a:C5aR1])/dt = 1/Plasma*((([k^+_C5aC5aR1]*C5a*C5aR1)*Plasma-([k^-_C5aC5aR1]*[C5a:C5aR1])*Plasma) - (([k^C5aC5aR1_catIL6]*[C5a:C5aR1])*Plasma))

ODE-65

```
d([lgG:fC3b])/dt = 1/Plasma*(((r49.[k^+_lgGfC3b]*lgG*fC3b)*Plasma) -
(([k^+_lgGfC3bFB]*[lgG:fC3b]*FB)*Plasma-([k^-_lgGfC3bFB]*[lgG:fC3b:FB])*Plasma) +
((r50.[k^+_lgGfC3b]*[lgG:fC3b]*W)*Plasma))
```

ODE-66

```
d([C4b:C2])/dt = 1/Plasma*((([k^+_C4bC2]*C2*C4b)*Plasma-
([k^-_C4bC2]*[C4b:C2])*Plasma) -
((r74.[k^C1_catC2]*[C4b:C2]*C1/(r74.[k^C1_mC2]*
(1+[C4b:C2]/r74.[k^C1_mC2]+
[fC3b:C4b:C2]/r74.[k^C1_mC2]+C4/r74.[k^C1_mC4]))+
r74.[k^MASP1_catC2]*[MBL:MASP1]*[C4b:C2]/(r74.[k^MASP1_mC2]*
(1+[C4b:C2]/r74.[k^MASP1_mC2]+
[fC3b:C4b:C2]/r74.[k^MASP1_mC2]+C4/r74.[k^MASP1_mC4]))+
r74.[k^MASP2_catC2]*[MBL:MASP2]*[C4b:C2]/(r74.[k^MASP2_mC2]*
(1+[C4b:C2]/r74.[k^MASP2_mC2]+
[fC3b:C4b:C2]/r74.[k^MASP2_mC2]+C4/r74.[k^MASP2_mC4])))*Plasma))
```

ODE-67

```
d(P)/dt = 1/Plasma*(-(([K^+_C3bP]*P*C3b)*Plasma-
([K^-_C3bP]*[C3b:P])*Plasma) -
(([k^+_fC3bBbP]*P*[fC3b:Bb]-r99.[k^-_fC3bBbP]*[fC3b:Bb:P])*Plasma) -
(([k^+_fC3bBbC3bP]*P*[fC3b:Bb:C3b])*Plasma-
(r118.[k^-_fC3bBbC3bP]*[fC3b:Bb:C3b:P])*Plasma) -
(([k^+_fC3bFBP]*P*[fC3b:FB])*Plasma-([k^-_fC3bFBP]*[fC3b:FB:P])*Plasma) -
(([k^-_dP]*P)*Plasma) + (([k^+_sP])*Plasma) -
(([k^+_lgGfC3bFBP]*P*[lgG:fC3b:FB])*Plasma-
([k^-_lgGfC3bFBP]*[lgG:fC3b:FB:P])*Plasma) -
(([k^+_fC3bC4bP]*P*[fC3b:C4b])*Plasma-
([k^-_fC3bC4bP]*[fC3b:C4b:P])*Plasma))
```

ODE-68

```
d([C3b:P])/dt = 1/Plasma*((([K^+_C3bP]*P*C3b)*Plasma-
([K^-_C3bP]*[C3b:P])*Plasma))
```

ODE-69

```
d([fC3b:Bb:P])/dt = 1/Plasma*((([k^+_fC3bBbP]*P*[fC3b:Bb]-
r99.[k^-_fC3bBbP]*[fC3b:Bb:P])*Plasma))
```

ODE-70

```
d([fC3b:Bb:C3b:P])/dt = 1/Plasma*((([k^+_fC3bBbC3bP]*P*[fC3b:Bb:C3b])*Plasma-
(r118.[k^-_fC3bBbC3bP]*[fC3b:Bb:C3b:P])*Plasma))
```

ODE-71

```
d([fC3b:FB:P])/dt = 1/Plasma*((([k^+_fC3bFBP]*P*[fC3b:FB])*Plasma-
([k^-_fC3bFBP]*[fC3b:FB:P])*Plasma) -
((r110.[k^FD_catfC3bFBP]*[fC3b:FB:P]*FD/(r110.[k^FD_mfC3bFBP]+
[fC3b:FB:P]+[fC3b:FB]+[C3W:FB]+[1gG:fC3b:FB]+
[1gG:fC3b:FB:P]+[C3b:FB]+[fC3b:C4b:FB]+[fC3b:C4b:P:FB]+
[fC3b:C3b:FB]))*Plasma))
```

ODE-72

```
d([C1r:C1s])/dt = 1/Plasma*(-(([k^+_C1qrs]*[C1r:C1s]*C1q)*Plasma-
([k^-_C1qrs]*[C1q:C1r:C1s])*Plasma) +
(([k^+_C1rC1s]*C1r*C1s)*Plasma-([k^-_C1rC1s]*[C1r:C1s])*Plasma))
```

ODE-73

```
d([C1q:C1r:C1s])/dt = 1/Plasma*((([k^+_C1qrs]*[C1r:C1s]*C1q)*Plasma-
([k^-_C1qrs]*[C1q:C1r:C1s])*Plasma) - (([k^+_C1]*[C1q:C1r:C1s])*Plasma))
```

ODE-74

```
d(C1)/dt = 1/Plasma*(-(([k^+_C1C1INH]*C1INH*C1)*Plasma) +
(([k^+_C1]*[C1q:C1r:C1s])*Plasma))
```

ODE-75

```
d([C3W:Bb:FH])/dt = 1/Plasma*((([k^+_C3WBbFH]*FH*[C3W:Bb])*Plasma-
([k^-_C3WBbFH]*[C3W:Bb:FH])*Plasma) - ((r40.[k^-_dC3WBbFH]*[C3W:Bb:FH])*Plasma))
```

ODE-76

```
d(Ba)/dt = 1/Plasma*((([k^FD_catfC3bFB]*[fC3b:FB]*FD/([k^FD_mfC3bFB]+
[fC3b:FB]+[fC3b:FB:P]+[C3W:FB]+[lgG:fC3b:FB]+[lgG:fC3b:FB:P]+[C3b:FB]+
[fC3b:C4b:FB]+[fC3b:C4b:P:FB]+[fC3b:C3b:FB])+
[k^KAL_catfC3bFB]*[fC3b:FB]*KAL/([k^KAL_mfC3bFB]+[fC3b:FB]))*Plasma) +
((r26.[k^FD_catC3WFB]*[C3W:FB]*FD/(r26.[k^FD_mC3WFB]+[C3W:FB]+[fC3b:FB]+
[fC3b:FB:P]+[lgG:fC3b:FB]+[lgG:fC3b:FB:P]+[C3b:FB]+[fC3b:C4b:FB]+[fC3b:C4b:P:FB]+
[fC3b:C3b:FB])+[k^KAL_catC3WFB]*[C3W:FB]*KAL/([k^KAL_mC3WFB]+[C3W:FB]))*Plasma) +
((r43.[k^FD_catfC3bFBP]*[fC3b:FB:P]*FD/(r43.[k^FD_mfC3bFBP]+[fC3b:FB:P]+
[lgG:fC3b:FB]+[lgG:fC3b:FB:P]+[C3b:FB]+[fC3b:C4b:FB]+[fC3b:C4b:P:FB]+[fC3b:C3b:FB])+
r43.[k^FD_catlgGfC3bFB]*[lgG:fC3b:FB]*FD/(r43.[k^FD_mlgGfC3bFB]+[lgG:fC3b:FB]+
[fC3b:FB:P]+[lgG:fC3b:FB:P]+[C3b:FB]+[fC3b:C4b:FB]+[fC3b:C4b:P:FB]+[fC3b:C3b:FB])+
[k^FD_catlgGfC3bFBP]*[lgG:fC3b:FB:P]*FD/([k^FD_mlgGfC3bFBP]+[lgG:fC3b:FB:P]+[fC3b:FB:P]+
[lgG:fC3b:FB]+[C3b:FB]+[fC3b:C4b:FB]+[fC3b:C4b:P:FB]+[fC3b:C3b:FB])+
r43.[k^FD_catfC3bC4bFB]*[fC3b:C4b:FB]*FD/(r43.[k^FD_mfC3bC4bFB]+[fC3b:C4b:FB]+
[fC3b:FB:P]+[lgG:fC3b:FB]+[lgG:fC3b:FB:P]+[C3b:FB]+[fC3b:C4b:P:FB]+[fC3b:C3b:FB])+
r43.[k^FD_catC3bFB]*[C3b:FB]*FD/(r43.[k^FD_mC3bFB]+[C3b:FB]+[fC3b:C4b:FB]+[fC3b:FB:P]+
[lgG:fC3b:FB]+[lgG:fC3b:FB:P]+[fC3b:C4b:P:FB]+[fC3b:C3b:FB])+
r43.[k^FD_catfC3bC4bPFB]*[fC3b:C4b:P:FB]*FD/(r43.[k^FD_mfC3bC4bPFB]+
[fC3b:C4b:P:FB]+[C3b:FB]+[fC3b:C4b:FB]+[fC3b:FB:P]+[lgG:fC3b:FB]+[lgG:fC3b:FB:P]+
[fC3b:C3b:FB])+r43.[k^FD_catfC3bC3bFB]*[fC3b:C3b:FB]*FD/(r43.[k^FD_mfC3bC3bFB]+
[fC3b:C3b:FB]+[C3b:FB]+[fC3b:FB:P]+[lgG:fC3b:FB]+[lgG:fC3b:FB:P]+
[fC3b:C4b:FB]+[fC3b:C4b:P:FB]))*Plasma)-(([k^-_dBa]*Ba)*Plasma))
```

ODE-77

```
d(Bb)/dt = 1/Plasma*(((r29.[k^-_dC3WBbFH]*[C3W:Bb:FH]+r29.[k^-_dfC3bBbFH]*[fC3b:Bb:FH]+
r29.[k^-_fC3bBbP]*[fC3b:Bb:P]+r29.[k^-_dfC3bBbCR1]*[fC3b:Bb:CR1]+
r29.[k^-_dfC3bBbDAF]*[fC3b:Bb:DAF]+r29.[k^-_dfC3bBbC3bFH]*[fC3b:Bb:C3b:FH]+
r29.[k^-_fC3bBb]*[fC3b:Bb]+r29.[k^-_dfC3bBbC3bCR1]*[fC3b:Bb:C3b:CR1]+
r29.[k^-_dfC3bBbC3bDAF]*[fC3b:Bb:C3b:DAF]+[k^-_dC4bC2aC3bDAF]*[C4b:C2a:C3b:DAF]+
r29.[k^-_dC4bC2aC3bCR1]*[C4b:C2a:C3b:CR1]+r29.[k^-_dC4bC2aCR1]*[C4b:C2a:CR1]+
r29.[k^-_dC4bC2aDAF]*[C4b:C2a:DAF]+r29.[k^-_fC3bBbC3bP]*[fC3b:Bb:C3b:P])*Plasma) +
((r28.[k^-_C3WBb]*[C3W:Bb])*Plasma) - (([k^-_dBb]*Bb)*Plasma))
```

ODE-78

```
d([lgG:fC3b:FB])/dt = 1/Plasma*((([k^+_lgGfC3bFB]*[lgG:fC3b]*FB)*Plasma-
([k^-_lgGfC3bFB]*[lgG:fC3b:FB])*Plasma) - (([k^+_lgGfC3bFBP]*P*[lgG:fC3b:FB])*Plasma-
([k^-_lgGfC3bFBP]*[lgG:fC3b:FB:P])*Plasma) -
((r61.[k^FD_catlgGfC3bFB]*[lgG:fC3b:FB]*FD/(r61.[k^FD_mlgGfC3bFB]+
[lgG:fC3b:FB]+[fC3b:FB]+[fC3b:FB:P]+[lgG:fC3b:FB:P]+[C3W:FB]+[C3b:FB]+
[fC3b:C4b:FB]+[fC3b:C4b:P:FB]+[fC3b:C3b:FB]))*Plasma))
```

ODE-79

```
d([lgG:fC3b:FB:P])/dt = 1/Plasma*((([k^+_lgGfC3bFBP]*P*[lgG:fC3b:FB])*Plasma-
([k^-_lgGfC3bFBP]*[lgG:fC3b:FB:P])*Plasma) -
((r265.[k^FD_catC3WFB]*[C3W:FB]*FD/(r265.[k^FD_mC3WFB]+[C3W:FB]+[fC3b:FB]+[fC3b:FB:P]+
[lgG:fC3b:FB]+[lgG:fC3b:FB:P]+[C3b:FB]+[fC3b:C4b:FB]+[fC3b:C4b:P:FB]+[fC3b:C3b:FB]))*Plasma))
```

ODE-80

```
d(W)/dt = 1/Plasma*(-(([k^+_fC3b]*fC3b*W)*Plasma))
```

ODE-81

```
d([fC3b:CR1])/dt = 1/Plasma*((([k^+_fC3bCR1]*CR1*fC3b)*Plasma-
([k^-_fC3bCR1]*[fC3b:CR1])*Plasma) -
((r112.[k^FI_catfC3bCR1]*[fC3b:CR1]*FI/(r112.[k^FI_mfC3bCR1]+
[fC3b:CR1]+[C3b:CR1]+[C3b:FH]+[fC3b:FH]+
[C3W:FH]+[fC3b:C4b:FH]+[fC3b:C4b:CR1]))*Plasma))
```

ODE-82

```
d([C3b:FB])/dt = 1/Plasma*((([k^+_C3bFB]*FB*C3b)*Plasma-
([k^-_C3bFB]*[C3b:FB])*Plasma) -
((r91.[k^FD_catC3bFB]*[C3b:FB]*FD/(r91.[k^FD_mC3bFB]+[C3b:FB]+
[fC3b:FB]+[fC3b:FB:P]+[lgG:fC3b:FB]+
[lgG:fC3b:FB:P]+[C3W:FB]+[fC3b:C4b:FB]+[fC3b:C4b:P:FB]+[fC3b:C3b:FB]))*Plasma) -
(([k^+_fC3bC3bFB]*[C3b:FB]*fC3b)*Plasma-([k^-_fC3bC3bFB]*[fC3b:C3b:FB])*Plasma))
```

ODE-83

```
d(MASP2)/dt = 1/Plasma*(-((r14.[k^+_C1INHMASP2]*C1INH*MASP2)*Plasma) -
(([k^+_MBLMASP2]*MBL*MASP2)*Plasma-([k^-_MBLMASP2]*[MBL:MASP2])*Plasma) -
(([k^+_MASP2C4]*MASP2*C4)*Plasma-([k^-_MASP2C4]*[MASP2:C4])*Plasma) +
(([k^+_CoV2NMASP2]*MASP2*CoV2N)*Plasma))
```

ODE-84

```
d([MBL:MASP2])/dt = 1/Plasma*((([k^+_MBLMASP2]*MBL*MASP2)*Plasma-
([k^-_MBLMASP2]*[MBL:MASP2])*Plasma) -
((r11.[k^+_C1INHMASP2]*C1INH*[MBL:MASP2])*Plasma))
```

ODE-85

```
d([C1INH:MASP2])/dt = 1/Plasma*(((r14.[k^+_C1INHMASP2]*C1INH*MASP2)*Plasma) +
((r15.[k^+_C1INHMASP2]*C1INH*[MBL:MASP2])*Plasma))
```

ODE-86

```
d([C1INH:MASP1])/dt = 1/Plasma*(((r12.[k^+_C1INHMASP1]*C1INH*MASP1)*Plasma) +
((r13.[k^+_C1INHMASP1]*C1INH*[MBL:MASP1])*Plasma))
```

ODE-87

```
d([fC3b:C4b])/dt = 1/Plasma*((([k^+_fC3bC4b]*fC3b*C4b)*Plasma) -
(([k^+_fC3bC4bFB]*FB*[fC3b:C4b]-[k^-_fC3bC4bFB]*[fC3b:C4b:FB])*Plasma) -
(([k^+_fC3bC4bFH]*FH*[fC3b:C4b])*Plasma-([k^-_fC3bC4bFH]*[fC3b:C4b:FH])*Plasma) -
(([k^+_fC3bC4bCR1]*[fC3b:C4b]*CR1)*Plasma-([k^-_fC3bC4bCR1]*[fC3b:C4b:CR1])*Plasma) -
(([k^+_fC3bC4bP]*P*[fC3b:C4b])*Plasma-([k^-_fC3bC4bP]*[fC3b:C4b:P])*Plasma) -
(([k^+_fC3bC4bC2]*[fC3b:C4b]*C2)*Plasma-([k^-_fC3bC4bC2]*[fC3b:C4b:C2])*Plasma))
```

ODE-88

```
d([fC3b:C4b:FB])/dt = 1/Plasma*((([k^+_fC3bC4bFB]*FB*[fC3b:C4b]-
[k^-_fC3bC4bFB]*[fC3b:C4b:FB])*Plasma) -
((r67.[k^FD_catfC3bC4bFB]*[fC3b:C4b:FB]*FD/(r67.[k^FD_mfC3bC4bFB]+
[fC3b:C4b:FB]+[C3W:FB]+[fC3b:FB]+[fC3b:FB:P]+[lgG:fC3b:FB]+
[lgG:fC3b:FB:P]+[C3b:FB]+[fC3b:C4b:P:FB]+[fC3b:C3b:FB]))*Plasma))
```

ODE-89

```
d([fC3b:C4b:FH])/dt = 1/Plasma*((([k^+_fC3bC4bFH]*FH*[fC3b:C4b])*Plasma-
([k^-_fC3bC4bFH]*[fC3b:C4b:FH])*Plasma) -
((r82.[k^FI_catfC3bC4bFH]*[fC3b:C4b:FH]*FI/(r82.[k^FI_mfC3bC4bFH]+
[fC3b:C4b:FH]+[C3W:FH]+[fC3b:FH]+[fC3b:CR1]+
[C3b:FH]+[C3b:CR1]+[fC3b:C4b:CR1]))*Plasma))
```

ODE-90

```
d([fC3b:C4b:CR1])/dt = 1/Plasma*((([k^+_fC3bC4bCR1]*[fC3b:C4b]*CR1)*Plasma-
([k^-_fC3bC4bCR1]*[fC3b:C4b:CR1])*Plasma) -
((r267.[k^FI_catfC3bC4bCR1]*[fC3b:C4b:CR1]*FI/(r267.[k^FI_mfC3bC4bCR1]+
[fC3b:C4b:CR1]+[C3W:FB]+[fC3b:FH]+[fC3b:CR1]+
[C3b:FH]+[C3b:CR1]+[fC3b:C4b:FH]))*Plasma))
```

ODE-91

```
d([fC3b:C4b:P])/dt = 1/Plasma*((([k^+_fC3bC4bP]*P*[fC3b:C4b])*Plasma-
([k^-_fC3bC4bP]*[fC3b:C4b:P])*Plasma) -
(([k^+_fC3bC4bPFB]*FB*[fC3b:C4b:P])*Plasma-
([k^-_fC3bC4bPFB]*[fC3b:C4b:P:FB])*Plasma))
```

ODE-92

```
d([fC3b:C4b:C2])/dt = 1/Plasma*((([k^+_fC3bC4bC2]*[fC3b:C4b]*C2)*Plasma-
([k^-_fC3bC4bC2]*[fC3b:C4b:C2])*Plasma) -
((r107.[k^C1_catC2]*[fC3b:C4b:C2]*C2/(r107.[k^C1_mC2]*(1+[C4b:C2]/r107.[k^C1_mC2]+
[fC3b:C4b:C2]/r107.[k^C1_mC2]+C4/r107.[k^C1_mC4]))+
r107.[k^MASP1_catC2]*[MBL:MASP1]*[fC3b:C4b:C2]/(r107.[k^MASP1_mC2]*(1+[C4b:C2]/r107.[k^MASP1_mC2]+
[fC3b:C4b:C2]/r107.[k^MASP1_mC2]+C4/r107.[k^MASP1_mC4]))+
r107.[k^MASP2_catC2]*[MBL:MASP2]*[fC3b:C4b:C2]/(r107.[k^MASP2_mC2]*(1+[C4b:C2]/r107.[k^MASP2_mC2]+
[fC3b:C4b:C2]/r107.[k^MASP2_mC2]+C4/r107.[k^MASP2_mC4])))*Plasma))
```

ODE-93

```
d([fC3b:C4b:P:FB])/dt = 1/Plasma*((([k^+_fC3bC4bPFB]*FB*[fC3b:C4b:P])*Plasma-
([k^-_fC3bC4bPFB]*[fC3b:C4b:P:FB])*Plasma) -
((r69.[k^FD_catfC3bC4bPFB]*[fC3b:C4b:P:FB]*FD/(r69.[k^FD_mfC3bC4bPFB]+
[fC3b:C4b:P:FB]+[fC3b:C4b:FB]+[C3W:FB]+[fC3b:FB]+[fC3b:FB:P]+[lgG:fC3b:FB]+
[lgG:fC3b:FB:P]+[C3b:FB]+[fC3b:C3b:FB]))*Plasma))
```

ODE-94

```
d(CoV2N)/dt = 1/Plasma*(-(([k^+_CoV2NMASP2]*MASP2*CoV2N)*Plasma) -
(([k^CoV2N_catgC1qR]*gC1qR*CoV2N)*Plasma) -
(([k^+_CoV2NHK]*HK*CoV2N)*Plasma))
```

ODE-95

```
d(IL-6)/dt = 1/Plasma*((([k^C5aC5aR1_catIL6]*[C5a:C5aR1])*Plasma) +
(([k^C3aC3aR1_catIL6]*[C3a:C3aR1])*Plasma) - (([k^IL6_catF1]*IL-6)*Plasma) +
(([k^BKB2R_catIL6]*[BK:B2R])*Plasma) - (([k^-_dIL6]*IL-6)*Plasma))
-(([k^+_IL-6IL-6R]*IL-6*IL-6R)*Plasma - ([k^-_IL-6IL-6R]*[IL-6:IL-6R])*Plasma))
```

ODE-96

```
d(C1r)/dt = 1/Plasma*(-(([k^+_C1rC1s]*C1r*C1s)*Plasma-
([k^-_C1rC1s]*[C1r:C1s])*Plasma) + (([k^F12a_catC1r]*C1r*F12a)*Plasma))
```

ODE-97

```
d(C1s)/dt = 1/Plasma*(-(([k^+_C1rC1s]*C1r*C1s)*Plasma-
([k^-_C1rC1s]*[C1r:C1s])*Plasma) - (([k^+_C1sC4]*C4*C1s)*Plasma-
([k^-_C1sC4]*[C1s:C4])*Plasma)) -
(([k^+_C1INHC1s]*C1INH*C1s)*Plasma))
```

ODE-98

```
d([C1s:C4])/dt = 1/Plasma*((([k^+_C1sC4]*C4*C1s)*Plasma-
([k^-_C1sC4]*[C1s:C4])*Plasma))
```

ODE-99

```
d([MASP2:C4])/dt = 1/Plasma*((([k^+_MASP2C4]*MASP2*C4)*Plasma-
([k^-_MASP2C4]*[MASP2:C4])*Plasma))
```

ODE-100

```
d([fC3b:C3b:FB])/dt = 1/Plasma*((([k^+_fC3bC3bFB]*[C3b:FB]*fC3b)*Plasma-
([k^-_fC3bC3bFB]*[fC3b:C3b:FB])*Plasma))
```

ODE-101

```
d(F7)/dt = 1/Plasma*(-(([k^+_TFF7]*F7*TF-[k^-_TFF7]*[TF:F7])*Plasma) -
(([k^TFF7a_catF7]*F7*[TF:F7a]+[k^F10a_catF7]*F7*F10a+[k^F2a_catF7]*F7*F2a)*Plasma))
```

ODE-102

```
d(F7a)/dt = 1/Plasma*(-(([k^+_TFF7a]*F7a*TF)*Plasma-([k^-_TFF7a]*[TF:F7a])*Plasma) +
(([k^TFF7a_catF7]*F7*[TF:F7a]+[k^F10a_catF7]*F7*F10a+[k^F2a_catF7]*F7*F2a)*Plasma) -
(([k^+_F10F7a]*F7a*F10-[k^-_F10F7a]*[F10:F7a])*Plasma) - (([k^+_F9F7a]*F9*F7a)*Plasma-
([k^-_F9F7a]*[F9:F7a])*Plasma) +
((r194.[k^F7a_catF9a]*[F9:F7a]+r194.[k^F7a_catF10a]*[F10:F7a])*Plasma))
```

ODE-103

```
d(F9)/dt = 1/Plasma*(-(([k^+_F9F7a]*F9*F7a)*Plasma-([k^-_F9F7a]*[F9:F7a])*Plasma) -
(([k^+_TFF7aF9]*[TF:F7a]*F9)*Plasma-([k^-_TFF7aF9]*[TF:F7a:F9])*Plasma) -
(([k^+_F11aF9]*F9*F11a)*Plasma-([k^-_F11aF9]*[F11a:F9])*Plasma))
```

ODE-104

d(F9a)/dt = 1/Plasma*(-(([k^+_F9aF10]*F10*F9a)*Plasma-([k^-_F9aF10]*[F9a:F10])*Plasma) - (([k^+_F9aAT3]*AT3*F9a)*Plasma) + ((r203.[k^F9a_catF10a]*[F9a:F10])*Plasma) - (([k^+_F9aF8a]*F9a*F8a)*Plasma-([k^-_F9aF8a]*[F9a:F8a])*Plasma) + ((r202.[k^F7a_catF9a]*[F9:F7a])*Plasma) + ((r208.[k^F11a_catF9a]*[F11a:F9])*Plasma))

ODE-105

d(F10)/dt = 1/Plasma*(-(([k^+_F9aF10]*F10*F9a)*Plasma-([k^-_F9aF10]*[F9a:F10])*Plasma) - (([k^+_F10F7a]*F7a*F10-[k^-_F10F7a]*[F10:F7a])*Plasma) - (([k^+_TFF7aF10]*[TF:F7a]*F10)*Plasma-([k^-_TFF7aF10]*[TF:F7a:F10])*Plasma) - (([k^+_F9aF8aF10]*[F9a:F8a]*F10)*Plasma-([k^-_F9aF8aF10]*[F9a:F8a:F10])*Plasma))

ODE-106

d(F10a)/dt = 1/Plasma*(-(([k^+_F10aAT3]*F10a*AT3)*Plasma) - (([k^+_F10aTFPI]*F10a*TFPI)*Plasma-([k^-_F10aTFPI]*[F10a:TFPI])*Plasma) - (([k^F10a_catCoV2S]*CoV2S*F10a/([k^F10a_mCoV2S]+CoV2S))*Plasma) - (([k^+_TFF7aF10a]*F10a*[TF:F7a])*Plasma-([k^-_TFF7aF10a]*[TF:F7a:F10a])*Plasma) - (([k^+_F10aF5a]*F5a*F10a)*Plasma-([k^-_F10aF5a]*[F10a:F5a])*Plasma) + ((r200.[k^F9a_catF10a]*[F9a:F10])*Plasma) - (([k^+_F10aF8]*F10a*F8)*Plasma-([k^-_F10aF8]*[F10a:F8])*Plasma) + ((r217.[k^F9aF8a_catF10]*[F9a:F8a:F10])*Plasma) + ((r201.[k^F10a_catF8a]*[F10a:F8])*Plasma) + ((r198.[k^F7a_catF10a]*[F10:F7a])*Plasma))

ODE-107

d(F8)/dt = 1/Plasma*(-(([k^F2a_catF8]*F8*F2a)*Plasma) - (([k^+_F10aF8]*F10a*F8)*Plasma-([k^-_F10aF8]*[F10a:F8])*Plasma))

ODE-108

d(F8a)/dt = 1/Plasma*((([k^F2a_catF8]*F8*F2a)*Plasma) + ((r219.[k^F10a_catF8a]*[F10a:F8])*Plasma) - (([k^+_F9aF8a]*F9a*F8a)*Plasma-([k^-_F9aF8a]*[F9a:F8a])*Plasma))

ODE-109

d(F2a)/dt = 1/Plasma*(((([k^F10a_catF2]*F2*F10a+[k^MASP1_catF2]*F2*MASP1+[k^MASP2_catF2]*F2*MASP2)*Plasma) - (([k^+_F2aAT3]*F2a*AT3)*Plasma) - (([k^+_F2aA2M]*F2a*A2M)*Plasma) - (([k^F2a_catCoV2S]*CoV2S*F2a/([k^F2a_mCoV2S]+CoV2S))*Plasma) - (([k^+_F11F2a]*F2a*F11)*Plasma-([k^-_F11F2a]*[F11:F2a])*Plasma) + ((r180.[k^F11_catF2a]*[F11:F2a])*Plasma) - (([k^+_C1INHF2a]*C1INH*F2a)*Plasma) - (([k^+_FHF2a]*FH*F2a)*Plasma) - (([k^+_F2aF1]*F2a*F1)*Plasma-([k^-_F2aF1]*[F2a:F1])*Plasma))

ODE-110

```
d(F5)/dt = 1/Plasma*(-(([k^F2a_catF5]*F5*F2a)*Plasma))
```

ODE-111

```
d(F5a)/dt = 1/Plasma*((([k^F2a_catF5]*F5*F2a)*Plasma) -
(([k^+_F10aF5a]*F5a*F10a)*Plasma-([k^-_F10aF5a]*[F10a:F5a])*Plasma))
```

ODE-112

```
d(TF)/dt = 1/Plasma*(-(([k^+_TFF7]*F7*TF-[k^-_TFF7]*[TF:F7])*Plasma) -
(([k^+_TFF7a]*F7a*TF)*Plasma-([k^-_TFF7a]*[TF:F7a])*Plasma) +
(([k^C5a_catTF]*C5a)*Plasma) + (([k^C3a_catTF]*C3a)*Plasma))
```

ODE-113

```
d(F2)/dt = 1/Plasma*(-(([k^F10a_catF2]*F2*F10a+[k^MASP1_catF2]*F2*MASP1+
[k^MASP2_catF2]*F2*MASP2)*Plasma) - (([k^+_F10aF5aF2]*[F10a:F5a]*F2)*Plasma-
([k^-_F10aF5aF2]*[F10a:F5a:F2])*Plasma) +
(([k^MAC_catF2]*[C5b:C6:C7:C8:C9])*Plasma))
```

ODE-114

```
d(Pn)/dt = 1/Plasma*(-(([k^+_C1INHPn]*C1INH*Pn)*Plasma) +
(([k^KAL_catGlu-Pg]*[Glu-Pg]*KAL/([k^KAL_mGlu-Pg]+[Glu-Pg])+
[k^tPA_catGlu-Pg]*[Glu-Pg]*tPA/([k^tPA_mGlu-Pg]+[Glu-Pg]))*Plasma) -
(([k^Pn_catLys-Pg]*Pn)*Plasma))
```

ODE-115

```
d(AT3)/dt = 1/Plasma*(-(([k^+_F10aAT3]*F10a*AT3)*Plasma) -
(([k^+_F2aAT3]*F2a*AT3)*Plasma) - (([k^+_F9aAT3]*AT3*F9a)*Plasma) -
(([k^+_TFF7aAT3]*[TF:F7a]*AT3)*Plasma) -
(([k^+_F12aAT3]*AT3*F12a)*Plasma) - (([k^+_F11aAT3]*AT3*F11a)*Plasma))
```

ODE-116

```
d([F10a:AT3])/dt = 1/Plasma*((([k^+_F10aAT3]*F10a*AT3)*Plasma))
```

ODE-117

```
d([F2a:AT3])/dt = 1/Plasma*((([k^+_F2aAT3]*F2a*AT3)*Plasma))
```

ODE-118

```
d([F9a:AT3])/dt = 1/Plasma*((([k^+_F9aAT3]*AT3*F9a)*Plasma))
```

ODE-119

```
d(TFPI)/dt = 1/Plasma*(-(([k^+_F10aTFPI]*F10a*TFPI)*Plasma-
([k^-_F10aTFPI]*[F10a:TFPI])*Plasma) -
(([k^+_TFF7aF10aTFPI]*[TF:F7a:F10a]*TFPI)*Plasma-
([k^-_TFF7aF10aTFPI]*[TF:F7a:F10a:TFPI])*Plasma))
```

ODE-120

```
d([F10a:TFPI])/dt = 1/Plasma*((([k^+_F10aTFPI]*F10a*TFPI)*Plasma-
([k^-_F10aTFPI]*[F10a:TFPI])*Plasma) -
(([k^+_F10aTFPITFF7a]*[F10a:TFPI]*[TF:F7a])*Plasma))
```

ODE-121

```
d(A2M)/dt = 1/Plasma*(-(([k^+_F2aA2M]*F2a*A2M)*Plasma))
```

ODE-122

```
d([F2a:A2M])/dt = 1/Plasma*((([k^+_F2aA2M]*F2a*A2M)*Plasma))
```

ODE-123

```
d([TF:F7])/dt = 1/Plasma*((([k^+_TFF7]*F7*TF-[k^-_TFF7]*[TF:F7])*Plasma))
```

ODE-124

```
d([TF:F7a])/dt = 1/Plasma*((([k^+_TFF7a]*F7a*TF)*Plasma-
([k^-_TFF7a]*[TF:F7a])*Plasma) - (([k^+_F10aTFPITFF7a]*[F10a:TFPI]*[TF:F7a])*Plasma) -
(([k^+_TFF7aF10a]*F10a*[TF:F7a])*Plasma -
([k^-_TFF7aF10a]*[TF:F7a:F10a])*Plasma) - (([k^+_TFF7aF10]*[TF:F7a]*F10)*Plasma-
([k^-_TFF7aF10]*[TF:F7a:F10])*Plasma) - (([k^+_TFF7aAT3]*[TF:F7a]*AT3)*Plasma) -
(([k^+_TFF7aF9]*[TF:F7a]*F9)*Plasma-([k^-_TFF7aF9]*[TF:F7a:F9])*Plasma) +
(([k^TFF7a_catF9]*[TF:F7a:F9])*Plasma))
```

ODE-125

```
d([F10:F7a])/dt = 1/Plasma*((([k^+_F10F7a]*F7a*F10-[k^-_F10F7a]*[F10:F7a])*Plasma) -
((r198.[k^F7a_catF10a]*[F10:F7a])*Plasma))
```

ODE-126

```
d([F9:F7a])/dt = 1/Plasma*((([k^+_F9F7a]*F9*F7a)*Plasma-([k^-_F9F7a]*[F9:F7a])*Plasma) -
((r202.[k^F7a_catF9a]*[F9:F7a])*Plasma))
```

ODE-127

```
d([TF:F7a:F10a])/dt = 1/Plasma*((([k^+_TFF7aF10a]*F10a*[TF:F7a])*Plasma-
([k^-_TFF7aF10a]*[TF:F7a:F10a])*Plasma) + (([k^TFF7a_catF10]*[TF:F7a:F10])*Plasma) -
(([k^+_TFF7aF10aTFPI]*[TF:F7a:F10a]*TFPI)*Plasma-
([k^-_TFF7aF10aTFPI]*[TF:F7a:F10a:TFPI])*Plasma))
```

ODE-128

```
d([F10a:F5a])/dt = 1/Plasma*((([k^+_F10aF5a]*F5a*F10a)*Plasma-
([k^-_F10aF5a]*[F10a:F5a])*Plasma) - (([k^+_F10aF5aF2]*[F10a:F5a]*F2)*Plasma-
([k^-_F10aF5aF2]*[F10a:F5a:F2])*Plasma))
```

ODE-129

```
d([F10a:F5a:F2])/dt = 1/Plasma*((([k^+_F10aF5aF2]*[F10a:F5a]*F2)*Plasma-
([k^-_F10aF5aF2]*[F10a:F5a:F2])*Plasma))
```

ODE-130

```
d([F9a:F10])/dt = 1/Plasma*((([k^+_F9aF10]*F10*F9a)*Plasma-
([k^-_F9aF10]*[F9a:F10])*Plasma) -
((r200.[k^F9a_catF10a]*[F9a:F10])*Plasma))
```

ODE-131

```
d([F11:F2a])/dt = 1/Plasma*((([k^+_F11F2a]*F2a*F11)*Plasma-
([k^-_F11F2a]*[F11:F2a])*Plasma) - ((r180.[k^F11_catF2a]*[F11:F2a])*Plasma))
```

ODE-132

```
d([TF:F7a:F10])/dt = 1/Plasma*((([k^+_TFF7aF10]*[TF:F7a]*F10)*Plasma-
([k^-_TFF7aF10]*[TF:F7a:F10])*Plasma) - (([k^TFF7a_catF10]*[TF:F7a:F10])*Plasma))
```

ODE-133

```
d([TF:F7a:F10a:TFPI])/dt = 1/Plasma*((([k^+_F10aTFPITFF7a]*[F10a:TFPI]*[TF:F7a])*Plasma) +
(([k^+_TFF7aF10aTFPI]*[TF:F7a:F10a]*TFPI)*Plasma-
([k^-_TFF7aF10aTFPI]*[TF:F7a:F10a:TFPI])*Plasma))
```

ODE-134

```
d([F10a:F8])/dt = 1/Plasma*((([k^+_F10aF8]*F10a*F8)*Plasma-
([k^-_F10aF8]*[F10a:F8])*Plasma) -
((r219.[k^F10a_catF8a]*[F10a:F8])*Plasma))
```

ODE-135

```
d([F9a:F8a])/dt = 1/Plasma*((([k^+_F9aF8a]*F9a*F8a)*Plasma-
([k^-_F9aF8a]*[F9a:F8a])*Plasma) -
(([k^+_F9aF8aF10]*[F9a:F8a]*F10)*Plasma-
([k^-_F9aF8aF10]*[F9a:F8a:F10])*Plasma) +
((r206.[k^F9aF8a_catF10]*[F9a:F8a:F10])*Plasma))
```

ODE-136

```
d([F9a:F8a:F10])/dt = 1/Plasma*((([k^+_F9aF8aF10]*[F9a:F8a]*F10)*Plasma-
([k^-_F9aF8aF10]*[F9a:F8a:F10])*Plasma) -
((r217.[k^F9aF8a_catF10]*[F9a:F8a:F10])*Plasma))
```

ODE-137

```
d([TF:F7a:AT3])/dt = 1/Plasma*((([k^+_TFF7aAT3]*[TF:F7a]*AT3)*Plasma))
```

ODE-138

```
d([TF:F7a:F9])/dt = 1/Plasma*((([k^+_TFF7aF9]*[TF:F7a]*F9)*Plasma-
([k^-_TFF7aF9]*[TF:F7a:F9])*Plasma) -
(([k^TFF7a_catF9]*[TF:F7a:F9])*Plasma))
```

ODE-139

```
d(F12)/dt = 1/Plasma*(-((([k^+_F12F12a]*F12*F12a)*Plasma-
([k^-_F12F12a]*[F12:F12a])*Plasma) -
(([k^+_KALF12]*F12*KAL)*Plasma-([k^-_KALF12]*[KAL:F12])*Plasma) -
(([k^+_CoV2SF12]*F12*CoV2S+[k^+_CoV2NF12]*F12*CoV2N+[k^+_CoV2MF12]*F12*CoV2M +
[k^+_CoV2EF12]*F12*CoV2E+[k^Pn_catF12]*Pn*F12)*Plasma) -
(([k^+_gC1qRF12]*gC1qR*F12)*Plasma-
([k^-_gC1qRF12]*[gC1qR:F12])*Plasma))
```

ODE-140

```
d(F12a)/dt = 1/Plasma*(-((([k^+_F12F12a]*F12*F12a)*Plasma-
([k^-_F12F12a]*[F12:F12a])*Plasma) -
(([k^+_F12aAT3]*AT3*F12a)*Plasma) - (([k^+_F12aF11]*F11*F12a)*Plasma-
([k^-_F12aF11]*[F12a:F11])*Plasma) +
(([k^KAL_catF12a]*[KAL:F12])*Plasma) - (([k^F12a_catC1r]*C1r*F12a)*Plasma) -
(([k^+_C1INHF12a]*C1INH*F12a)*Plasma) + ((r182.[k^F12a_catF11a]*[F12a:F11])*Plasma) +
(([k^F12F12a_catF12a]*[F12:F12a])*Plasma) +
(([k^+_CoV2SF12]*F12*CoV2S+[k^+_CoV2NF12]*F12*CoV2N+[k^+_CoV2MF12]*F12*CoV2M+
[k^+_CoV2EF12]*F12*CoV2E+[k^Pn_catF12]*Pn*F12)*Plasma) -
(([k^+_PKALF12a]*F12a*[P-KAL])*Plasma-([k^-_PKALF12a]*[P-KAL:F12a])*Plasma) +
(([k^gC1qRF12_catF12a]*[gC1qR:F12])*Plasma))
```

ODE-141

```
d([F12:F12a])/dt = 1/Plasma*(((([k^+_F12F12a]*F12*F12a)*Plasma-
([k^-_F12F12a]*[F12:F12a])*Plasma) -
(([k^F12F12a_catF12a]*[F12:F12a])*Plasma))
```

ODE-142

```
d([F12a:AT3])/dt = 1/Plasma*(((([k^+_F12aAT3]*AT3*F12a)*Plasma))
```

ODE-143

```
d(F11)/dt = 1/Plasma*(-((([k^+_F11F2a]*F2a*F11)*Plasma-([k^-_F11F2a]*[F11:F2a])*Plasma) -
(([k^+_F12aF11]*F11*F12a)*Plasma-([k^-_F12aF11]*[F12a:F11])*Plasma) -
(([k^+_HKF11]*HK*F11)*Plasma-([k^-_HKF11]*[HK:F11])*Plasma))
```

ODE-144

```
d([F12a:F11])/dt = 1/Plasma*((([k^+_F12aF11]*F11*F12a)*Plasma-
([k^-_F12aF11]*[F12a:F11])*Plasma) - ((r185.[k^F12a_catF11a]*[F12a:F11])*Plasma))
```

ODE-145

```
d(F11a)/dt = 1/Plasma*(((r185.[k^F12a_catF11a]*[F12a:F11])*Plasma) -
(([k^+_F11aF9]*F9*F11a)*Plasma-([k^-_F11aF9]*[F11a:F9])*Plasma) -
(([k^+_F11aAT3]*AT3*F11a)*Plasma) +
((r186.[k^F11a_catF9a]*[F11a:F9]+r186.[k^F11_catF2a]*[F11:F2a])*Plasma) -
(([k^+_F11aC1INH]*C1INH*F11a)*Plasma) + (([k^HKF11_catF11a]*[HK:F11])*Plasma))
```

ODE-146

```
d([F11a:F9])/dt = 1/Plasma*((([k^+_F11aF9]*F9*F11a)*Plasma-
([k^-_F11aF9]*[F11a:F9])*Plasma) - ((r208.[k^F11a_catF9a]*[F11a:F9])*Plasma))
```

ODE-147

```
d([F11a:AT3])/dt = 1/Plasma*((([k^+_F11aAT3]*AT3*F11a)*Plasma))
```

ODE-148

```
d(KAL)/dt = 1/Plasma*(-(([k^+_KALF12]*F12*KAL)*Plasma-([k^-_KALF12]*[KAL:F12])*Plasma) -
(([k^+_C1INHKAL]*C1INH*KAL)*Plasma) - (([k^KAL_catHK]*KAL*HK)*Plasma) +
(([k^+_KALPKAL]*[P-KAL]*KAL)*Plasma) + ((r170.[k^F12a_catKAL]*[P-KAL:F12a])*Plasma))
```

ODE-149

```
d([KAL:F12])/dt = 1/Plasma*((([k^+_KALF12]*F12*KAL)*Plasma-
([k^-_KALF12]*[KAL:F12])*Plasma) - (([k^KAL_catF12a]*[KAL:F12])*Plasma))
```

ODE-150

```
d(F1a)/dt = 1/Plasma*(-(([k^+_FHF1a]*FH*F1a)*Plasma) +
(([k^F2a_catF1a]*[F2a:F1])*Plasma) +
(([k^F2a_catF1]*F2a*F1/([k^F2a_mF1]+F1)+
[k^Pn_catF1]*Pn*F1/([k^Pn_mF1]+F1)+
[k^MASP1_catF1]*F1*MASP1+
[k^MASP2_catF1]*F1*MASP2+[k^LysPg_catF1]*F1*[Lys-Pg])*Plasma) -
(([k^+_F1aLys-Pg]*F1a*[Lys-Pg])*Plasma-([k^-_F1aLys-Pg]*[F1a:Lys-Pg])*Plasma) -
(([k^Pn_catF1a]*F1a*Pn/([k^Pn_mF1a]+F1a))*Plasma))
```

ODE-151

```
d(F1)/dt = 1/Plasma*((([k^IL6_catF1]*IL6)*Plasma) -
(([k^+_F2aF1]*F2a*F1)*Plasma-([k^-_F2aF1]*[F2a:F1])*Plasma) -
(([k^F2a_catF1]*F2a*F1/([k^F2a_mF1]+F1)+
[k^Pn_catF1]*Pn*F1/([k^Pn_mF1]+F1)+[k^MASP1_catF1]*F1*MASP1+
[k^MASP2_catF1]*F1*MASP2+[k^LysPg_catF1]*F1*[Lys-Pg])*Plasma))
```

ODE-152

```
d([F2a:F1])/dt = 1/Plasma*((([k^+_F2aF1]*F2a*F1)*Plasma-
([k^-_F2aF1]*[F2a:F1])*Plasma) - (([k^F2a_catF1a]*[F2a:F1])*Plasma))
```

ODE-153

```
d(gC1qR)/dt = 1/Plasma*(-(([k^+_C1qgC1qR]*C1q*gC1qR)*Plasma-
([k^-_C1qgC1qR]*[C1q:gC1qR])*Plasma) +
(([k^CoV2E_catgC1qR]*gC1qR*CoV2E)*Plasma) +
(([k^CoV2N_catgC1qR]*gC1qR*CoV2N)*Plasma) +
(([k^CoV2S_catgC1qR]*CoV2S*gC1qR)*Plasma) +
(([k^CoV2M_catgC1qR]*gC1qR*CoV2M)*Plasma) -
(([k^+_gC1qRF12]*gC1qR*F12)*Plasma-
([k^-_gC1qRF12]*[gC1qR:F12])*Plasma) -
(([k^+_gC1qRHK]*HK*gC1qR)*Plasma-
([k^-_gC1qRHK]*[gC1qR:HK])*Plasma))
```

ODE-154

```
d([C1q:gC1qR])/dt = 1/Plasma*((([k^+_C1qgC1qR]*C1q*gC1qR)*Plasma-
([k^-_C1qgC1qR]*[C1q:gC1qR])*Plasma))
```

ODE-155

```
d(CoV2E)/dt = 1/Plasma*(-((([k^CoV2E_catgC1qR]*gC1qR*CoV2E)*Plasma) -
(([k^+_CoV2EHK]*HK*CoV2E)*Plasma))
```

ODE-156

```
d(CoV2M)/dt = 1/Plasma*(-((([k^CoV2M_catgC1qR]*gC1qR*CoV2M)*Plasma) -
(([k^+_CoV2MHK]*HK*CoV2M)*Plasma))
```

ODE-157

```
d(HK)/dt = 1/Plasma*((([k^KAL_catHK]*KAL*HK)*Plasma) +
(([k^+_CoV2MHK]*HK*CoV2M)*Plasma) +
(([k^+_CoV2EHK]*HK*CoV2E)*Plasma) +
(([k^+_CoV2NHK]*HK*CoV2N)*Plasma) +
(([k^+_CoV2SHK]*HK*CoV2S)*Plasma) -
(([k^+_PKALHK]*HK*[P-KAL]-[k^-_PKALHK]*[P-KAL:HK])*Plasma) -
(([k^+_HKF11]*HK*F11)*Plasma-([k^-_HKF11]*[HK:F11])*Plasma) -
(([k^+_gC1qRHK]*HK*gC1qR)*Plasma-([k^-_gC1qRHK]*[gC1qR:HK])*Plasma))
```

ODE-158

```
d([P-KAL])/dt = 1/Plasma*(-((([k^+_KALPKAL]*[P-KAL]*KAL)*Plasma) -
(([k^+_PKALF12a]*F12a*[P-KAL])*Plasma-([k^-_PKALF12a]*[P-KAL:F12a])*Plasma) -
(([k^+_PKALHK]*HK*[P-KAL]-[k^-_PKALHK]*[P-KAL:HK])*Plasma))
```

ODE-159

```
d(BK)/dt = 1/Plasma*((([k^PKALHK_catBK]*[P-KAL:HK])*Plasma) -
(([k^+_BKB2R]*B2R*BK)*Plasma-([k^-_BKB2R]*[BK:B2R])*Plasma) +
(([k^gC1qRHK_catBK]*[gC1qR:HK])*Plasma) - (([k^+_BKTAFIa]*TAFIa*BK)*Plasma))
```

ODE-160

```
d(B2R)/dt = 1/Plasma*(-(([k^+_BKB2R]*B2R*BK)*Plasma-
([k^-_BKB2R]*[BK:B2R])*Plasma))
```

ODE-161

```
d([BK:B2R])/dt = 1/Plasma*(-(([k^BKB2R_catIL6]*[BK:B2R])*Plasma) -
(([k^BKB2R_cattPA]*[BK:B2R])*Plasma) +
(([k^+_BKB2R]*B2R*BK)*Plasma-([k^-_BKB2R]*[BK:B2R])*Plasma))
```

ODE-162

```
d(TAFIa)/dt = 1/Plasma*(-(([k^+_C3aTAFIa]*TAFIa*C3a)*Plasma) +
(([k^F2a_catTAFI]*TAFI*F2a/([k^F2a_mTAFI]+TAFI)+
[k^Pn_catTAFI]*TAFI*Pn/([k^Pn_mTAFI]+TAFI)+
[k^MASP1_catTAFI]*TAFI*MASP1/([k^MASP1_mTAFI]+TAFI))*Plasma) -
(([k^+_C5aTAFIa]*TAFIa*C5a)*Plasma) -
(([k^+_Lys-PgTAFIa]*TAFIa*[Lys-Pg])*Plasma) -
(([k^+_BKTAFIa]*TAFIa*BK)*Plasma) -
((r273.[k^+_Glu-PgTAFIa]*[Glu-Pg:FDPs]*TAFIa)*Plasma) -
((r272.[k^+_Lys-PgFDPs]*[Lys-Pg:FDPs]*TAFIa)*Plasma) -
((r257.[k^+_Glu-PgTAFIa]*TAFIa*[Glu-Pg])*Plasma))
```

ODE-163

```
d(TAFI)/dt = 1/Plasma*(-(([k^F2a_catTAFI]*TAFI*F2a/([k^F2a_mTAFI]+TAFI)+
[k^Pn_catTAFI]*TAFI*Pn/([k^Pn_mTAFI]+TAFI)+
[k^MASP1_catTAFI]*TAFI*MASP1/([k^MASP1_mTAFI]+TAFI))*Plasma))
```

ODE-164

```
d([Glu-Pg])/dt = 1/Plasma*(-(([k^KAL_catGlu-Pg]*[Glu-Pg]*KAL/([k^KAL_mGlu-Pg]+[Glu-Pg])+
[k^tPA_catGlu-Pg]*[Glu-Pg]*tPA/([k^tPA_mGlu-Pg]+[Glu-Pg]))*Plasma) +
((r254.[k^F1a_cattPA]*tPA*F1a/(r254.[k^F1a_mtPA]+tPA))*Plasma) -
(([k^+_Glu-PgFDPs]*[Glu-Pg]*FDPs)*Plasma-([k^-_Glu-PgFDPs]*[Glu-Pg:FDPs])*Plasma) -
((r257.[k^+_Glu-PgTAFIa]*TAFIa*[Glu-Pg])*Plasma) -
(([k^Pn_catGlu-Pg]*[Glu-Pg]*Pn/([k^Pn_mGlu-Pg]+[Glu-Pg]))*Plasma))
```

ODE-165

```
d(tPA)/dt = 1/Plasma*(-((r254.[k^F1a_cattPA]*tPA*F1a/(r254.[k^F1a_mtPA]+tPA))*Plasma) +
(([k^BKB2R_cattPA]*[BK:B2R])*Plasma) - (([k^+_tPAPAI-1]*[PAI-1]*tPA)*Plasma) -
((r252.[k^F1a_cattPA]*tPA*F1a/(r252.[k^F1a_mtPA]+tPA))*Plasma) +
((r249.[k^F1a_cattPA]*[F1a:Lys-Pg])*Plasma))
```

ODE-166

```
d([Lys-Pg])/dt = 1/Plasma*((([k^Pn_catLys-Pg]*Pn)*Plasma) +
((r252.[k^F1a_cattPA]*tPA*F1a/(r252.[k^F1a_mtPA]+tPA))*Plasma) -
(([k^+_Lys-PgTAFIa]*TAFIa*[Lys-Pg])*Plasma) -
(([k^+_F1aLys-Pg]*F1a*[Lys-Pg])*Plasma-
([k^-_F1aLys-Pg]*[F1a:Lys-Pg])*Plasma) -
((r260.[k^+_Lys-PgFDPs]*[Lys-Pg]*FDPs)*Plasma -
([k^-_Lys-PgFDPs]*[Lys-Pg:FDPs])*Plasma) +
(([k^Pn_catGlu-Pg]*[Glu-Pg]*Pn/([k^Pn_mGlu-Pg]+[Glu-Pg]))*Plasma))
```

ODE-167

```
d(CD59)/dt = 1/Plasma*(-(([k^+_CD59MAC]*CD59*[C5b:C6:C7:C8:C9])*Plasma))
```

ODE-168

```
d([P-KAL:F12a])/dt = 1/Plasma*((([k^+_PKALF12a]*F12a*[P-KAL])*Plasma-
([k^-_PKALF12a]*[P-KAL:F12a])*Plasma) -
((r184.[k^F12a_catKAL]*[P-KAL:F12a])*Plasma))
```

ODE-169

```
d([PAI-1])/dt = 1/Plasma*(-(([k^+_tPAPAI-1]*[PAI-1]*tPA)*Plasma))
```

ODE-170

```
d([F1a:Lys-Pg])/dt = 1/Plasma*(-((r249.[k^F1a_cattPA]*[F1a:Lys-Pg])*Plasma) +
(([k^+_F1aLys-Pg]*F1a*[Lys-Pg])*Plasma-([k^-_F1aLys-Pg]*[F1a:Lys-Pg])*Plasma))
```

ODE-171

```
d([P-KAL:HK])/dt = 1/Plasma*(-(([k^PKALHK_catBK]*[P-KAL:HK])*Plasma) +
(([k^+_PKALHK]*HK*[P-KAL]-[k^-_PKALHK]*[P-KAL:HK])*Plasma))
```

ODE-172

```
d([gC1qR:F12])/dt = 1/Plasma*((([k^+_gC1qRF12]*gC1qR*F12)*Plasma-
([k^-_gC1qRF12]*[gC1qR:F12])*Plasma) -
(([k^gC1qRF12_catF12a]*[gC1qR:F12])*Plasma))
```

ODE-173

```
d([HK:F11])/dt = 1/Plasma*((([k^+_HKF11]*HK*F11)*Plasma-
([k^-_HKF11]*[HK:F11])*Plasma) -
(([k^HKF11_catF11a]*[HK:F11])*Plasma))
```

ODE-174

```
d([gC1qR:HK])/dt = 1/Plasma*((([k^+_gC1qRHK]*HK*gC1qR)*Plasma-
([k^-_gC1qRHK]*[gC1qR:HK])*Plasma) -
(([k^gC1qRHK_catBK]*[gC1qR:HK])*Plasma))
```

ODE-175

```
d(PFD)/dt = 1/Plasma*(-(([k^MASP1_catPFD]*PFD*MASP1+
[k^MASP2_catPFD]*PFD*MASP2+[k^F2a_catPFD]*PFD*F2a)*Plasma))
```

ODE-176

```
d(FDPs)/dt = 1/Plasma*(-(([k^+_Glu-PgFDPs]*[Glu-Pg]*FDPs)*Plasma-
([k^-_Glu-PgFDPs]*[Glu-Pg:FDPs])*Plasma) -
((r260.[k^+_Lys-PgFDPs]*[Lys-Pg]*FDPs)*Plasma-
([k^-_Lys-PgFDPs]*[Lys-Pg:FDPs])*Plasma) +
(([k^Pn_catF1a]*F1a*Pn/([k^Pn_mF1a]+F1a))*Plasma))
```

ODE-177

```
d([Glu-Pg:FDPs])/dt = 1/Plasma*((([k^+_Glu-PgFDPs]*[Glu-Pg]*FDPs)*Plasma-
([k^-_Glu-PgFDPs]*[Glu-Pg:FDPs])*Plasma) -
((r273.[k^+_Glu-PgTAFIa]*[Glu-Pg:FDPs]*TAFIa)*Plasma))
```

ODE-178

```
d([Lys-Pg:FDPs])/dt = 1/Plasma*(((r260.[k^+_Lys-PgFDPs]*[Lys-Pg]*FDPs)*Plasma-
([k^-_Lys-PgFDPs]*[Lys-Pg:FDPs])*Plasma) -
((r272.[k^+_Lys-PgFDPs]*[Lys-Pg:FDPs]*TAFIa)*Plasma))
```

ODE-179

```
d(IL-6R)/dt = 1/Plasma*(-(([k^+_IL-6IL-6R]*IL-6*IL-6R)*Plasma - ([k^-_IL-6IL-6R]*[IL-6:IL-6R])*Plasma))
```

```
ODE-180

d([IL-6:IL-6R])/dt = 1/Plasma*((([k^+_IL-6IL-6R]*IL-6*IL-6R)*Plasma - ([k^-_IL-6IL-6R]*[IL-6:IL-6R])*Plasma))
```
