# Supplementary material for: Unravelling the impact of SARS-CoV-2 on hemostatic and complement systems: a systems immunology perspective
Source: Front Immunol. 2025 Jan 13;15:1457324. doi: 10.3389/fimmu.2024.1457324 (PMC11781117; doi:10.3389/fimmu.2024.1457324)
Supplement: Supplementary file 9 [file DataSheet9.pdf]

## *Supplementary Material*

# Ordinary differential equations (ODEs)

```
ODE-5

d(C2)/dt = 1/Plasma*(-(([k^+_C4bC2]*C2*C4b)*Plasma-
([k^-_C4bC2]*[C4b:C2])*Plasma) -
(([k^+_fC3bC4bC2]*[fC3b:C4b]*C2)*Plasma-
([k^-_fC3bC4bC2]*[fC3b:C4b:C2])*Plasma) -
((r75.[k^MASP2_catC2]*C2*MASP2/(r75.[k^MASP2_mC2]+C2)+
[k^C1s_catC2]*C2*C1s/([k^C1s_mC2]+C2))*Plasma)
- (([k^+_HepC2]*Heparin*C2)*Plasma-([k^-_HepC2]*[Hep:C2])*Plasma))
```

```
ODE-8

d(C4)/dt = 1/Plasma*(-((([k^C1_catC4]*C4*C1/(r35.[k^C1_mC4]*
(1+[C4b:C2]/r35.[k^C1_mC2]+
[fC3b:C4b:C2]/r35.[k^C1_mC2]+C4/r35.[k^C1_mC4]))+
[k^MASP1_catC4]*[MBL:MASP1]*C4/(r35.[k^MASP1_mC4]*
(1+[C4b:C2]/r35.[k^MASP1_mC2]+
[fC3b:C4b:C2]/r35.[k^MASP1_mC2]+C4/r35.[k^MASP1_mC4]))+
[k^MASP2_catC4]*[MBL:MASP2]*C4/(r35.[k^MASP2_mC4]*
(1+[C4b:C2]/r35.[k^MASP2_mC2]+
[fC3b:C4b:C2]/r35.[k^MASP2_mC2]+C4/r35.[k^MASP2_mC4])))*Plasma) -
(([k^+_C1sC4]*C4*C1s)*Plasma-([k^-_C1sC4]*[C1s:C4])*Plasma) -
(([k^+_MASP2C4]*MASP2*C4)*Plasma-([k^-_MASP2C4]*[MASP2:C4])*Plasma)
- (([k^+_HepC4]*Heparin*C4)*Plasma-([k^-_HepC4]*[Hep:C4])*Plasma))
```

ODE-13

```
d(C3)/dt = 1/Plasma*(-(([k^C3WBb_catC3]*[C3W:Bb]*C3/([k^C3WBb_mC3]+C3)+
[k^C4bC2a_catC3]*[C4b:C2a]*C3/([k^C4bC2a_mC3]*(1+C3/[k^C4bC2a_mC3]+
C5/[k^C4bC2a_mC5]))+[k^fC3bBb_catC3]*([fC3b:Bb]+[fC3b:Bb:P])*
C3/(r122.[k^fC3bBb_mC3]*(1+C3/r122.[k^fC3bBb_mC3]+C5/r122.[k^fC3bBb_mC5]))
+[k^fC3bBb_catC3]*[fC3b:Bb:C3b]*C3/(r122.[k^fC3bBb_mC3]*
(1+C3/r122.[k^fC3bBb_mC3]+C5/r122.[k^fC3bBbC3b_mC5]))+
[k^C4bC2a_catC3]*[C4b:C2a:C3b]*C3/([k^C4bC2a_mC3]*
(1+C3/[k^C4bC2a_mC3]+C5/[k^C4bC2aC3b_mC5]))+
[k^fC3bBb_catC3]*[fC3b:Bb:C3b:P]*C3/(r122.[k^fC3bBb_mC3]*
(1+C3/r122.[k^fC3bBb_mC3]+C5/r122.[k^fC3bBbC3b_mC5]))+
[k^KAL_catC3]*KAL*C3/([k^KAL_mC3]+C3)+[k^Pn_catC3]*
Pn*C3/([k^Pn_mC3]+C3)+[k^F2a_catC3]*F2a*C3/([k^F2a_mC3]+C3)+
[k^F10a_catC3]*F10a*C3/([k^F10a_mC3]+C3))*Plasma) -
((r124.[k^+_C3W]*C3)*Plasma) + (([k^+_sC3])*Plasma) -
(([k^+_HepC3]*Heparin*C3)*Plasma-
([k^-_HepC3]*[Hep:C3])*Plasma))
```

ODE-52

```
d(C5)/dt = 1/Plasma*(-(([k^fC3bBb_catC5]*[fC3b:Bb]*
C5/(r140.[k^fC3bBb_mC5]*(1+C3/r140.[k^fC3bBb_mC3]+
C5/r140.[k^fC3bBb_mC5]))+[k^fC3bBb_catC5]*[fC3b:Bb:P]*
C5/(r140.[k^fC3bBb_mC5]*(1+C3/r140.[k^fC3bBb_mC3]+
C5/r140.[k^fC3bBb_mC5]))+[k^C4b2a_catC5]*[C4b:C2a]*
C5/([k^C4b2a_mC5]*(1+C3/[k^C4b2a_mC3]+C5/[k^C4b2a_mC5]))+
[k^C4b2a3b_catC5]*[C4b:C2a:C3b]*C5/([k^C4b2a3b_mC5]*
(1+C3/[k^C4b2a_mC3]+C5/[k^C4b2a3b_mC5]))+
[k^fC3bBbC3b_catC5]*([fC3b:Bb:C3b]+[fC3b:Bb:C3b:P])*
C5/(r140.[k^fC3bBbC3b_mC5]*(1+C3/r140.[k^fC3bBb_mC3]+
C5/r140.[k^fC3bBbC3b_mC5]))+[k^KAL_catC5]*
KAL*C5/([k^KAL_mC5]+C5)+[k^Pn_catC5]*Pn*C5/([k^Pn_mC5]+C5)+
[k^F2a_catC5]*F2a*C5/([k^F2a_mC5]+C5)+[k^F10a_catC5]*
F10a*C5/([k^F10a_mC5]+C5))*Plasma) -
(([k^-_dC5]*C5)*Plasma) + (([k^+_sC5])*Plasma) -
(([k^+_HepC5]*Heparin*C5)*Plasma-
([k^-_HepC5]*[Hep:C5])*Plasma))
```

ODE-55

```
d(C6)/dt = 1/Plasma*(-(([k^+_C5bC6]*C5b*C6-[k^-_C5bC6]*[C5b:C6])*Plasma) -
(([k^-_dC6]*C6)*Plasma + (([k^+_sC6])*Plasma) -
(([k^+_HepC6]*Heparin*C6)*Plasma-
([k^-_HepC6]*[Hep:C6])*Plasma))
```

ODE-57

```
d(C7)/dt = 1/Plasma*(-((([k^+_C5bC6C7]*C7*[C5b:C6])*Plasma-
([k^-_C5bC6C7]*[C5b:C6:C7])*Plasma) +
(([k^+_sC7])*Plasma) - (([k^-_dC7]*C7)*Plasma)-
(([k^+_HepC7]*Heparin*C7)*Plasma-
([k^-_HepC7]*[Hep:C7])*Plasma))
```

ODE-59

```
d(C8)/dt = 1/Plasma*(-((([k^+_C5bC6C7C8]*C8*[C5b:C6:C7])*Plasma-
([k^-C5bC6C7C8]*[C5b:C6:C7:C8])*Plasma) -
(([k^-_dC8]*C8)*Plasma) + (([k^+_sC8])*Plasma)-
 (([k^+_HepC8]*Heparin*C8)*Plasma-
([k^-_HepC8]*[Hep:C8])*Plasma))
```

ODE-61

```
d(C9)/dt = 1/Plasma*(-((([k^+_C5bC6C7C8C9]*C9*[C5b:C6:C7:C8])*Plasma-
([k^-_C5bC6C7C8C9]*[C5b:C6:C7:C8:C9])*Plasma) -
(([k^-_dC9]*C9)*Plasma) + (([k^+_sC9])*Plasma)-
(([k^+_HepC9]*Heparin*C9)*Plasma-
([k^-_HepC9]*[Hep:C9])*Plasma))
```

ODE-63

```
d(C5aR1)/dt = 1/Plasma*(-((([k^+_C5aC5aR1]*C5a*C5aR1)*Plasma-
([k^-_C5aC5aR1]*[C5a:C5aR1])*Plasma) -
(([k^+_AvdC5aR1]*Avdoralimab*C5aR1)*Plasma))
```

ODE-115

```
d(AT3)/dt = 1/Plasma*(-(([k^+_F10aAT3]*F10a*AT3)*Plasma) -
(([k^+_F2aAT3]*F2a*AT3)*Plasma) -
(([k^+_F9aAT3]*AT3*F9a)*Plasma) -
(([k^+_TFF7aAT3]*[TF:F7a]*AT3)*Plasma) -
(([k^+_F12aAT3]*AT3*F12a)*Plasma) -
(([k^+_F11aAT3]*AT3*F11a)*Plasma) +
(([k^+_HepAT3]*Heparin*AT3)*Plasma))
```

```
ODE-149

d([KAL:F12])/dt = 1/Plasma*((([k^+_KALF12]*F12*KAL)*Plasma-
([k^-_KALF12]*[KAL:F12])*Plasma) -
(([k^KAL_catF12a]*[KAL:F12])*Plasma) -
 (([k^+_GaradKALF12]*Garadacimab*[KAL:F12])*Plasma))


ODE-165

d(tPA)/dt = 1/Plasma*(-((r254.[k^F1a_cattPA]*
tPA*F1a/(r254.[k^F1a_mtPA]+tPA))*Plasma) +
(([k^BKB2R_cattPA]*[BK:B2R])*Plasma) -
(([k^+_tPAPAI-1]*[PAI-1]*tPA)*Plasma) -
((r252.[k^F1a_cattPA]*
tPA*F1a/(r252.[k^F1a_mtPA]+tPA))*Plasma) +
((r249.[k^F1a_cattPA]*[F1a:Lys-Pg])*Plasma)
- (([k^+_TXAtPA]*TXA*tPA)*Plasma))


ODE-179

d(Heparin)/dt = 1/Plasma*(-(([k^+_HepC4]*Heparin*C4)*Plasma-
([k^-_HepC4]*[Hep:C4])*Plasma) -
(([k^+_HepC2]*Heparin*C2)*Plasma-([k^-_HepC2]*[Hep:C2])*Plasma) -
(([k^+_HepC3]*Heparin*C3)*Plasma-([k^-_HepC3]*[Hep:C3])*Plasma) -
(([k^+_HepC5]*Heparin*C5)*Plasma-([k^-_HepC5]*[Hep:C5])*Plasma) -
(([k^+_HepC6]*C6*Heparin)*Plasma-([k^-_HepC6]*[Hep:C6])*Plasma) -
(([k^+_HepC7]*Heparin*C7)*Plasma-([k^-_HepC7]*[Hep:C7])*Plasma) -
(([k^+_HepC8]*C8*Heparin)*Plasma-([k^-_HepC8]*[Hep:C8])*Plasma) -
(([k^+_HepC9]*C9*Heparin)*Plasma-([k^-_HepC9]*[Hep:C9])*Plasma) -
(([k^+_HepAT3]*Heparin*AT3)*Plasma))


ODE-180

d([Hep:C4])/dt = 1/Plasma*((([k^+_HepC4]*Heparin*C4)*Plasma-
([k^-_HepC4]*[Hep:C4])*Plasma))


ODE-181

d([Hep:C2])/dt = 1/Plasma*((([k^+_HepC2]*Heparin*C2)*Plasma-
([k^-_HepC2]*[Hep:C2])*Plasma))
```

ODE-182

```
d([Hep:C3])/dt = 1/Plasma*((([k^+_HepC3]*Heparin*C3)*Plasma-
([k^-_HepC3]*[Hep:C3])*Plasma))
```

ODE-183

```
d([Hep:C5])/dt = 1/Plasma*((([k^+_HepC5]*Heparin*C5)*Plasma-
([k^-_HepC5]*[Hep:C5])*Plasma))
```

ODE-184

```
d([Hep:C6])/dt = 1/Plasma*((([k^+_HepC6]*C6*Heparin)*Plasma-
([k^-_HepC6]*[Hep:C6])*Plasma))
```

ODE-185

```
d([Hep:C7])/dt = 1/Plasma*((([k^+_HepC7]*Heparin*C7)*Plasma-
([k^-_HepC7]*[Hep:C7])*Plasma))
```

ODE-186

```
d([Hep:C8])/dt = 1/Plasma*((([k^+_HepC8]*C8*Heparin)*Plasma-
([k^-_HepC8]*[Hep:C8])*Plasma))
```

ODE-187

```
d([Hep:C9])/dt = 1/Plasma*((([k^+_HepC9]*C9*Heparin)*Plasma-
([k^-_HepC9]*[Hep:C9])*Plasma))
```

ODE-188

```
d(Garadacimab)/dt = 1/Plasma*(-(([k^+_GaradKALF12]*Garadacimab*[KAL:F12])*Plasma))
```

ODE-189

```
d(TXA)/dt = 1/Plasma*(-(([k^+_TXAtPA]*TXA*tPA)*Plasma))
```

ODE-190

```
d(Avdoralimab)/dt = 1/Plasma*(-(([k^+_AvdC5aR1]*Avdoralimab*C5aR1)*Plasma))
```

ODE-191

```
d(TCZ)/dt = 1/Plasma*(-(([k^+_TCZIL-6R]*TCZ*IL-6R)*Plasma))
```

ODE-192

```
d(IL-6R)/dt = 1/Plasma*(-(([k^+_TCZIL-6R]*TCZ*IL-6R)*Plasma))
```
