# Supplementary material for: Unravelling the impact of SARS-CoV-2 on hemostatic and complement systems: a systems immunology perspective
Source: Front Immunol. 2025 Jan 13;15:1457324. doi: 10.3389/fimmu.2024.1457324 (PMC11781117; doi:10.3389/fimmu.2024.1457324)
Supplement: Supplementary file 10 [file DataSheet10.pdf]

Table S1a. The hemostatic system including coagulation and fibrinolytic entities interaction kinetic parameters.

Meaning	Rate constant	Value adopted	Evidences and References
Association rate for TF and F7<br><br>Disassociation rate for TF:F7	k^{+}_{TFF7}<br><br>k^{-}_{TFF7}	$3.2 \times 10^{6}\ M^{-1}s^{-1}$<br><br>$3.1 \times 10^{-3}\ s^{-1}$	[1], [2].
Association rate for TF and F7a<br><br>Disassociation rate for TF:F7a	k^{+}_{TFF7a}<br><br>k^{-}_{TFF7a}	$2.3 \times 10^{7}\ M^{-1}s^{-1}$<br><br>$3.1 \times 10^{-3}\ s^{-1}$	[1], [2].
Activation of F7 by TF:F7a	k^{TFF7a}_{catF7}	$4.4 \times 10^{5}\ M^{-1}s^{-1}$	[2]
Activation of F7 by F10a	k^{F10a}_{catF7}	$1.3 \times 10^{7}\ M^{-1}s^{-1}$	[2]
Activation of F7 by F2a	k^{F2a}_{catF7}	$2.3 \times 10^{4}\ M^{-1}s^{-1}$	[2]
Association rate for F7a and F9<br><br>Disassociation rate for F7a:F9	k^{+}_{F7aF9}<br><br>k^{-}_{F7aF9}	$1.0 \times 10^{8}\ M^{-1}s^{-1}$<br><br>$0.9\ s^{-1}$	[2]
Production of F9a by F7a	k^{F7a}_{catF9a}	$3.6 \times 10^{-5}\ s^{-1}$	[2]
Association rate for F7a and F10<br><br>Disassociation rate for F7a:F10	k^{+}_{F7aF10}<br><br>k^{-}_{F7aF10}	$1.0 \times 10^{8}\ M^{-1}s^{-1}$<br><br>$210.0\ s^{-1}$	[2]
Production of F10a by F7a	$k^{F7a}_{catF10a}$	$1.6 \times 10^{-6}\ s^{-1}$	[2]
Association rate for TF:F7a and F9<br><br>Disassociation rate for TF:F7a:F9	$k^{+}_{TFF7aF9}$<br><br>$k^{-}_{TFF7aF9}$	$1.0 \times 10^{7}\ M^{-1}s^{-1}$<br><br>$2.4\ s^{-1}$	[2]
Activation of F9 by TF:F7a	k^{TFF7a}_{catF9}	$1.8\ s^{-1}$	[2]

Association rate for TF:F7a and F10<br><br>Disassociation rate for TF:F7a:F10	$k^{+}_{TFF7aF10}$<br><br>$k^{-}_{TFF7aF10}$	$2.5 \times 10^{7}\ M^{-1}s^{-1}$<br><br>$1.05\ s^{-1}$	[2]
Activation of F10 by TF:F7a	k^{TFF7a}_{catF10}	$6.0\ s^{-1}$	[2]
Association rate for TF:F7a and F10a<br><br>Disassociation rate for TF:F7a:F10a	$k^{+}_{TFF7aF10a}$<br><br>$k^{-}_{TFF7aF10a}$	$2.2 \times 10^{7}\ M^{-1}s^{-1}$<br><br>$19.0\ s^{-1}$	[2]
Association rate for F10a and TFPI<br><br>Disassociation rate for F10a:TFPI	$k^{+}_{F10aTFPI}$<br><br>$k^{-}_{F10aTFPI}$	$9.0 \times 10^{5}\ M^{-1}s^{-1}$<br><br>$3.6 \times 10^{-4}\ s^{-1}$	[2]
Binding of TF:F7a:F10a and TFPI<br><br>Disassociation of TF:F7a:F10a	$k^{+}_{TFF7aF10aTFPI}$<br><br>$k^{-}_{TFF7aF10aTFPI}$	$3.2 \times 10^{8}\ M^{-1}s^{-1}$<br><br>$1.1 \times 10^{-4}\ s^{-1}$	[2]
Binding of F10a:TFPI and TF:F7a	$k^{+}_{F10aTFPITFF7a}$	$5.0 \times 10^{7}\ M^{-1}s^{-1}$	[2]
Association rate for F11a and F9<br><br>Disassociation rate for F11a:F9	k^{+}_{F11aF9}<br><br>k^{-}_{F11aF9}	$1.0 \times 10^{8}\ M^{-1}s^{-1}$<br><br>$41.0\ s^{-1}$	[2], [3].
Generation of F9a by F11a	k^{F11a}_{catF9a}	$7.7\ s^{-1}$	F11a can generate F9a [2], [3].
Inhibition of F11a by AT3	$k^{+}_{F11aAT3}$	$3.2 \times 10^{2}\ M^{-1}s^{-1}$	Destruction of F11a by inhibitor AT3 [2], [3].
Binding of F12a and F11<br><br>Disassociation of F12a:F11	$k^{+}_{F12aF11}$<br><br>$k^{-}_{F12aF11}$	$1.0 \times 10^{8}\ M^{-1}s^{-1}$<br><br>$200.0\ s^{-1}$	[2], [3].
Production of F11a by F12a	$k^{F12a}_{catF11a}$	$5.7 \times 10^{-3}\ s^{-1}$	F12a can produce F11a [3].
Binding of F12 and F12a<br><br>Disassociation of F12:F12a	$k^{+}_{F12F12a}$<br><br>$k^{-}_{F12F12a}$	$1.0 \times 10^{8}\ M^{-1}s^{-1}$<br><br>$750.0\ s^{-1}$	[2], [3].

Autocatalysis of F12a amplification by F12:F12a	$k_{catF12a}^{F12F12a}$	$3.3 \times 10^{-2}\ s^{-1}$	The product of F12 and F12a autocatalysis of F12a and increase it expression level [2], [3].
Association rate for P-KAL and F12a<br><br>Disassociation rate for P-KAL:F12a	$k_{PKALF12a}^{+}$<br><br>$k_{PKALF12a}^{-}$	$1.0 \times 10^{8}\ M^{-1}s^{-1}$<br><br>$3.6 \times 10^{3}\ s^{-1}$	Binding of P-KAL with F12 and disassociation of their product P-KAL:F12 [2], [3].
Association rate for KAL and F12<br><br>Disassociation rate for KAL:F12	k_{KALF12}^{+}<br><br>k_{KALF12}^{-}	$1.0 \times 10^{8}\ M^{-1}s^{-1}$<br><br>$45.3\ s^{-1}$	Binding of KAL with F12 and disassociation of their product KAL:F12 [2], [3].
Production of F12a by KAL	$k_{catF12a}^{KAL}$	$5.7\ s^{-1}$	[2], [3].
Association rate for F9a and F10<br><br>Disassociation rate for F9a:F10	k_{F9aF10}^{+}<br><br>k_{F9aF10}^{-}	$1.0 \times 10^{8}\ M^{-1}s^{-1}$<br><br>$0.64\ s^{-1}$	[2], [3].
Production of F10a by F9a	$k_{catF10a}^{F9a}$	$7.0 \times 10^{-4}\ s^{-1}$	[2], [3].
Association rate for F9a and F8a<br><br>Disassociation rate for F9a:F8a	k_{F9aF8a}^{+}<br><br>k_{F9aF8a}^{-}	$1.0 \times 10^{7}\ M^{-1}s^{-1}$<br><br>$5.0 \times 10^{-3}\ s^{-1}$	[2], [3].
Binding of F9a and AT3	k_{F9aAT3}^{+}	$4.9 \times 10^{2}\ M^{-1}s^{-1}$	Inhibition of F9a by AT3 [2], [3].
Association rate for F9a:F8a and F10<br><br>Disassociation rate for F9a:F8a:F10	$k_{F9aF8aF10}^{+}$<br><br>$k_{F9aF8aF10}^{-}$	$1.0 \times 10^{8}\ M^{-1}s^{-1}$<br><br>$1.0 \times 10^{-3}\ s^{-1}$	[2], [3].
Activation of F10 by F9a:F8a	k_{catF10}^{F9aF8a}	$8.2\ s^{-1}$	[2], [3].
Association rate for F10a and F8<br><br>Disassociation rate for F10a:F8	k_{F10aF8}^{+}<br><br>k_{F10aF8}^{-}	$1.0 \times 10^{8}\ M^{-1}s^{-1}$<br><br>$2.1\ s^{-1}$	[2], [3].
Production of F8a by F10a	k_{catF8a}^{F10a}	$2.3 \times 10^{-2}\ s^{-1}$	F10a produce F8a [2], [3].

Binding of F10a and F5a<br><br>Disassociation of F10a:F5a	$k^{+}_{F10aF5a}$<br><br>$k^{-}_{F10aF5a}$	$4.0 \times 10^{8}\ M^{-1}s^{-1}$<br><br>$0.2\ s^{-1}$	[2], [3].
Inhibition of F0a by AT3	$k^{+}_{F10aAT3}$	$1.5 \times 10^{3}\ M^{-1}s^{-1}$	Binding of F10a and AT3 [2], [3].
Binding of F10a:F5a and F2<br><br>Disassociation of F10a:F5a:F2	$k^{+}_{F10aF5aF2}$<br><br>$k^{-}_{F10aF5aF2}$	$1.0 \times 10^{8}\ M^{-1}s^{-1}$<br><br>$103.0\ s^{-1}$	[2], [3].
Activation of F2 by F10a	k^{F10a}_{catF2}	$7.5 \times 10^{3}\ M^{-1}s^{-1}$	Binding of F10a and F2 [2], [3].
Inhibition of F2a by A2M	k^{+}_{F2aA2M}	$2.5 \times 10^{3}\ M^{-1}s^{-1}$	Association rate of A2M and F2a [4].
Association rate for TF:F7a and AT3	$k^{+}_{TFF7aAT3}$	$2.3 \times 10^{2}\ M^{-1}s^{-1}$	Inhibition of TF:F7a by AT3 [2]
Binding of F11 and F2a<br><br>Disassociation of F11:F2a	k^{+}_{F11F2a}<br><br>k^{-}_{F11F2a}	$1.0 \times 10^{8}\ M^{-1}s^{-1}$<br><br>$5.0\ s^{-1}$	[2], [3].
Generation of F11a	k^{F11}_{catF2a}	$1.3 \times 10^{-4}\ s^{-1}$	[2], [3].
Inhibition of F2a by AT3	k^{+}_{F2aAT3}	$7.1 \times 10^{3}\ M^{-1}s^{-1}$	Binding of AT3 and F2a [2], [3].
Activation of F5 by F2a	k^{F2a}_{catF5}	$2.0 \times 10^{7}\ M^{-1}s^{-1}$	[2], [3].
Binding of F2a and F1<br><br>Disassociation of F2a:F1	k^{+}_{F2aF1}<br><br>k^{-}_{F2aF1}	$1.17 \times 10^{7}\ M^{-1}s^{-1}$<br><br>$84.0\ s^{-1}$	[5], [6].
Production of F1a	k^{F2a}_{catF1a}	$84\ \ s^{-1}$	[5], [6].
Activation of F8 by F2a	k^{F2a}_{catF8}	$2.0 \times 10^{7}\ M^{-1}s^{-1}$	F2a binds with F8 [2], [3].
Inhibation of F12a by AT3	$k^{+}_{F12aAT3}$	$21.6\ M^{-1}s^{-1}$	AT3 is associated with F12a and inhibits it [2], [3].
Production of KAL by F12a	k^{F12a}_{catKAL}	$40\ \ s^{-1}$	F12a can generates KAL) [2], [3].

Activation of KAL by P-KAL	$k^{+}_{KALPKAL}$	$2.7 \times 10^{4}\ M^{-1}s^{-1}$	KAL auto-activation [2], [3].
Generation of Lys-Pg by Glu-Pg	$k^{Glu-Pg}_{catLys-Pg}$	$0.1\ s^{-1}$	Pg variants are Lys-Pg and Glu-Pg [7]. Lys-Pg can be generated by Pn hydrolysis of Glu-Pg [8]. Conversion of Glu- to Lys-Lys-Pg [9]–[11]. In literature the generation rate of Lys-Pg not available. Assume a small value for it.
Activation of Glu-Pg by tPA	k^{F1a}_{cattPA}<br><br>k^{F1a}_{mtPA}	$4.1 \times 10^{-7}\ s^{-1}$<br><br>0.073 M	tPA activates Glu-Pg in the presence of F1a [8].
Activation of Lys-Pg by tPA	k^{F1a}_{cattPA}<br><br>k^{F1a}_{mtPA}	$2.0 \times 10^{-8}\ s^{-1}$<br><br>0.064 M	tPA activates Lys-Pg in the presence of F1a [8].
Association rate for Glu-Pg and FDPs<br><br>Disassociation rate for Glu-Pg:FDPs	$k^{+}_{Glu-PgFDPs}$<br><br>$k^{-}_{Glu-PgFDPs}$	$5.71 \times 10^{5}\ M^{-1}s^{-1}$<br><br>$0.1\ s^{-1}$	Native Plasminogen (Glu-Pg) binds to FDPs with affinity ($k_d = 175 \times 10^{-9}$M) [12]. The Association rate (k^{+}) can be computed from the formula $k_d = \frac{k^{-}}{k^{+}}$. Assume a small value for $k^{-} = 0.1s^{-1}$.
Association rate for Lys-Pg and FDPs<br><br>Disassociation rate for Lys-Pg:FDPs	$k^{+}_{Lys-PgFDPs}$<br><br>$k^{-}_{Lys-PgFDPs}$	$1.11 \times 10^{6}\ M^{-1}s^{-1}$<br><br>$0.1\ s^{-1}$	Lys-Pg binds to FDPs with affinity ($k_d = 90 \times 10^{-9}$M) [12]. The Association rate (k^{+}) can be computed from the formula $k_d = \frac{k^{-}}{k^{+}}$. Assume a small value for $k^{-} = 0.1s^{-1}$.
Inhibition of Glu-Pg:FDPs by TAFIa	$k^{+}_{Glu-PgFDPsTAFIa}$	$1.7133 \times 10^{7}\ M^{-1}s^{-1}$	Glu-Pg is degraded by TAFIa in the presence of FDPs. Treatment of the FDPs with TAFIa removes Glu-Pg binding sites. When FDPs are treated with TAFIa the k_d increases to 1×10^{-6} M, $k_{cat} = 2.35\ s^{-1}$, $k_m = 0.143\mu$M [12]. The Association rate (k^{+}) can be computed from the formula $k^{+} = \frac{k^{-} + k_{cat}}{k_m}$. Assume a small value for $k^{-} = 0.1s^{-1}$.
Inhibition of Lys-Pg:FDPs by TAFIa	$k^{+}_{Lys-PgFDPsTAFIa}$	$1.051 \times 10^{7}\ M^{-1}s^{-1}$	Lys-Pg is degraded by TAFIa in the presence of FDPs. Treatment of the FDPs with TAFIa removes Glu-Pg binding sites. When FDPs are treated with TAFIa the k_d increases to 1.55×10^{-6}M, $k_{cat} = 0.888\ s^{-1}$, $k_m = 0.094\mu$M [12]. The Association rate (k^{+}) can be computed from the formula $k^{+} = \frac{k^{-} + k_{cat}}{k_m}$. Assume a small value for $k^{-} = 0.1s^{-1}$.

Association rate for F1a and Lys-Pg<br><br>Disassociation rate for F1a:Lys-Pg	$k^{+}_{F1aLys-Pg}$<br><br>$k^{-}_{F1aLys-Pg}$	$1.0 \times 10^{-7}\ M^{-1}s^{-1}$<br><br>$3.8\ s^{-1}$	Lys-Pg binds to F1a [7], [8]. The binding rate constant for Pg to F1 and the unbinding rate constant for Pg from F1 [13].
Activation of F1 by Lys-Pg	k^{Lys-Pg}_{catF1}	$1.0 \times 10^{-7}\ M^{-1}s^{-1}$	Lys-Pg can binds to F1a, the binding rate measured $1.0 \times 10^{-7}\ M^{-1}s^{-1}$ [7], [8]. Assumption for activation rate of F1 to F1a by Lys-Pg.
Inhibition of tPA by PAI-1	$k^{+}_{tPAPAI-1}$	$2 \times 10^{7}\ M^{-1}s^{-1}$	PAI-1 is an efficient inhibitor of tPA [14].
Production of tPA by F1a:Lys-Pg	$k^{F1aLys-Pg}_{cattPA}$	$0.1\ s^{-1}$	Assumption for the rate of tPA production by F1a: Lys-Pg, as in propagation phase of fibrinolysis, F1a can generates tPA in the presence of Lys-Pg [15].
Generation of Lys-Pg by Pn	$k^{Pn}_{catLys-Pg}$	$0.1\ s^{-1}$	Assumed a small value for the rate of Lys-Pg production by Pn. As in propagation phase of fibrinolysis, Lys-Pg can be produced by Pn [15].
Association rate for P-KAL and HK<br><br>Disassociation rate for P-KAL:HK	k^{+}_{PKALHK}<br><br>k^{-}_{PKALHK}	$8.3 \times 10^{7}\ M^{-1}s^{-1}$<br><br>$1s^{-1}$	P-KAL binds to HK with high specificity and affinity ($k_d = 1.2 \times 10^{-8}$M) [16]. The Association rate (k^{+}) can be computed from the formula $k_d = \frac{k^{-}}{k^{+}}$. Assumed a small value for $k^{-} = 1s^{-1}$.
Activation of HK by KAL	k^{KAL}_{catHK}	$8.3 \times 10^{7}\ M^{-1}s^{-1}$	Estimated based on association rate of P-KAL and HK.
Generation of BK by P-KAL:HK	k^{PKALHK}_{catBK}	$0.1\ s^{-1}$	The product of P-KAL and HK can release BK [17]. The generation rate is unknown, estimated a small value for it.
Association rate for BK and B2R<br><br>Disassociation rate for BK:B2R	k^{+}_{BKB2R}<br><br>k^{-}_{BKB2R}	$3.4 \times 10^{8} M^{-1}s^{-1}$<br><br>$1\ s^{-1}$	BK binds to B2R with a high affinity ($k_d = 2.90 \times 10^{-9}$) [18]. The disassociation rate (k^{-1}) is unknown, assumed a small value for $k^{-1} = 1\ s^{-1}$. Using the formula $k_d = \frac{k^{-}}{k^{+}}$, determined the association rate (k^{+}).
Association rate for HK and F11<br><br>Disassociation rate for HK:F11	k^{+}_{HKF11}<br><br>k^{-}_{HKF11}	$0.40 \times 10^{7} M^{-1}s^{-1}$<br><br>$1\ s^{-1}$	F11 can binds to HK with an affinity $k_d = (2-3) \times 10^{-7}$M [19], [20]. Considered $k_d = 2.5 \times 10^{-7}$M. The disassociation rate (k^{-1}) is unknown, assumed $k^{-1} = 1\ s^{-1}$. Using the formula $k_d = \frac{k^{-}}{k^{+}}$, determined the association rate (k^{+}).
Production of F11a by HK:F11	$k^{HKF11}_{catF11a}$	$0.1\ s^{-1}$	The association of HK and F11 can generate F11a. Assumed, a small value for the production of F11a by product of HK and F11.

Production of tPA by BK:B2R	k_{cattPA}^{BKB2R}	$0.1\ s^{-1}$	The product of BK:B2R stimulates tPA [20]. Assumed, a small value for the production of tPA by BK: B2R.
Activation of the F1 by F2a	k_{catF1}^{F2a}<br><br>k_{mF1}^{F2a}	$59\ s^{-1}$<br><br>3.16×10^{-6}M	Enzyme reactions between F2a and F1 [1].
Cleavage of TAFI by Pn	$k_{catTAFI}^{Pn}$<br><br>k_{mTAFI}^{Pn}	$4.0 \times 10^{-4} s^{-1}$<br><br>$5.5 \times 10^{-8}$M	Pn cleaves TAFI at Arg92, generating TAFIa. Pn-mediated TAFI activation [9].
Cleavage of TAFI by F2a	$k_{catTAFI}^{F2a}$<br><br>k_{mTAFI}^{F2a}	$0.17\ s^{-1}$<br><br>8.3×10^{-7}M	F2a can cleaves TAFI and generates active TAFIa [21].
Inhibition of Glu-Pg by TAFIa	$k_{Glu-PgTAFIa}^{+}$	$1.69 \times 10^{7} M^{-1} s^{-1}$	TAFIa inhibits the activation of Glu-Pg and the conversion of Glu- to Lys-Pg [9]–[11]. $k_{cat} = 2.30\ s^{-1}$, k_m= 0.142μM [22], [23]. The disassociation rate (k^-) is unknown, assumed a small value for $k^- = 0.1 s^{-1}$. Using the formula $k^+ = \frac{k^- + k_{cat}}{k_m}$, determined the association rate (k^+).
Inhibition of Lys-Pg by TAFIa	$k_{Lys-PgTAFIa}^{+}$	$1.051 \times 10^{7}\ M^{-1} s^{-1}$	Assumption for association rate of Lys-Pg and TAFIa based on inhibition of Lys-Pg:FDPs by TAFIa.
Inhibition of C3a by TAFIa	$k_{C3aTAFIa}^{+}$	$2.36 \times 10^{5} M^{-1} s^{-1}$	TAFIa can inhibit C3a. $k_{cat} = 8.4\ s^{-1}$, k_m= 35.9μM [22], [23]. Assume a small value for $k^- = 0.1 s^{-1}$. Using the formula $k^+ = \frac{k^- + k_{cat}}{k_m}$, determined the association rate (k^+).
Inhibition of C5a by TAFIa	$k_{C5aTAFIa}^{+}$	$1.35 \times 10^{5} M^{-1} s^{-1}$	TAFIa can inhibit C5a. $k_{cat} = 29.5\ s^{-1}$, k_m= 219.0 μM [22], [23]. Assume a small value for $k^- = 0.1 s^{-1}$. Using the formula $k^+ = \frac{k^- + k_{cat}}{k_m}$, determined the association rate (k^+).
Inhibition of BK by TAFIa	$k_{BKTAFIa}^{+}$	$2.8 \times 10^{5} M^{-1} s^{-1}$	TAFIa can inactivate BK. $k_{cat} = 19.7\ s^{-1}$, k_m= 70.6 μM [22], [23]. Assume a small value for $k^- = 0.1 s^{-1}$. Using the formula $k^+ = \frac{k^- + k_{cat}}{k_m}$, determined the association rate (k^+).

Activation of F12 by Pn	k_{catF12}^{Pn}	$1.0 \times 10^{3} M^{-1} s^{-1}$	Pn can cleave and activate F12 [24]. Estimated a small value for activation of F12 by Pn.
Cleavage of F1 by Pn	k_{catF1}^{Pn}<br><br>k_{mF1}^{Pn}	$7.1\ s^{-1}$<br><br>$6.5 \times 10^{-6} M$	Pn can activates and cleaves F1 [25].
Cleavage of F1a by Pn	k_{catF1a}^{Pn}<br><br>k_{mF1a}^{Pn}	$6.4\ s^{-1}$<br><br>$0.14 \times 10^{-6} M$	Degradation of F1a by Pn which cleaved F1a [26].
Cleavage of Glu-Pg by Pn	$k_{catGlu-Pg}^{Pn}$<br><br>$k_{mGlu-Pg}^{Pn}$	$0.1\ s^{-1}$<br><br>$1.0 \times 10^{-4} M$	Plasmin also enhances fibrinolysis by converting native Pg (Glu-Pg) to Lys-Pg. Pn cleaves Glu-Pg at lysine 77/78 result in Lys-Pg generation [12]. Assumption for the cleavage of Glu-Pg by Pn.
Cleavage of Glu-Pg by KAL	$k_{catGlu-Pg}^{KAL}$<br><br>$k_{mGLu-Pg}^{KAL}$	$1.6 \times 10^{-4}\ s^{-1}$<br><br>$5.6 \times 10^{-7} M$	[27]
Cleavage of Glu-Pg by tPA	$k_{catGlu-Pg}^{tPA}$<br><br>$k_{mGlu-Pg}^{tPA}$	$4.1 \times 10^{-7}\ s^{-1}$<br><br>0.073 M	[8]

Table S1b. The complement system entities interaction kinetic parameters

Meaning	Rate constant	Value adopted	Evidences and References
Binding rate for C1r and C1s<br><br>Disassociation rate for C1r:C1s	k^{+}_{C1rC1s}<br><br>k^{-}_{C1rC1s}	$0.61 \times 10^{6}\ M^{-1}s^{-1}$<br><br>$3.44 \times 10^{-3}\ s^{-1}$	Association and disassociation rates of C1r and C1s are measured with respect to different modules such as x-ray structure of the N-terminal CUB-epidermal growth factor (EGF) marked by different residues. Among the high affinity sites at $C1rCUB_1$–Y56A, binding rates and disassociation rates measured as $k^{+} = 0.61 \times 10^{6}\ M^{-1}s^{-1}$ and $k^{-} = 3.44 \times 10^{-3}\ s^{-1}$ [28].
Binding rate for C1q and C1r:C1s<br><br>Disassociation rate for C1q :C1r:C1s	k^{+}_{C1qrs}<br><br>k^{-}_{C1qrs}	$0.82 \times 10^{6}\ M^{-1}s^{-1}$<br><br>$1.2 \times 10^{-3}\ s^{-1}$	[28]–[31].
Binding rate for C1s and C4<br><br>Disassociation rate for C1s:C4	k^{+}_{C1sC4}<br><br>k^{-}_{C1sC4}	$7.9 \times 10^{5}\ M^{-1}s^{-1}$<br><br>$4.8 \times 10^{-1}\ s^{-1}$	[32].
Binding rate for MASP2 and C4<br><br>Disassociation rate for MASP2:C4	$k^{+}_{MASP2C4}$<br><br>$k^{-}_{MASP2C4}$	$7.9 \times 10^{5}\ M^{-1}s^{-1}$<br><br>$4.8 \times 10^{-1}\ s^{-1}$	Estimation based on MASP2 functionally homologous of C1q [32], [33].
Binding rate for C3W and FB<br><br>Disassociation rate for C3W:FB	k^{+}_{C3WB}<br><br>k^{-}_{C3WB}	$1.1 \times 10^{4} M^{-1}s^{-1}$<br><br>$1.4 \times 10^{-3} s^{-1}$	[30], [31], [34].
Binding rate for C3W and Factor H (FH)<br><br>Disassociation rate for C3W:FH	k^{+}_{C3WH}<br><br>k^{-}_{C3WH}	$1.1 \times 10^{6} M^{-1}s^{-1}$<br><br>$6.0 \times 10^{-2} s^{-1}$	Estimation structurally/functionally homologous protein C3b and FH [30], [31], [34].
Binding rate for fC3b and FB<br><br>Disassociation rate for fC3b:FB	k^{+}_{fC3bFB}<br><br>k^{-}_{fC3bFB}	$21.3 \times 10^{4} M^{-1}s^{-1}$<br><br>$15.5 \times 10^{-2} s^{-1}$	[29]–[31].

Binding rate for fC3b:C4b:P and FB<br><br>Disassociation rate for fC3b:C4b:P:FB	$k^{+}_{fC3bC4bPFB}$<br><br>$k^{-}_{fC3bC4bPFB}$	$21.3 \times 10^{4} M^{-1}s^{-1}$<br><br>$15.5 \times 10^{-2} s^{-1}$	[29]–[31], [35].
Binding rate for lgG:fC3b and FB<br><br>Disassociation rate for lgG:fC3b:FB	$k^{+}_{lgGfC3bFB}$<br><br>$k^{-}_{lgGfC3bFB}$	$21.3 \times 10^{4} M^{-1}s^{-1}$<br><br>$15.5 \times 10^{-2} s^{-1}$	Estimation functionally homologous protein fC3b and FB.
Binding rate for C3b and P<br><br>Disassociation rate for C3b:P	k^{+}_{C3bP}<br><br>k^{-}_{C3bP}	$1.5 \times 10^{5} M^{-1}s^{-1}$<br><br>$15.5 \times 10^{-5} s^{-1}$	[29]–[31], [36].
Binding rate for fC3b:C4b and P<br><br>Disassociation rate for fC3b:C4b:P	$k^{+}_{fC3bC4bP}$<br><br>$k^{-}_{fC3bC4bP}$	$1.5 \times 10^{5} M^{-1}s^{-1}$<br><br>$15.5 \times 10^{-5} s^{-1}$	[29]–[31].
Binding rate for C3b and FH<br><br>Disassociation rate for C3b:FH	k^{+}_{C3bFH}<br><br>k^{-}_{C3bFH}	$1.1 \times 10^{6} M^{-1}s^{-1}$<br><br>$5.9 \times 10^{-2} s^{-1}$	[29]–[31], [37].
Binding rate for fC3b:Bb and FH<br><br>Disassociation rate for fC3b:Bb:FH	$k^{+}_{fC3bBbFH}$<br><br>$k^{-}_{fC3bBbFH}$	$1.1 \times 10^{6} M^{-1}s^{-1}$<br><br>$5.9 \times 10^{-2} s^{-1}$	Estimation functionally homologous protein C3b and FH.
Binding rate for C3b:Bb:C3b and FH<br><br>Disassociation rate for C3b:Bb:C3b:FH	$k^{+}_{C3bBbC3bFH}$<br><br>$k^{-}_{C3bBbC3bFH}$	$1.1 \times 10^{6} M^{-1}s^{-1}$<br><br>$5.9 \times 10^{-2} s^{-1}$	Estimation functionally homologous protein C3b and FH.
Binding rate for C4b and C4BP<br><br>Disassociation rate for C4b:C4BP	$k^{+}_{C4bC4BP}$<br><br>$k^{-}_{C4bC4BP}$	$2.0 \times 10^{5} M^{-1}s^{-1}$<br><br>$1.6 \times 10^{-2} s^{-1}$	[29]–[31], [38]–[40].
Binding rate for C4b:C2a and C4BP<br><br>Disassociation rate for C4b:C2a:C4BP	$k^{+}_{C4bC2aC4BP}$<br><br>$k^{-}_{C4bC2aC4BP}$	$2.0 \times 10^{5} M^{-1}s^{-1}$<br><br>$1.6 \times 10^{-2} s^{-1}$	Estimation functionally homologous protein C4b and C4BP.

Binding rate for C3b and CR1<br><br>Disassociation rate for C3b:CR1	k^{+}_{C3bCR1}<br><br>k^{-}_{C3bCR1}	$4.4 \times 10^{6}\ M^{-1}s^{-1}$<br><br>$5.7 \times 10^{-2}\ s^{-1}$	[29]–[31], [37].
Binding rate for C3b:Bb and CR1<br><br>Disassociation rate for C3b:Bb:CR1	$k^{+}_{C3bBbCR1}$<br><br>$k^{-}_{C3bBbCR1}$	$4.4 \times 10^{6}\ M^{-1}s^{-1}$<br><br>$5.7 \times 10^{-2}\ s^{-1}$	Estimation functionally homologous protein C3b and CR1.
Binding rate for fC3b:Bb:C3b and CR1<br><br>Disassociation rate for fC3b:Bb:C3b:CR1	$k^{+}_{fC3bBbC3bCR1}$<br><br>$k^{-}_{fC3bBbC3bCR1}$	$9.8 \times 10^{4}\ M^{-1}s^{-1}$<br><br>$2.1 \times 10^{-3}\ s^{-1}$	[29]–[31], [37].
Binding rate for fC3b:Bb and DAF<br><br>Disassociation rate for fC3b:Bb:DAF	$k^{+}_{fC3bBbDAF}$<br><br>$k^{-}_{fC3bBbDAF}$	$1.4 \times 10^{3}\ M^{-1}s^{-1}$<br><br>$1.2 \times 10^{-3}\ s^{-1}$	[29]–[31], [41].
Binding rate for C4b:C2a and DAF<br><br>Disassociation rate for C4b:C2a:DAF	$k^{+}_{C4bC2aDAF}$<br><br>$k^{-}_{C4bC2aDAF}$	$1.4 \times 10^{3}\ M^{-1}s^{-1}$<br><br>$1.2 \times 10^{-3}\ s^{-1}$	Estimation functionally homologous protein C3 convertase (fC3b:Bb) and DAF.
Binding rate for C4b:C2a:C3b and DAF<br><br>Disassociation rate for C4b:C2a:C3b:DAF	$k^{+}_{C4bC2aC3bDAF}$<br><br>$k^{-}_{C4bC2aC3bDAF}$	$1.4 \times 10^{3}\ M^{-1}s^{-1}$<br><br>$1.2 \times 10^{-3}\ s^{-1}$	Estimation functionally homologous protein C3 convertase (C4b:C2a) and DAF.
Binding rate for fC3b:Bb:C3b and DAF<br><br>Disassociation rate for fC3b:Bb:C3b:DAF	$k^{+}_{fC3bBbC3bDAF}$<br><br>$k^{-}_{fC3bBbC3bDAF}$	$1.4 \times 10^{3}\ M^{-1}s^{-1}$<br><br>$1.2 \times 10^{-3}\ s^{-1}$	Estimation functionally homologous protein C3 convertase (fC3b:Bb) and DAF.
Binding rate for C4b:C2a and CR1<br><br>Disassociation rate for C4b:C2a:CR1	$k^{+}_{C4bC2aCR1}$<br><br>$k^{-}_{C4bC2aCR1}$	$3.8 \times 10^{6}\ M^{-1}s^{-1}$<br><br>$4.2 \times 10^{-2}\ s^{-1}$	[29]–[31].
Binding rate for C3W:Bb and FH<br><br>Disassociation rate for C3W:Bb:FH	$k^{+}_{C3WBbFH}$<br><br>$k^{-}_{C3WBbFH}$	$1.1 \times 10^{6}\ M^{-1}s^{-1}$<br><br>$5.9 \times 10^{-2}\ s^{-1}$	Estimation functionally homologous protein C3b and FH.
Binding rate for C5b and C6<br><br>Disassociation rate for C5b:C6	k^{+}_{C5bC6}<br><br>k^{-}_{C5bC6}	$6.0 \times 10^{4}\ M^{-1}s^{-1}$<br><br>$9.0 \times 10^{-8}\ s^{-1}$	[29]–[31], [38], [42].

Binding rate for C5b:C6 and C7<br><br>Disassociation rate for C5b:C6:C7	$k^{+}_{C5bC6C7}$<br><br>$k^{-}_{C5bC6C7}$	$7.3 \times 10^{5}\ M^{-1}s^{-1}$<br><br>$1.5 \times 10^{-7}\ s^{-1}$	[29]–[31], [38], [42].
Binding rate for C5b:C6:C7 and C8<br><br>Disassociation rate for C5b:C6:C7:C8	$k^{+}_{C5bC6C7C8}$<br><br>$k^{-}_{C5bC6C7C8}$	$1.1 \times 10^{6}\ M^{-1}s^{-1}$<br><br>$9.8 \times 10^{-7}\ s^{-1}$	[29]–[31], [38], [42].
Binding rate for C5b:C6:C7:C8 and C9<br><br>Disassociation rate for C5b:C6:C7:C8:C9	$k^{+}_{C5bC6C7C8C9}$<br><br>$k^{-}_{C5bC6C7C8C9}$	$2.8 \times 10^{6}\ M^{-1}s^{-1}$<br><br>$2.8 \times 10^{-6}\ s^{-1}$	[29]–[31], [38], [42].
Association of fC3b to H_2O	k^{+}_{fC3b}	$4.2 \times 10^{8}\ M^{-1}s^{-1}$	[29]–[31].
Binding rate for fC3b and IgG	$k^{+}_{IgGfC3b}$	$4.2 \times 10^{8}\ M^{-1}s^{-1}$	[29]–[31].
Activation rate of C1	k^{+}_{C1}	$2.1 \times 10^{-5}\ s^{-1}$	[29]–[31].
Binding rate for C1 and inhibitor C1INH	$k^{+}_{C1C1INH}$	$4.3 \times 10^{5}\ M^{-1}s^{-1}$	[29]–[31], [43].
Binding rate of C1INH and MASP1	$k^{+}_{C1INHMASP1}$	$6.3 \times 10^{3}\ M^{-1}s^{-1}$	[14], [29]–[31], [44].
Binding rate of C1INH and MASP2	$k^{+}_{C1INHMASP2}$	$2.2 \times 10^{7}\ M^{-1}s^{-1}$	[29]–[32].
Disassociation of initial C3-convertase C3W:Bb<br>Disassociation of C3-convertase fC3b:Bb	k^{-}_{C3WBb}<br><br>k^{-}_{fC3bBb}	$9.0 \times 10^{-3}\ s^{-1}$<br><br>$7.70 \times 10^{-3}\ s^{-1}$	Estimation is based on the increases in decay rate of fC3b:Bb. The enzyme C3W:Bb is less active and less stable compared to C3bBb [29]–[31], [43].
Binding rate for C4b and C2<br><br>Disassociation rate for C4b:C2	k^{+}_{C4bC2}<br><br>k^{-}_{C4bC2}	$1.6 \times 10^{6}\ M^{-1}s^{-1}$<br><br>$4.2 \times 10^{-3}\ s^{-1}$	[29]–[31], [34].
Binding rate for fC3b:C4b and C2<br><br>Disassociation rate for fC3b:C4b:C2	$k^{+}_{fC3bC4bC2}$<br><br>$k^{-}_{fC3bC4bC2}$	$1.6 \times 10^{6}\ M^{-1}s^{-1}$<br><br>$4.2 \times 10^{-3}\ s^{-1}$	[29]–[31].

Binding rate for C4b and C2a<br><br>Disassociation rate for C4b:C2a	k^{+}_{C4bC2a}<br><br>k^{-}_{C4bC2a}	$4.4 \times 10^{3}\ M^{-1}s^{-1}$<br><br>$5.8 \times 10^{-3}\ s^{-1}$	In Quasi steady-state approximation (QSSA), rapid equilibrium leads to $k_m \approx k_d$,<br>QSSA leads to $k_m \approx \frac{k_{cat}}{k^{+}}$ [45].<br>$\frac{k_{cat}}{k_m}$ = 4.4× $10^{3}\ M^{-1}s^{-1}$ [46] ,<br>k^{-} = 5.8× $10^{-3}s^{-1}$ [38].
Binding rate for C4b:C2a and C3b<br><br>Disassociation of C5-convertase C4b:C2a:C3b	$k^{+}_{C4bC2aC3b}$<br><br>$k^{-}_{C4bC2aC3b}$	$1.5 \times 10^{7}\ M^{-1}s^{-1}$<br><br>$5.0 \times 10^{-3}\ s^{-1}$	In Quasi steady-state approximation (QSSA), rapid equilibrium leads to $k_m \approx k_d$,<br>QSSA leads to $k_m \approx \frac{k_{cat}}{k^{+}}$ [45].<br>$\frac{k_{cat}}{k_m}$ = 1.5× $10^{7}\ M^{-1}s^{-1}$ [46] ,<br>k^{-} = 5.0× $10^{-3}s^{-1}$ [30].
Binding rate for fC3b and C3b:FB<br><br>Disassociation of fC3b:C3b:FB	$k^{+}_{fC3bC3bFB}$<br><br>$k^{-}_{fC3bC3bFB}$	$21.3 \times 10^{4}M^{-1}s^{-1}$<br><br>$15.5 \times 10^{-2}s^{-1}$	Estimation functionally homologous protein fC3b and FB.
Binding rate for fC3b and C3b:FB<br><br>Disassociation of fC3b:C3b:FB	$k^{+}_{fC3bC4bFB}$<br><br>$k^{-}_{fC3bC4bFB}$	$21.3 \times 10^{4}M^{-1}s^{-1}$<br><br>$15.5 \times 10^{-2}s^{-1}$	Estimation functionally homologous protein fC3b and FB.
Decay of C3-convertase (fC3b:Bb) by down-regulator Factor H (FH)	$k^{-}_{dfC3bBbFH}$	$1.7 \times 10^{-2}\ s^{-1}$	[47]
Binding rate for fC3b and C4b	$k^{+}_{fC3bC4b}$	$4.2 \times 10^{8}M^{-1}s^{-1}$	[29]–[31].
Decay of initial C3-convertase (C3W:Bb) by inhibitor FH	$k^{-}_{dC3WBbFH}$	$1.7 \times 10^{-2}\ s^{-1}$	It is assumed based on the decay of functionally homologous protein fC3b:Bb down-regulated by FH.
Decay of C3-convertase (fC3b:Bb) by inhibitor DAF	$k^{-}_{dfC3bBbDAF}$	$1.7 \times 10^{-2}\ s^{-1}$	It is assumed based on the decay of functionally homologous protein fC3b:Bb down-regulated by FH.
Decay of C3-convertase (fC3b:Bb) by inhibitor CR1	$k^{-}_{dfC3bBbCR1}$	$1.7 \times 10^{-2}\ s^{-1}$	It is assumed based on the decay of functionally homologous protein fC3b:Bb down-regulated by FH.

Disassociation of C3-convertase (fC3b:Bb) and properdin (P) complex (fC3b:Bb:P)	$k^{-}_{fC3bBbP}$	$7.7 \times 10^{-4}\ s^{-1}$	[29]–[31], [48].
Disassociation of C5-convertase (C3b:Bb:C3b) and properdin (P) complex (C3b:Bb:C3b:P)	$k^{-}_{C3bBbC3bP}$	$5.7 \times 10^{-4}\ s^{-1}$	[30], [31], [48].
Decay of C5-convertase (C3b:Bb:C3b) by inhibitor FH	$k^{-}_{dfC3bBbC3bFH}$	$1.7 \times 10^{-2}\ s^{-1}$	It is assumed based on the decay of functionally homologous protein fC3b:Bb down-regulated by FH.
Decay of C5-convertase (fC3b:Bb:C3b) by inhibitor CR1	$k^{-}_{dfC3bBbC3bCR1}$	$1.7 \times 10^{-2}\ s^{-1}$	It is assumed based on the decay of functionally homologous protein fC3b:Bb down-regulated by FH.
Decay of C5-convertase (C3b:Bb:C3b) by inhibitor DAF on host cell	$k^{-}_{dC3bBbC3bDAF}$	$1.7 \times 10^{-2}\ s^{-1}$	It is assumed based on the decay of functionally homologous protein fC3b:Bb down-regulated by FH.
Decay of C5-convertase (C4b:C2a:C3b) by inhibitor CR1on host cell	$k^{-}_{dC4bC2aC3bCR1}$	$1.7 \times 10^{-2}\ s^{-1}$	It is assumed based on the decay of functionally homologous protein fC3b:Bb down-regulated by FH.
Decay of C5-convertase (C4b:C2a:C3b) by inhibitor DAF on host cell	$k^{-}_{dC4bC2aC3bDAF}$	$1.7 \times 10^{-2}\ s^{-1}$	It is assumed based on the decay of functionally homologous protein fC3b:Bb down-regulated by FH.
Decay of C3-convertase (C4b:C2a) by down-regulator C4BP on host cell	$k^{-}_{dC4bC2aC4BP}$	$1.7 \times 10^{-2}\ s^{-1}$	It is assumed based on the decay of functionally homologous protein fC3b:Bb down-regulated by FH.
Decay of C3-convertase (C4b:C2a) by down-regulator CR1 on host cell	$k^{-}_{dC4bC2aCR1}$	$1.7 \times 10^{-2}\ s^{-1}$	It is assumed based on the decay of functionally homologous protein fC3b:Bb down-regulated by FH.
Decay of C3-convertase (C4b:C2a) by down-regulator CR1 on host cell	$k^{-}_{dC4bC2aDAF}$	$1.7 \times 10^{-2}\ s^{-1}$	It is assumed based on the decay of functionally homologous protein fC3b:Bb down-regulated by FH.

Decay of C5-convertase (C4b:C2a:C3b) by down-regulator CR1 on host cell	$k^{-}_{dC4bC2aC3bCR1}$	$1.7 \times 10^{-2}\ s^{-1}$	It is assumed based on the decay of functionally homologous protein C4b:C2a down-regulated by CR1.
Disassociation of C5-convertase C4b:C2a:C3b	$k^{-}_{C4bC2aC3b}$	$5.0 \times 10^{-3}\ s^{-1}$	[29]–[31], [46].
Binding rate for CR1 and fC3b:C4b<br><br>Disassociation rate for fC3b:C4b:CR1	$k^{+}_{fC3bC4bCR1}$<br><br>$k^{-}_{fC3bC4bCR1}$	$9.8 \times 10^{4}\ M^{-1}s^{-1}$<br><br>$2.1 \times 10^{-3}\ s^{-1}$	[29]–[31], [49].
Binding rate for fC3b:C4b and FH<br><br>Disassociation rate for fC3b:C4b:FH	$k^{+}_{fC3bC4bFH}$<br><br>$k^{-}_{fC3bC4bFH}$	$1.1 \times 10^{6}\ M^{-1}s^{-1}$<br><br>$5.9 \times 10^{-2}\ s^{-1}$	Estimation functionally homologous protein fC3b and FH.
Binding rate for CR1 and fC3b:C4b:C2a<br><br>Disassociation rate for fC3b:C4b:C2a:CR1	$k^{+}_{fC3bC4bC2aCR1}$<br><br>$k^{-}_{fC3bC4bC2aCR1}$	$9.8 \times 10^{4}\ M^{-1}s^{-1}$<br><br>$2.1 \times 10^{-3}\ s^{-1}$	Estimation functionally homologous protein fC3b:C4b and CR1.
Binding rate for fC3b:Bb and P<br><br>Disassociation rate for fC3b:Bb:P	$k^{+}_{fC3bBbP}$<br><br>$k^{-}_{fC3bBbP}$	$1.5 \times 10^{5} M^{-1}s^{-1}$<br><br>$15.5 \times 10^{-5}\ s^{-1}$	Estimation functionally homologous protein fC3b and P.
Binding rate for fC3b:Bb:C3b and P<br><br>Disassociation rate for fC3b:Bb:C3b:P	$k^{+}_{fC3bBbC3bP}$<br><br>$k^{-}_{fC3bBbC3bP}$	$1.5 \times 10^{5} M^{-1}s^{-1}$<br><br>$15.5 \times 10^{-5}\ s^{-1}$	Estimation functionally homologous protein fC3b and P.
Binding rate for fC3b:FB and P<br><br>Disassociation rate for fC3b:FB:P	$k^{+}_{fC3bFBP}$<br><br>$k^{-}_{fC3bFBP}$	$1.5 \times 10^{5} M^{-1}s^{-1}$<br><br>$15.5 \times 10^{-5}\ s^{-1}$	Estimation functionally homologous protein fC3b and P.
Binding rate for lgG:fC3b:FB and P<br><br>Disassociation rate for lgG:fC3b:FB:P	$k^{+}_{fC3bFBP}$<br><br>$k^{-}_{fC3bFBP}$	$1.5 \times 10^{5} M^{-1}s^{-1}$<br><br>$15.5 \times 10^{-5}\ s^{-1}$	Estimation functionally homologous protein fC3b and P.
Synthesis rate of FB	k^{+}_{sFB}	$0.013 \times 10^{-9}\ Ms^{-1}$	[50] .
Degradation rate of FB	k^{-}_{dFB}	$5.5 \times 10^{-6}\ s^{-1}$	[50].

Synthesis rate of FH	k^{+}_{sFH}	$0.0117 \times 10^{-9}\ Ms^{-1}$	[50].
Degradation rate of FH	k^{-}_{dFH}	$3.7 \times 10^{-6}\ s^{-1}$	[50].
Synthesis rate of properdin (P)	k^{+}_{sP}	$1.17 \times 10^{-12}\ Ms^{-1}$	[50].
Degradation rate of properdin (P)	k^{-}_{dP}	$2.7 \times 10^{-7}\ s^{-1}$	[50].
Synthesis rate of C3	k^{+}_{sC3}	$0.053 \times 10^{-9}\ Ms^{-1}$	[50].
Degradation rate of C3	k^{-}_{dC3}	$6.5 \times 10^{-6}\ s^{-1}$	[50].
Hydrolysis of C3W	k^{+}_{C3W}	$4.5 \times 10^{-6}\ s^{-1}$	[29]–[31].
Binding rate for MBL and MASP1<br><br>Disassociation rate for MBL:MASP1	$k^{+}_{MBLMASP1}$<br><br>$k^{-}_{MBLMASP1}$	$2.1 \times 10^{5} M^{-1}s^{-1}$<br><br>$6.8 \times 10^{-4}\ s^{-1}$	[31], [51].
Binding rate for MBL and MASP2<br><br>Disassociation rate for MBL:MASP2	$k^{+}_{MBLMASP2}$<br><br>$k^{-}_{MBLMASP2}$	$2.3 \times 10^{5} M^{-1}s^{-1}$<br><br>$5.5 \times 10^{-4}\ s^{-1}$	[31], [51].
Cleavage of C3 by initial C3-convertase (C3W:Bb)	k^{C3WBb}_{catC3}<br><br>k^{C3WBb}_{mC3}	$0.78\ s^{-1}$<br><br>$11.6 \times 10^{-6}\ M$	The C3b:Bb have a half-life ($t_{\frac{1}{2}}$) of $90 \pm 2s$ and $\frac{k_{cat}}{k_m}$ of $31.1 \times 10^{4} \pm 0.8 \times 10^{4}$ $M^{-1}\ s^{-1}$. The C3b:Bb structurally/functionally homologous C3W:Bb [31] is found slightly less stable than C3b:Bb. It is known that C3W:Bb have $t_{\frac{1}{2}}$ of $77 \pm 3s$ and presented only half the activity of C3b:Bb ($\frac{k_{cat}}{k_m}$ is $16.3 \times 10^{4} \pm 16.3 \times 10^{4}$ $M^{-1}\ s^{-1}$) [43]. Assumption k_m of C3W:Bb found from increasing k_m of C3b:Bb [31].
Cleavage of C3 by C3-convertase (C3b:Bb)	k^{C3bBb}_{catC3}<br><br>k^{C3bBb}_{mC3}	$1.78\ s^{-1}$<br><br>$5.86 \times 10^{-6} M$	[29]–[31], [43].
Cleavage of C3 by C3-convertase (C4b:C2a)	k^{C4b2a}_{catC3}<br><br>k^{C4b2a}_{mC3}	$3.17\ s^{-1}$<br><br>$1.8 \times 10^{-6}\ M$	[29]–[31], [38].

Cleavage of C5 by C3-convertase (C3b:Bb)	k_{catC5}^{C3bBb}<br>k_{mC5}^{C3bBb}	$1.1 \times 10^{-2}\ s^{-1}$<br>$24.0 \times 10^{-6}$ M	[29]–[31], [38].
Cleavage of C5 by C3-convertase (C4b:C2a)	k_{catC5}^{C4b2a}<br>k_{mC5}^{C4b2a}	$2.2 \times 10^{-2}\ s^{-1}$<br>$8.9 \times 10^{-6}$ M	[29]–[31], [38].
Cleavage of C5 by C5-convertase (C4b:C2a:C3b)	$k_{catC5}^{C4b2a3b}$<br>$k_{mC5}^{C4b2a3b}$	$2.0 \times 10^{-2}\ s^{-1}$<br>$5.1 \times 10^{-9}$ M	[38].
Cleavage of C5 by C5-convertase (C3b:Bb:C3b)	$k_{catC5}^{C3bBbC3b}$<br>$k_{mC5}^{C3bBbC3b}$	$3.0 \times 10^{-3}\ s^{-1}$<br>$48.0 \times 10^{-9}$ M	[29]–[31], [38].
Activation of the product/complex C3W:FB by Factor D (FD)	$k_{catC3WFB}^{FD}$<br>k_{mC3WFB}^{FD}	$5.0\ s^{-1}$<br>2.5×10^{-6} M	[29]–[31].
Down-regulation/inhibition of the complex fC3b:FH by negative regulator Factor I (FI)	$k_{catfC3bFH}^{FI}$<br>$k_{mfC3bFH}^{FI}$	$1.32\ s^{-1}$<br>2.52×10^{-7} M	It is assumed based on the FI inhibition of functionally homologous protein C3b:FH.
Down-regulation of the complex C3b:FH by inhibitor FI	$k_{catC3bFH}^{FI}$<br>k_{mC3bFH}^{FI}	$1.32\ s^{-1}$<br>2.52×10^{-7} M	[29]–[31].
Down-regulation of the complex C3W:FH by FI	$k_{catC3WFH}^{FI}$<br>k_{mC3WFH}^{FI}	$1.32\ s^{-1}$<br>2.52×10^{-7} M	It is assumed based on the FI inhibition of functionally homologous protein C3b:FH.
Down-regulation of the complex fC3b:C4b:FH by FI	$k_{catfC3bC4bFH}^{FI}$<br>$k_{mfC3bC4bFH}^{FI}$	$1.32\ s^{-1}$<br>2.52×10^{-7} M	It is assumed based on the FI inhibition of functionally homologous protein C3b:FH.

Up-regulation of the complex fC3b:FB by FD	$k^{FD}_{catfC3bFB}$ $k^{FD}_{mfC3bFB}$	$1.32\ s^{-1}$ 2.52×10^{-7} M	Estimation structurally/functionally homologous protein C3W and Factor B [29]–[31], [34].
Inhibition of the complex fC3b:CR1 by FI	$k^{FI}_{catfC3bCR1}$ $k^{FI}_{mfC3bCR1}$	$1.32\ s^{-1}$ 2.52×10^{-7} M	It is assumed based on the FI inhibition of functionally homologous protein C3b:FH.
Inhibition of the complex C3b:CR1 by FI	$k^{FI}_{catC3bCR1}$ $k^{FI}_{mC3bCR1}$	$1.32\ s^{-1}$ 2.52×10^{-7} M	It is assumed based on the FI inhibition of functionally homologous protein C3b:FH.
Inhibition of the complex fC3b:C4b:CR1 by FI	$k^{FI}_{catfC3bC4bCR1}$ $k^{FI}_{mfC3bC4bCR1}$	$1.32\ s^{-1}$ 2.52×10^{-7} M	It is assumed based on the FI inhibition of functionally homologous protein C3b:FH.
Activation of the complex fC3b:FB:P by FD	$k^{FD}_{catfC3bFBP}$ $k^{FD}_{mfC3bFBP}$	$5.0\ s^{-1}$ 2.5×10^{-6} M	It is assumed based on the FD upregulation of functionally homologous protein C3W:FB.
Activation of the complex lgG:fC3b:FB by FD	$k^{FD}_{catlgGfC3bFB}$ $k^{FD}_{mlgGfC3bFB}$	$5.0\ s^{-1}$ 2.5×10^{-6} M	It is assumed based on the FD upregulation of functionally homologous protein C3W/FB.
Activation of the complex lgG:fC3b:FB:P by FD	$k^{FD}_{catlgGfC3bFBP}$ $k^{FD}_{mlgGfC3bFBP}$	$5.0\ s^{-1}$ 2.5×10^{-6} M	It is assumed based on the FD upregulation of functionally homologous protein C3W:FB.
Activation of the complex fC3b:C4b:FB by FD	$k^{FD}_{catfC3bC4bFB}$ $k^{FD}_{mfC3bC4bFB}$	$5.0\ s^{-1}$ 2.5×10^{-6} M	It is assumed based on the FD upregulation of functionally homologous protein C3W:FB .
Activation of the complex C3b:FB by FD	$k^{FD}_{catC3bFB}$ k^{FD}_{mC3bFB}	$5.0\ s^{-1}$ 2.5×10^{-6} M	It is assumed based on the FD upregulation of functionally homologous protein fC3b:FB [29]–[31].

Activation of the complex fC3b:C4b:P:FB by FD	$k^{FD}_{catfC3bC4bPFB}$<br><br>$k^{FD}_{mfC3bC4bPFB}$	$5.0\ s^{-1}$<br><br>2.5×10^{-6} M	It is assumed based on the FD upregulation of functionally homologous protein C3W:FB [29]–[31].
Cleavage rate of MASP fragment (MASP1) on C2 substrate	k^{MASP1}_{catC2}<br><br>k^{MASP1}_{mC2}	$0.1\ s^{-1}$<br><br>4.8×10^{-6} M	[31], [52].
Cleavage rate of MASP fragment (MASP2) on C2 substrate	k^{MASP2}_{catC2}<br><br>k^{MASP2}_{mC2}	$5.6\ s^{-1}$<br><br>5.2×10^{-6} M	[31], [53].
Cleavage rate of MASP fragment (MASP1) on C4 substrate	k^{MASP1}_{catC4}<br><br>k^{MASP1}_{mC4}	$2.0 \times 10^{-3}\ s^{-1}$<br><br>$5.2 \times 10^{-6}$ M	[31], [53].
Cleavage rate of MASP fragment (MASP2) on C4 substrate	k^{MASP2}_{catC4}<br><br>k^{MASP2}_{mC4}	$1.9\ s^{-1}$<br><br>8.5×10^{-8} M	[31], [53].
Cleavage rate of C2 by C1	k^{C1}_{catC2}<br><br>k^{C1}_{mC2}	$5.1\ s^{-1}$<br><br>6.1×10^{-6} M	[31], [53].
Cleavage rate of C4 by C1	k^{C1}_{catC4}<br><br>k^{C1}_{mC4}	$5.4\ s^{-1}$<br><br>6.10×10^{-6} M	[31], [53].
Cleavage rate of fC3b:C3b:FB by FD	$k^{FD}_{catfC3bC3bFB}$<br><br>$k^{FD}_{mfC3bC3bFB}$	$5.0\ s^{-1}$<br><br>2.5×10^{-6} M	[29]–[31].
Binding rate for C3a and C3aR1<br><br>Disassociation rate for C3a:C3aR1	$k^{+}_{C3aC3aR1}$<br><br>$k^{-}_{C3aC3aR1}$	$2.6 \times 10^{8}\ M^{-1}s^{-1}$<br><br>$0.1\ s^{-1}$	Disassociation constant (k_d) is 3.85×10^{-9}M [54]. Assume $k^{-} = 0.1\ s^{-1}$, Estimation for k^{+} by formula $k_d = \frac{k^{-}}{k^{+}}$

Binding rate for C5a and C5aR1<br><br>Disassociation rate for C5a:C5aR1	$k^{+}_{C5aC5aR1}$<br><br>$k^{-}_{C5aC5aR1}$	$2.2 \times 10^{8}\ M^{-1}s^{-1}$<br><br>$0.1\ s^{-1}$	Disassociation constant (k_d) for the product C5a:C5aR1 is $\approx 4.5 \times 10^{-9}$M and free energy of association (ΔG_{bind}) is -13.6 ± 4.1 kcalmol^{-1} [55]. Assumed $k^{-} =$ $0.1\ s^{-1}$, estimated for k^{+} by formula $k_d = \frac{k^{-}}{k^{+}} = e^{\frac{\Delta G_{bind}}{RT}}$.
Inhibition of C1q by C1INH	$k^{+}_{C1INHC1q}$	$9.5 \times 10^{4}\ M^{-1}s^{-1}$	C1 inhibitor binds to C1q [56].
Degradation rate of FD	k^{-}_{dFD}	$2.2 \times 10^{-4}\ s^{-1}$	[57], [58].
Synthesis rate of FD	k^{+}_{sFD}	$1.3 \times 10^{-11}\ Ms^{-1}$	[57].
Synthesis rate of C5	k^{+}_{sC5}	$2.2 \times 10^{-12}\ Ms^{-1}$	[57].
Degradation rate of C5	k^{-}_{dC5}	$5.6 \times 10^{-6}\ s^{-1}$	[57].
Synthesis rate of C6	k^{+}_{sC6}	$1.8 \times 10^{-10}\ Ms^{-1}$	[57].
Degradation rate of C6	k^{-}_{dC6}	$2.9 \times 10^{-4}\ s^{-1}$	[57].
Synthesis rate of C7	k^{+}_{sC7}	$1.9 \times 10^{-12}\ Ms^{-1}$	[57].
Degradation rate of C7	k^{-}_{dC7}	$3.2 \times 10^{-6}\ s^{-1}$	[57].
Synthesis rate of C8	k^{+}_{sC8}	$1.4 \times 10^{-12}\ Ms^{-1}$	[57].
Degradation rate of C8	k^{-}_{dC8}	$4.01 \times 10^{-6}\ s^{-1}$	[57].
Synthesis rate of C9	k^{+}_{sC9}	$3.4 \times 10^{-12}\ Ms^{-1}$	[57].
Degradation rate of C9	k^{-}_{dC9}	$4.01 \times 10^{-6}\ s^{-1}$	[57].
Degradation rate of Ba	k^{-}_{dBa}	$7.7 \times 10^{-5}\ s^{-1}$	[57].
Degradation rate of Bb	k^{-}_{dBb}	$7.7 \times 10^{-5}\ s^{-1}$	[57].
Degradation rate of C3W	k^{-}_{dC3W}	$4.01 \times 10^{-6}\ s^{-1}$	[57].
Degradation rate of C3WFB	k^{-}_{dC3WFB}	$4.01 \times 10^{-6}\ s^{-1}$	[57].

Degradation rate of C3WBb	k^{-}_{dC3WBb}	$4.01 \times 10^{-6}\ s^{-1}$	[57].
Degradation rate of fC3b	k^{-}_{dfC3b}	$4.01 \times 10^{-6}\ s^{-1}$	[57].
Degradation rate of fC3bFB	$k^{-}_{dfC3bFB}$	$4.01 \times 10^{-6}\ s^{-1}$	[57].
Degradation rate of fC3bBb	$k^{-}_{dfC3bBb}$	$4.01 \times 10^{-6}\ s^{-1}$	[57].
Degradation rate of C5b	k^{-}_{dC5b}	$5.0 \times 10^{-3}\ s^{-1}$	[57], [59].
Degradation rate of C5a	k^{-}_{dC5a}	$1.15 \times 10^{-2}\ s^{-1}$	[57], [60].
Degradation rate of C3b	k^{-}_{dC3b}	$4.01 \times 10^{-6}\ s^{-1}$	[57].
Degradation rate of C3a	k^{-}_{dC3a}	$1.15 \times 10^{-2}\ s^{-1}$	[57].
Binding rate for C1q and gC1qR<br><br>Disassociation rate for C1q:gC1qR	$k^{+}_{C1qgC1qR}$<br><br>$k^{-}_{C1qC1qR}$	$4.0 \times 10^{2}\ M^{-1}s^{-1}$<br><br>$0.1\ s^{-1}$	C1q binds to gC1q-receptor (gC1qR) with an affinity ($k_d = 250 \times 10^{-6}$) [61][50]. The disassociation rate (k^{-1}) is unknown, assumed a small value for $k^{-1} = 0.1\ s^{-1}$. Using the formula $k_d = \frac{k^-}{k^+}$, determined the association rate (k^+).
Inhibition of MAC by CD59	$k^{+}_{MACCD59}$	$1.0 \times 10^{6}\ M^{-1}s^{-1}$	The terminal complement complex (TCC),<br>C5b-9 (MAC) bind to CD59 glycoprotein, also known as MAC-inhibitory protein (MAC-IP) [29].

Table S1c. The hemostatic and complement systems cross-talk entities interaction kinetic parameters.

Meaning	Rate constant	Value adopted	Evidences and References
Inhibition of F12a by C1INH	$k^{+}_{C1INHF12a}$	$3.6 \times 10^{3}\ M^{-1}s^{-1}$	C1INH suppresses F12a [62], [63].
Inhibition of KAL by C1INH	$k^{+}_{C1INHKAL}$	$1.7 \times 10^{4}\ M^{-1}s^{-1}$	C1INH suppresses KAL [62], [63].<br>Association rate of C1INH and KLK [64].
Inhibation of F2a by FH	k^{+}_{FHF2a}	$3.367 \times 10^{7}\ M^{-1}s^{-1}$	For FH and F2a the disassociation constant (k_d) is measured 29.9nM. Binding affinity of FH and F2a range (3.1 - 200 nM) [65]. Estimation for association rate (k^{+}_{FHF2a}) based on k_d.
Inhibation of F1a by FH	k^{+}_{FHF1a}	$2.62 \times 10^{7}\ M^{-1}s^{-1}$	FH and F1a disassociation constant (k_d) is measured 38.2nM. Binding affinity of FH and F2a range (3.1 - 200 nM) [65]. Estimation for association rate (k^{+}_{FHF1a}) based on k_d.
Cleavage of C5 by Pn	k^{Pn}_{catC5}<br>k^{Pn}_{mC5}	$0.056\ s^{-1}$<br>2.4×10^{-6}	Pn can cleaves C5 generating C5a with a catalytic efficiency ($\frac{k_{cat}}{k_m}$) of 2.3×10^{4} $M^{-1}s^{-1}$ [66], [67].
Cleavage of C5 by F2a	k^{F2a}_{catC5}<br>k^{F2a}_{mC5}	$0.56\ s^{-1}$<br>2.4×10^{-6}	F2a is less effective than Pn in cleaving C5 to generates C5a [66]. We could estimate catalytic efficiency ($\frac{k_{cat}}{k_m}$) of $2.3 \pm 0.6 \times 10^{3}\ M^{-1}s^{-1}$ based on Pn catalytic efficiency.
Cleavage of C3 by Pn	k^{Pn}_{catC3}<br>k^{Pn}_{mC3}	$0.056\ s^{-1}$<br>2.4×10^{-6}	Pn can cleaves C3. The cleavage of C3 by Pn is assumed based on functionally homologous C5.
Cleavage of C3 by F2a	k^{F2a}_{catC3}<br>k^{F2a}_{mC3}	$0.56\ s^{-1}$<br>2.4×10^{-6}	The cleavage of C3 by F2a is assumed based on functionally homologous C5.
Cleavage of C3 by F10a	k^{F10a}_{catC3}<br>k^{F10a}_{mC3}	$0.056\ s^{-1}$<br>2.4×10^{-5}	F10a is less effective than Pn in cleaving C3 to generates C3a [66]. We could estimate catalytic efficiency ($\frac{k_{cat}}{k_m}$) of $2.3 \times 10^{3}\ M^{-1}s^{-1}$ based on Pn catalytic efficiency.

Cleavage of C5 by F10a	k_{catC5}^{F10a}<br>k_{mC5}^{F10a}	$0.056\ s^{-1}$<br>2.4×10^{-5}	F10a is less effective than Pn in cleaving C5 to generates C5a, respectively [66]. We could estimate catalytic efficiency ($\frac{k_{cat}}{k_m}$) of $2.3 \pm 0.6 \times 10^3\ M^{-1}s^{-1}$ based on Pn catalytic efficiency.
Production of TF by C5a	k_{catTF}^{C5a}	$0.01 \times 10^{-3}\ s^{-1}$	C5a can increase the activity and expression of TF [68]. C5a stimulate the expression of TF on neutrophils via C5aR [62], [69]. Estimated a small value.
Production of TF by C3a	k_{catTF}^{C3a}	$0.01 \times 10^{-3}\ s^{-1}$	C3a increases the expression of TF [68]. Assumption based on C3a functional homologous C5a.
Inhibition of F11a by C1INH	$k_{C1INHF11a}^{+}$	$1.8 \times 10^3\ M^{-1}s^{-1}$	CIINH binds with F11a and inhibit it [2], [3]
Inhibition of F2a by C1INH	$k_{C1INHF2a}^{+}$	$1.0 \times 10^2\ M^{-1}s^{-1}$	C1-INH can inhibit F2a. Assumption.
Inhibition of Plasmin by C1INH	$k_{C1INHPlasmin}^{+}$	$5.5 \times 10^3\ M^{-1}s^{-1}$	Kinetic analysis for inhibition experiment with Pn, generated an association rate for plasma C1INH [70].
Generation of F1 by IL6	k_{catF1}^{IL6}	$0.01 \times 10^{-6}\ s^{-1}$	IL6 induces the expression of F1. IL-6 correlated positively with F1 concentration [71]. Assume a small random value for generation of F1 by IL6.
Activation rate of F2 by MASP1	k_{catF2}^{MASP1}	$141.0 \times 10^3\ M^{-1}s^{-1}$	MASP1 activates and cleaves F2 to F2a [63], [72], [73]. Activation of F2 wildtype by MASP1 computed via kinetic analysis [74].
Activation rate of F2 by MASP2	k_{catF2}^{MASP2}	$141.0 \times 10^3\ M^{-1}s^{-1}$	MASP2 activates and cleaves F2 into F2a [63], [73]. Activation rate of F2 by MASP2 estimated based on functionally homologous MASP1.
Activation rate of F1 by MASP1	k_{catF1}^{MASP1}	$141.0 \times 10^3\ M^{-1}s^{-1}$	MASP1 activate F1 to F1a [63], [72], [73]. Assumed based on the activation rate of F2 by MASP1.
Activation rate of F1 by MASP2	k_{catF1}^{MASP2}	$141.0 \times 10^3\ M^{-1}s^{-1}$	MASP1 activate F1 to F1a [63], [72], [73]. Activation rate of F1 by MASP2 estimated based on functionally homologous MASP1.
Generation of F2 by MAC	k_{catF2}^{MAC}	$0.1\ s^{-1}$	Estimation for MAC (C5b-C9) can generate F2 [62].
Activation of C1r by F12a	k_{catC1r}^{F12a}	$1.0 \times 10^2\ M^{-1}s^{-1}$	F12a can activate the C1r [62], [68], [75]. Assumed a small value for the activation of C1r by F12a.
Association rate for gC1qR and F12	$k_{gC1qRF12}^{+}$	$8.3 \times 10^{13} M^{-1}s^{-1}$	gC1qR binds to F12 with disassociation constant $k_d = 120 \times 10^{-9}$M [76]. Assumed a small value for $k^{-1} = 0.1\ s^{-1}$.

Disassociation rate for gC1qR:F12	$k^{-}_{gC1qRF12}$	0.1 s^{-1}	Using formula $k_d = \frac{k^-}{k^+}$, estimated for association rate (k^+).
Production of F12a by gC1qR:F12	$k^{gC1qRF12}_{catF12a}$	0.1 s^{-1}	The complex of gC1qR and F12 can generates F12a [77].
Association rate for gC1qR and HK<br><br>Disassociation rate for gC1qR:HK	$k^{+}_{gC1qRHK}$<br><br>$k^{-}_{gC1qRHK}$	$5.3 \times 10^{11} M^{-1}s^{-1}$<br><br>0.1 s^{-1}	gC1q-receptor (gC1qR) binds to HK with disassociation constant $k_d = 1.9 \times 10^{-9}$M [76]. Assumed a small value for $k^{-1} = 0.1\ s^{-1}$.<br>Using formula $k_d = \frac{k^-}{k^+}$, estimated the association rate (k^+).
Production of BK by gC1qR:HK	$k^{gC1qRHK}_{catBK}$	0.1 s^{-1}	The product of gC1qR and HK can release BK [77]. The generation rate is unknown, estimated a small value for it.
Cleavage of FB by KAL	k^{KAL}_{catFB}<br><br>k^{KAL}_{mFB}	0.01 s^{-1}<br><br>$1.0 \times 10^{-12}\ M$	KAL can cleaves FB [68], [77]. Assumed small values for the catalytic rate constant and Michaelis constant. FB co-exist with hydrolyzed C3 (C3W) and fC3b
Cleavage of C3 by KAL	k^{KAL}_{catC3}<br><br>k^{KAL}_{mC3}	0.01 s^{-1}<br><br>$1.0 \times 10^{-12}\ M$	KAL can cleaves C3 [68], [77]. Assumed small values for the catalytic rate constant and Michaelis constant.
Cleavage of C5 by KAL	k^{KAL}_{catC5}<br><br>k^{KAL}_{mC5}	0.01 s^{-1}<br><br>$1.0 \times 10^{-12}\ M$	Kallikrein can cleaves C5 [68]. Assumed small values for the catalytic rate constant and Michaelis constant.
Degradation rate of IL-6	k^{-}_{dIL6}	0.01s^{-1}	Assumption
Production of IL6 by BK:B2R	k^{BKB2R}_{IL6}	0.1 s^{-1}	BK via B2R can stimulate the production of IL6 [78]<br>Estimation for the production rate of IL6 by BK and B2R complex.
Production of IL6 by C3a:C3aR	$k^{C3aC3aR1}_{IL6}$	0.1 s^{-1}	C3a via C3aR1 can induce IL6 [79]. Estimation for the production rate of IL6 by C3a and C3aR complex.
Production of IL6 by C5a:C5aR	$k^{C5aC5aR1}_{IL6}$	0.1 s^{-1}	C5a via C5aR1 can induce interleukin 6 (IL6) [79]. Estimation for production rate of IL6 by C5a and C5aR complex.
Activation of Pro-FD by MASP1	$k^{MASP1}_{catPro-DF}$	$3.9 \times 10^{3}\ M^{-1}s^{-1}$	Activation and cleavage of Pro-FD by MASP1 that can be converted into active FD [80].

Activation of Pro-FD by MASP2	$k_{catPro-FD}^{MASP2}$	$7.2 \times 10^{3}\ M^{-1}s^{-1}$	Activation and cleavage of Pro-FD by MASP2 that can be converted into active FD [80].
Activation of Pro-FD by F2a	$k_{catPro-FD}^{F2a}$	$4.6 \times 10^{2}\ M^{-1}s^{-1}$	The activation rates of pro-FD by thrombin (F2a) [80].
Cleavage of TAFI by MASP1	$k_{catTAFI}^{MASP1}$<br><br>k_{mTAFI}^{MASP1}	$0.1\ s^{-1}$<br><br>$3.9 \times 10^{-8}\ M$	MASP-1 directly activates/ cleaves TAFI [72], [77].
Association rate for IL-6 and IL-6R<br><br>Disassociation rate for IL-6:IL-6R	$k_{IL-6IL-6R}^{+}$<br><br>$k_{IL-6IL-6R}^{-}$	$1.0 \times 10^{5} M^{-1}s^{-1}$<br><br>$1.0 \times 10^{-3}\ s^{-1}$	Assumption

Table S1d. The hemostatic and complement systems entities and SARS-CoV-2 structure proteins interaction kinetic parameters.

Meaning	Rate constant	Value adopted	Evidences and References
Association rate for gC1qR and CoV2S	$k_{gC1qRCoV2S}^{+}$	$1.1 \times 10^{10} M^{-1}s^{-1}$	Binding of S protein of SARS-CoV-2 (termed as CoV2S) on the surface of wild-type gC1qR of classical pathway of the complement system. The binding affinity is $k_d = 91 \times 10^{-12}$M [81]. The disassociation rate (k^-) is unknown, assume $k^- = 1s^{-1}$. Using $k_d = \frac{k^-}{k^+}$ determined the association rate (k^+).
Association rate for gC1qR and CoV2N	$k_{C1qRCoV2N}^{+}$	$1.7 \times 10^{5} M^{-1}s^{-1}$	Binding of N protein of SARS-CoV-2 (termed as CoV2N) on the surface of wild-type gC1qR of classical pathway of the complement system. The binding affinity is $k_d = 6 \times 10^{-6}$M [81].<br>Assume $k^- = 1s^{-1}$. Using $k_d = \frac{k^-}{k^+}$ determined the association rate (k^+).
Association rate for gC1qR and CoV2M	$k_{C1qRCoV2M}^{+}$	$2.4 \times 10^{9}\ M^{-1}s^{-1}$	Binding of the M protein of SARS-CoV-2 (termed as CoV2M) on the surface of wild-type C1qR of classical pathway of the complement system. The binding affinity is $k_d = 410 \times 10^{-12}$ M [81].<br>Assume $k^- = 1s^{-1}$. Using $k_d = \frac{k^-}{k^+}$ determined the association rate (k^+).
Association rate for gC1qR and CoV2E	$k_{C1qRCoV2E}^{+}$	$2.4 \times 10^{9}\ M^{-1}s^{-1}$	Binding of the E protein of SARS-CoV-2 (termed as CoV2E) on the surface of wild-type C1qR classical pathway of the complement system. The binding affinity $k_d = 410 \times 10^{-12}$ M [81].

			Assume $k^{-} = 1s^{-1}$. Using $\mathrm{k_d} = \frac{\mathrm{k}^{-}}{\mathrm{k}^{+}}$ determined the association rate (k^{+}).
Association rate for F12 and CoV2S	$\mathrm{k}^{+}_{\mathrm{CoV2SF12}}$	$1.0 \times 10^{6} M^{-1}s^{-1}$	S protein of SARS-CoV-2 (termed as CoV2S) binds to inactive coagulation factor F12 of contact pathway that is intrinsic pathway of the coagulation cascade [81]. Assumed for binding rate of F12 and CoV2S.
Association rate for F12 and CoV2N	$\mathrm{k}^{+}_{\mathrm{CoV2NF12}}$	$1.0 \times 10^{4} M^{-1}s^{-1}$	N protein of SARS-CoV-2 (termed as CoV2N) binds to coagulation factor F12 [81]. Assumption for binding rate of F12 and CoV2N.
Association rate for F12 and CoV2M	$\mathrm{k}^{+}_{\mathrm{CoV2MF12}}$	$1.0 \times 10^{4} M^{-1}s^{-1}$	M protein of SARS-CoV-2 (termed as CoV2M) binds to coagulation factor F12 [81]. Assumption for binding rate of F12 and CoV2M.
Association rate for F12 and CoV2E	$\mathrm{k}^{+}_{\mathrm{CoV2EF12}}$	$1.0 \times 10^{4} M^{-1}s^{-1}$	E protein of SARS-CoV-2 (termed as CoV2E) binds to coagulation factor F12 [81]. Assumption for binding rate of F12 and CoV2S.
Association rate for HK and CoV2S	$\mathrm{k}^{+}_{\mathrm{HKCoV2S}}$	$1.0 \times 10^{3} M^{-1}s^{-1}$	S protein of SARS-CoV-2 (termed as CoV2S) binds to HK of the system KKS [81]. Assumption for binding rate.
Association rate for HK and CoV2N	$\mathrm{k}^{+}_{\mathrm{HKCoV2N}}$	$1.0 \times 10^{3} M^{-1}s^{-1}$	N protein of SARS-CoV-2 (termed as CoV2N) binds to HK of the system KKS [81]. Assumption for binding rate.
Association rate for HK and CoV2M	$\mathrm{k}^{+}_{\mathrm{HKCoV2M}}$	$1.0 \times 10^{3} M^{-1}s^{-1}$	M protein of SARS-CoV-2 (termed as CoV2M) binds to HK of the system KKS [81]. Assumption for binding rate.
Association rate for HK and CoV2E	$\mathrm{k}^{+}_{\mathrm{HKCoV2E}}$	$1.0 \times 10^{3} M^{-1}s^{-1}$	E protein of SARS-CoV-2 (termed as CoV2E) binds to HK of the system KKS [81]. Assumption for binding rate.
Association rate for MASP2 by CoV2N protein	$\mathrm{k}^{+}_{\mathrm{MASP2CoV2N}}$	$6.9 \times 10^{4} M^{-1}s^{-1}$	The N protein of SARS-CoV-2 (termed as CoV2N) can cleaved/ activate MASP2 [82]. $\mathrm{k_{cat}} = 3.64\ s^{-1}$ and $k_m = 5.437 \times 10^{-5}$ M. Assume a small value for disassociation rate $k^{-} = 0.1\mathrm{s}^{-1}$. Using the formula $k^{+} = \frac{k^{-} + \mathrm{k_{cat}}}{k_m}$, determined the association rate (k^{+}).
Binding rate for lgG and CoV2S<br>Disassociation rate for the complex lgG/CoV2S	$\mathrm{k}^{+}_{\mathrm{CoV2SlgG}}$<br>$\mathrm{k}^{-}_{\mathrm{CoV2SlgG}}$	$2.14 \times 10^{5} M^{-1}s^{-1}$<br>$1.70 \times 10^{-2}\ s^{-1}$	Association rate for lgG via Fab (ID-1F4) bind with spike (S) protein of SARS-CoV2 (termed as CoV2N) [83].
Association rate of CoV2S/lgG and C1q	$\mathrm{k}^{+}_{\mathrm{CoV2SlgGC1q}}$	$2.3 \times 10^{5} M^{-1}s^{-1}$	S protein of SARS-CoV2 binding by antibody lgG mediated C1q activate complement system through classical pathway [44]. An antibody IgG1 subclass antibodies Rituximab

			(RTX) and Ofatumumab (OFA) bind with C1q. Association rates of C1q and RTX, C1q and OFA are $5.2 \times 10^4 M^{-1}s^{-1}$ and $2.3 \times 10^5 M^{-1}s^{-1}$ [84].<br>Estimation for lgG based on structurally/ functionally homologous lgG1 subclass.
Cleavage rate of CoV2S by F10a	$k_{catCoV2S}^{F10a}$<br><br>k_{mCoV2S}^{F10a}	$1.79 \times 10^{-1}\ s^{-1}$<br><br>$4.035 \times 10^{-5}\ M$	Cleavage rate of S protein by active coagulation factor F10a [85].
Cleavage rate of CoV2S by F2a	$k_{catCoV2S}^{F2a}$<br><br>k_{mCoV2S}^{F2a}	$5.20 \times 10^{-2}\ s^{-1}$<br><br>$16.34 \times 10^{-6}\ M$	Cleavage rate of S protein by activated coagulation factor F2a [85].

Table S1e. The Drug-Target interaction kinetic parameters.

Meaning	Rate constant	Value adopted	Evidences and References
Association of Heparin to C2<br><br>Disassociation of Heparin:C2	k_{HepC2}^{+}<br><br>k_{HepC2}^{-}	$4.13 \times 10^3 M^{-1}s^{-1}$<br><br>$1.32 \times 10^{-3}\ s^{-1}$	[86]
Association of Heparin to C3<br><br>Disassociation of Heparin:C3	k_{HepC3}^{+}<br><br>k_{HepC3}^{-}	$1.83 \times 10^4 M^{-1}s^{-1}$<br><br>$5.73 \times 10^{-4}\ s^{-1}$	[86]
Association of Heparin to C4<br><br>Disassociation of Heparin:C4	k_{HepC4}^{+}<br><br>k_{HepC4}^{-}	$9.64 \times 10^4 M^{-1}s^{-1}$<br><br>$3.46 \times 10^{-3}\ s^{-1}$	[86]
Association of Heparin to C5<br><br>Disassociation of Heparin:C5	k_{HepC5}^{+}<br><br>k_{HepC5}^{-}	$2.43 \times 10^5 M^{-1}s^{-1}$<br><br>$1.35 \times 10^{-3}\ s^{-1}$	[86]
Association of Heparin to C6	k_{HepC6}^{+}	$1.72 \times 10^4 M^{-1}s^{-1}$	[86]

Disassociation of Heparin:C6	k^{-}_{HepC6}	$5.58 \times 10^{-4}\ s^{-1}$	
Association of Heparin to C7<br><br>Disassociation of Heparin:C7	k^{+}_{HepC7}<br><br>k^{-}_{HepC7}	$6.25 \times 10^{4} M^{-1} s^{-1}$<br><br>$9.60 \times 10^{-4}\ s^{-1}$	[86]
Association of Heparin to C8<br><br>Disassociation of Heparin:C8	k^{+}_{HepC8}<br><br>k^{-}_{HepC8}	$4.17 \times 10^{4} M^{-1} s^{-1}$<br><br>$5.74 \times 10^{-3}\ s^{-1}$	[86]
Association of Heparin to C9<br><br>Disassociation of Heparin:C9	k^{+}_{HepC9}<br><br>k^{-}_{HepC9}	$3.43 \times 10^{4} M^{-1} s^{-1}$<br><br>$4.51 \times 10^{-3}\ s^{-1}$	[86]
Activation of AT3 by Heparin	k^{+}_{HepAT3}	$1 \times 10^{2} M^{-1} s^{-1}$	Heparin as potentiator of AT3 [87]. Assumption
Inhibition of C5aR1 by Avdoralimab	$k^{+}_{AvdC5aR1}$	$1.0 \times 10^{3} M^{-1} s^{-1}$	Avdoralimab (IPH5401) is a therapeutic antibody that specifically binds and blocks C5a receptors (C5aR1). It is safely used in COVID-19 patients [88]. Assumption.
Inhibition of tPA by Tranexamic acid (TXA)	k^{+}_{TXAtPA}	$1.0 \times 10^{2} M^{-1} s^{-1}$	Assumption.
Inhibition of IL-6R by Tocilizumab (TCZ)	$k^{+}_{TCZIL-6R}$	$1.0 \times 10^{3} M^{-1} s^{-1}$	

Abbreviations

MBL, Mannose binding lectin; MASP1 and MASP2 stand for MBL associated Mannan-binding lectin serine protease MASP-1 and MASP-2;

C2, Complement component 2; C2a, Complement component 2a; C2b, Complement component 2a; C3aR1, C3a anaphylatoxin Receptor 1;

C4BP, Complement component 4 binding protein; CR1, Complement receptor 1; DAF, Decay Accelerating Factor;

FB, FD, FH and FI stand for Complement factor B, D, H and I, respectively;

P, Properdin; IgG, Immunoglobulin G; C1q, C1r, C1s Complement components C1q, C1r, C1s; C1, Activated Complement component 1;

Ba, cleavage fragment of FB; Bb, cleavage fragment of FB; MAC, Membrane attack complex (MAC); IL6, Interleukin 6;

F1 stand for Coagulation factor 1 (Fibrinogen); F1a, Coagulation factor 1a (Fibrin); F2, Coagulation factor 2 (Pro-Thrombin);

F2a, Coagulation factor 2a (Thrombin); F5, Coagulation factor 5 (Labile factor, Proaccelerin);

F5a, Activated coagulation factor 5; F7, Coagulation Factor 7 (Proconvertin); F7a, Activated coagulation factor 7;

F8, Coagulation factor 8 (Antihemophilic factor A); F8a, Activated coagulation factor 8; F9, Coagulation factor 9 (Antihemophilic factor B);

F9a, Activated coagulation factor 9; F10, Coagulation factor 10 (Thrombokinase);   F10, Activated coagulation factor 10;

F11, Coagulation factor 11 (Plasma thromboplastin antecedent); F11, Activated coagulation factor 11; F12, Coagulation factor 12 (Hageman factor);

F12a, Activated coagulation factor 12; KAL, Kallikrein; P-KAL, Pre-Kallikrein;

Pn, Plasmin; Pg, Plasminogen; TF, Tissue factor (Tissue thromboplastin); TFPI, Tissue factor pathway inhibitor;

TAFI, Thrombin-activatable fibrinolysis inhibitor; TAFIa, Activated Thrombin-activatable fibrinolysis inhibitor; BK, Bradykinin;

C3a and C5a stand for complement anaphylatoxins;

HK, High-molecular-weight kininogen; tPA, Tissue-type plasminogen activator;   A2M, Association rate of Alpha-2 macroglobulin;
AT3, Anti-Thrombin 3 (Serpin); PAI-1, Plasminogen activator inhibitor-1; B2R, B2-receptor.

S stand for Spike protein; N, Nucleocapsid; M, Membrane; E, Envelope;

KKS, Kallikrein-kinin system.

Reference

[1] A. M. Susree and B. M. Anand, "Reaction Mechanisms and Kinetic Constants used in Mechanistic Models of Coagulation and Fibrinolysis," *Math. Model. Nat. Phenom.*, vol. 11, no. 6, pp. 71–90, 2016, doi: 10.1051/mmnp/201611605.

[2] M. S. Chatterjee, W. S. Denney, H. Jing, and S. L. Diamond, "Systems biology of coagulation initiation: Kinetics of thrombin generation in resting and activated human blood," *PLoS Comput. Biol.*, vol. 6, no. 9, 2010, doi: 10.1371/journal.pcbi.1000950.

[3] R. Méndez Rojano *et al.*, "Kinetics of the coagulation cascade including the contact activation system: sensitivity analysis and model reduction," *Biomech. Model. Mechanobiol.*, vol. 18, no. 4, pp. 1139–1153, 2019, doi: 10.1007/s10237-019-01134-4.

[4] R. D. Feinman, A. I. Yuan, S. R. Windwer, and D. Wang, “Kinetics of the reaction of thrombin and α2-macroglobulin,” *Biochem. J.*, vol. 231, no. 2, pp. 417–423, 1985, doi: 10.1042/bj2310417.

[5] M. A. Kelley and K. Leiderman, *Mathematical modeling to understand the role of bivalent thrombin-fibrin binding during polymerization*, vol. 18, no. 9. 2022. doi: 10.1371/journal.pcbi.1010414.

[6] M. C. Naski and J. A. Shafer, “A kinetic model for the α-thrombin-catalyzed conversion of plasma levels of fibrinogen to fibrin in the presence of antithrombin III,” *J. Biol. Chem.*, vol. 266, no. 20, pp. 13003–13010, 1991, doi: 10.1016/s0021-9258(18)98795-8.

[7] M. Nesheim, J. C. Fredenburgh, and G. R. Larsen, “The dissociation constants and stoichiometries of the interactions of Lys-plasminogen and chloromethyl ketone derivatives of tissue plasminogen activator and the variant ΔFEIX with intact fibrin,” *J. Biol. Chem.*, vol. 265, no. 35, pp. 21541–21548, 1990, doi: 10.1016/s0021-9258(18)45774-2.

[8] P. Y. Kim, L. D. Tieu, A. R. Stafford, J. C. Fredenburgh, and J. I. Weitz, “A high affinity interaction of plasminogen with fibrin is not essential for efficient activation by tissue-type plasminogen activator,” *J. Biol. Chem.*, vol. 287, no. 7, pp. 4652–4661, 2012, doi: 10.1074/jbc.M111.317719.

[9] B. N. Bouma and J. C. M. Meijers, “Thrombin-activatable fibrinolysis inhibitor (TAFI, plasma procarboxypeptidase B, procarboxypeptidase R, procarboxypeptidase U),” *J. Thromb. Haemost.*, vol. 1, no. 7, pp. 1566–1574, 2003, doi: 10.1046/j.1538-7836.2003.00329.x.

[10] W. Wang, M. B. Boffa, L. Bajzar, J. B. Walker, and M. E. Nesheim, “A study of the mechanism of inhibition of fibrinolysis by activated thrombin-activable fibrinolysis inhibitor,” *J. Biol. Chem.*, vol. 273, no. 42, pp. 27176–27181, 1998, doi: 10.1074/jbc.273.42.27176.

[11] L. Bajzar, R. Manuel, and M. E. Nesheim, “Purification and characterization of TAFI, a thrombin-activable fibrinolysis inhibitor,” *J. Biol. Chem.*, vol. 270, no. 24, pp. 14477–14484, 1995, doi: 10.1074/jbc.270.24.14477.

[12] J. Foley, “A Quantitative and Mechanistic Assessment of Activated Thrombin-Activatable Fibrinolysis Inhibitor and its Role in Pathological Bleeding and Thrombosis,” 2010.

[13] B. E. Bannish, I. N. Chernysh, J. P. Keener, A. L. Fogelson, and J. W. Weisel, “Molecular and Physical Mechanisms of Fibrinolysis and Thrombolysis from Mathematical Modeling and Experiments,” *Sci. Rep.*, vol. 7, no. 1, pp. 1–11, 2017, doi: 10.1038/s41598-017-06383-w.

[14] J. R. Yaron, L. Zhang, Q. Guo, S. E. Haydel, and A. R. Lucas, “Fibrinolytic Serine Proteases, Therapeutic Serpins and Inflammation: Fire Dancers and Firestorms,” *Front. Cardiovasc. Med.*, vol. 8, no. March, pp. 1–21, 2021, doi: 10.3389/fcvm.2021.648947.

[15] L. O. Mosnier and B. N. Bouma, “Regulation of fibrinolysis by Thrombin Activatable Fibrinolysis Inhibitor, an unstable carboxypeptidase B that unites the pathways of coagulation and fibrinolysis,” *Arterioscler. Thromb. Vasc. Biol.*, vol. 26, no. 11, pp. 2445–2453, 2006, doi: 10.1161/01.ATV.0000244680.14653.9a.

[16] T. Renné, J. Dedio, J. C. M. Meijers, D. Chung, and W. Müller-Esterl, “Mapping of the discontinuous H-kininogen binding site of plasma

prekallikrein. Evidence for a critical role of apple domain-2," *J. Biol. Chem.*, vol. 274, no. 36, pp. 25777–25784, 1999, doi: 10.1074/jbc.274.36.25777.

[17] M. L. Merchant *et al.*, "Plasma kininogen and kininogen fragments are biomarkers of progressive renal decline in type 1 diabetes," *Kidney Int.*, vol. 83, no. 6, pp. 1177–1184, Jun. 2013, doi: 10.1038/ki.2013.8.

[18] J. Feierler, M. Wirth, B. Welte, S. Schüssler, M. Jochum, and A. Faussner, "Helix 8 plays a crucial role in bradykinin B 2 receptor trafficking and signaling," *J. Biol. Chem.*, vol. 286, no. 50, pp. 43282–43293, 2011, doi: 10.1074/jbc.M111.256909.

[19] F. S. Seaman, F. A. Baglia, J. A. Gurr, B. A. Jameson, and P. N. Walsh, "Binding of high-molecular-mass kininogen to the Apple 1 domain of factor XI is mediated in part by Val64 and Ile77," *Biochem. J.*, vol. 304, no. 3, pp. 715–721, 1994, doi: 10.1042/bj3040715.

[20] S. Meini *et al.*, "Understanding the Pathophysiology of COVID-19: Could the Contact System Be the Key?," *Front. Immunol.*, vol. 11, no. August, pp. 1–9, 2020, doi: 10.3389/fimmu.2020.02014.

[21] L. Bajzar, J. Morser, and M. Nesheim, "TAFI, or plasma procarboxypeptidase B, couples the coagulation and fibrinolytic cascades through the thrombin-thrombomodulin complex," *J. Biol. Chem.*, vol. 271, no. 28, pp. 16603–16608, 1996, doi: 10.1074/jbc.271.28.16603.

[22] J. H. Foley, P. F. Cook, and M. E. Nesheim, "Kinetics of activated thrombin-activatable fibrinolysis inhibitor (TAFIa)-catalyzed cleavage of C-terminal lysine residues of fibrin degradation products and removal of plasminogen-binding sites," *J. Biol. Chem.*, vol. 286, no. 22, pp. 19280–19286, 2011, doi: 10.1074/jbc.M110.215061.

[23] T. Myles *et al.*, "Thrombin Activatable Fibrinolysis Inhibitor, a Potential Regulator of Vascular Inflammation," *J. Biol. Chem.*, vol. 278, no. 51, pp. 51059–51067, 2003, doi: 10.1074/jbc.M306977200.

[24] F. Napolitano and N. Montuori, "The role of the plasminogen activation system in angioedema: Novel insights on the pathogenesis," *J. Clin. Med.*, vol. 10, no. 3, pp. 1–14, 2021, doi: 10.3390/jcm10030518.

[25] J. Vakili, L. Ständker, M. Detheux, G. Vassart, W.-G. Forssmann, and M. Parmentier, "Urokinase Plasminogen Activator and Plasmin Efficiently Convert Hemofiltrate CC Chemokine 1 into Its Active [9–74] Processed Variant," *J. Immunol.*, vol. 167, no. 6, pp. 3406–3413, 2001, doi: 10.4049/jimmunol.167.6.3406.

[26] E. Komorowicz, K. Kolev, and R. Machovich, "Fibrinolysis with des-kringle derivatives of plasmin and its modulation by plasma protease inhibitors," *Biochemistry*, vol. 37, no. 25, pp. 9112–9118, 1998, doi: 10.1021/bi980180d.

[27] K. A. Of, P. Acyivation, I. X. Iuufild, P. Igudredi, and C. E. Pi-qjsiology, "Kinetic analysis of plasminogen activation by purified plasma kallikrein," no. c, 1985.

[28] I. Bally, V. Rossi, T. Lunardi, N. M. Thielens, C. Gaboriaud, and G. J. Arlaud, "Identification of the C1q-binding sites of human C1r and C1s. A refined three-dimensional model of the C1 complex of complement," *J. Biol. Chem.*, vol. 284, no. 29, pp. 19340–19348, 2009, doi: 10.1074/jbc.M109.004473.

[29] N. Zewde, R. D. Gorham, A. Dorado, and D. Morikis, "Quantitative modeling of the alternative pathway of the complement system," *PLoS One*, vol. 11, no. 3, pp. 1–26, 2016, doi: 10.1371/journal.pone.0152337.

[30] N. Zewde and D. Morikis, "A computational model for the evaluation of complement system regulation under homeostasis, disease, and drug intervention," *PLoS One*, vol. 13, no. 6, pp. 1–28, 2018, doi: 10.1371/journal.pone.0198644.

[31] N. T. Zewde, R. V. Hsu, D. Morikis, and G. Palermo, "Systems Biology Modeling of the Complement System Under Immune Susceptible Pathogens," *Front. Phys.*, vol. 9, no. April, pp. 1–15, 2021, doi: 10.3389/fphy.2021.603704.

[32] A. J. Perry *et al.*, "A molecular switch governs the interaction between the human complement protease C1s and its substrate, complement C4," *J. Biol. Chem.*, vol. 288, no. 22, pp. 15821–15829, 2013, doi: 10.1074/jbc.M113.464545.

[33] R. T. Kidmose *et al.*, "Structural basis for activation of the complement system by component C4 cleavage," *Proc. Natl. Acad. Sci. U. S. A.*, vol. 109, no. 38, pp. 15425–15430, 2012, doi: 10.1073/pnas.1208031109.

[34] A. Laich and R. B. Sim, "Complement C4bC2 complex formation: An investigation by surface plasmon resonance," *Biochim. Biophys. Acta - Protein Struct. Mol. Enzymol.*, vol. 1544, no. 1–2, pp. 96–112, 2001, doi: 10.1016/S0167-4838(00)00208-9.

[35] H. Chen *et al.*, "Allosteric inhibition of complement function by a staphylococcal immune evasion protein," *Proc. Natl. Acad. Sci. U. S. A.*, vol. 107, no. 41, pp. 17621–17626, 2010, doi: 10.1073/pnas.1003750107.

[36] D. V Pedersen *et al.*, "Functional and structural insight into properdin control of complement alternative pathway amplification," *EMBO J.*, vol. 36, no. 8, pp. 1084–1099, 2017, doi: 10.15252/embj.201696173.

[37] J. Bernet, J. Mullick, Y. Panse, P. B. Parab, and A. Sahu, "Kinetic Analysis of the Interactions between Vaccinia Virus Complement Control Protein and Human Complement Proteins C3b and C4b," *J. Virol.*, vol. 78, no. 17, pp. 9446–9457, 2004, doi: 10.1128/jvi.78.17.9446-9457.2004.

[38] A. A. Korotaevskiy, L. G. Hanin, and M. A. Khanin, "Non-linear dynamics of the complement system activation," *Math. Biosci.*, vol. 222, no. 2, pp. 127–143, 2009, doi: 10.1016/j.mbs.2009.10.003.

[39] C. D. C. Vidy, "A Quantitative Analysis of Binding and the Role of C4-binding Protein in Proteolysis of Cell-bound C4b," *Rockefeller Univ. Press*, vol. 157, pp. 1239–1251, 1983.

[40] R. J. Ziccardi, B. Dahlback, and H. J. Muller-Eberhard, "Characterization of the interaction of human C4b-binding protein with physiological ligands," *J. Biol. Chem.*, vol. 259, no. 22, pp. 13674–13679, 1984, doi: 10.1016/s0021-9258(18)89797-6.

[41] C. L. Harris, R. J. M. Abbott, R. A. Smith, B. P. Morgan, and S. M. Lea, "Molecular dissection of interactions between components of the alternative pathway of complement and decay accelerating factor (CD55)," *J. Biol. Chem.*, vol. 280, no. 4, pp. 2569–2578, 2005, doi: 10.1074/jbc.M410179200.

[42] T. B. Laboratories, “RATE PROCESSES IN THE FINAL STAGE OF COMPLEMENT HEMOLYSIS,” *Immunochemistry*, vol. 14, pp. 421–428, 1977, doi: 10.1016/0019-2791(77) 90167-7.

[43] M. K. Pangburn and H. J. Muller-Eberhardt, “The C3 convertase of the alternative pathway of human complement,” *Biochem. J*, vol. 235, no. 3, pp. 723–730, 1986.

[44] R. E. Lamerton *et al.*, “SARS-CoV-2 Spike- and Nucleoprotein-Specific Antibodies Induced After Vaccination or Infection Promote Classical Complement Activation,” *Front. Immunol.*, vol. 13, no. July, pp. 1–13, 2022, doi: 10.3389/fimmu.2022.838780.

[45] J. L. Beltrán and R. Franco, “The meaning of the Michaelis-Menten constant: Km describes a steady-state,” *bioRxiv*, pp. 1–16, 2019, doi: https://doi.org/10.1101/608232.

[46] N. Rawal and M. K. Pangburn, “Formation of high affinity C5 convertase of the classical pathway of complement,” *J. Biol. Chem.*, vol. 278, no. 40, pp. 38476–38483, 2003, doi: 10.1074/jbc.M307017200.

[47] J. Wu, Y. Q. Wu, D. Ricklin, B. J. C. Janssen, J. D. Lambris, and P. Gros, “Structure of complement fragment C3b-factor H and implications for host protection by complement regulators,” *Nat. Immunol.*, vol. 10, no. 7, pp. 728–733, 2009, doi: 10.1038/ni.1755.

[48] D. E. Hourcade, “The role of properdin in the assembly of the alternative pathway C3 convertases of complement,” *J. Biol. Chem.*, vol. 281, no. 4, pp. 2128–2132, 2006, doi: 10.1074/jbc.M508928200.

[49] L. B. Klickstein, S. F. Barbashov, T. Liu, R. M. Jack, and A. Nicholson-Weller, “Complement receptor type 1 (CR1, CD35) is a receptor for C1q,” *Immunity*, vol. 7, no. 3, pp. 345–355, 1997, doi: 10.1016/S1074-7613(00)80356-8.

[50] S. Bakshi *et al.*, “Mathematical Modelling of Alternative Pathway of Complement System,” *Bull. Math. Biol.*, vol. 82, no. 2, 2020, doi: 10.1007/s11538-020-00708-z.

[51] F. Teillet, B. Dublet, J. Andrieu, and C. Gaboriaud, “The Two Major Oligomeric Forms of Human Mannan-Binding Lectin: Chemical Characterization, Carbohydrate-Binding Properties, and Interaction with MBL-Associated Serine Proteases,” *J. Immunol.*, vol. 174, no. 5, pp. 2870–2877, 2005, [Online]. Available: https://doi.org/10.4049/jimmunol.174.5.2870%0D

[52] G. Ambrus *et al.*, “Natural Substrates and Inhibitors of Mannan-Binding Lectin-Associated Serine Protease-1 and -2: A Study on Recombinant Catalytic Fragments,” *J. Immunol.*, vol. 170, no. 3, pp. 1374–1382, 2003, doi: 10.4049/jimmunol.170.3.1374.

[53] V. Rossi, F. Teillet, N. M. Thielens, I. Bally, and G. J. Arlaud, “Functional characterization of complement proteases C1s/mannan-binding lectin-associated serine protease-2 (MASP-2) chimeras reveals the higher C4 recognition efficacy of the MASP-2 complement control protein modules,” *J. Biol. Chem.*, vol. 280, no. 51, pp. 41811–41818, 2005, doi: 10.1074/jbc.M503813200.

[54] T. H. Chao, J. A. Ember, M. Wang, Y. Bayon, T. E. Hugli, and R. D. Ye, “Role of the second extracellular loop of human C3a receptor in agonist binding and receptor function,” *J. Biol. Chem.*, vol. 274, no. 14, pp. 9721–9728, 1999, doi: 10.1074/jbc.274.14.9721.

[55] A. C. Dumitru *et al.*, “Submolecular probing of the complement C5a receptor–ligand binding reveals a cooperative two-site binding mechanism,” *Commun. Biol.*, vol. 3, no. 1, pp. 1–13, 2020, doi: 10.1038/s42003-020-01518-8.

[56] T. Sulikowski, B. A. Bauer, and P. A. Patston, “α1-Proteinase inhibitor mutants with specificity for plasma kallikrein and C1s but not C1,” *Protein Sci.*, vol. 11, no. 9, pp. 2230–2236, 2009, doi: 10.1110/ps.0207302.

[57] L. Bansal *et al.*, “Mathematical Modeling of Complement Pathway Dynamics for Target Validation and Selection of Drug Modalities for Complement Therapies,” *Front. Pharmacol.*, vol. 13, no. April, pp. 1–20, 2022, doi: 10.3389/fphar.2022.855743.

[58] M. Pascual, G. Steiger, J. Estreicher, K. Macon, J. E. Volanakis, and J. A. Schifferli, “Metabolism of complement factor D in renal failure,” *Kidney Int.*, vol. 34, no. 4, pp. 529–536, 1988, doi: 10.1038/ki.1988.214.

[59] J. D. Greenstein, P. W. Peake, and J. A. Charlesworth, “The kinetics and distribution of C9 and SC5b-9 in vivo: Effects of complement activation,” *Clin. Exp. Immunol.*, vol. 100, no. 1, pp. 40–46, 1995, doi: 10.1111/j.1365-2249.1995.tb03601.x.

[60] M. Oppermann and O. Götze, “Plasma clearance of the human C5a anaphylatoxin by binding to leucocyte C5a receptors.,” *Immunology*, vol. 82, no. 4, pp. 516–21, 1994, [Online]. Available: http://www.ncbi.nlm.nih.gov/pubmed/7835913%0Ahttp://www.pubmedcentral.nih.gov/articlerender.fcgi?artid=PMC1414921

[61] I. Ghiran, S. R. Tyagi, L. B. Klickstein, and A. Nicholson-Weller, “Expression and function of C1q receptors and C1q binding proteins at the cell surface,” *Immunobiology*, vol. 205, no. 4–5, pp. 407–420, 2002, doi: 10.1078/0171-2985-00142.

[62] U. Amara *et al.*, “Molecular Intercommunication between the Complement and Coagulation Systems,” *J. Immunol.*, vol. 185, no. 9, pp. 5628–5636, Nov. 2010, doi: 10.4049/jimmunol.0903678.

[63] A. Fletcher-Sandersjöö and B. M. Bellander, “Is COVID-19 associated thrombosis caused by overactivation of the complement cascade? A literature review,” *Thromb. Res.*, vol. 194, no. April, pp. 36–41, 2020, doi: 10.1016/j.thromres.2020.06.027.

[64] D. J. Sexton *et al.*, “Comparison Of Plasma Kallikrein Inhibition By The Endogenous C1-Inhibitor Versus DX-2930, a Monoclonal Antibody Inhibitor,” *Blood*, vol. 122, no. 21, pp. 1066–1066, 2013, doi: 10.1182/blood.v122.21.1066.1066.

[65] Ryan, Cooper, and Tauer, “Complement Regulator Factor H is a Cofactor for Thrombin in both Pro- and Anticoagulant Roles,” *Pap. Knowl. . Towar. a Media Hist. Doc.*, vol. 7, no. max 7, pp. 12–26, 2013.

[66] J. H. Foley *et al.*, “Complement Activation in Arterial and Venous Thrombosis is Mediated by Plasmin,” *EBioMedicine*, vol. 5, pp. 175–182, 2016, doi: 10.1016/j.ebiom.2016.02.011.

[67] N. Rawal and M. K. Pangburn, “Formation of High-Affinity C5 Convertases of the Alternative Pathway of Complement,” *J. Immunol.*, vol. 166, no. 4, pp. 2635–2642, 2001, doi: 10.4049/jimmunol.166.4.2635.

[68] E. M. Conway, “Reincarnation of ancient links between coagulation and complement,” *J. Thromb. Haemost.*, vol. 13, no. S1, pp. S121–

S132, 2015, doi: 10.1111/jth.12950.

[69] K. Ritis *et al.*, "A novel C5a receptor-tissue factor cross-talk in neutrophils links innate immunity to coagulation pathways.," *J. Immunol.*, vol. 177, no. 7, pp. 4794–802, Oct. 2006, doi: 10.4049/jimmunol.177.7.4794.

[70] E. Eldering, C. C. M. Huijbregts, Y. T. P. Lubbers, C. Longstaff, and C. E. Hack, "Characterization of recombinant C1 inhibitor P1 variants," *J. Biol. Chem.*, vol. 267, no. 10, pp. 7013–7020, 1992, doi: 10.1016/s0021-9258(19)50529-4.

[71] H. T. Cronjé, C. Nienaber-Rousseau, L. Zandberg, Z. De Lange, F. R. Green, and M. Pieters, "Fibrinogen and clot-related phenotypes determined by fibrinogen polymorphisms: Independent and IL-6-interactive associations," *PLoS One*, vol. 12, no. 11, pp. 1–18, 2017, doi: 10.1371/journal.pone.0187712.

[72] K. Hess, R. Ajjan, F. Phoenix, J. Dobó, P. Gál, and V. Schroeder, "Effects of MASP-1 of the complement system on activation of coagulation factors and plasma clot formation," *PLoS One*, vol. 7, no. 4, 2012, doi: 10.1371/journal.pone.0035690.

[73] V. Bumiller-Bini *et al.*, "Masps at the crossroad between the complement and the coagulation cascades-the case for covid-19," *Genet. Mol. Biol.*, vol. 44, no. 1, pp. 1–21, 2021, doi: 10.1590/1678-4685-gmb-2020-0199.

[74] L. Jenny, J. Dobó, P. Gál, and V. Schroeder, "MASP-1 Induced Clotting - The First Model of Prothrombin Activation by MASP-1," *PLoS One*, vol. 10, no. 12, pp. 1–13, 2015, doi: 10.1371/journal.pone.0144633.

[75] A. Fletcher-Sandersjöö, M. Maegele, and B. M. Bellander, "Does complement-mediated hemostatic disturbance occur in traumatic brain injury? A literature review and observational study protocol," *Int. J. Mol. Sci.*, vol. 21, no. 5, pp. 1–15, 2020, doi: 10.3390/ijms21051596.

[76] B. G. Kaira *et al.*, "Factor XII and kininogen asymmetric assembly with gC1qR/C1QBP/P32 is governed by allostery," *Blood*, vol. 136, no. 14, pp. 1685–1697, Jun. 2020, doi: 10.1182/blood.2020004818.

[77] Z. Bekassy, I. Lopatko Fagerström, M. Bader, and D. Karpman, "Crosstalk between the renin–angiotensin, complement and kallikrein–kinin systems in inflammation," *Nat. Rev. Immunol.*, vol. 22, no. 7, pp. 411–428, 2022, doi: 10.1038/s41577-021-00634-8.

[78] G. Wang, Y. Ye, X. Zhang, and J. Song, "Bradykinin stimulates IL-6 production and cell invasion in colorectal cancer cells," *Oncol. Rep.*, vol. 32, no. 4, pp. 1709–1714, 2014, doi: 10.3892/or.2014.3366.

[79] P. Conigliaro, P. Triggianese, C. Perricone, M. S. Chimenti, and R. Perricone, "COVID-19: disCOVering the role of complement system," *Clin Exp Rheumatol*, vol. 38, pp. 587–591, 2020.

[80] G. Oroszlán *et al.*, "MASP-1 and MASP-2 Do Not Activate Pro–Factor D in Resting Human Blood, whereas MASP-3 Is a Potential Activator: Kinetic Analysis Involving Specific MASP-1 and MASP-2 Inhibitors," *J. Immunol.*, vol. 196, no. 2, pp. 857–865, 2016, doi: 10.4049/jimmunol.1501717.

[81] A. G. Savitt *et al.*, "SARS-CoV-2 Exacerbates COVID-19 Pathology Through Activation of the Complement and Kinin Systems," *Front.*

Immunol., vol. 12, no. November, pp. 1–11, 2021, doi: 10.3389/fimmu.2021.767347.

[82] S. Kang *et al.*, “A SARS-CoV-2 antibody curbs viral nucleocapsid protein-induced complement hyperactivation,” *Nat. Commun.*, vol. 12, no. 1, pp. 1–11, 2021, doi: 10.1038/s41467-021-23036-9.

[83] B. Wang *et al.*, “Bivalent binding of a fully human IgG to the SARS-CoV-2 spike proteins reveals mechanisms of potent neutralization,” *bioRxiv*, p. 2020.07.14.203414, 2020, [Online]. Available: https://doi.org/10.1101/2020.07.14.203414

[84] S. Bondza *et al.*, “Complement-Dependent Activity of CD20-Specific IgG Correlates With Bivalent Antigen Binding and C1q Binding Strength,” *Front. Immunol.*, vol. 11, no. January, pp. 1–17, 2021, doi: 10.3389/fimmu.2020.609941.

[85] E. R. Kastenhuber *et al.*, “Coagulation factors directly cleave SARSCoV-2 spike and enhance viral entry,” *Elife*, vol. 11, 2022, doi: 10.7554/eLife.77444.

[86] H. Yu, E. M. Muñoz, R. E. Edens, and R. J. Linhardt, “Heparin regulation of the complement system,” *Chem. Biol. Heparin Heparan Sulfate*, no. 6, pp. 313–343, 2005, doi: 10.1016/B978-008044859-6/50012-5.

[87] D. Joshi, S. Manohar, G. Goel, S. Saigal, A. P. Pakhare, and A. Goyal, “Adequate Antithrombin III Level Predicts Survival in Severe COVID-19 Pneumonia,” *Cureus*, vol. 13, no. 10, 2021, doi: 10.7759/cureus.18538.

[88] J. Carvelli *et al.*, “Avdoralimab (Anti-C5aR1 mAb) Versus Placebo in Patients With Severe COVID-19: Results From a Randomized Controlled Trial (FOR COVID Elimination [FORCE])*,” *Crit. Care Med.*, vol. 50, no. 12, pp. 1788–1798, 2022, doi: 10.1097/CCM.0000000000005683.
