# Supplementary material for: Unravelling the impact of SARS-CoV-2 on hemostatic and complement systems: a systems immunology perspective
Source: Front Immunol. 2025 Jan 13;15:1457324. doi: 10.3389/fimmu.2024.1457324 (PMC11781117; doi:10.3389/fimmu.2024.1457324)
Supplement: Supplementary file 11 [file DataSheet11.pdf]

Table S2a. Average gene expression values of the complement and coagulation entities acquired from the analysis of microarray dataset (source: GEO accession GSE177477, Title: Type I interferon pathways in SARS-CoV-2-infected individuals from Pakistan)

S.no	Gene ID	Model genes symbol	Description of genes in the microarray expression dataset	Average symptomatic	Average normal
**1.**	TC0100007290.hg.1		complement C1q A chain (C1QA)[a]	5.766016649	5.441527489
**2.**	TC0100007292.hg.1		complement C1q B chain (C1QB)[a]	5.674930545	5.33326366
**3.**	TC0100007291.hg.1		complement C1q C chain (C1QC)[a]	6.578637501	5.931605926
		C1q	Assumed mean value of C1QA, C1QB and C1QC	6.006528232	5.568799025
**4.**	TC1200012744.hg.1	C1r	complement C1r (C1R)	2.789566056	2.717710458
**5.**	TC1200012592.hg.1	C1s	complement C1s (C1S)	3.067417806	3.050936823
**6.**	TC0600014105.hg.1	C2	complement C2	6.169682911	5.983513771
**7.**	TC1900009443.hg.1	C3	complement C3	5.205237102	5.051191456
**8.**	TC0600007628.hg.1	C4b	complement C4B (C4B)	5.484938717	5.703110477
**9.**	TC0600007628.hg.1	C4a	complement C4A (C4A)	5.484938717	5.703110477
**10.**	TC0900011394.hg.1	C5	complement C5	3.85969341	4.266834172
**11.**	TC0500010592.hg.1	C6	complement C6	4.003036475	4.082914683
**12.**	TC0500007238.hg.1	C7	complement C7	3.228319779	3.525506561
**13.**	TC0100008454.hg.1		complement C8 alpha chain (C8A)[a]	3.088029143	3.116979119
**14.**	TC0900009247.hg.1		complement C8 gamma chain (C8G)[a]	5.098091152	4.91679831
		C8	Assumed mean value of C8A and C8G	4.093060148	4.016888715
**15.**	TC0500013300.hg.1	C9	complement C9	3.455719368	3.02688555

**16.**	TC0100011397.hg.1		complement component 4 binding protein beta (C4BPB)[a]	2.729762065	2.491267509
**17.**	TC0100011402.hg.1		complement component 4 binding protein alpha(C4BPA)[a]	4.507298229	4.467124573
		C4BP	Assumed mean value of C4BPA and C4BPB	3.618530147	3.479196041
**18.**	TC0300013461.hg.1	MASP1	MBL associated serine protease 1	4.760726973	4.545585038
**19.**	TC0100012868.hg.1	MASP2	MBL associated serine protease 2	4.426790654	4.376543657
**20.**	TC0500012965.hg.1	MBL	lectin, mannose binding 2 (LMAN2)	8.665749334	8.527253909
**21.**	TC1200009807.hg.1	C3aR1	complement C3a receptor 1 (C3AR1)	7.705047159	6.761735891
**22.**	TC1900011770.hg.1	C5aR1	complement C5a receptor 1 (C5AR1)	10.16623494	9.970222126
**23.**	TC0100011064.hg.1	FH	complement factor H (CFH)	3.711950407	3.906269206
**24.**	TC0600014106.hg.1	FB	complement factor B (CFB)	2.768328985	2.455255566
**25.**	TC0400011580.hg.1	FI	complement factor I (CFI)	4.079502469	3.780938586
**26.**	TC1900006494.hg.1	PFD	complement factor D (CFD)	6.597460437	6.375797414
**27.**	TC0X00009580.hg.1	P	complement factor properdin (CFP)	7.061182572	7.366588348
**28.**	TC0100011406.hg.1	DAF	CD55 molecule/Decay accelerating factor (CD55)	11.44265642	11.27302089
**29.**	TC0100011414.hg.1	CR1	complement C3b/C4b receptor 1 (Knops blood group)	9.94664775	9.121356826
**30.**	TC0700006890.hg.1	IL-6	interleukin 6	3.578358241	2.921238268
**31.**	TC0100010078.hg.1	IL-6R	interleukin 6 receptor	7.927322874	9.081260049
**32.**	TC1400010563.hg.1	IgG	immunoglobulin heavy variable 5-78 (IGHV5-78) (pseudogene)	4.822236599	5.091758207
**33.**	TC1100007684.hg.1	C1-INH	serpin family G member 1; SERPING1 produce C1-INH	4.71138401	5.354422324
**34.**	TC1100013161.hg.1	CD59	CD59 molecule (CD59 blood group)	7.617894872	6.59121831

**35.**	TC0500012971.hg.1	F12	coagulation factor XII	5.956006529	5.967694807
**36.**	TC0400009551.hg.1	F11	coagulation factor XI	4.121960712	4.020726337
**37.**	TC1300008109.hg.1	F10	coagulation factor X	5.684616975	5.563305824
**38.**	TC0X00008578.hg.1	F9	coagulation factor IX	2.261295916	2.394016489
**39.**	TC1300008108.hg.1	F7	coagulation factor VII	6.213601721	5.955238006
**40.**	TC0X00008874.hg.1		coagulation factor VIII associated 2(F8A2)[a]	8.479164615	8.676219291
**41.**	TC0X00008874.hg.1		coagulation factor VIII associated 3(F8A3)[a]	8.479164615	8.676219291
**42.**	TC0X00008874.hg.1		coagulation factor VIII associated 1(F8A1)[a]	8.479164615	8.676219291
		F8	Assumed mean value of F8A1, F8A2 and F8A3	8.479164615	8.676219291
**43.**	TC0100016356.hg.1	F5	coagulation factor V	7.43315821	5.906725668
**44.**	TC1100007463.hg.1	F2	coagulation factor II, thrombin	5.153948356	5.053519954
**45.**	TC0100014988.hg.1	F3	coagulation factor III, tissue factor	4.979708717	4.298106767
**46.**	TC0400012181.hg.1	F1	fibrinogen alpha chain (FGA)	3.207277987	3.267204417
**47.**	TC0100016445.hg.1	AT3	serpin family C member 1 (SERPINC1)	2.803441006	2.663167435
**48.**	TC0200015194.hg.1	TFPI	tissue factor pathway inhibitor	4.791216244	3.120922171
**49.**	TC1200009847.hg.1	A2M	alpha-2-macroglobulin	4.553430733	5.167942675
**50.**	TC0600010085.hg.1	Pg	Plasminogen (PLG)	4.291519448	4.536142732
**51.**	TC1900011223.hg.1	KAL	Kallikrein (KLK)	4.858593207	4.774580567
**52.**	TC0400012875.hg.1	Pre-KAL	kallikrein B1 (KLKB1)	3.289896258	3.409488905

**53.**	TC0800010275.hg.1	tPA	plasminogen activator, tissue type (PLAT)	6.859149007	6.357348869
**54.**	TC1400010647.hg.1	B2R	bradykinin receptor B2 (BDKRB2)	4.155524838	4.046332648
**55.**	TC0300009777.hg.1	HK	High molecular weight kininogen (HK); Kininogen 1 (KNG1)	2.733984514	2.452658997
**56.**	TC0700008582.hg.1	PAI-1	Plasminogen activator inhibitor-1 known as Serpin family E member 1 belong to SERPINE1.	4.611072364	4.09542592

Table S1b. Concentration of the entities explored from experimental studies and estimation.

S. no	Model genes symbol	Description	Concentration
**1.**	CoV2S	Assay: S-Protein samples [S1], [S2] and [S3] = 5, 20, 80 ($\frac{\text{pg}}{\text{mL}}$), respectively. Consider average value for $[\text{CoV2S}]^b = 35\ \frac{\text{pg}}{\text{mL}}$ (Cai et al. 2021).	1.942855×10^{-9}
**2.**	CoV2N	Assay: N-Protein samples [N1], [N2] and [N3] = 8, 80, 800 ($\frac{\text{pg}}{\text{mL}}$), respectively. Consider average value for $[\text{CoV2N}]^b = 296\frac{\text{pg}}{\text{mL}} =$ (Cai et al. 2021).	1.643096×10^{-8}
**3.**	CoV2M	Assumption	1.0×10^{-12}
4.	CoV2E	Assumption	1.0×10^{-12}
5.	gC1qR	Range of concentration [gC1qR], 0.0 to 5.0 $\frac{\mu\text{g}}{\text{mL}}$ (Savitt et al. 2021). Assumed for $[\text{gC1qR}]^c = 2.5\frac{\mu\text{g}}{\text{mL}}$.	1.38775×10^{-7}
6.	TAFI	Normal values of [TAFI] is 1.76 to 28.9 $\frac{\text{ng}}{\text{mL}}$ (Nougier et al. 2020). Assumed for $[\text{TAFI}]^d = 15.345\frac{\text{ng}}{\text{mL}}$.	8.52×10^{-7}
7.	TAFIa	For Activated TAFI, $[\text{TAFIa}]^d = 60.2\frac{\text{ng}}{\text{mL}}$ (Nougier et al. 2020).	3.341702×10^{-6}
8.	Glu-Pg	The expression of Glu-Plasminogen [Glu-Pg] is taken same as the value for [Pg] available in the microarray expression data that is [Pg] = 4.291519448.	4.291519448
9.	Lys-Pg	Assumption for the expression of Lys-Plasminogen (Lys-Pg) based on the expression of Glu-Pg.	5.291519448
10.	BK	Assumption for the expression of BK based on the expression of bradykinin receptor B2 (B2R)	3.046332648

- **Mathematical calculation**

(b) [CoV2S] = $35\frac{\text{pg}}{\text{mL}} = 35\,\frac{\text{ng}}{\text{L}} = (35 \times 10^{-9})(0.05551)\frac{\text{Mol}}{\text{L}} = 1.942855 \times 10^{-9}$ M. Implies $35\,\frac{\text{pg}}{\text{mL}} = 1.942855 \times 10^{-9}$ M

[CoV2N] = $296\,\frac{\text{pg}}{\text{mL}} = 16.43096 \times 10^{-9}$ M.

(c) [gC1qR] = $2.5\,\frac{\mu\text{g}}{\text{mL}} = 0.138775 \times 10^{-6}$ M.

(d) [TAFI] = $15.345\,\frac{\text{ng}}{\text{mL}} = 15.345\,\frac{\mu\text{g}}{\text{L}} = 0.852 \times 10^{-6}$M.

[TAFIa] = $60.2\,\frac{\text{ng}}{\text{mL}} = 60.2\,\frac{\mu\text{g}}{\text{L}} = 3.341702 \times 10^{-6}$M.

- As, 1g = 0.05551 mol.
- $\frac{\text{Mol}}{\text{L}}$ can be taken in molarity (M).

References

Cai, Qiyong, Jingjing Mu, Yang Lei, Jia Ge, Aaron Albert Aryee, Xiaoge Zhang, and Zhaohui Li. 2021. "Simultaneous Detection of the Spike and Nucleocapsid Proteins from SARS-CoV-2 Based on Ultrasensitive Single Molecule Assays." *Analytical and Bioanalytical Chemistry* 413 (18): 4645–54. https://doi.org/10.1007/s00216-021-03435-z.

Nougier, Christophe, Remi Benoit, Marie Simon, Helene Desmurs-Clavel, Guillaume Marcotte, Laurent Argaud, Jean Stephane David, Aurelie Bonnet, Claude Negrier, and Yesim Dargaud. 2020. "Hypofibrinolytic State and High Thrombin Generation May Play a Major Role in SARS-COV2 Associated Thrombosis." *Journal of Thrombosis and Haemostasis* 18 (9): 2215–19. https://doi.org/10.1111/jth.15016.

Savitt, Anne G., Samantha Manimala, Tiara White, Marina Fandaros, Wei Yin, Huiquan Duan, Xin Xu, et al. 2021. "SARS-CoV-2 Exacerbates COVID-19 Pathology Through Activation of the Complement and Kinin Systems." *Frontiers in Immunology* 12 (November): 1–11. https://doi.org/10.3389/fimmu.2021.767347.
