# Supplementary material for: Unravelling the impact of SARS-CoV-2 on hemostatic and complement systems: a systems immunology perspective
Source: Front Immunol. 2025 Jan 13;15:1457324. doi: 10.3389/fimmu.2024.1457324 (PMC11781117; doi:10.3389/fimmu.2024.1457324)
Supplement: Supplementary file 12 [file DataSheet12.pdf]

Table S3. In treatment state, available drugs, along with their targets.

Drug	Doses	Start time (min)	Amount (mol/L)	Rate (k)	Interval	Repeated count	Target
Avdoralimab	Dose-1	0	0.3339	0.01	1200	3	C5aR1
	Dose-2	1440	1.017	0.01	1200	3	
Tranexamic acid	Dose-1	0	0.69	0.2	1500	7	tPA
	Dose-2	720	1.40	0.2	1500	7	
	Dose-3	1440	6.86	0.2	1500	7	
Heparin	Dose-1	0	2.325	0.2	1200	7	C2-9
	Dose-2	180	4.650	0.2	1200	7	
	Dose-3	360	9.300	0.2	1200	7	
Heparin	Dose-1	0	0.561	1	1200	3	AT3
	Dose-2	120	2.8034	1	1200	3	
	Dose-3	240	14.017	1	1200	3	
	Dose-4	360	19.62	1	1200	3	
Garadicimab	Dose-1	0	0.66	3	500	2	KAL:F12
	Dose-2	0	3.3	3	500	2	
Tocilizumab	Dose-1	0	1.5855	0.01	600	3	IL-6R
	Dose-2	120	7.9273	0.01	600	3	
