# Supplementary material for: Unravelling the impact of SARS-CoV-2 on hemostatic and complement systems: a systems immunology perspective
Source: Front Immunol. 2025 Jan 13;15:1457324. doi: 10.3389/fimmu.2024.1457324 (PMC11781117; doi:10.3389/fimmu.2024.1457324)
Supplement: Supplementary file 13 [file DataSheet13.pdf]

Supplementary Material

ODE-BASED MODELING FRAMEWORK

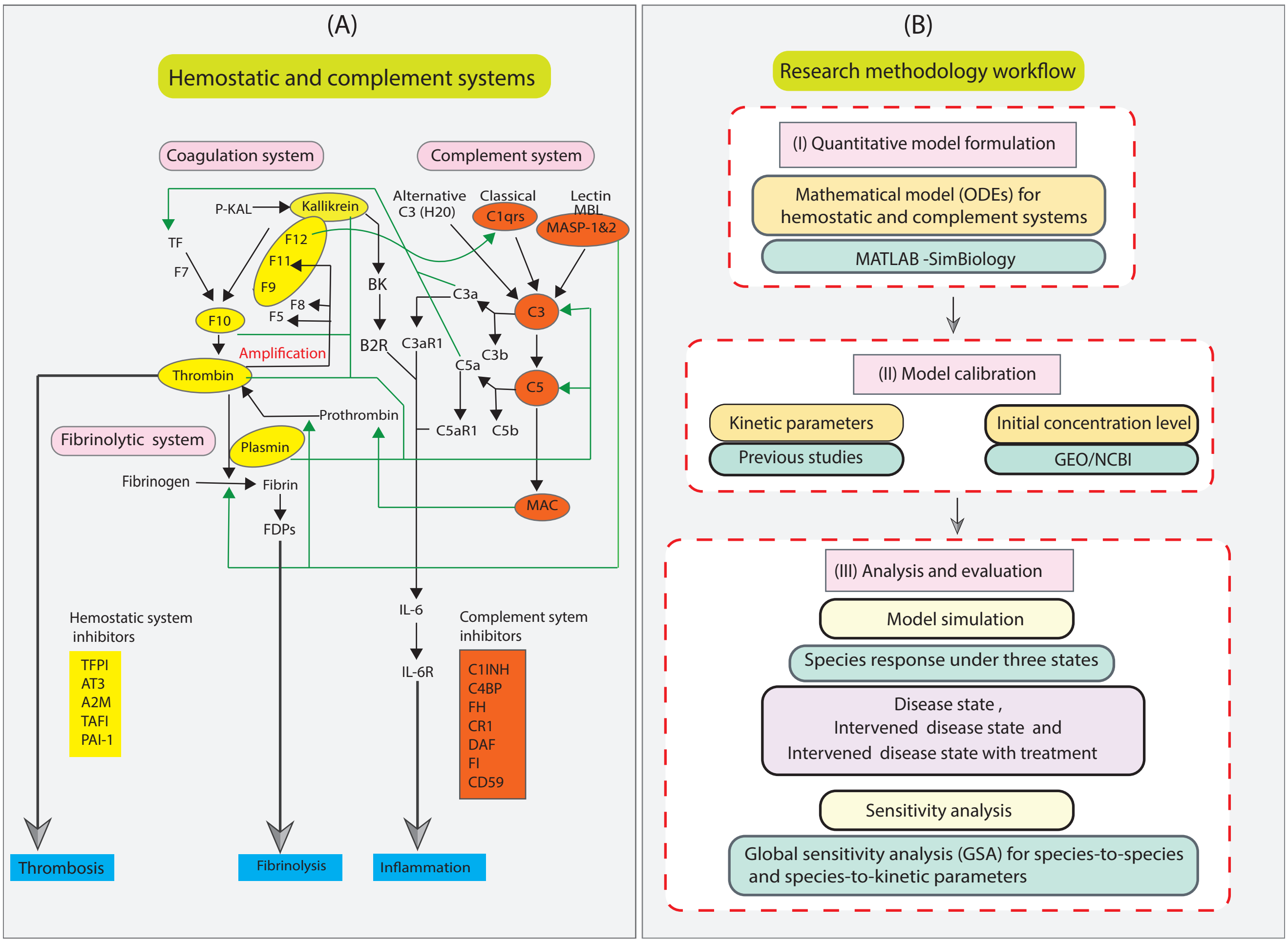

Figure S1. Study workflow. (A) The computational modeling and analysis start by examining the hemostatic system, which includes the coagulation and fibrinolytic pathways, as well as the complement system, consisting of the alternative, classical, and lectin pathways. This information is gathered from the KEGG database and related literature. Refer to the complete SARS-CoV-2-induced regulated hemostatic and complement systems in **Fig. 1**. (B) A quantitative model based on ordinary differential equations (ODEs) is developed using MATLAB-SimBiology. The model is further calibrated with kinetic parameters obtained from literature and estimation. Initial concentration levels (initial conditions) of entities are derived from microarray gene expression data (data source: GEO/NCBI), with some values adopted from previous experimental studies. The model simulates the concentration-time profiles of entities under three conditions: the disease state, the intervened disease state (with perturbations), and the treatment state (the intervened disease state with treatment). The disease state represents an unhealthy, symptomatic COVID-19 condition. Perturbations account for impairments caused by decreased levels of FH, C1INH, TFPI, and A2M. The treatment state incorporates drug interventions. Global sensitivity analysis (GSA) is performed to evaluate species-to-species interactions and the effects of kinetic parameters.
