# Supplementary material for: Unravelling the impact of SARS-CoV-2 on hemostatic and complement systems: a systems immunology perspective
Source: Front Immunol. 2025 Jan 13;15:1457324. doi: 10.3389/fimmu.2024.1457324 (PMC11781117; doi:10.3389/fimmu.2024.1457324)
Supplement: Supplementary file 14 [file DataSheet14.pdf]

frontiers

Supplementary Material

DUMMY MATHEMATICAL MODEL

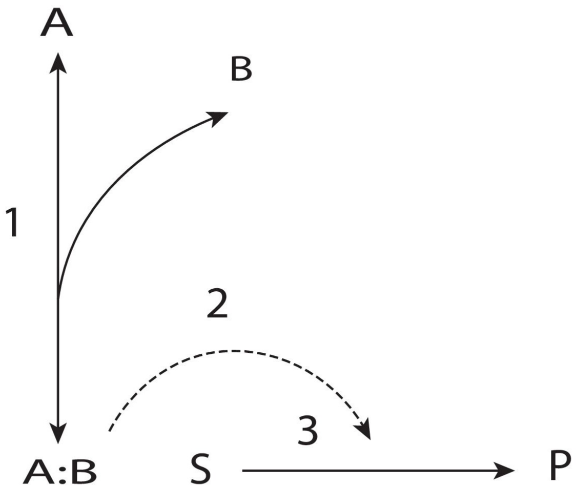

Figure S1. Reaction network diagram of dummy mathematical model.

$$\frac{d(A)}{dt} = -[k^{+}] * A * B + [k^{-}] * AB$$
$$\frac{d(B)}{dt} = -[k^{+}] * A * B + [k^{-}] * AB$$
$$\frac{d(AB)}{dt} = [k^{+}] * A * B - [k^{-}] * AB$$
$$\frac{d(S)}{dt} = -\frac{[k_{catS}^{AB}] * S * AB}{([k_{mS}^{AB}] + S)}$$
$$\frac{d(P)}{dt} = \frac{[k_{catS}^{AB}] * S * AB}{([k_{mS}^{AB}] + S)}$$

Assumed species A, B represent genes/proteins, and AB their complex. S and P indicate substrate and product. By kinetic law of mass action suppose association rate (k^{+}) and disassociation rate (k^{-}).

Assumed, AB cleaved substrate S, the cleavage rate is represented by $\frac{[k_{catS}^{AB}]*S*AB}{([k_{mS}^{AB}]+S)}$. Where k_{catS}^{AB} implies catalytic rate and k_{mS}^{AB} implies Michaelis constants such that $k_{mS}^{AB} = \frac{k^{-}+k_{catS}^{AB}}{k^{+}}$, disassociation constant $k_d = \frac{k^{-}}{k^{+}}$ (Liu et al., 2011).

REFERENCES

Liu, B., Zhang, J., Tan, P. Y., Hsu, D., Blom, A. M., Leong, B., et al. (2011). A computational and experimental study of the regulatory mechanisms of the complement system. *PLoS Computational*

Biology 7, e1001059. doi:10.1371/journal.pcbi.1001059
