# Supplementary material for: Unravelling the impact of SARS-CoV-2 on hemostatic and complement systems: a systems immunology perspective
Source: Front Immunol. 2025 Jan 13;15:1457324. doi: 10.3389/fimmu.2024.1457324 (PMC11781117; doi:10.3389/fimmu.2024.1457324)
Supplement: Supplementary file 15 [file DataSheet15.pdf]

%%

Range of kinetic rate constants used in global sensitivity analysis for F2a.

%%

**Use**	**Component Name**			**Current Value**
true	[k^F10a_catF2]			7500
	Distribution	=	Uniform	
	Lower =	6750		
	Upper =	8250		
true	[k^MASP1_catF2]			141000
	Distribution	=	Uniform	
	Lower =	126900		
	Upper =	155100		
true	[k^MASP2_catF2]			141000
	Distribution	=	Uniform	
	Lower =	126900		
	Upper =	155100		
true	[k^+_F2aA2M]			2500
	Distribution	=	Uniform	
	Lower =	2250		
	Upper =	2750		
true	[k^+_F2aAT3]			7100
	Distribution	=	Uniform	
	Lower =	6390		
	Upper =	7810.000000000001		
true	[k^F2a_catCoV2S]			0.00001634
	Distribution	=	Uniform	
	Lower =	0.000014706		
	Upper =	0.000017974		
true	[k^F2a_mCoV2S]			0.052

	Distribution	=	Uniform
	Lower =	0.0468	
	Upper =	0.0572	
true	[k^+_F11F2a]		100000000
	Distribution	=	Uniform
	Lower =	90000000	
	Upper =	110000000.00000001	
true	[k^-_F11F2a]		5
	Distribution	=	Uniform
	Lower =	4.5	
	Upper =	5.5	
true	[k^F11_catF2a]		0.00013
	Distribution	=	Uniform
	Lower =	0.000117	
	Upper =	0.000143	
true	[k^+_C1INHF2a]		100
	Distribution	=	Uniform
	Lower =	90	
	Upper =	110.00000000000001	
true	[k^+_FHF2a]		33670000
	Distribution	=	Uniform
	Lower =	30303000	
	Upper =	37037000	
true	[k^+_F2aF1]		11700000
	Distribution	=	Uniform
	Lower =	10530000	
	Upper =	12870000.000000002	
true	[k^-_F2aF1]		84
	Distribution	=	Uniform

Lower = 75.60000000000001

Upper = 92.4

%%%

Range of kinetic rate constants used in global sensitivity analysis for F11a.

%%%

Use Component Name Current Value

true [k^F12a_catF11a] 0.0057

Distribution = Uniform

Lower = 0.00513

Upper = 0.00627

true [k^+_F11aF9] 100000000

Distribution = Uniform

Lower = 90000000

Upper = 110000000.00000001

true [k^-_F11aF9] 41

Distribution = Uniform

Lower = 36.9

Upper = 45.1

true [k^+_F11aAT3] 320

Distribution = Uniform

Lower = 288

Upper = 352

true [k^F11a_catF9a] 7.7

Distribution = Uniform

Lower = 6.930000000000001

Upper = 8.47

true [k^F11_catF2a] 0.00013

Distribution = Uniform

Lower = 0.000117

Upper = 0.000143

true [k^+_F11aC1INH] 1800

Distribution = Uniform

Lower = 1620

Upper = 1980.0000000000002

true [k^HKF11_catF11a] 0.1

Distribution = Uniform

Lower = 0.09000000000000001

Upper = 0.11000000000000001

%%%

Range of kinetic rate constants used in global sensitivity analysis for F12a.

%%%

Use Component Name Current Value

true [k^+_F12F12a] 100000000

Distribution = Uniform

Lower = 90000000

Upper = 110000000.00000001

true [k^-_F12F12a] 750

Distribution = Uniform

Lower = 675

Upper = 825.0000000000001

true [k^+_F12aAT3] 21.6

Distribution = Uniform

Lower = 19.44

Upper = 23.760000000000005

true [k^+_F12aF11] 100000000

Distribution = Uniform

Lower = 90000000

Upper = 110000000.00000001

true [k^-_F12aF11] 200

Distribution = Uniform

Lower = 180

Upper = 220.00000000000003

true [k^KAL_catF12a] 5.7

Distribution = Uniform

Lower = 5.13

Upper = 6.2700000000000005

true [k^F12a_catC1r] 100

Distribution = Uniform

Lower = 90

Upper = 110.00000000000001

true [k^+_C1INHF12a] 3600

Distribution = Uniform

Lower = 3240

Upper = 3960.0000000000005

true [k^F12a_catF11a] 0.0057

Distribution = Uniform

Lower = 0.00513

Upper = 0.00627

true r177.[k^F12F12a_catF12a] 0.033

Distribution = Uniform

Lower = 0.0297

Upper = 0.036300000000000006

true [k^+_CoV2SF12] 1000000

Distribution = Uniform

Lower = 900000

Upper = 1100000

true [k^+_CoV2NF12] 10000

Distribution = Uniform

Lower = 9000

Upper = 11000

true [k^+_CoV2MF12] 10000

Distribution = Uniform

Lower = 9000

Upper = 11000

true [k^+_CoV2EF12] 10000

Distribution = Uniform

Lower = 9000

Upper = 11000

true [k^Pn_catF12] 1000

Distribution = Uniform

Lower = 900

Upper = 1100

true [k^+_PKALF12a] 100000000

Distribution = Uniform

Lower = 90000000

Upper = 110000000.00000001

true [k^-_PKALF12a] 3600

Distribution = Uniform

Lower = 3240

Upper = 3960.0000000000005

true [k^gC1qRF12_catF12a] 0.1

Distribution = Uniform

Lower = 0.09000000000000001

Upper = 0.11000000000000001

%%

Range of kinetic rate constants used in global sensitivity analysis for Pn

%%

Use	Component Name		Current Value
true	[k^+_C1INHPn]		5500
	Distribution =	Uniform	
	Lower =	4950	
	Upper =	6050.000000000001	
true	[k^KAL_catGlu-Pg]		0.00016
	Distribution =	Uniform	
	Lower =	0.000144	
	Upper =	0.00017600000000000002	
true	[k^KAL_mGlu-Pg]		5.6e-7
	Distribution =	Uniform	
	Lower =	5.040000000000001e-7	
	Upper =	6.160000000000001e-7	
true	[k^tPA_catGlu-Pg]		4.1e-7
	Distribution =	Uniform	
	Lower =	3.69e-7	
	Upper =	4.51e-7	
true	[k^tPA_mGlu-Pg]		0.073
	Distribution =	Uniform	
	Lower =	0.0657	
	Upper =	0.0803	
true	[k^Pn_catLys-Pg]		0.1
	Distribution =	Uniform	
	Lower =	0.09000000000000001	
	Upper =	0.11000000000000001	

%%

Range of kinetic rate constants used in global sensitivity analysis for FDPs

%%

**Use**	**Component Name**			**Current Value**
true	[k^+_Glu-PgFDPs]			571000
	Distribution	=	Uniform	
	Lower =	513900		
	Upper =	628100		
true	[k^-_Glu-PgFDPs]			0.1
	Distribution	=	Uniform	
	Lower =	0.09000000000000001		
	Upper =	0.11000000000000001		
true	[k^+_Lys-PgFDPs]			1110000
	Distribution	=	Uniform	
	Lower =	999000		
	Upper =	1221000		
true	[k^-_Lys-PgFDPs]			0.1
	Distribution	=	Uniform	
	Lower =	0.09000000000000001		
	Upper =	0.11000000000000001		
true	[k^Pn_catF1a]			6.4
	Distribution	=	Uniform	
	Lower =	5.760000000000001		
	Upper =	7.040000000000001		
true	[k^Pn_mF1a]			1.4e-7
	Distribution	=	Uniform	
	Lower =	1.2600000000000002e-7		
	Upper =	1.5400000000000003e-7		

%%

Range of kinetic rate constants used in global sensitivity analysis for IL-6

%%%

**Use**	**Component Name**			**Current Value**
true	[k^C3aC3aR1_catIL6]			0.1
	Distribution	=	Uniform	
	Lower	=	0.09000000000000001	
	Upper	=	0.11000000000000001	
true	[k^C5aC5aR1_catIL6]			0.1
	Distribution	=	Uniform	
	Lower	=	0.09000000000000001	
	Upper	=	0.11000000000000001	
true	[k^BKB2R_catIL6]			0.1
	Distribution	=	Uniform	
	Lower	=	0.09000000000000001	
	Upper	=	0.11000000000000001	
true	[k^-_dIL6]			0.01
	Distribution	=	Uniform	
	Lower	=	0.09000000000000001	
	Upper	=	0.11000000000000001	
